# Effectiveness of Interventions to Reduce Carbon‐Emissions Within Secondary Healthcare: Systematic Review and Evidence and Gap Map

**DOI:** 10.1002/cl2.70077

**Published:** 2025-12-23

**Authors:** Liz Shaw, Noreen Orr, Hassanat M. Lawal, Simon Briscoe, Xiaoyu Yan, Jo Thompson Coon, G. J. Melendez‐Torres, Clara Martin‐Pintado, Ruth Garside

**Affiliations:** ^1^ Exeter Policy Research Programme Evidence Review Facility, University of Exeter Medical School, Faculty of Health and Life Sciences, St Luke's Campus University of Exeter Exeter UK; ^2^ Environment and Sustainability Institute, Faculty of Environment, Science and Economy, Penryn Campus University of Exeter Penryn Cornwall UK; ^3^ European Centre for Environment and Human Health, University of Exeter Medical School University of Exeters Penryn Cornwall UK

## Abstract

‘The climate emergency is also a health emergency’ (England 2024). Climate change directly impacts the health of the human population through events such as earthquakes, flooding, heatwaves and drought, which increase the risk of injury, displacement, disruption of food supplies, infectious diseases and mental ill health (England 2024; Lenzen et al. 2020; Tennison et al. 2021; The Lancet Respiratory Medicine 2023). The impact on population health of these climate events, alongside indirect health consequences such as increased prevalence of respiratory conditions due to air pollution, places an increased burden on health services (Royal College of Physicians 2017). The environmental footprint of healthcare services contributes between 1% and 5% towards total global environmental impacts (Lenzen et al. 2020; Tennison et al. 2021). Reducing the impact of the healthcare system on climate change has the potential to benefit population health through improved air quality and diet, and increased activity levels (Mailloux et al. 2021). Due to the lack of systematic reviews which consider carbon emissions associated with the patient pathway within individual specialities, further research is needed to enable the identification and transformation of the most carbon‐intensive clinical pathways, while ensuring future models of care can be delivered in a cost‐effective manner without increasing emissions or compromising patient care. In 2008, the Climate Change Act set national targets for the 100% reduction of carbon emissions in England to 1990 levels by 2050 (‘Climate Change Act’ 2008). Within the United Kingdom, the National Health Service (NHS) has an important role in helping to achieve these targets, as the organisation accounts for 4% of England's carbon footprint (NHS England 2022). The UK government's Greener NHS team from NHS England asked the Exeter Policy Research Programme Evidence Review Facility to bring together and analyse research which assesses different ways carbon emissions resulting from hospital‐led care can be reduced, without affecting the care patients receive in hospitals, at home and in clinics. Work focusing on identifying and delivering interventions to reduce carbon emissions within known carbon hotspots, such as NHS estates and facilities, travel and transport, supply chain, and certain medicines and medical and anaesthetic gases that have high global warming potential is already underway, alongside examining the effectiveness of different models of care delivery across all specialities (NHS England 2022; NHS Shared Business Services 2022). Evidence focusing on the effectiveness of interventions in reducing carbon emissions within secondary healthcare would be a useful complement to this work. An approach which considers the patient pathway may be beneficial in identifying interventions which consider wider healthcare systems and thus have a meaningful impact on reducing carbon emissions. This review was commissioned by the Greener NHS team and could serve as a useful case study for wider net‐zero ambitions elsewhere in the world. We aimed to carry out a systematic review examining the effectiveness of interventions in reducing the carbon footprint within specific medical specialities in secondary healthcare and explored where this evidence could inform the patient care pathway. In July 2023, we searched a selection of bibliographic databases with coverage of both health care and environmental science journals, including MEDLINE, Embase and Environment Complete, which we supplemented by inspecting the HealthcareLCA database, conducting forwards and backwards citation chasing on all studies which met our inclusion criteria, searching reference lists of topically relevant reviews, and searching Google Scholar and a selection of relevant websites. We included studies using any comparative study design evaluating any intervention intended to reduce the environmental impact of a procedure, process, treatment, or pathway delivered within secondary healthcare in the following specialities: cardiology, gastroenterology, obstetrics, oncology, ophthalmology, orthopaedics and trauma, radiology, renal, respiratory and high volume, low complexity surgeries (specifically: ear, nose, and throat [ENT], gynaecology and urology). We extracted descriptive data regarding study sample, intervention/control group, carbon emission methodology, PROGRESS‐PLUS criteria (related to equity) and environmental, patient and cost outcomes. We appraised the quality of studies using life cycle assessment (LCA) methods with a predetermined scoring system informed by Weidema's (1997) guidelines (B.P.W. 1997). We synthesised findings from studies drawing on LCA methods and non‐LCA studies separately using narrative synthesis. Within each group, studies were clustered into five broad intervention categories: (1) Accessing care, (2) Product level, (3) Care delivery, (4) Setting and (5) Multiple components. We examined and explained patterns across studies within the same speciality which evaluated similar interventions. We also developed an evidence and gap map (EGM) to highlight where evidence relevant to the review aims could inform a generic patient care pathway for each speciality and future research on lower carbon pathways. Input from the Greener NHS team at NHS England, LCA methods experts and patient and public representatives was incorporated throughout. Eighty‐eight studies (92 articles) met eligibility criteria, 28 used LCA‐informed methods to calculate carbon emissions (19 of these utilised a full LCA approach). Of the LCA studies, 9 were of Low risk of bias, 14 of Medium risk of bias and 5 of High risk of bias. Urology (*n* = 14), gastroenterology (*n* = 13), oncology/radiation oncology (*n* = 13) and renal (*n* = 11) were the most common specialities represented. Across different specialities, most evidence was found in the first three stages of the patient care pathway (Initial assessment/diagnostic tests, initial treatment, or routine follow‐up). The exception was the renal speciality, where most evidence was within ‘Ongoing care’ segment. There was limited evidence within the ‘Discharge’ segment of the care pathway across all specialities. Evidence relating to the wider healthcare setting was clustered within gastroenterology (*n* = 5) and radiology specialities (*n* = 5). The two largest groups of evidence were for studies evaluating telehealth (*n* = 26) and reuseable equipment (*n* = 13) interventions. Telehealth interventions were predominantly evaluated using non‐LCA methods (*n* = 23). While carbon‐emissions favoured telemedicine interventions versus face‐to‐face care, these calculations often only considered patient‐travel saved and did not account for carbon emissions associated with other parts of the delivery of the service, such as digital technology used or energy use of building or clinic equipment for face‐to‐face appointments, or wider impact on the patient care pathway such as potential need to travel for additional primary care appointments. The majority of patient and cost outcomes favoured telemedicine interventions, although most were based on non‐statistical analyses. Interventions comparing carbon emissions associated with the use of reuseable versus disposable surgical equipment represented the largest group of studies using LCA methods. For studies within gastroenterology, reuseable equipment was associated with reduced carbon emissions. Within urology, this finding was reversed, although questions regarding the accuracy of use of characterisation factors, quantity of materials used in disposable versus reuseable equipment packs and how carbon emissions were assigned to the reprocessing of reuseable equipment mean confidence in this finding is uncertain. While waste management/reduction interventions were associated with reduced carbon emissions, interventions were highly heterogeneous, with limited consideration of patient or cost outcomes. Eight non‐LCA studies found that reduced carbon emissions were associated with energy conservation interventions, such as turning equipment off when not in use or choosing imaging techniques with lower energy use, the majority of which were conducted within radiology/radiotherapy settings. This systematic review synthesises quantitative evidence evaluating the effectiveness of interventions intended to reduce carbon emissions within high‐volume secondary healthcare specialities. It highlights a highly heterogeneous evidence base, and the methodological limitations associated with studies based on LCA and non‐LCA methods. While we identified several large clusters of studies evaluating similar interventions within the same speciality, future research needs to address these methodological limitations to support confident decision‐making within policy commissioning and clinical practice. Our EGM displays the included evidence according to individual speciality along the patient pathway, enabling evidence users to identify research which meets their requirements as well as identifying potential gaps where further research may be required.

**Abbreviations**
DHSC: Department of Health and Social Care
EGM: evidence and gap map
LCA: life cycle assessment
NHS: National Health Service
NHSE: National Health Service England
PHE: Public Health England
PPE: personal protective equipment
PRISMA: Preferred Reporting Items for Systematic Reviews and Meta‐Analyses
PRP: Policy Research Programme
PT: physio or physical therapist

## 1 Plain Language Summary

Strategies to reduce carbon use in hospital‐based healthcare generally seem to be effective; however, there are limitations in the methods used to assess this and gaps in the evidence about treatment across the patient pathway.

### 1.1 Background

The National Health Service (NHS) in England wants to reduce the amount of carbon they produce because it currently contributes 4% to England's overall carbon emissions.

### 1.2 What We Want to Know?

The UK government's Greener NHS team from NHS England asked the Exeter Policy Research Programme Evidence Review Facility to bring together and analyse research which assesses different ways carbon emissions resulting from hospital‐led care can be reduced, without affecting the care patients receive in hospitals, at home and in clinics.

#### 1.2.1 What Did We Do?

We looked for research related to medical specialities with high levels of inpatient activity, as these are likely to have the greatest impact on carbon emissions. These were:

- Cardiology
- Gastroenterology
- Ophthalmology
- Orthopaedics and trauma
- Renal
- Respiratory
- High volume, low complexity surgery – specifically:
  ∘ Ear, nose, and throat (ENT)
  ∘ Gynaecologist
  ∘ Urology

#### 1.2.2 What Studies Are Included?

We were only interested in studies which compared a group of patients who received an intervention with a group of patients who did not. This included randomised controlled trials (RCTs), cohort and before‐and‐after studies.

Studies had to have measured how an intervention affected carbon emissions. We also looked for other outcomes such as:

– Cost‐effectiveness,
– Patient outcomes (health outcomes, safety, satisfaction),
– Other environmental impacts, such as water or air pollution.

#### 1.2.3 What Are the Main Findings of This Review and Evidence and Gap Map (EGM)?

We included 88 studies in our review. 33 were carried out in the United Kingdom.

The most common types of strategies assessed were ones focusing on delivering care online or ones which replaced disposable surgical equipment with reuseable equipment.

The most common specialities in which research was conducted were:

- Urology: 14
- Gastroenterology: 13
- Oncology: 13
- Renal: 11

We showed how this evidence could inform the patient care pathway in an EGM.

Most evidence was found in the first three stages of the patient care pathway (Initial assessment/diagnostic tests, initial treatment or follow‐up).

The two largest groups of evidence were for studies evaluating telehealth (*n* = 26) and reuseable equipment (*n* = 12) interventions.

#### 1.2.4 What Do the Findings of the Review and EGM Mean?

If you try to reduce carbon emissions, it generally works:

- Telehealth: Reduces carbon emissions by reducing the distance patients or staff need to travel,
- Turning equipment off saves energy – most common in radiology settings,
- Can reduce waste (and thus carbon emissions) through:
  ∘ reducing packaging on equipment used for surgery.
  ∘ reducing information sent to patients on paper.
  ∘ increasing recycling of waste produced in operating theatres.

However, there were several flaws in the way carbon emissions were measured:

- Not many of the studies took a ‘Life Cycle Assessment’ approach to measuring carbon emissions,
- Lots of studies excluded important things from their calculations of carbon emissions, for example, heating and electricity, staff transport.

Although some controversy about whether reuseable or disposable equipment is better for reducing carbon emissions in Urology surgery. For other types of surgery, reuseable equipment is linked with lower carbon emissions.

#### 1.2.5 How Up‐to‐Date Is This EGM?

The authors searched for evidence published from 2008 to 12th July 2023.

## 2 Background

### 2.1 Description of the Problem

‘The climate emergency is also a health emergency’ (England 2024). Climate change directly impacts the health of the human population through events such as earthquakes, flooding, heatwaves and drought, which increase the risk of injury, displacement, disruption of food supplies, infectious diseases and mental ill health (England 2024; Lenzen et al. 2020; Tennison et al. 2021; The Lancet Respiratory Medicine 2023). The impact on population health of these climate events, alongside indirect health consequences such as increased prevalence of respiratory conditions due to air pollution, places an increased burden on health services (Royal College of Physicians 2017). This health burden disproportionately impacts populations of lower‐income countries, despite contributing a lower proportion to global greenhouse gas (GHG) emissions than developed countries (Bhargawa Ruma 2023; Levy and Patz 2015; Schöngart et al. 2025), in part due to increased exposure risk of exposure to climate events, fewer resources available to support adaptation (Schöngart et al. 2025), and exacerbating existing barriers to accessing health services (Martins et al. 2024; Naser et al. 2024). Concerns have been raised regarding whether high‐income countries are doing enough to meet decarbonisation obligations agreed to as part of the Paris Agreement 2015 (United Nations (Paris Agreement) 2015; Vogel and Hickel 2023; Barratt 2022; Brownlee et al. 2017; Lenzen et al. 2020; Or and Seppänen 2024).

### 2.2 Why It Is Important to Do This Review

While necessary for improving and maintaining human well‐being, the environmental footprint of healthcare services contributes between 1% and 5% towards total global environmental impacts (Lenzen et al. 2020; Tennison et al. 2021). If the healthcare system were a country, it would be the fifth largest producer of GHGs in the world (Barratt 2022), with contributions towards climate change expected to increase as demand for care increases worldwide (Or and Seppänen 2024).

Reducing the impact of the healthcare system on climate change has the potential to benefit population health through improved air quality and diet and increased activity levels (Mailloux et al. 2021). Within UK healthcare, common sources of carbon emissions between 1990 and 2019 included supply chains (62%), and other sources including delivery of care (24%), travel to and from sites by staff, patients and visitors (10%) and private health and care services commissioned by the NHS (4%) (Tennison et al. 2021). In addition to these, other factors contributing to the environmental footprint of global health services include the overuse of medical services, such as unnecessary tests or treatments (Brownlee et al. 2017), and heat and electricity use (Lenzen et al. 2020). These environmental hotspots provide opportunities for targeted interventions to reduce the climate impact of health services.

Within the United Kingdom, the NHS has exceeded its commitments under the Climate Change Act by reducing its carbon footprint by 30% (Climate Change Act 2008; England 2024). The NHS England Greener NHS, alongside the Primary Care and Medicines policy teams, have been working closely with patients, clinicians and industry to minimise emissions from medicines and anaesthetic gases, reducing waste, ensuring the right medicines are available to patients, and finding mechanisms to support shared, informed decision making (NHS England 2022). Other work includes focusing on identifying and delivering interventions to reduce carbon emissions within known carbon hotspots, such as estates and facilities, travel and transport, supply chain, and certain medicines and medical and anaesthetic gases that have high global warming potential is already underway, alongside examining the effectiveness of different models of care delivery across all specialities, to enable safe, patient‐centred lower carbon care models (NHS England 2022; NHS Shared Business Services 2022).

Evidence focusing on the effectiveness of interventions to reduce carbon emissions within secondary healthcare would be a useful complement to work already underway both within the United Kingdom and other high‐income countries. An approach which considers how care is delivered across patient care pathways within individual specialities may help support the delivery of equitable and accessible high‐quality care. Such an approach can also consider other wider health services policies and ensure that all those who are involved in the design and delivery of care are involved. This review was commissioned by the Greener NHS team and could serve as a useful case study for wider net‐zero ambitions elsewhere in the world.

### 2.3 Existing Systematic Review Evidence

Scoping of the evidence base indicates that there are several systematic reviews which examine different types of interventions to reduce carbon emissions, which are summarised below.

Four systematic reviews focus on interventions to reduce carbon emissions within operating theatres (Keil et al. 2023; Papadopoulou et al. 2022; Perry et al. 2023; Siu et al. 2017). Papadopoulou et al. (2022) examine the environmental sustainability of minimally invasive surgery techniques (including robotic and laparoscopic surgery) and include studies from a variety of different specialities which examine different interventions such as cost‐awareness campaigns and reusable instruments or report a life cycle assessment (LCA) for a particular surgical procedure (Papadopoulou et al. 2022). The number of studies evaluating/modelling the effect of an intervention in this review was limited (*n* = 6), with gynaecology and gastroenterology being the main surgical specialities represented (Papadopoulou et al. 2022). In the review conducted by Perry et al. (2023), studies evaluated interventions focusing on recycling and waste management, waste reduction, reuse, reprocessing/LCA, energy and resource reduction and anaesthetic gases (Perry et al. 2023). Searches were confined to the medical literature, and carbon emission data were not routinely reported for all the included primary studies. Keil et al. (2023) included LCAs which compared single‐use and reusable healthcare products with similar functions (Keil et al. 2023). Interventions focused on non‐invasive medical devices, inhalers, invasive medical devices and protective equipment. The review synthesis predominantly focused on GHG emission data, rather than carbon emissions, and did not consider the influence of individual specialities (Keil et al. 2023). Finally, the review conducted by Siu et al. (2017) compared the environmental impact of reusable versus disposable laparoscopic instruments (Siu et al. 2017). Searches for this review were limited to sources from the medical field, and the review authors did not conduct a quality appraisal of the included studies or report carbon‐emission outcomes.

Two systematic reviews explored the environmental impact of telemedicine interventions in place of face‐to‐face patient care (Lange et al. 2022; Ravindrane and Patel 2022). The review by Ravindrane and Patel (2022) encompassed renal medicine, head and neck cancer, vascular surgery and urology specialities (Ravindrane and Patel 2022). While the review reported the impact of this type of intervention on carbon emissions, it did not consider variation in the use of telemedicine within different specialities (Ravindrane and Patel 2022). Lange et al. (2022) applied a transparency checklist for carbon footprint calculations within a systematic review of virtual care interventions (Lange et al. 2022). Overall, the review highlighted a saving of 148 kg carbon dioxide equivalents per patient, but indicated the evidence was weak, with the reported carbon footprint being highly heterogeneous (Lange et al. 2022). This review did not calculate contributions of individual specialities/pathways (Lange et al. 2022). In addition, these existing systematic reviews do not consider the evidence relating to the environmental impact of these interventions alongside the impact on patient and financial outcomes.

### 2.4 Why Is It Important to Carry Out Another Systematic Review and Develop an EGM?

Due to the lack of systematic reviews which consider carbon emissions associated with the patient pathway within individual specialities, further research is needed to enable the identification and transformation of the most carbon‐intensive clinical pathways, while ensuring future models of care can be delivered in a cost‐effective manner without increasing emissions or compromising patient care. A systematic review will enable the synthesis of existing evidence to address these research needs.

An EGM provides an overview of the quantity, quality and nature of primary evidence which already exists in this area (White et al. 2020), and how this evidence may help inform the patient care pathway. The interactive features of the EGM will enable users to identify and access evidence most suited to their needs.

## 3 Objectives

To carry out a systematic review that examines the effectiveness of interventions in reducing the carbon footprint within medical specialities with high levels of inpatient activity in secondary healthcare.

Our research question is as follows:

What is the effectiveness of interventions for reducing the carbon footprint of medical interventions carried out in the following medical specialities within secondary healthcare:

- Cardiology
- Gastroenterology
- Ophthalmology
- Orthopaedics and trauma
- Renal
- Respiratory
- High volume low complexity surgery, specifically:
  ∘ ENT
  ∘ Gynaecology
  ∘ Urology

We focused our research question on medical specialities with high levels of inpatient activity, as these are likely to have the greatest impact on carbon emissions.

## 4 Methods

Our review protocol was prospectively registered on the Open Repository Exeter (Shaw et al. 2023). The methods used to conduct and report the findings are consistent with the best practice approach for the conduct of systematic reviews and reporting of evidence synthesis and EGM (Centre for Reviews and Dissemination 2009; Page et al. 2021; Zumsteg et al. 2012). The methods reported below apply to both the processes of conducting the systematic review and producing the EGM.

### 4.1 Stakeholder Engagement

We have consulted with and worked closely alongside several stakeholder groups throughout the conduct of this review and the production of this review and EGM. Stakeholders included those requesting the review from the NHS England Greener NHS team, people with expertise in LCA methods or studies evaluating interventions to reduce carbon emissions within healthcare settings from the University of Exeter, and members of the PERSPEX patient and public involvement group. The method of engaging with and respective impact on the review process, of each of these stakeholder groups on the review process and outputs is summarised in Table 1.

**Table 1:** Impact of stakeholder involvement on review.

Stage of review	Method of stakeholder involvement	Impact on review
Research question and protocol development	12.01.23/07.03.23: 2 × 1 h face‐to‐face meetings via MT with government policy stakeholders, communication via email.	Clarification regarding policy context for the review, aim, intended use and identifying focused research question. Identification of high‐volume specialities which likely to be associated with highest carbon‐emissions. Highlighted Healthcare LCA database and other websites as resources to include in search strategy and facilitated contact with individuals who maintain this resource. Approved inclusion criteria, with particular input regarding outcomes of interest. Approved search terms and search strategy.
	Email contact with individuals who maintain HealthcareLCA database.	Informing review search strategy.
	1 × 20 min face‐to‐face meeting via MT with members of PERSPEX.	Discussion of research questions and aims of review highlighted importance of including interventions targeting waste management, for example, use of paper and incineration. Emphasised importance of consideration of patient outcomes, such as patient satisfaction and safety alongside carbon emission evidence. Identified need for plain language protocol to share with patient/public collaborators.
	13.9.23: 1 × 45 min face‐to‐face meeting via MT with clinical/methods expert.	Sense check of protocol content and signposting to other potential experts regarding LCA methodology. Clarification of different ways carbon emissions can be measured and/or referred to in non‐LCA studies and definition of these. Provided context of how carbon emissions evaluated within NHS settings and associated challenges. Provided thoughts on synthesis strategy to enable identification of key messages for individual specialties.
Screening	18.09.23: 1 × 1 h meeting via MT with government policy stakeholders, communication via email.	Comment on summary of included studies as to whether these met expectations on type and range of evidence eligible for inclusion in review and if this aligned with their perception of the purpose of the review. Helped us resolve uncertainties regarding study eligibility at full‐text screening.
Data extraction	22.09.23: 1 × 45 min face‐to‐face meeting via MT with methods expert.	Discussed draft data extraction and quality appraisal forms.
	23.11.23: 1 × 45 min face‐to‐face meeting via MT with XY, email communication.	Provided an introductory overview to LCA methodology and answered specific queries regarding key concepts such as differentiating LCA from inventory analysis, representativeness of data, sensitivity/uncertainty analysis. Reviewed content draft data extraction and quality appraisal forms, which pertained to studies utilising LCA methods.
	14.12.23: 1 × 1 h face‐to‐face meeting via MT with government policy stakeholders, communication via email.	Approved content of draft data extraction form. Particular request for details regarding research funding and conflict of interest. Decision to prioritise studies utilising LCA methodology for full data extraction and quality appraisal. Supported identifying key study characteristics to extract from non‐LCA studies.
Quality appraisal	Communication via email with XY.	Resolved specific queries regarding key concepts relating to quality appraisal of LCA studies and identifying studies which, while adopting features and language from LCA study designs, were non‐LCA studies.
Synthesis	14.12.23/23.01.24: 2 × 1 h face‐to‐face meetings via MT with government policy stakeholders, communication via email.	Decision to prioritise evidence based on LCA study design, with shorter narrative overview of findings from non‐LCA studies based on discussion. Broad intervention categories initially identified by MP, refined by Exeter PRP review team and agreed upon through discussion. Need for evidence and gap map identified through stakeholder need to see how evidence included in the review mapped onto patient care pathway.
	06.03.24: 1 × 30 min meeting via MT with members of PERSPEX. Communication via email.	Provided feedback on preliminary findings of review and structure and accessibility of the evidence and gap map.>
Write up	Communication via email with XY.	Reviewed and provided feedback on draft internal report before it being sent to government stakeholders.
	Communication via email with government stakeholders.	Reviewed and provided feedback on draft report before it being finalised.
Dissemination	Communication via email with government stakeholders. 03.04.24: 1 × 30 min meeting via MT with members of PERSPEX. Communication via email.	Government stakeholders and PERSPEX members involved in identifying key audiences for potential dissemination products and pathways for sharing these. Reviewed dissemination materials, including a plain language summary and briefing paper. Also provided feedback on structure of simplified patient care pathway on which evidence and gap map based and accessibility of draft evidence and gap map.

Abbreviations: LCA; life cycle assessment; MT, Microsoft Teams; NHS, National Health Service.

### 4.2 Dimensions

#### 4.2.1 Types of Study Design

This systematic review and EGM includes studies of any comparative study design, including (but not limited to):

– RCTs,
– Controlled trials,
– Prospective and retrospective cohort studies,
– Before‐and‐after studies,
– Interrupted time series,
– Modelling studies,
– LCAs that compare different treatments/processes.

We defined studies using a full LCA approach, which comprises both:

1. An inventory analysis, evaluating the energy consumption, emissions and resources associated with an intervention throughout the life‐cycle of the product, process or activity, and
2. An impact assessment, converting inventory data from the LCA into a set of potential impacts on the environment (e.g., carbon emissions, eutrophication, ecosystem quality, non‐renewable resources, etc.).

This definition is consistent with ISO guidelines (14040, 2006). However, we also included studies based only on inventory analyses.

We excluded the following study designs:

– LCAs that provide only an estimate of carbon emissions associated with a particular treatment/process but present no comparison between different treatment/process options,
– Patient case studies,
– Systematic, scoping, or narrative reviews,
– Qualitative studies,
– Conference abstracts.

This focus on comparative quantitative primary studies was consistent with the review's aim to establish the effectiveness of interventions to reduce carbon emissions within secondary healthcare settings.

#### 4.2.2 Types of Intervention/Problem

We included any intervention intended to reduce the environmental impact of a process, treatment, or pathway. Examples of eligible interventions include (but are not limited to): waste reduction, remote clinics, surgical techniques, technology/instruments, treatment pathways, manufacturing, imaging, tests, and medication.

Interventions compared with any comparator were included.

We excluded any intervention not associated with a speciality listed below.

#### 4.2.3 Types of Population

We included procedures, processes, or pathways within the following specialities:

– Cardiology
– Gastroenterology
– Obstetrics
– Oncology
– Ophthalmology
– Orthopaedics and trauma
– Radiology
– Renal
– Respiratory
– High volume, low complexity surgery, including:
  ∘ ENT
  ∘ Gynaecology
  ∘ Urology

This list of specialities was agreed in consultation with the Greener NHS team at NHS England and is based on inpatient hospital data showing high volumes of activity with subsequent implications for carbon footprints. We excluded any procedures, processes or pathways within specialities not listed above.

#### 4.2.4 Types of Outcome Measures

Included: Carbon‐emission data had to be measured (using any metric/calculation), with estimated carbon‐emissions based upon LCA also eligible.

Excluded: Studies only measuring outcomes related to patient, clinical, safety and/or satisfaction, with no measurement of carbon‐emission outcomes.

#### 4.2.5 Other Eligibility Criteria

##### 4.2.5.1 Types of Location

Unrestricted.

##### 4.2.5.2 Types of Settings

Interventions needed to be delivered within secondary healthcare and included those focusing on travel to/from/between secondary sites and remote delivery of care.

Excluded: Any treatment, pathway or process associated with the above‐listed specialties in primary or community healthcare settings, for example, General practice, community nursing care.

##### 4.2.5.3 Language

Studies published in English. This was a pragmatic decision based on resource limitations and the need to ensure the review was delivered within the timeframe available.

##### 4.2.5.4 Date

Studies published from 2008 onwards. This corresponds with the 2008 Climate Change Act, before which evidence shows there were very few studies on carbon emissions in health care systems compared to exponential growth since this date (HealthcareLCA 2021).

### 4.3 Search Methods and Sources

Our review protocol was prospectively registered on the Open Repository Exeter (Shaw et al. 2023). The methods used to conduct and report the findings are consistent with the best practice approach for the conduct of systematic reviews and reporting of evidence synthesis (Centre for Reviews and Dissemination 2009; Page et al. 2021; Zumsteg et al. 2012). Below, we summarise how we identified relevant primary studies, quality appraised these and synthesised their findings.

The search strategy was developed by an information specialist (S.B.) in consultation with the review team and stakeholders (for further information, see ‘Stakeholder involvement’ section below). Our overall approach combined searches of bibliographic databases with backward and forward citation searches of studies that met the inclusion criteria, web searches of topically relevant organisations, searches of Google Search, and checking the included studies of topically similar systematic reviews. In addition, we inspected the Healthcare LCA database for relevant studies.

On 11th and 12th July 2023, we searched the following bibliographic databases with coverage of both health care and environmental science journals, including the health care databases MEDLINE and Embase (both via Ovid), the environmental science database Environment Complete (via EBSCO) and the multidisciplinary Science Citation Index database (via Web of Science, Clarivate Analytics). Searches of MEDLINE and Embase combined search terms for carbon emissions with search terms for relevant specialities (see MEDLINE search in Appendix 1, Search strategies). Medical speciality terms included generic terms for each speciality (e.g., gastroenterology, cardiology, etc.), diseases within each speciality which are treated in secondary care settings, and procedures within each speciality which are carried out in secondary care settings. A different approach was used to search Environment Complete and the Science Citation Index, which combined search terms for carbon emissions with generic terminology for hospital settings and secondary care (see Environment Complete search in Appendix 1, Search strategies). This approach was informed by our scoping searches, which suggested that potentially relevant studies published in environmental science journals typically use more generic terminology to describe medical settings than studies in medical and health care journals. A date limit of 2008 was applied across all databases. English language limits were applied where available.

The results of the bibliographic database searches were exported to EndNote 20 (Clarivate Analytics, Philadelphia, PA, USA) and de‐duplicated using the automated de‐duplication feature and manual checking.

We supplemented our bibliographic database searches by inspecting the HealthcareLCA database (https://healthcarelca.com/). This regularly updated resource indexes studies of LCAs of medical technologies and procedures, including carbon emissions, and can be filtered to identify studies relevant to specific medical specialities, including several specialities which are included in this review.

Forward and backward citation searches were conducted on all studies that met our inclusion criteria. Forward citation searching was carried out via the Science Citation Index (Web of Science, Clarivate Analytics) and Google Scholar (https://scholar.google.co.uk/), depending on which citation index indexed the relevant studies, which were identified. We also checked the included studies for any topically relevant systematic reviews that we identified during our scoping and screening processes.

We searched Google Search to identify studies not indexed in bibliographic databases or citation indexes, such as hospital‐led evaluations published in grey literature format. Finally, we searched a selection of websites (see Appendix 1, Search strategies) for relevant studies.

The search strategies used for Google Search and websites are available in Appendix 1, Search strategies.

### 4.4 Data Collection and Analysis

#### 4.4.1 Screening and Study Selection

As an initial calibration exercise to determine the clarity of our inclusion and exclusion criteria, four reviewers applied the criteria to a sample (*n* = 100) of search results (L.S., S.B., N.O., H.M.L.). Decisions were discussed in a group meeting to ensure consistent application of the criteria. Inclusion and exclusion criteria were revised to enable more consistent reviewer interpretation and judgement and applied to a second sample of 100 studies. Once finalised, the revised inclusion and exclusion criteria were applied to the title and abstract of each identified citation independently by two reviewers (L.S., S.B., N.O., H.M.L.), with disagreements resolved through discussion or referral to a third reviewer as required. The full text of each record was assessed for inclusion in the same way. Study selection was supported by EndNote v.20 software, and a PRISMA‐style flowchart was produced, detailing study selection and the reason for exclusion of each record retrieved at full text.

#### 4.4.2 Data Extraction and Management

Due to the high number of eligible studies identified by our search and screening strategy, we made the pragmatic decision to prioritise the studies using the most robust methods to evaluate the impact of interventions to reduce carbon emissions, for full data extraction and quality appraisal. This two‐tier approach meant that the complete data extraction form, based upon the items pre‐specified in our protocol, was applied to included studies which used LCA methodology, while an abbreviated version was applied to other study designs (see Appendix 2, Data extraction items for included studies). This approach enabled the review team to prioritise resources to ensure that review findings pertaining to carbon emissions were based upon the strongest evidence and that the review remained deliverable within the timeframe available.

The review team developed and piloted a standardised data extraction form (L.S., N.O., S.B., H.M.L.) on a sample of LCA studies (*n* = 3) using Microsoft Excel. The revised form was used to collect information pertaining to population characteristics, interventions evaluated, study methods and outcomes. The full data extraction form was applied to LCA studies by one reviewer (L.S., H.M.L.) and checked by a second (H.M. L., S.B., N.O., J.T.C., R.G., L.S.), with the same process applied to non‐LCA studies using a shorter data extraction form. The data extracted from LCA and non‐LCA studies can be viewed Appendix 2, Data extraction items for included studies.

#### 4.4.3 Assessing Risk of Bias of Primary Studies

We critically appraised the LCA studies using a predetermined scoring system, which was informed by Weidema's (1997) guidelines for critical review of LCAs and additional work by Drew et al. (2021) (B.P.W. 1997; Drew et al. 2021). The scoring system comprised 16 appraisal criteria divided across the four stages of the LCA. LCAs should, in accordance with ISO standards, include goal and scope definition, inventory analysis, impact assessment and interpretation of results. Appendix 3 Critical appraisal criteria applied to LCA studies illustrates the critical appraisal items that were applied to each LCA study. We added the points for each criterion and calculated a total score out of 35 points for each study. Studies were awarded a rating of ‘Low’ risk of bias if they scored 26 points or over, a ‘Medium’ rating if they scored 17.5 or over and a ‘High’ risk of bias rating if they scored below 17.5. Due to the absence of existing guidelines, these thresholds reflected natural divisions in total study scores across the critically appraised studies as determined by the review team. Critical appraisal was completed by one of three reviewers (L.S., H.M.L. and N.O.), checked by a second and consultation with a third to resolve any disagreements.

In a deviation from our protocol, no formal guidelines were used to critically appraise non‐LCA studies. Instead, the findings of individual non‐LCA studies were considered alongside the study design and methods for calculating carbon emissions; this was used to inform statements regarding the confidence which could be placed in their synthesised findings. This allowed us to prioritise the most methodologically robust evidence with respect to carbon emissions for synthesis and deliver the review within the timeframe available. This decision was also informed by the lack of validated quality appraisal tools to assess methods of carbon emission calculation in non‐LCA studies, with highly heterogeneous study designs; issues identified within other systematic reviews on related topics (Lange et al. 2022).

#### 4.4.4 Narrative Synthesis

Data summarising the population, intervention, methodological and quality characteristics of the included studies were summarised in tables and described narratively. To support the narrative synthesis, we first categorised included studies into five groups according to the broad type of intervention being evaluated. These are as follows:

1. Accessing care: Interventions changing patient access to, or pathway through, secondary healthcare. Interventions within this category included: Telehealth or virtual care‐based interventions and de‐centralised care.
2. Product level: Interventions focused on the products used for patient care, for example, reuseable surgery equipment or the type of equipment used.
3. Care delivery: Interventions targeting how treatment is delivered, for example, alterations to care regimens, care pathways or surgical procedures.
4. Setting: Interventions which focus on systems and/or processes supporting the delivery of patient care, for example, waste management or energy conservation initiatives.
5. Multiple: Multi‐component Interventions, which encompassed two or more of the above.

Within each of these five categories, studies were separated into those based on LCA methodology versus those which were not. Studies evaluating similar interventions were then grouped together within these methodological categories, and narrative synthesis was used to identify and explain, where possible, patterns in intervention effectiveness in reducing carbon emissions, with reference to study quality (for LCAs) and/or methods used to calculate carbon emissions. Summary statements were produced for each of the five broad intervention groups with regard to what the evidence base could tell us regarding the impact of interventions on carbon emissions, patient outcomes (e.g., patient safety, satisfaction) and service costs, with consideration of study quality (Healthcare). Multiple interventions evaluated within a single study were considered separately.

### 4.5 Methods for Mapping

#### 4.5.1 Framework Development and Scope

The aim of this EGM was to indicate how the quantitative primary evidence evaluating the effectiveness of interventions to reduce carbon emissions within secondary healthcare identified by this systematic review can inform the hospital patient care pathway.

We used EPPI‐Reviewer and EPPI mapper software to present studies as an EGM to highlight where evidence could inform key points of a generic patient care pathway for each speciality (Appendix 4, Patient care pathway) (Centre DSFaE 2022; Thomas et al. 2022). This patient pathway includes (1) Initial assessment (including diagnostic tests) within secondary care, (2) Initial treatment, (3) Follow‐up care, (4) Ongoing secondary care, (5) Discharge from secondary care and (6) Setting. Definitions for each of these parts of the patient care pathway are as follows:

- Initial assessment: entry into the secondary care pathway, which includes the initial review (consultation) and diagnostic tests needed to get to the next part of the pathway, that is, treatment.
- Initial treatment: the primary treatment received following assessment and diagnostics, based on the diagnosis and management plan. Typically delivered once, for example, a joint replacement operation.
- Routine follow‐up appointments: Routine follow‐up following initial treatment.
- Ongoing secondary care: further treatment or treatment that is delivered as a course or regimen for patients who require longer‐term treatment, for example, haemodialysis.
- Discharge from secondary care: discharge of patients from secondary care.
- Systemic interventions: Interventions which influence the setting or environment within which patient care is delivered. This part of the patient care pathway is for interventions targeting more systemic aspects of the care delivery system, including those which (a) could influence more than one stage of the patient care pathway or (b) sit outside of the pathway (e.g., interventions intended to reduce equipment packaging), but are still associated with the care patients receive.

To provide an accessible structure, the systematic review evidence was mapped according to speciality and the patient care pathway, from initial access of secondary care health services, through to discharge from secondary care. Each included study was assigned to a position on the pathway by one reviewer (L.S.) and checked by a second (N.O.). Disagreements were resolved through discussion. Due to the nature of the interventions evaluated by the included studies, a study may sit in multiple places in the EGM.

#### 4.5.2 Filters for Presentation

The content of the map can be changed using the ‘Filters’ option at the top right‐hand side of the map, according to different features of the systematic reviews. Different filter options are based on the key features of the studies:

1. **Methods used** (e.g., LCA or non‐LCA studies),
2. **Specific type of intervention** (e.g., telehealth, reusable equipment, etc.),
3. **Geographic location** (e.g., UK vs. non‐UK).

#### 4.5.3 Analysis and Presentation

The abstracts of the primary studies included in the review can be viewed by clicking on the individual segments within the EGM, alongside details of the background, methods, results, main findings of the study and links to its full text. Within each segment of the grid, systematic review evidence is presented in bubbles according to broad intervention categories as described above, with the colour and size of the bubble indicating the type of intervention and amount of evidence available within that section of the grid. We produced summaries of the number and type of studies at each stage of the care pathway for each speciality.

## 5 Results

### 5.1 Description of Studies

#### 5.1.1 Results of the Search

**Figure 1:**
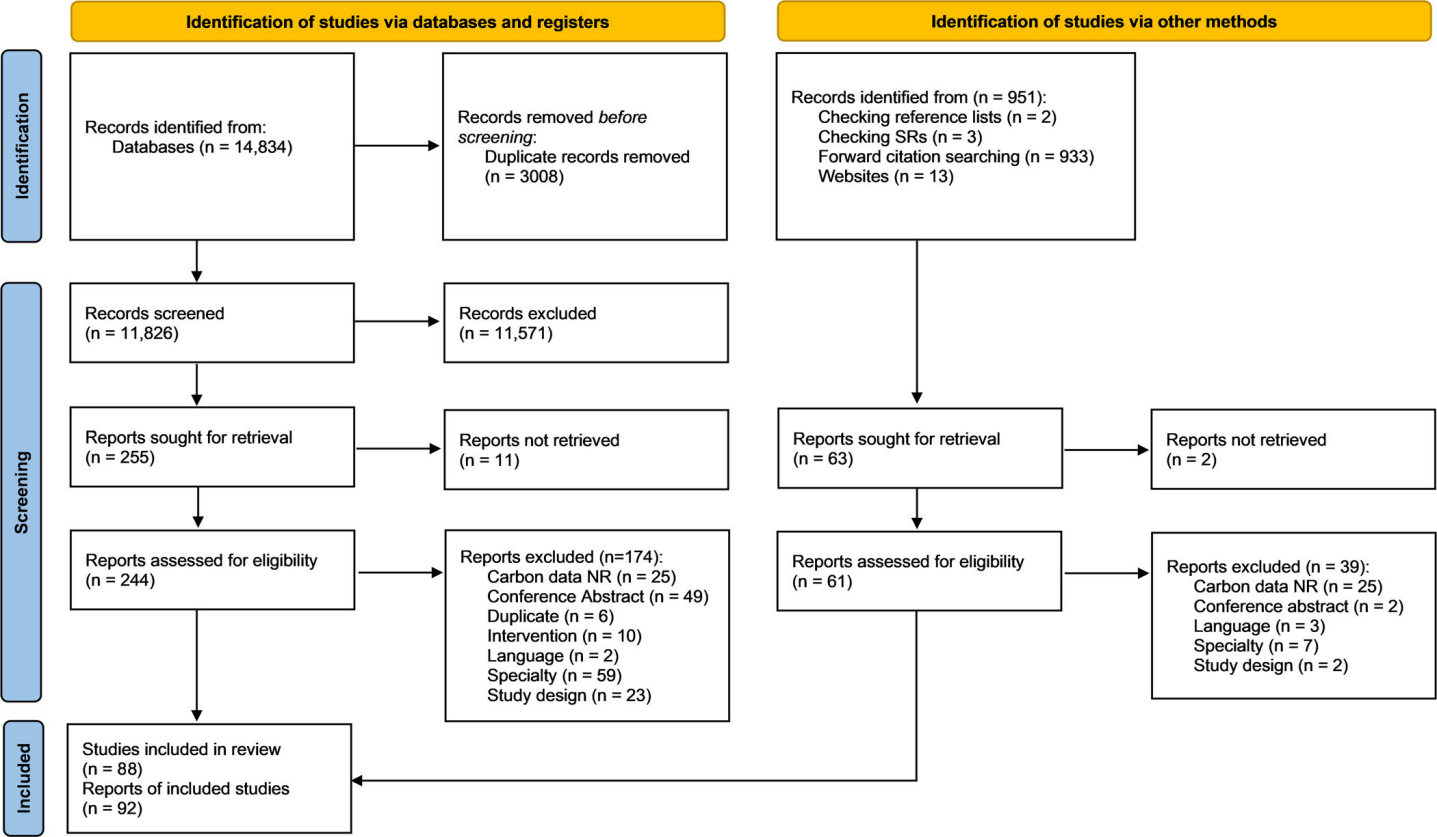
PRISMA diagram showing study selection process.

The bibliographic database searches identified 14,834 records. Following de‐duplication, there were 11,826 unique records. At title and abstract screening, 11,571 records were excluded, leaving 255 to screen at full text. A further 951 records were identified via alternative search methods, including forwards citation chasing (*n* = 933), backwards citation chasing (*n* = 2), website searches (*n* = 13) and checking reference lists of relevant reviews (*n* = 3), of which 63 were sought for retrieval. Of the 305 full texts which could be retrieved, 213 were excluded for the reasons listed in Figure 1. Eighty‐eight studies (92 articles) met eligibility criteria for inclusion in this review and are described in Supporting Material S1.

#### 5.1.2 Excluded Studies

Studies excluded after screening at full text, with reasons for exclusion, can be found at the end of this report: List of ‘Excluded studies’. The most common reasons for exclusion were study design or type of speciality.

### 5.2 Overview of Included Studies

Of the 88 studies (92 articles) which met eligibility criteria for this review, the majority were published in peer‐reviewed academic journals, aside from 13 published as non‐peer‐reviewed project web reports as part of carbon‐reduction initiatives at individual NHS Trusts (Betts 2022; Bird 2022; Burton 2022; Chan 2023; Hardy 2022; Kodumuri et al. 2023; Kodumuri 2022; Langstaff 2023; Lewis et al. 2009; Materacki 2023; Milne 2010; Milne 2023; Nielsen 2022; Owens 2023). Thirty‐three studies were conducted within the United Kingdom (Betts 2022; Bird 2022; Bond et al. 2009; Burton 2022; Chan 2023; Chuter et al. 2023; Connor, Lillywhite, et al. 2011; Connor, Mortimer, et al. 2011; Connor et al. 2019; Coombs et al. 2016; Cooper 2022; Cooper et al. 2023; Curtis et al. 2021; de Preux and Rizmie 2018; Dorrian et al. 2009; Hardy 2022; King et al. 2022; Kodumuri et al. 2023; Kodumuri 2022; Langstaff 2023; Lewis et al. 2009; Materacki 2023; Miah et al. 2019; Milne 2010; Milne 2023; Moussa et al. 2022; Moussa et al. 2021; Natale et al. 2022; Nielsen 2022; Owens 2023; Phull et al. 2023; Richards et al. 2022; Rizan and Bhutta 2022a; Udayaraj et al. 2019; Yong et al. 2022; Zander et al. 2011). Other countries included the United States (*n* = 19) (Beswick et al. 2016; Field et al. 2023; Frick et al. 2023; Jiang et al. 2021; Kemble et al. 2023; Le et al. 2022; Leapman et al. 2023; Meiklejohn et al. 2023; Mojdehbakhsh et al. 2021; Patel et al. 2023; Sherman et al. 2018; Sillcox, Blaustein, et al. 2023; Sillcox, Gitonga, et al. 2023; Thiel et al. 2015; Thiel et al. 2023; Thiel et al. 2018; Thota et al. 2020; Vo et al. 2023; Woods et al. 2015; Woolen et al. 2023), France (*n* = 5) (Baboudjian et al. 2022; Bendine et al. 2020; Chambrin et al. 2023; Filfilan et al. 2021; Rouviere et al. 2022), Germany (*n* = 6) (Arndt et al. 2023; Buttner et al. 2021; Klein 2023; Leiden et al. 2020; Muschol et al. 2022; Schulte et al. 2021), Australia (*n* = 4) (Andrew et al. 2020; Davis et al. 2018; McAlister et al. 2022; Wombwell et al. 2023), Canada (*n* = 4) (Cheung et al. 2023; Forner et al. 2021; Lambert et al. 2023; Tselapedi‐Sekeitto 2023), Sweden (*n* = 3) (Boberg et al. 2022; Holmner et al. 2014; Stripple et al. 2008), Ireland (*n* = 3) (Croghan et al. 2021; Hogan et al. 2022; McCarthy et al. 2014), multiple countries (*n* = 2) (Asghari and Al‐e‐Hashem 2020; Vaidya et al. 2022), and one study was conducted within Austria (Winklmair et al. 2023), China (Chen et al. 2017), Denmark (Sørensen and Grüttner 2018), Italy (Fuschi et al. 2023), New Zealand (McLachlan et al. 2021), Portugal (Neves et al. 2022), Spain (Lopez‐Munoz et al. 2023), Switzerland (Heye et al. 2023).

Twenty‐eight studies used LCA‐informed methods to calculate carbon emissions. Six studies conducted inventory analysis (Holmner et al. 2014; Kemble et al. 2023; Sillcox, Gitonga, et al. 2023; Thiel et al. 2018; Winklmair et al. 2023; Wombwell et al. 2023), or used methods informed by a LCA approach (*n* = 3) (Chuter et al. 2023; Connor, Lillywhite, et al. 2011; de Ridder et al. 2022), such as healthcare sustainability mode and effect analysis (de Ridder et al. 2022), or component analysis (Connor, Lillywhite, et al. 2011). Nineteen studies used a full LCA approach (Baboudjian et al. 2022; Boberg et al. 2022; Davis et al. 2018; Fuschi et al. 2023; Hogan et al. 2022; Le et al. 2022; Leapman et al. 2023; Leiden et al. 2020; Lopez‐Munoz et al. 2023; McAlister et al. 2022; Meiklejohn et al. 2023; Rizan and Bhutta 2022a; Rouviere et al. 2022; Schulte et al. 2021; Sherman et al. 2018; Sørensen and Grüttner 2018; Stripple et al. 2008; Thiel et al. 2015; Thiel et al. 2023). Of the 35 studies conducted within the United Kingdom, one used full LCA methodology (Rizan and Bhutta 2022a).

Of the studies which did not use LCA methods to calculate carbon emissions, 19 were based on an experimental study design, including three RCTs (Coombs et al. 2016; Field et al. 2023; Muschol et al. 2022), five controlled trials (Chen et al. 2017; Cooper et al. 2023; Frick et al. 2023; Hogan et al. 2022; Tselapedi‐Sekeitto 2023), one feasibility study (Beswick et al. 2016), and 11 before and after studies (Arndt et al. 2023; Betts 2022; Cheung et al. 2023; Kodumuri et al. 2023; Kodumuri 2022; Langstaff 2023; Materacki 2023; McCarthy et al. 2014; McLachlan et al. 2021; Neves et al. 2022; Nielsen 2022; Udayaraj et al. 2019). Nine were modelling studies (Asghari and Al‐e‐Hashem 2020; Bird 2022; Burton 2022; de Preux and Rizmie 2018; Hardy 2022; Milne 2010; Milne 2023; Owens 2023; Zander et al. 2011). The remaining studies used observational methods, with the most common study designs being cross‐sectional (*n* = 9) (Buttner et al. 2021; Connor, Mortimer, et al. 2011; Croghan et al. 2021; Filfilan et al. 2021; Forner et al. 2021; King et al. 2022; Lewis et al. 2009; Mojdehbakhsh et al. 2021; Patel et al. 2023), retrospective or prospective cohort (*n* = 16) (Bendine et al. 2020; Beswick et al. 2016; Chambrin et al. 2023; Connor et al. 2019; Curtis et al. 2021; Dorrian et al. 2009; Heye et al. 2023; Jiang et al. 2021; Miah et al. 2019; Natale et al. 2022; Richards et al. 2022; Thota et al. 2020; Vaidya et al. 2022; Vo et al. 2023; Woolen et al. 2023; Yong et al. 2022), and database review (*n* = 6) (Andrew et al. 2020; Bond et al. 2009; Lambert et al. 2023; Phull et al. 2023; Sillcox, Blaustein, et al. 2023; Woods et al. 2015). Further detail regarding the methods used in the studies based on LCA is provided as Supporting Material S2.

Studies were classified according to the five broad intervention categories: ‘Accessing care’ (*n* = 29) (Andrew et al. 2020; Arndt et al. 2023; Asghari and Al‐e‐Hashem 2020; Beswick et al. 2016; Bond et al. 2009; Connor, Mortimer, et al. 2011; Connor et al. 2019; Croghan et al. 2021; Curtis et al. 2021; Dorrian et al. 2009; Filfilan et al. 2021; Forner et al. 2021; Holmner et al. 2014; Jiang et al. 2021; King et al. 2022; Lambert et al. 2023; Lewis et al. 2009; McLachlan et al. 2021; Miah et al. 2019; Mojdehbakhsh et al. 2021; Muschol et al. 2022; Natale et al. 2022; Patel et al. 2023; Richards et al. 2022; Sillcox, Blaustein, et al. 2023; Sillcox, Gitonga, et al. 2023; Thiel et al. 2023; Thota et al. 2020; Tselapedi‐Sekeitto 2023; Udayaraj et al. 2019), ‘Setting’ (*n* = 20) (Betts 2022; Bird 2022; Buttner et al. 2021; Chambrin et al. 2023; de Ridder et al. 2022; Heye et al. 2023; Klein 2023; Kodumuri et al. 2023; Kodumuri 2022; Lopez‐Munoz et al. 2023; McAlister et al. 2022; McCarthy et al. 2014; Milne 2010; Milne 2023; Neves et al. 2022; Owens 2023; Vo et al. 2023; Winklmair et al. 2023; Woolen et al. 2023; Yong et al. 2022), ‘Product level’ (*n* = 16) (Baboudjian et al. 2022; Boberg et al. 2022; Chan 2023; Davis et al. 2018; Field et al. 2023; Hogan et al. 2022; Kemble et al. 2023; Le et al. 2022; Leiden et al. 2020; Moussa et al. 2022; Moussa et al. 2021; Rizan and Bhutta 2022a; Schulte et al. 2021; Sherman et al. 2018; Sørensen and Grüttner 2018; Stripple et al. 2008; Wombwell et al. 2023), ‘Care delivery’ (*n* = 16) (Burton 2022; Chen et al. 2017; Connor, Lillywhite, et al. 2011; Coombs et al. 2016; Cooper 2022; Cooper et al. 2023; de Preux and Rizmie 2018; Frick et al. 2023; Fuschi et al. 2023; Langstaff 2023; Leapman et al. 2023; Meiklejohn et al. 2023; Nielsen 2022; Phull et al. 2023; Thiel et al. 2015; Vaidya et al. 2022; Woods et al. 2015; Zander et al. 2011), and ‘Multiple components’ (*n* = 7) (Bendine et al. 2020; Cheung et al. 2023; Chuter et al. 2023; Hardy 2022; Materacki 2023; Rouviere et al. 2022; Thiel et al. 2018). Urology (*n* = 14) (Baboudjian et al. 2022; Connor et al. 2019; Croghan et al. 2021; Davis et al. 2018; Filfilan et al. 2021; Fuschi et al. 2023; Hogan et al. 2022; Kemble et al. 2023; Leapman et al. 2023; Miah et al. 2019; Natale et al. 2022; Phull et al. 2023; Stripple et al. 2008; Wombwell et al. 2023), Gastroenterology (*n* = 12) (Betts 2022; Boberg et al. 2022; King et al. 2022; Le et al. 2022; Lopez‐Munoz et al. 2023; Materacki 2023; Neves et al. 2022; Owens 2023; Rizan and Bhutta 2022a; Sherman et al. 2018; Sillcox, Blaustein, et al. 2023; Sillcox, Gitonga, et al. 2023; Yong et al. 2022), and Oncology/Radiation oncology (*n* = 13) were the most common specialities represented (Beswick et al. 2016; Cheung et al. 2023; Coombs et al. 2016; Forner et al. 2021; Frick et al. 2023; Jiang et al. 2021; Lambert et al. 2023; Langstaff 2023; Lewis et al. 2009; Patel et al. 2023; Thota et al. 2020; Vaidya et al. 2022; Woods et al. 2015). The most common intervention evaluated was telemedicine (*n* = 26) (Andrew et al. 2020; Arndt et al. 2023; Beswick et al. 2016; Connor, Mortimer, et al. 2011; Connor et al. 2019; Croghan et al. 2021; Curtis et al. 2021; Dorrian et al. 2009; Filfilan et al. 2021; Holmner et al. 2014; Jiang et al. 2021; King et al. 2022; Lambert et al. 2023; Lewis et al. 2009; McLachlan et al. 2021; Miah et al. 2019; Mojdehbakhsh et al. 2021; Muschol et al. 2022; Natale et al. 2022; Patel et al. 2023; Richards et al. 2022; Sillcox, Blaustein, et al. 2023; Sillcox, Gitonga, et al. 2023; Thiel et al. 2023; Thota et al. 2020; Tselapedi‐Sekeitto 2023; Udayaraj et al. 2019), which included three studies using LCA methods (Holmner et al. 2014; Sillcox, Gitonga, et al. 2023; Thiel et al. 2023). LCA methods were most commonly used to evaluate interventions within the ‘Product level’ category, specifically interventions comparing carbon emissions associated with reuseable versus single use equipment (*n* = 12) (Baboudjian et al. 2022; Boberg et al. 2022; Davis et al. 2018; Hogan et al. 2022; Kemble et al. 2023; Le et al. 2022; Leiden et al. 2020; Rizan and Bhutta 2022a; Schulte et al. 2021; Sherman et al. 2018; Sørensen and Grüttner 2018; Wombwell et al. 2023), with the highest number in urology product‐level interventions (*n* = 6) (Baboudjian et al. 2022; Davis et al. 2018; Hogan et al. 2022; Kemble et al. 2023; Stripple et al. 2008; Wombwell et al. 2023). Further detail regarding interventions evaluated by the studies using LCA methods is provided in Appendix 5, Description of interventions evaluated by LCA studies.

### 5.3 Quality Appraisal of Included Studies

The quality appraisal scores of LCA studies ranged from 11.5 to 34 (see Table 2). The majority of the studies had a clear aim or rationale and were clear on the paper's intended application and/or audience. All but three studies stated the lifecycle assessment method clearly (Chuter et al. 2023; Hogan et al. 2022; Rouviere et al. 2022). However, only eight studies explicitly reported that they had conducted the study in accordance with ISO standards (ISO 14040 series) (Boberg et al. 2022; Fuschi et al. 2023; Leapman et al. 2023; McAlister et al. 2022; Rizan and Bhutta 2022a; Schulte et al. 2021; Sherman et al. 2018; Thiel et al. 2023).

**Table 2:** Critical appraisal of LCA studies.

References	Study goal clearly stated, study's rationale (1), intended application, and/or intended audience (1)	Life cycle assessment method clearly stated (1)	Functional unit clearly defined and measurable (1), justified (1), and consistent with study's intended application (1)	System adequately described with clearly stated boundaries (1), lifecycle stages (1), and justification of omitted stages (1)	The system covers production (1), use/reuse (1) and disposal (1) of materials and energy	Data collection process clearly explained, including source(s) foreground material weights/energy values (1); source(s) reference data (1); what data included (1)	Representativeness of data discussed (1), differences in electricity generating mix accounted for (1), and potential significance of exclusions/assumptions addressed (1)	Allocation procedures, where necessary, are described and appropriately justified (1)	Impact categories (1), characterisation method (1), and software used (1) are documented transparently	Results are clearly reported in the context of the functional unit (1) (0.5 if graphically, 0 if only normalized results reported)	A contribution analysis is performed and clearly reported (1), and hotspots are identified (1)	Conclusions consistent with goal and scope (1) and supported by impact assessment (1)	Results are contextualised through the use of sensitivity analysis (1) and uncertainty analysis (1)	Limitations adequately discussed (1), and potential impact of omissions/assumptions on study's outcomes described (1)	Assessment has been critically appraised (1)	Source(s) of funding (1) and potential COI disclosed (1), and unlikely to be source of bias (1)	Overall rating
Baboudjian et al. (2022)	2	1	3	3	1.5	1	2	0	2	0	2	2	1	2	1	2	25.5
Boberg et al. (2022)	2	1	3	3	2	3	2	1	2	1	2	2	2	2	1	3	32
Chuter et al. (2023)	2	0	2	2	0.5	2	1	NA	NA	1	2	2	NA	1	1	3	19.5
Connor, Lillywhite, et al. (2011)^a^	2	1	1	3	3	3	3	1	0	0	2	2	0	2	1	0	24
Davis et al. (2018)	2	1	0	1	3	2	1	NA	0	0.5	2	0	0	0	1	1	14.5
de Ridder et al. (2022)^b^	2	1	0	2	1	2	0.5	1	NA	0	2	2	0	1	1	3	18.5
Fuschi et al. (2023)	1	1	0	3	3	3	0	0	3	0.5	0	1	0	0	1	1	17.5
Hogan et al. (2022)	1	0	0	2	1.5	2	1	1	NA	0	2	2	0	2	1	1	16.5
Holmner et al. (2014)	2	1	3	3	3	3	2	NA	0	1	0	1	2	2	1	3	27
Kemble et al. (2023)	2	1	0	3	2	3	0.5	0	0	1	2	1	0	2	1	3	19.5
Le et al. (2022)	1	1	2	2	3	3	1	0	2	1	2	2	1	2	1	0	24
Leapman et al. (2023)	2	1	2	3	3	3	1	0	0	1	2	2	1	2	1	1	25
Leiden et al. (2020)	2	1	2	2	1.5	2	2	0	2	0.5	2	2	1	0	1	1	22
López‐Muñoz et al. (2023)	2	1	0	3	1.5	3	2	1	1	0	2	2	0	2	1	3	24.5
McAlister et al. (2022)	2	1	2	3	3	3	3	0	0	2	2	2	0	2	1	3	29
Meiklejohn et al. (2023)	2	1	2	2	3	3	1	0	2	1	2	2	1	1	1	0	24
Rizan and Bhutta (2022a)	2	1	2	3	3	3	2	1	3	1	2	2	1	2	1	1	30
Rouviere et al. (2022)	2	0.5	1	1	0	0	0	0	1	0	0	1	0	1	1	3	11.5
Schulte et al. (2021)	2	1	3	3	3	3	3	1	3	1	2	2	1	2	1	3	34
Sherman et al. (2018)	2	1	2	2	2	3	2	1	2	1	2	2	1	0	1	3	27
Sillcox, Gitonga, et al. (2023)	1	1	2	3	3	3	2	NA	0	1	1	2	1	2	1	1	24
Sørensen and Grüttner (2018)	2	1	2	2	2	3	1	0	1	0.5	1	2	1	2	1	2	23.5
Stripple et al. (2008)	2	1	3	3	2	1	1	0	2	0.5	0	2	1	0	1	1	20.5
Thiel et al. (2015)	3	1	2	2	2.5	3	2	1	3	1	1	2	1	2	1	3	31.5
Thiel et al. (2018)	2	1	0	0	1.5	2	1	1	0	0	2	2	0	0	1	0	13.5
Thiel et al. (2023)	2	1	2	3	3	3	0.5	NA	2	1	2	2	2	1	1	3	28.5
Winklmair et al. (2023)	2	1	0	2	0.5	2	1	NA	0	0	0	2	0	1	1	2	14.5
Wombwell et al. (2023)	2	1	0	2	1.5	2	0.5	1	0	0	1	2	0	2	1	3	19

*Note:* Top score – 35. Low risk of bias – > 26 (Green), Medium risk of bias – > 17.5 (Blue), High risk of bias – 17.5 (Amber). Inventory analysis (pink). The numbers 0, 1, 2, or 3 within the quality appraisal table indicate the extent to which each study meets the quality criteria stated at the top of each column. The criteria associated with one (1) point is indicated within the column heading.

Abbreviations: COI, conflict of interest; NA, not applicable.

^a^ Component analysis.

^b^ Healthcare Sustainability Mode and Effect Analysis.

Not all studies were classified as full LCAs with some stating that the study was a ‘simplified’ LCA (Hogan et al. 2022; Holmner et al. 2014), others were inventory analyses as they did not consider environmental impacts beyond carbon emissions (Chuter et al. 2023; Davis et al. 2018; Fuschi et al. 2023; Hogan et al. 2022; Kemble et al. 2023; Leapman et al. 2023; McAlister et al. 2022; Sillcox, Gitonga, et al. 2023; Winklmair et al. 2023; Wombwell et al. 2023). Only five studies reported full details on the functional unit (Baboudjian et al. 2022; Boberg et al. 2022; Holmner et al. 2014; Schulte et al. 2021; Stripple et al. 2008), while nine did not report any details at all (Davis et al. 2018; de Ridder et al. 2022; Fuschi et al. 2023; Hogan et al. 2022; Kemble et al. 2023; Lopez‐Munoz et al. 2023; Thiel et al. 2018; Winklmair et al. 2023; Wombwell et al. 2023). Thirteen studies reported details on the systems studied and defined the system boundaries, often described as ‘cradle to grave’ (Baboudjian et al. 2022; Connor, Mortimer, et al. 2011; Fuschi et al. 2023; Holmner et al. 2014; Kemble et al. 2023; Leapman et al. 2023; Lopez‐Munoz et al. 2023; McAlister et al. 2022; Rizan and Bhutta 2022a; Schulte et al. 2021; Sillcox, Gitonga, et al. 2023; Stripple et al. 2008; Thiel et al. 2023). Just over a third of the studies fully reported details on the system covered – production, use/reuse and disposal of materials and energy (Connor, Lillywhite, et al. 2011; Fuschi et al. 2023; Holmner et al. 2014; Leapman et al. 2023; McAlister et al. 2022; Meiklejohn et al. 2023; Schulte et al. 2021; Sillcox, Gitonga, et al. 2023; Stripple et al. 2008; Thiel et al. 2023). More than half the studies (*n* = 17) fully explained the data collection process, the data included, and the source(s) of reference data which was most commonly the Ecoinvent database (Boberg et al. 2022; Connor, Lillywhite, et al. 2011; Fuschi et al. 2023; Holmner et al. 2014; Kemble et al. 2023; Le et al. 2022; Leapman et al. 2023; Lopez‐Munoz et al. 2023; McAlister et al. 2022; Meiklejohn et al. 2023; Rizan and Bhutta 2022a; Schulte et al. 2021; Sherman et al. 2018; Sillcox, Gitonga, et al. 2023; Sørensen and Grüttner 2018; Thiel et al. 2015; Thiel et al. 2023). Authors could report obtaining detailed data from manufacturers (e.g., Hogan et al. 2022), and others noted a lack of data on the material composition of devices (e.g., Baboudjian et al. 2022; Le et al. 2022). Allocation was not always necessary (Chuter et al. 2023; Davis et al. 2018; Holmner et al. 2014; Sillcox, Gitonga, et al. 2023; Thiel et al. 2023; Winklmair et al. 2023), but where environmental exchanges had to be allocated to different products, a number of studies described and justified the allocation (Boberg et al. 2022; Connor, Lillywhite, et al. 2011; de Ridder et al. 2022; Lopez‐Munoz et al. 2023; Rizan and Bhutta 2022a; Schulte et al. 2021; Sherman et al. 2018; Thiel et al. 2015; Thiel et al. 2018; Wombwell et al. 2023). Only five studies scored highly on reporting impact assessment (Baboudjian et al. 2022; Fuschi et al. 2023; Rizan and Bhutta 2022a; Schulte et al. 2021; Thiel et al. 2015). Some reported the tools used for assessing environmental impacts: (i) the US EPA's impact assessment model, TRACI (Tool for the Reduction and Assessment of Chemical and other Environmental Impacts) (Leapman et al. 2023; Meiklejohn et al. 2023; Sherman et al. 2018; Thiel et al. 2015; Thiel et al. 2023; Thiel et al. 2018); (ii) ReCiPe Midpoint Hierarchist model (Le et al. 2022; Leiden et al. 2020; McAlister et al. 2022; Rizan and Bhutta 2022a); and (iii) the Eco‐Indicator 99 tool (Stripple et al. 2008).

Only three studies used both uncertainty analyses and sensitivity analyses to contextualise their results (Boberg et al. 2022; Holmner et al. 2014; Thiel et al. 2023). For the majority of studies (*n* = 23), their conclusions were consistent with their goals and supported by their results. In total, three of the 19 LCA studies (15.8%) report 95% CI data (Baboudjian et al. 2022; Lopez‐Munoz et al. 2023; Thiel et al. 2015). Three studies did not disclose a potential conflict of interest or include a funding declaration (Connor, Lillywhite, et al. 2011; Meiklejohn et al. 2023; Thiel et al. 2018). Five studies made a funding declaration but asserted that receiving funding from manufacturing companies such as Amu A/S and Neo Medical S. A. had not influenced the results of studies (Baboudjian et al. 2022; Leiden et al. 2020; Rizan and Bhutta 2022a; Sørensen and Grüttner 2018; Stripple et al. 2008). One study reported a research agreement with an industry partner (Vanguard AG, Germany) who provided primary data for the ‘medical remanufacturing route’ and the ‘bill of materials’ for the catheter under investigation (Schulte et al. 2021).

### 5.4 Summary of Main Findings: EGM

This EGM provides a brief description of where primary research exists across the patient pathway for 13 specialities in secondary healthcare. Further details regarding intervention findings specific to individual specialities can be found in Appendix 6, Speciality‐specific findings and link to online interactive EGM.

#### 5.4.1 Urology

In urology, there are six intervention studies that are categorised as ‘Accessing care’, five of which relate to ‘initial assessment and/or diagnostic test’ (Connor et al. 2019; Croghan et al. 2021; Filfilan et al. 2021; Leapman et al. 2023; Natale et al. 2022), five to ‘follow‐up care’ (Connor et al. 2019; Croghan et al. 2021; Filfilan et al. 2021; Leapman et al. 2023; Miah et al. 2019), and one to ‘discharge from secondary care’ (Miah et al. 2019). There are seven product level studies with four in ‘initial assessment and/or diagnostic test’ (Baboudjian et al. 2022; Hogan et al. 2022; Kemble et al. 2023; Wombwell et al. 2023), and three in ‘initial treatment’ (Davis et al. 2018; Fuschi et al. 2023; Stripple et al. 2008). There is one intervention study categorised as ‘care delivery’ and is in the ‘initial treatment’ part of the care pathway (Phull et al. 2023).

#### 5.4.2 Gastroenterology

In gastroenterology, there are two ‘accessing care’ intervention studies in ‘initial assessment and/or diagnostic test’ (King et al. 2022; Sillcox, Blaustein, et al. 2023; Sillcox, Gitonga, et al. 2023), and one in ‘follow‐up care’ parts of the care pathway (King et al. 2022). There are also four product level studies, two of which are in ‘initial assessment and/or diagnostic test’ (Le et al. 2022; Sherman et al. 2018), and two in ‘initial treatment’ (Boberg et al. 2022; Rizan and Bhutta 2022a). Within this speciality, there are four ‘setting’ studies (i.e., with a focus on waste management or energy conservation), three of which relate to ‘initial assessment and/or diagnostic test’ (Lopez‐Munoz et al. 2023; Neves et al. 2022; Yong et al. 2022), and two in ‘systemic intervention’ (see Section 4) (Betts 2022; Neves et al. 2022). There are also two multicomponent studies in the ‘systemic intervention’ part of the care pathway (Materacki 2023; Owens 2023).

#### 5.4.3 Oncology

Within the cancer speciality, ‘accessing care’ intervention studies dominate across the care pathway, with seven in ‘initial assessment and/or diagnostic test’ (Beswick et al. 2016; Dorrian et al. 2009; Forner et al. 2021; Jiang et al. 2021; Lewis et al. 2009; Patel et al. 2023; Thota et al. 2020), six in ‘follow‐up care’ (Beswick et al. 2016; Forner et al. 2021; Jiang et al. 2021; Lambert et al. 2023; Lewis et al. 2009; Patel et al. 2023), two in ‘ongoing care’ (Lewis et al. 2009; Thota et al. 2020), and one in ‘discharge from secondary care’ (Lewis et al. 2009). There are also five ‘care delivery’ studies that are split across ‘initial treatment’ (*n* = 1) (Woods et al. 2015), and ‘ongoing secondary care’ (*n* = 4) (Coombs et al. 2016; Frick et al. 2023; Langstaff 2023; Vaidya et al. 2022).

#### 5.4.4 Ophthalmology

Ophthalmology has only four studies, two product‐level studies in the ‘initial treatment’ part of the care pathway (Moussa et al. 2022; Moussa et al. 2021), and two ‘setting’ studies in the ‘systemic intervention category of the care pathway (Vo et al. 2023; Winklmair et al. 2023).

#### 5.4.5 Respiratory

There is only one product level study in the respiratory speciality, relating to the ‘initial assessment and/or diagnostic test’ part of the care pathway (Sørensen and Grüttner 2018).

#### 5.4.6 Renal

Almost all the studies within the renal speciality are found within ‘ongoing secondary care’: four are ‘accessing care’ intervention studies (Andrew et al. 2020; Asghari and Al‐e‐Hashem 2020; Connor, Lillywhite, et al. 2011; Udayaraj et al. 2019), two are ‘care delivery’ studies (Chen et al. 2017; Connor, Mortimer, et al. 2011; de Preux and Rizmie 2018), three are multicomponent studies (Bendine et al. 2020; Hardy 2022; Milne 2023), and four are ‘setting’ studies (Bendine et al. 2020; Bird 2022; Milne 2010; Milne 2023). One ‘setting’ study fits within the ‘follow‐up’ care part of the care pathway (Bird 2022).

#### 5.4.7 Cardiac

There are four studies in the cardiac speciality; two ‘accessing care’ intervention studies (one in ‘follow‐up care’ [Nielsen 2022], and one in ‘ongoing secondary care’ [McLachlan et al. 2021]), one care delivery study in ‘initial treatment’ (Zander et al. 2011), and one product level study in ‘initial assessment and/or diagnostic test’ (Schulte et al. 2021).

#### 5.4.8 ENT

There are four studies in the ENT speciality; three accessing care intervention studies and one care delivery study, split across the ‘initial assessment and/or diagnostic test’ (Dorrian et al. 2009; Tselapedi‐Sekeitto 2023), and ‘initial treatment’ parts of the clinical pathway (Burton 2022; Meiklejohn et al. 2023).

#### 5.4.9 Orthopaedic and/or Trauma

Eight of the studies within the orthopaedics/or trauma speciality are accessing care intervention studies spread across three parts of the care pathway, ‘initial assessment and/or diagnostic test’ (*n* = 1) (Arndt et al. 2023), ‘initial treatment’ (*n* = 2) (Cooper et al. 2023; Curtis et al. 2021), and ‘follow‐up care’ (*n* = 5) (Arndt et al. 2023; Cooper 2022; Cooper et al. 2023; Muschol et al. 2022; Richards et al. 2022). The remaining studies are two multicomponent studies, one in ‘initial treatment’ (Kodumuri 2022), and one in ‘systemic interventions’ (Kodumuri et al. 2023), and two product‐level studies found in ‘initial treatment’ (Chan 2023; Leiden et al. 2020).

#### 5.4.10 Radiology

With radiology, there are three ‘accessing care’ intervention studies (two in ‘initial assessment and/or diagnostic test’ [Bond et al. 2009; Jiang et al. 2021], and one in ‘follow‐up care’ [Jiang et al. 2021]); there are five ‘care delivery’ studies, one in ‘follow‐up care’ (Chuter et al. 2023), and four in ‘ongoing secondary care’ (Chuter et al. 2023; Coombs et al. 2016; Frick et al. 2023; Langstaff 2023). There are four ‘setting’ studies (one in ‘initial assessment and/or diagnostic test’ [Buttner et al. 2021], and four in the ‘systemic interventions’ part of the care pathway [Buttner et al. 2021; Heye et al. 2023; McCarthy et al. 2014; Woolen et al. 2023]). There is one multicomponent study in ‘initial assessment and/or diagnostic test’ and in ‘systemic interventions’ (Klein 2023), and one product‐level study (‘initial assessment and/or diagnostic test’ [McAlister et al. 2022]).

#### 5.4.11 Obstetrics

There are only four studies in the obstetrics pathway, one ‘accessing care’ study in ‘follow‐up care’ (Mojdehbakhsh et al. 2021), one ‘care delivery’ study in ‘initial treatment’ (Thiel et al. 2015), and two multicomponent studies in ‘initial treatment’ (de Ridder et al. 2022; Thiel et al. 2018).

#### 5.4.12 Multiple

The ‘multiple’ speciality has two ‘accessing care’ intervention studies, each sitting within the ‘initial assessment and/or diagnostic test’, ‘initial treatment’ and ‘follow‐up care’ parts of the care pathway (Holmner et al. 2014; Thiel et al. 2023). It also has three multicomponent studies, two of which are in ‘initial treatment’ (Field et al. 2023; Rouviere et al. 2022), and the other in ‘systemic interventions’ (Chambrin et al. 2023).

#### 5.4.13 EGM Overall Summary

Urology (*n* = 14), gastroenterology (*n* = 12), oncology/radiation oncology (*n* = 13) and renal (*n* = 11) were the most common specialities represented, and gynaecology (*n* = 3), obstetrics (*n* = 1) and respiratory (*n* = 1) were the least well represented. Across different specialities, the majority of evidence was found in the first three stages of the patient care pathway (initial assessment/diagnostic tests, initial treatment or follow‐up). The exception to this was the renal speciality, where most of the evidence was within the ‘ongoing care’ segment of the patient care pathway. There was limited evidence within the ‘discharge’ segment of the care pathway across all specialities. Evidence relating to the wider healthcare setting was clustered within the gastroenterology (*n* = 5) and radiology specialities (*n* = 5).

### 5.5 Narrative Synthesis

This section presents the narrative synthesis of all included studies, grouped into five broad intervention categories: Accessing care (*n* = 29), Setting (*n* = 19), Product level (*n* = 16), Care delivery (*n* = 16) and Multiple components (*n* = 7). Within each of these categories, studies are grouped into those using LCA‐informed methods and those that use non‐LCA methods. Within each of these methodological groups, studies evaluating similar interventions were clustered together to explore key outcomes such as carbon emissions, patient outcomes and service costs.

A summary providing an overview of findings from each intervention category is provided at the start of each section. Please see Supporting Materials S3 and S4 for details regarding methods and specific outcomes from individual studies.

#### 5.5.1 Accessing Care

**Summary: Accessing Care**

Twenty‐nine studies (30 articles) evaluated the effectiveness of interventions which changed how patients accessed care. Interventions within this category included telehealth or remote care interventions (*n* = 26) (Andrew et al. 2020; Arndt et al. 2023; Beswick et al. 2016; Connor, Mortimer, et al. 2011; Connor et al. 2019; Croghan et al. 2021; Curtis et al. 2021; Dorrian et al. 2009; Filfilan et al. 2021; Holmner et al. 2014; Jiang et al. 2021; King et al. 2022; Lambert et al. 2023; Lewis et al. 2009; McLachlan et al. 2021; Miah et al. 2019; Mojdehbakhsh et al. 2021; Muschol et al. 2022; Natale et al. 2022; Patel et al. 2023; Richards et al. 2022; Sillcox, Blaustein, et al. 2023; Sillcox, Gitonga, et al. 2023; Thiel et al. 2023; Thota et al. 2020; Tselapedi‐Sekeitto 2023; Udayaraj et al. 2019), and de‐centralised care (*n* = 3) (Asghari and Al‐e‐Hashem 2020; Bond et al. 2009; Forner et al. 2021). Three of the studies within this intervention category were LCAs evaluating telehealth interventions (Holmner et al. 2014; Sillcox, Gitonga, et al. 2023; Thiel et al. 2023).

**LCA studies (*n*** **=** **3)** * **:** While the reductions in carbon emissions associated with the use of telemedicine or virtual appointments is consistent across the three studies appraised as Medium to Low risk of bias, the lack of duplication of these findings within specialities and heterogeneity of intervention content and delivery limits confidence in the reliability of these findings and how widely they can be generalised. There was limited evidence regarding the impact of such interventions on patient outcomes or service costs.

**Non‐LCA studies (*n*** **=** 26)*: All 23 studies which compared telehealth interventions to face‐to‐face care reported reduced carbon emissions within the telehealth intervention group. However, most of these conclusions were based on carbon‐emission calculations which considered only patient‐travel saved and did not account for carbon emissions associated with other parts of the system, for example, energy associated with infrastructure use within patient homes and healthcare facilities where staff are based, or emissions associated with the extraction, manufacture, transport and/or disposal of relevant materials, for example, car/petrol production. This is reflected in the limited range of patient outcomes measured, of which patient travel distance and time saved were the most common. Sixteen studies evaluated cost outcomes. In general, the majority of patient and cost outcomes favoured the telemedicine intervention, although most outcomes were analysed descriptively or narratively. Three studies reported that interventions aiming to de‐centralise care demonstrated reduced carbon emissions when compared to standard care, with carbon emission calculations predominantly based on travel saved. Specialities represented included renal (Asghari and Al‐e‐Hashem 2020), oncology (Forner et al. 2021), and radiology (Bond et al. 2009).

##### 5.5.1.1 LCAs

Three studies stated that they used LCA methods to evaluate an intervention which changed access to care. All three studies evaluated the effectiveness of telehealth or virtual care interventions and indicated a reduction in carbon emissions following intervention implementation (Holmner et al. 2014; Sillcox, Gitonga, et al. 2023; Thiel et al. 2023). One study appraised as Medium risk of bias was conducted within gastroenterology and estimated GHG emissions ranged from 2.26 to 2.99 kg CO_2_eq, 25 times less than emissions produced during telemedicine visits (*p* < 0.01) (Sillcox, Gitonga, et al. 2023). Two studies appraised as Low risk of bias were relevant to multiple specialities (Holmner et al. 2014; Thiel et al. 2023). Within a hand/plastic surgery clinic, the carbon cost of 238 telemedicine appointments was estimated at 602 kg CO_2_eq (1.4%–2.8% of face‐to‐face appointments) (Holmner et al. 2014). The other study calculated a reduction in GHG emissions of approximately 17,000 metric tonnes through using virtual care versus in‐person treatment (per visit, virtual medicine emits < 1% GHGs in‐person visit (range 0.02–0.08 kg CO_2_eq/visit, depending on department) (Thiel et al. 2023). Further details regarding carbon emission findings and other outcomes reported are summarised in Table 3 below.

**Table 3:** Accessing care – Outcomes from LCA studies.

Study, speciality: Study design (Comparison)	Carbon emission findings	Other outcomes											
		Patient travel saved	Cancellations	Ozone depletion	Smog	Acidification	Eutrophication	Carcinogenics	Non‐carcinogenics	Respiratory effects	Ecotoxicity	Fossil fuel depletion	Costs
*Telehealth*													
Holmner (2014), Hand and plastic surgery: Inventory analysis (C1: Telerehab vs. C2: Face‐to‐face)(Holmner et al. 2014)	***Favours C1 (telemedicine):*** Hand/plastic surgery clinic: carbon cost of 238 telemedicine appointments = 602 kg CO_2_eq (average of 1.4%–2.8% of carbon costs of travelling to/from clinic by car or subsidised taxi services, and total avoided travel distance of 82,310 km for patients. Based on upper + lower bound scenarios, 1× telerehabilitation visit generated 0.4%–0.9% and 3.2%–6.4% of carbon costs for 1× face‐to‐face appointment. Similar numbers obtained in speech therapy clinic. Summary: telerehabilitation activities of two clinics cut carbon emissions by 15–250 times for telemedicine work model compared versus traditional care. Based on the upper and lower bound scenarios, 1 × 1 h telemedicine appointment estimated to generate 1.86 and 8.43 kg CO_2_eq, respectively. Telerehabilitation carbon cost‐effective if patient travels min. 3.6 km by car for 1 × 1 h appointment (using Lenzen estimate)^a^ or 7.2 km (based on the Leduc estimate)^b^ Corresponding values for the upper bound videoconference scenario: 16 and 32 km, respectively.	C1 > C2 [N]											
Sillcox (2023), GE: Inventory analysis (C1: Telerehab vs. C2: Face‐to‐face)(Sillcox, Gitonga, et al. 2023)	***Favours C1 (telemedicine):*** In‐person visits: 145 patient travel distances recorded (median [IQR] distance travel distance of 29.5 [13.7, 85.1] miles) = 38.22–39.61 kg CO_2_eq emitted. Telemedicine visits: Mean (SD) visit time = 40.6 (17.1) min. Telemedicine GHG emissions ranged from 2.26 to 2.99 kg CO_2_eq depending on device used. In‐person visit = 25 times more GHG emissions compared to telemedicine visit (*p* < 0.001).	C1 > C2 [N]	><										
Thiel (2023), Multiple: LCA (C1: Virtual Care vs. C2: Face‐to‐face)(Thiel et al. 2023)	***Favours C1 (virtual care):*** VC system reduced 2021 GHG emissions by nearly 17,000 metric tons versus in‐person treatment, equivalent of over 2100 homes energy use/year or CO_2_ sequestered by nearly 20,000 acres of US forest in 1 year. 25 Departments with largest growth of telemedicine: psychiatry (88% visits virtual in 2021), medical specialties (73%), pain management (68%), GI surgery (63%), and cancer (47%). Specialties with smaller increases in telemedicine: ophthalmology (1% of visits were virtual in 2021), plastic surgery (7%), orthopaedics (11%), and otolaryngology (18%). Emissions/patient ranged by department and visit type. In‐person visits: primary care and paediatrics emitted least per visit (7.33 kg CO_2_eq/visit). Orthopaedics: largest per visit emissions (63.8 kg CO_2_eq/in‐person visit). Per visit, virtual medicine emits < 1% GHGs of in person visit, [range of 0.02 to 0.08 kg CO_2_eq/visit, depending on department]. Assumed mode of patient travel has largest impact on model. When modelled all patient travel as occurring via passenger car, in‐person visit emissions increased 77%, from 25,700 metric tons to 45,400 metric tons. For in‐person visits, energy sources had little influence on emissions outcomes. Solar power reduced modelled emissions slightly and US average grid mix increased emissions slightly. Changing assumed energy intensity of clinics for in‐person visits, or for in‐person visits avoided by virtual visits, did not change outcomes. Transportation of patients dominates SHC's per‐visit emissions. For VC specifically, changes to energy sources did impact modelled emissions, with solar reducing virtual visit emissions nearly 70% and US grid mix leading to 20% increase in estimated telehealth emissions. A maximum supply list, though unrealistic for most clinical visits, increased total GHG emissions from all in person 2021 visits by about 1.1% or 277,000 kg CO_2_eq. Modes of transportation change study results, with large caveat that access to various modes of transit are limited, for example, aircraft useless for short‐distance travel, bike useless for long‐distance, an appropriate bus route may not be accessible, or car may be unaffordable. Therefore, analysis speaks only to theoretical changes to emissions rather than practical changes.			C1 > C2 (S)	C1 > C2 (S)	C1 > C2 (S)	C1 > C2 (S)	C1 > C2 (S)	C1 > C2 (S)	C1 > C2 (S)	C1 > C2 (S)	C1 > C2 (S)	

*Note:* Green shaded cell: Study appraised as Low risk of bias; Blue shaded cell: Study appraised as Medium risk of bias; Grey shaded cell: No data, C1 > C2 – Analysis favoured Comparator 1 over C2, [N] – supported by narrative write up (no formal statistics), [S] – calculated using formal statistics,

Abbreviations: C, comparator; CA, component analysis; CO_2_eq, carbon dioxide equivalent; GE, gastroenterology; GHG, greenhouse gas; GI, gastrointestinal; h, hour; HHD, home haemodialysis; HVAC, heating, ventilation, and air conditioning; ICHD, in‐centre haemodialysis; IQR, interquartile range; kg CO2 eq, kg carbon dioxide equivalents; kWh, kilowatt hours; MRI, magnetic resonance imaging; SD, standard deviation; SHC, stanford health care; TRUS, transrectal ultrasound; US, United States; VC, virtual care.

^a^ Lenzen, M. 1999. Total Requirements of Energy and Greenhouse Gases for Australian Transport. *Transportation Research Part D: Transport and Environment* 4: 265–290.

^b^ Leduc, G., I. Mongelli, A. Uihlein, and F. Nemry. 2010. How Can Our Cars Become Less Polluting? An Assessment of the Environmental Improvement Potential of Cars. *Transport Policy* 17: 409–419.

One study appraised as Low risk of bias completed a full impact assessment, which indicated a significant difference between intervention and face‐to‐face control groups in favour of the virtual care intervention for the following impact categories: Ozone depletion, smog, acidification, eutrophication, carcinogenics, non‐carcinogenics, respiratory effects and ecotoxicity (Thiel et al. 2023).

Patient outcomes were limited to the non‐statistical comparison of distance travelled (*n* = 2) (Holmner et al. 2014; Sillcox, Gitonga, et al. 2023), or number of cancellations (*n* = 1) between groups (Sillcox, Gitonga, et al. 2023), both of which favoured the telemedicine intervention or showed no difference.

##### 5.5.1.2 Non‐LCA Studies

###### 5.5.1.2.1 Telemedicine

Twenty‐three studies using a non‐LCA approach to evaluate interventions which focused on the remote delivery of services through telemedicine or video conferencing in comparison to face‐to‐face care (Andrew et al. 2020; Arndt et al. 2023; Beswick et al. 2016; Connor, Mortimer, et al. 2011; Connor et al. 2019; Croghan et al. 2021; Curtis et al. 2021; Dorrian et al. 2009; Filfilan et al. 2021; Jiang et al. 2021; King et al. 2022; Lambert et al. 2023; Lewis et al. 2009; McLachlan et al. 2021; Miah et al. 2019; Mojdehbakhsh et al. 2021; Muschol et al. 2022; Natale et al. 2022; Patel et al. 2023; Richards et al. 2022; Sillcox, Blaustein, et al. 2023; Thota et al. 2020; Tselapedi‐Sekeitto 2023; Udayaraj et al. 2019). Six observational studies were conducted within oncology/radiation oncology services (Beswick et al. 2016; Jiang et al. 2021; Lambert et al. 2023; Lewis et al. 2009; Patel et al. 2023; Thota et al. 2020), five observational studies were conducted within urology services (Connor et al. 2019; Croghan et al. 2021; Filfilan et al. 2021; Miah et al. 2019; Natale et al. 2022), four studies were conducted within the orthopaedics and/or trauma speciality (two observational [Curtis et al. 2021; Richards et al. 2022], and two using an experimental comparative study design [Arndt et al. 2023; Muschol et al. 2022]) three observational studies were conducted within the renal speciality (Andrew et al. 2020; Connor, Mortimer, et al. 2011; Udayaraj et al. 2019), two observational studies were conducted within gastroenterology (King et al. 2022; Sillcox, Blaustein, et al. 2023), two studies were conducted within ENT services (one observational [Dorrian et al. 2009], the other a prospective comparative study [Tselapedi‐Sekeitto 2023]), one before and after study was conducted within cardiology (McLachlan et al. 2021), and one observational study was carried out within gynaecological services (Mojdehbakhsh et al. 2021). Further details regarding individual study design can be found in Appendix 7, Accessing care – overview of non‐LCA studies.

All of the studies reported reduced carbon emissions within the telehealth intervention group, with carbon emission type, units and timeframes varying considerably between studies. Estimates of carbon emissions saved varied from total CO_2_ emissions reduction: 607 kg (McLachlan et al. 2021) to 51 tonnes CO_2_eq (Andrew et al. 2020). Full details for carbon emission outcomes for non‐LCA studies can be viewed in Appendix 7.

For specialities supported by data from more than one study, the following carbon emission savings were reported:

Three studies report carbon savings associated with telemedicine‐style interventions within a renal setting, with carbon savings reported as: a total of 51 tonnes of GHG emissions CO_2_eq (Anderew et al. 2020), an annual reduction of GHG emissions of 2818 kg CO_2_eq based on clinician travel saved (Connor, Mortimer, et al. 2011), and a total saving of 1035 kg CO_2_ based on patient‐reported travel miles saved (Udayaraj et al. 2019).

Four studies reported carbon savings associated with telemedicine/virtual care style interventions versus face‐to‐face care within an orthopaedics and trauma setting. Carbon savings reported included a significant reduction (< 0.001) in GHG emissions over 0.5 tons CO_2_eq (Arndt et al. 2023), a total carbon emissions reduction of 563.9 kg CO_2_eq or 3.1 kg CO_2_eq/person (Curtis et al. 2021), and a total GHG emissions saving (based on 26 patients in telehealth vs. face‐to‐face group) of 292.448 kg (Muschol et al. 2022), and, for 52 patients, a total carbon saving of between 2912 and 3952 kg CO_2_eq for a telehealth group, to a lower estimate of 3270 kg CO_2_eq or 62.9 kg CO_2_eq/patient per appointment (Richards et al. 2022).

Two studies reported carbon savings of telehealth interventions compared to face‐to‐face care in ENT specialities. Reported carbon savings included 123 kg CO_2_/person return journey total emissions saved for a return patient journey (Dorrian et al. 2009), and an estimate of environmental impact generated by in‐person group visits of 32 ± 39 kg CO_2_ emitted per consultation (Tselapedi‐Sekeitto 2023).

Five studies reported carbon savings associated with telemedicine interventions within a urology speciality. Savings reported included a total of 0.70–2.93 metric tonnes CO_2_eq (Connor et al. 2019), 6.07 tonnes CO_2_ (Croghan et al. 2021), 1.1 tonnes over 1 month (Filfilan et al. 2021), 1.05–4.35 metric tonnes CO_2_eq over 12 months (Miah et al. 2019), and a total saving of 637 kg CO_2_ (Natale et al. 2022).

Six studies calculated carbon emission reductions associated with telehealth interventions within oncology settings. Reported reductions were as follows:14.5 metric tons CO_2_ emissions (Beswick et al. 2016), a total of 35.5 metric tons CO_2_ based on 560 oncology encounters, or 106 metric tons of CO_2_ annually (Jiang et al. 2021), 87–55 metric tons/month (Lambert et al. 2023), total savings of 1696 and 2590 kg CO_2_eq across October 2006 and October 2007 (Lewis et al. 2009); savings of 19.8 kg CO_2_/visit for patients within 60 min of clinic and 98.6 kg CO_2_ savings for patients living over 60 min away (Patel et al. 2023), and a reduction of 150,000 kg for 119 patients over a 4‐year period (Thota et al. 2020).

Two studies reported carbon reduction savings within gastroenterology settings. One study reported an overall reduction of 1159.92 kg CO_2_eq (99.37%; *p* = 0.0001), although found no significant difference in carbon emissions between non‐tertiary and tertiary sites overall (group 1: *p* = 0.62, group 2: *p* = 0.95) or when adjusting for no. appointments (group 1: *p* = 0.45, group 2: *p* = 0.89) (King et al. 2022). The other study reported that in‐person visits resulted in 25 times greater GHG emissions compared to telemedicine visit (*p* < 0.001) (Sillcox, Blaustein, et al. 2023).

However, most carbon‐emission calculations considered only patient‐travel saved and did not account for carbon emissions associated with other parts of the system. For example, energy associated with infrastructure use within patient homes and healthcare facilities where staff are based, or emissions associated with the extraction, manufacture, transport and/or disposal of relevant materials, for example, car/petrol production, were not often considered. This is reflected in the limited range of patient outcomes measured, of which patient travel distance (*n* = 12) (Andrew et al. 2020; Connor, Mortimer, et al. 2011; Connor et al. 2019; Croghan et al. 2021; Jiang et al. 2021; Lambert et al. 2023; Miah et al. 2019; Mojdehbakhsh et al. 2021; Patel et al. 2023; Sillcox, Blaustein, et al. 2023; Thota et al. 2020; Udayaraj et al. 2019), and time saved (*n* = 9) were the most common (Andrew et al. 2020; Arndt et al. 2023; Beswick et al. 2016; Croghan et al. 2021; Filfilan et al. 2021; Jiang et al. 2021; Lambert et al. 2023; Muschol et al. 2022; Tselapedi‐Sekeitto 2023). Patient satisfaction (*n* = 3) (Andrew et al. 2020; Curtis et al. 2021; Tselapedi‐Sekeitto 2023), safety (e.g., adverse events) (*n* = 3) (Curtis et al. 2021; King et al. 2022; Sillcox, Blaustein, et al. 2023), and acceptability (i.e., attendance) (*n* = 5) were poorly reported (King et al. 2022; Muschol et al. 2022; Natale et al. 2022; Sillcox, Blaustein, et al. 2023; Udayaraj et al. 2019). The retrospective nature of these studies meant that while some data for these key outcomes were reported for the intervention group, complementary comparative data for the control group were often absent.

Overall, the results for most patient outcomes measured favoured the telehealth intervention group. Sixteen studies considered costs to patients (*n* = 10) (Andrew et al. 2020; Beswick et al. 2016; Croghan et al. 2021; Curtis et al. 2021; Filfilan et al. 2021; Jiang et al. 2021; Lambert et al. 2023; Muschol et al. 2022; Richards et al. 2022; Thota et al. 2020), and/or services (*n* = 6) (Connor et al. 2019; Dorrian et al. 2009; Lewis et al. 2009; Miah et al. 2019; Natale et al. 2022; Udayaraj et al. 2019). Findings were mainly based on narrative/descriptive cost‐calculations (i.e., were not formal cost‐effective studies and did not use statistical tests to establish if there was a significant difference between groups), indicating telehealth interventions were associated with reduced costs for both patients and services compared to face‐to‐face care. The exception to this was a feasibility study by Dorrian et al. (2009), which indicated that face‐to‐face care costs less than consultants supervising patient examination via videoconferencing software in a sample of 29 patients/year (Dorrian et al. 2009). In this study, the authors indicated that the threshold at which tele‐ENT became cheaper than travel (35 patients/year) was not met within the pilot study (see Supporting Material S4).

As indicated above, most studies within this category used observational study designs; thus, findings may be more susceptible to bias than findings arising from experimental studies using comparative study designs.

###### 5.5.1.2.2 De‐Centralised Care

Three studies evaluated interventions aiming to decentralise care, with carbon emission reduction calculations, which were predominantly based upon emissions associated with travel, being in favour of the care pathway intervention across all three studies. Interventions included an outreach clinic for head and neck cancer patients with a high estimate of emissions saved of 143,570.9 g over 3‐months for 100 patients (SD 29,040.0) (Forner et al. 2021), a breast cancer screening clinic with an estimated annual saving of 75 tonnes CO_2_ emissions (Bond et al. 2009), or modelling of optimal delivery systems for home haemodialysis (HHD) equipment, with an estimated 21% drop in carbon emissions (Asghari and Al‐e‐Hashem 2020). Study designs were a retrospective database review, a cross‐sectional survey and one modelling study.

The only other patient outcome measured was distance to travel to point of care, which was reduced by the care pathway intervention in one study (if all women had attended the nearest hospital site, the total (one‐way) distance would have been 1,102,715 km) (Bond et al. 2009). Cost outcomes were measured in one study, which indicated that an intervention facilitating direct sharing of equipment between users reduced costs by 25% when compared to delivery via a central depot (Asghari and Al‐e‐Hashem 2020).

Further detail regarding the non‐LCA studies evaluating interventions within the ‘Accessing Care’ category can be found in Appendix 7, Accessing care – overview of non‐LCA studies and Supporting Material S4.

#### 5.5.2 Setting

**Summary: Setting**

Twenty studies evaluated interventions which focused on changing behaviours within the wider healthcare delivery system. Interventions could be separated into two categories; waste management (*n* = 12) (Betts 2022; Bird 2022; Chambrin et al. 2023; de Ridder et al. 2022; Kodumuri et al. 2023; Kodumuri 2022; Lopez‐Munoz et al. 2023; Neves et al. 2022; Owens 2023; Vo et al. 2023; Winklmair et al. 2023; Yong et al. 2022), and energy conservation (*n* = 8) (Buttner et al. 2021; Heye et al. 2023; Klein 2023; McAlister et al. 2022; McCarthy et al. 2014; Milne 2010; Milne 2023; Woolen et al. 2023). Four of these studies were informed by LCA methods and are described further below (de Ridder et al. 2022; Lopez‐Munoz et al. 2023; McAlister et al. 2022; Winklmair et al. 2023).

***LCA studies*** **(*n*** = 4**):** Three studies drawing on LCA methods, appraised as ‘Medium’ or ‘High’ risk of bias, reported reductions in carbon emissions achieved through waste reduction interventions. Interventions were highly heterogeneous, and no other outcomes aside from waste reduction were measured. One inventory analysis appraised as Low risk of bias indicated CT and was associated with the highest carbon emissions.

**Non‐LCA studies (*n*** = **16):** Nine studies indicated carbon emissions were reduced following a waste management intervention, although calculations were often based on a narrow range of processes/stages within the systems being evaluated. Only four studies evaluated patient satisfaction (Bird 2022; Owens 2023), and/or patient clinical outcomes (Betts 2022; Bird 2022; Neves et al. 2022), with six studies reporting on service cost (Betts 2022; Bird 2022; Chambrin et al. 2023; Kodumuri et al. 2023; Kodumuri 2022; Neves et al. 2022; Owens 2023). All other outcomes favoured the waste management intervention being evaluated. The quantity of data available for synthesis was limited by the number of studies providing data on each outcome for each comparator. Seven studies used a non‐LCA approach to evaluate energy conservation interventions. The majority of these were conducted within radiology/radiotherapy settings (*n* = 5) (Buttner et al. 2021; Heye et al. 2023; Klein 2023; McCarthy et al. 2014; Woolen et al. 2023), four focusing on reducing energy associated with equipment when not in use (Buttner et al. 2021; Heye et al. 2023; McCarthy et al. 2014; Woolen et al. 2023), and one evaluating the construction and operation of an energy optimised medical centre (Klein 2023). The majority of studies found reduced carbon emissions associated with the intervention. Two related studies modelled the potential environmental and cost impacts associated with retrofitting heat exchangers to haemodialysis machines and reported findings that favoured the intervention condition (Milne 2010, 2023).

##### 5.5.2.1 LCAs

Details regarding findings for carbon emissions and other outcomes are reported in Table 4, with further information regarding study design and participants reported in Appendix 8, Setting – overview of non‐LCA studies and Supporting Material S4.

**Table 4:** Setting – Overview of main findings from LCA studies.

Study: Speciality (Study design)	Name of Interventions (C1 vs. C2, etc.)	Carbon emission findings (based on summary of reported findings for each study)	Summary findings: other outcomes					
			Time spent standby mode	Time/scan	Power consumption	Waste incineration	Waste reduction	Staff satisfaction
McAlister (2022): Radiology/Radiotherapy (Inventory analysis)^a^ (McAlister et al. 2022)	Image type C1: CXR versus C2: Ultrasound versus C3: MCXR versus C4: CT versus C5: MRI	***C4 (CT) and C5 (MRI) largest environmental impact:*** ALCA: MRI and CT had highest emissions = 17.5 and 9.2 kg CO₂eq per scan, respectively. Majority of impact (MRI – 94%, CT – 91%) resulting from electricity use. MRI‐impact of consumables came predominantly from cotton drawsheets (0.7 kg CO₂eq, or 4% total impact). For CT: originated primarily from contrast tubing, cotton sheet and pillowcase (both 0.4 kg CO₂eq, 4%) and contrast tubing (0.3 kg CO₂eq, 3%). US and CXR had similar carbon impacts (0.76 and 0.53 kg CO₂eq, respectively). While dominant emissions source for US (as for MRI and CT) is electricity (87%), for CXR = washing/drying of cotton sheet and pillowcase (0.67 kg CO₂eq, 88%), with electricity only contributing 0.02 kg CO₂eq, or 3% of total impact. CLCA: Carbon emissions for MRI, CT and US were 84%–94% lower compared to ALCA, due to exclusion of standby power in calculated impact. MRI and CT remained imaging modalities with largest impacts (1.1 and 1.09 kg CO₂eq respectively). For CXR, impact fell only slightly from 0.8 to 0.6 kg CO₂eq compared to ALCA, as main source of impact (sheet/pillowcase laundering) remained same.	All imaging devices spent more time in standby mode versus active mode (range 67% MRI time in standby to 99.6% for X‐ray).	C1 < C3 < C4 < C2 < C5	MRI highest mean power consumption/minute of operation in active and standby, had lower attributional power consumption versus CT due to CT scanner being in standby longer than MRI scanner (92% time vs. 67%) so each active minute of CT scanner having greater number standby minute.^b^			
De Ridder (2022): Obstetrics [HSMEA] (de Ridder et al. 2022)	C1: Waste reduction decision tool C2: Usual care	***Favours C1 (Decision tool):*** Sustainable solutions in preparation room and OR for C‐section: waste reduction of 600 g (−22%) and a carbon footprint reduction of 2.5 kg CO_2_eq (−22%). Of total CO_2_ footprint reduction, 98% attributable to revision of custom pack, 2% from paper/plastic recycling					C2 > C2 (N)	NCD
López‐Muñoz (2023): GE (Inventory Analysis) (Lopez‐Munoz et al. 2023)	C1: Recycling (Green mark intervention) C2: Biowaste	***Favours C1 (Green mark intervention):*** Reduction of 34.3% of emissions (95% CI: 28.1%–40.3%). GHG emissions reached up to 67.74 kg CO_2_eq during our 1‐week prospective study. Sustainability intervention could reduce environmental impact up to 27.44% (18.26 kg CO_2_eq). This allows recycling of 61.7% of the instrument total weight (4.69 kg).				C1 > C2 (N)		
Winklmair (2023): Ophthalmology (Inventory analysis)(Winklmair et al. 2023)	C1: Recycling components of cataract package versus C2: Incineration	***Favours C1 (Recycling):*** Cataract packages of three hospitals contained average 0.74 kg materials = 2.3 kg CO_2_eq/package. (not including: phaco cassettes, tubing, infusions with cutlery, other cataract package external disposables). GWP for all cataract packages sold in 2021 with an 100% assumed waste incineration rate for all products was 209,380 kg CO_2_eq (2.4 kg CO_2_eq/cataract package). With assumed recycling rate of 100% of all technically recyclable materials (i.e., packaging materials not contaminated in OR), carbon footprint was 195,804 kg CO_2_eq (2.2 kg CO_2_eq/cataract package). Difference in CO_2_ effect between cataract packages with 100% incineration and those with 100% recyclable materials was, therefore, approximately 6.5% (13,576 kg CO_2_eq).						

*Note:* Green shaded cell: Study appraised as Low risk of bias; Blue shaded cell: Study appraised as Medium risk of bias; Orange shaded cell: Study appraised as High risk of bias; Grey shaded cell: No data C1 > C2: favours C1.

Abbreviations: ALCA, attributional life cycle assessment; C, comparator; CI, confidence interval; CLCA, consequential life cycle assessment; CO_2_eq, carbon dioxide equivalent; CT, computerised tomography; CXR, chest X‐ray; GE, gastroenterology; LCA, life cycle assessment; MCXR, mobile chest X‐Ray; MRI, magnetic resonance imaging; N, narrative synthesis; NCD, no comparative data; OR, operating room; US, ultrasound.

^a^ Stated as LCA, but incomplete impact assessment.

^b^ ALCA power consumption higher than both mean power consumption and CLCA power consumption due to high proportion of time spent in standby for all modalities.

###### 5.5.2.1.1 Waste Management

Two studies undertook an inventory analysis approach to establish the impact of waste management interventions on carbon emissions (Lopez‐Munoz et al. 2023; Winklmair et al. 2023), with a further study undertaking a ‘Healthcare Sustainability Mode and Effect Analysis’ (de Ridder et al. 2022). Two were appraised as Medium risk of bias (de Ridder et al. 2022; Lopez‐Munoz et al. 2023), and one as High risk of bias (Winklmair et al. 2023). Overall, reductions in carbon emissions were achieved through waste reduction interventions which included a waste reduction decision tool for use in the operating room (OR) with a carbon footprint reduction of 2.5 kg CO_2_eq (−22%) (de Ridder et al. 2022), maximising recycling of components of surgical instruments associated with a reduction of 34.3% of emissions (95% CI: 28.1%–40.3%) (Lopez‐Munoz et al. 2023), and increased recycling of different parts of packaging of instruments used during cataract surgery resulting in estimated reduction of 6.5% (13,576 kg CO_2_eq) (Winklmair et al. 2023). No other outcomes aside from waste reduction were measured.

###### 5.5.2.1.2 Energy Conservation

One study, using an inventory analysis approach and appraised as Low risk of bias, compared carbon emissions associated with different types of scan types (McAlister et al. 2022), and reported that highest carbon emissions were attributable to magnetic resonance imaging (MRI) and CT scans (carbon emissions/scan (CO₂eq) of 17.5 kg/scan, 9.2 kg/scan, respectively) when compared to ultrasound, mobile X‐ray and X‐ray (0.8 kg/scan, 0.5 kg/scan and 0.5 kg/scan, respectively). MRI had the highest mean power consumption and time spent per scan, spending 67% of its time in standby mode (McAlister et al. 2022).

##### 5.5.2.2 Non‐LCA

###### 5.5.2.2.1 Waste Management

Nine studies used a non‐LCA approach to evaluate various waste management interventions. Overall, carbon emissions were reduced as a result of waste management interventions, although calculations were often based on a narrow range of processes/stages within the systems being evaluated. Four studies were conducted within gastroenterology settings (Betts 2022; Neves et al. 2022; Owens 2023; Yong et al. 2022). One study reported a combined carbon emission saving of 921.44 kg CO₂eq for a water bottle recycling and electronic Campylobacter‐like organism testing interventions (Betts 2022). The second reported a reduction in the carbon footprint of 31.6% (138.8 kg CO₂eq) 1 month after a 1‐week waste handling/segregation intervention (Neves et al. 2022). The third study evaluated a combined intervention focusing on reducing paper and contrast waste across three units, with an estimated saving of 6147.48 kg CO₂eq/year (Owens et al. 2023). The fourth study evaluated the carbon emission savings associated with reducing the number of specimen pots used, with pot usage determined by clinical features of polyps during removal for three patient groups (see Appendix 8 for further detail) (Yong et al. 2022). This study reported a reduction in carbon footprint to 572, 490, and 289 kg CO₂eq, respectively, with the reduction in carbon footprint by putting all small polyps in a pot for the whole colon, in comparison with one pot per hemi colon being statistically significant (*p* < 0.00001) (Yong et al. 2022). One study calculated a carbon emission reduction of 1219.9 kg CO₂eq/year following the introduction of a new postal system within a renal setting, based on an average of 39 low clearance/pre‐dialysis patients needing 3 monthly blood tests (Bird 2022). One study calculated the reduction in carbon footprint related of inhaled halogenated anaesthetics across multiple settings following an information campaign (Chambrin et al. 2023), reporting a reduction median carbon footprint associated with perioperative desflurane from 271.1 tons to 22.4 tons, and from 12.3 tons to 22.2 tons for sevoflurane (reduction of median emissions from perioperative inhaled halogenated anaesthetics from 66.2 kg CDE100/general anaesthesia to 6.5 kg CDE100/general anaesthesia when weighted by surgical activity) (Chambrin et al. 2023). One study reported an 80% carbon emission reduction (6.6 kg (range: 6.2–7.3)) within an orthopaedics/trauma setting following implementing a ‘lean and green model’ – carpal tunnel release system (Kodumuri et al. 2023; Kodumuri 2022). The final study analysed the environmental impact of reusing shipping materials relating to procedures within an ophthalmology setting, estimating a total reduction of carbon emissions (CO₂eq) by 43% (Vo et al. 2023).

A heterogeneous range of other outcomes was measured. Only four studies evaluated patient satisfaction (Bird 2022; Owens 2023), and/or patient clinical outcomes (Betts 2022; Bird 2022; Neves et al. 2022). The quantity of data available for synthesis was limited by the number of studies providing data on each outcome for each comparator. Six studies reported on service costs (Betts 2022; Bird 2022; Chambrin et al. 2023; Kodumuri et al. 2023; Kodumuri 2022; Neves et al. 2022; Owens 2023). All other outcomes favoured the waste management intervention being evaluated, with specific details reported within Appendix 8 and Supporting Material S4.

###### 5.5.2.2.2 Energy Conservation

Seven studies used a non‐LCA approach to evaluate energy conservation interventions. The majority of these were conducted within radiology/radiotherapy settings (*n* = 5) (Buttner et al. 2021; Heye et al. 2023; Klein 2023; McCarthy et al. 2014; Woolen et al. 2023), and two studies were conducted within a renal setting (Milne 2010; Milne 2023).

Of the five studies conducted within a radiology setting, four focused on reducing energy associated with equipment when not in use (Buttner et al. 2021; Heye et al. 2023; McCarthy et al. 2014; Woolen et al. 2023). Two of these four studies used a modelling approach (Buttner et al. 2021; Woolen et al. 2023), with the first estimating a potential saving of 22.2 tonnes of CO_2_/year and 14,388.28 USD/year following consistent automatic shutdown after core working hours (Buttner et al. 2021). The second modelling study calculated carbon emission reductions associated with different power‐saving scenarios for MRI use, with annual carbon savings ranging from 8.7 to 14.9 MTCO₂ eq with an additional 11.2–19.2 MTCO₂ eq from switching from off to power‐save mode (Woolen et al. 2023). The other two studies used a before‐and‐after design (Heye et al. 2023; McCarthy et al. 2014). One demonstrated that providing the results of a 1 week energy audit at a department meeting was not effective in reducing the number of desktop computers left on overnight, which may indicate that provision of information alone is insufficient to change staff behaviour (McCarthy et al. 2014). The second study identified a potential 9.26 metric tons in CO_2_ emissions through a Python descript designed to trace activity from idle energy‐consuming imaging and electronic devices (Heye et al. 2023). One retrospective comparative study compared the energy use across the building and operation of two radiological facilities using different energy‐friendly and/or regenerative technology, reporting an annual CO_2_ reduction of 54% from 153,146 to 70,631 kg/year (carbon emission savings associated with each condition are reported in Appendix 8, Setting – overview of non‐LCA studies) (Klein 2023). All five studies calculated potential or actual energy and costs saved, which favoured the intervention.

Two studies modelled the potential environmental and cost impacts associated with retrofitting heat exchangers to haemodialysis machines (Milne 2010, 2023), reporting carbon emissions savings favouring the intervention of 272.33 kg (0.272 tonnes) (Milne 2010), and 316.5 kg (0.3165 tonnes) CO₂eq per machine/year (Milne 2023).

#### 5.5.3 Product Level

**Summary: Product Level**

Sixteen studies evaluated interventions at the product level. Specialties represented by these studies include urology (*n* = 6) (Baboudjian et al. 2022; Davis et al. 2018; Hogan et al. 2022; Kemble et al. 2023; Stripple et al. 2008; Wombwell et al. 2023), gastroenterology (*n* = 4) (Boberg et al. 2022; Le et al. 2022; Rizan and Bhutta 2022a; Sherman et al. 2018; Yong et al. 2022), ophthalmology (*n* = 1) (Moussa et al. 2022; Moussa et al. 2021), cardiology (*n* = 1) (Schulte et al. 2021), respiratory (*n* = 1) (Sørensen and Grüttner 2018), orthopaedic and/or trauma (*n* = 2) (Chan 2023; Leiden et al. 2020), and multiple specialties (*n* = 1) (Field et al. 2023). Thirteen of these studies used LCA‐informed methodology (Baboudjian et al. 2022; Boberg et al. 2022; Davis et al. 2018; Hogan et al. 2022; Kemble et al. 2023; Le et al. 2022; Leiden et al. 2020; Rizan and Bhutta 2022a; Schulte et al. 2021; Sherman et al. 2018; Sørensen and Grüttner 2018; Stripple et al. 2008; Wombwell et al. 2023).

**LCA studies (*n*** = **13):** Thirteen studies, appraised as predominantly ‘High’ or Medium risk of bias, used LCA or inventory analysis methods to explore carbon emissions associated with reuseable equipment. Overall, reduced carbon emissions were associated with the use of reuseable equipment when compared to single‐use within gastroenterology specialities. Findings from urology specialities relating to reuseable equipment were more mixed. Studies finding in favour of reuseable equipment in terms of carbon emissions reported reduced impact (or little difference) in the majority of other environmental impact categories; and vice versa for studies reporting reduced carbon emissions associated with disposable equipment. Two studies evaluated costs of reuseable equipment within gastroenterology settings, both concluding reuseable/hybrid equipment costs less than disposable (Boberg et al. 2022; Sherman et al. 2018). Patient‐reported outcomes were not measured.

**Non‐LCA studies (*n*** = **3):** Heterogeneity in speciality and intervention type precluded meaningful analysis.

##### 5.5.3.1 LCAs

The findings relating to carbon emissions and other impact categories for the 13 LCA or inventory analysis studies are described narratively below within two sub‐categories: reuseable equipment and equipment composition. A summary of this information can be found in Table 5, with further information on outcomes from other impact categories and outcomes within Appendix 9, Product level – additional tables and Supporting Material S3.

**Table 5:** Product‐level – Overview of main findings from LCA studies.

Study, speciality: Study design	Name of Interventions compared (C1 vs. C2, etc.)	Carbon emission findings (based on summary of reported findings for each study)	Summary other impact categories
*Reuseable equipment*			
Baboudjian (2022), Urology; LCA (Baboudjian et al. 2022)	C1: Reuseable flexible cystoscopes versus C2: Single‐use cystoscope	***Favours C2 (Single‐use):*** Use of Single‐use aScope would allow a reduction of at least 33% on the climate change category (i.e., 33% reduction in CO_2_ emissions) compared with just disinfection reprocessing of reusable cystoscopes. For both devices main emissions generated during initial manufacture of materials and assembly of the device, regardless of impact category assessed.	***Four impact categories: 2 Favoured SU:*** Mineral resource depletion Acidification. ***2 No difference:*** Ecotoxicity, Eutrophication.
Hogan (2022), Urology; Prospective single‐centre cohort study: controlled trial/Simplified LCA^a^ ^,^ ^b^ (Hogan et al. 2022)	C1: Reusable versus C2: Disposable flexible cystoscopes	***Favours C2 (Single‐use):*** SU cystoscope weighs 158 g in total (146.31 g plastic, 6.32 g steel, 2.84 g electronics, and 2.53 g of rubber), giving manufacturing carbon footprint of 1.34 kg of CO₂/cystoscope. Solid waste disposed via incineration after SU flexible cystoscopy produced median 0.61 kg of CO₂ (IQR: 0.50–0.64), waste to landfill producing 0.11 kg of CO₂ (IQR: 0) per case. Sterilisation of SU endoscopes produces 0.3 kg of CO₂ (IQR: 0) per endoscope^c^ Transport of each SU cystoscope from manufacturing factory in Malaysia produced 0.049 kg of CO₂. Total median carbon footprint: 2.41 kg CO₂ (IQR: 2.40–2.44) per case for the SU flexible cystoscope. Manufacturing production of CO₂ of a reusable cystoscope based on weight 1.3 kg for Olympus cystoscope = 14.94 kg of CO₂/cystoscope. Each reuseable cystoscope performs approx. 1120 cystoscopies/lifetime = 0.013 kg of CO₂ (IQR: 0) per case. Solid waste disposal by incineration after reusable flexible cystoscopy = median of 0.52 kg of CO₂ (IQR: 0.51–0.60). Waste to landfill = 0.22 kg of CO₂ (IQR: 0) per case. Sterilisation performed within the department using Olympus ETD‐DoubleTM can reprocess up to three cystoscopes per cycle, consuming 10.5 kW of electricity, equating to 10.5 kg of CO₂ per cycle (3.5 kg of CO₂ [IQR: 0] per case).^c^ Total median carbon footprint significantly higher at 4.23 kg of CO₂ (IQR: 4.22–4.24) per case for reusable flexible cystoscope (*p* < 0.0001).	***One impact category: Favoured disposable*** – Solid Waste produced.
Kemble (2023), Urology; Inventory analysis (Kemble et al. 2023)	C1: SU versus C2: Reusable cystoscopes	***Favours C2 (Reuseable):*** A fleet of 16 reusable cystoscopes in service for up to 135 months averaged 207 cases between repairs and 3920 cases per lifecycle. Based on manufacturing carbon footprint of 11.49 kg CO₂/kg device for reusable flexible endoscopes and 8.54 kg CO₂/kg device for SU endoscopes, per‐case manufacturing cost was 1.37 kg CO₂ for SU devices and 0.0017 kg CO₂ for reusable devices. Solid mass of SU and reusable devices was 0.16 and 0.57 kg, respectively. For reusable devices, energy consumption of reusable device reprocessing using automated endoscope reprocessor = 0.20 kg CO₂, and per‐case costs of device repackaging and repair were 0.005 and 0.02 kg CO₂, respectively. Total estimated per‐case carbon footprint of SU and reusable devices was 2.40 and 0.53 kg CO₂, respectively, favouring reusable devices. Impact of reusable scopes estimated to be considerably less than SU scopes at all calculated case volumes.	NA
Wombwell (2023), Urology; Inventory analysis (Wombwell et al. 2023)	C1: SU Ambu aScope 4 Cysto System (Ambu) versus C2: Reusable Olympus CYF‐VH flexible video‐cystoscope	***Favours C2 (Single‐Use):*** Although basic manufacturing carbon footprint cost/use between Ambu (reuseable) and Olympus (SU) cystoscopes vastly different (1.18 vs. 0.02 kg CO₂), once cleaning of reusable cystoscope considered, carbon footprint of SU cystoscope is ultimately lower than the reusable cystoscope (1.43 vs. 2.22 kg CO₂). SU cystoscopes have 36% lower carbon footprint, compared with their reusable counterpart.	NA
Davis (2018), Urology; Inventory analysis^d^ (Davis et al. 2018)	C1: Reuseable flexible ureteroscopes versus Comparator 2: Disposable flexible ureteroscopes	***No significant difference:*** Main finding – environmental costs of single‐use and reusable flexible ureteroscopes are comparable. Total carbon footprint of lifecycle of both flexible ureteroscopes was < 5 kg COZ/case. SU scopes: Manufacturing cost 11.49 kg CO₂, manufacturing carbon footprint 3.45 kg CO₂ per 1 kg ureteroscope. Sterilisation: 0.3 kg CO₂. Solid waste generated from disposal: 0.3 or 0.3 kg of CO₂. Total carbon footprint of LCA: 4.43 kg CO₂/endourologic case. Reuseable scope (1 kg): Manufacturing carbon footprint: 11.49 kg CO₂. Manufacturing costs/aScope: 0.06 kg CO₂ (i.e., 1 kg/180). Washing/sterilisation: 7.89 kg CO₂ for simultaneous washing and sterilisation of 2 ureteroscopes or 3.94 kg and 82.5 L of water per ureteroscope. Repackaging costs negligible. Solid waste: 0.06 kg CO_2_ (i.e., 11.49 kg CO_2_/180). Cost of repair: 5 kg CO_2_, 0.31 kg CO_2_ per case (5 kg CO_2_/16). Total carbon footprint of lifecycle: 4.47 kg CO_2_/case.	NA
Boberg (2022), GE; LCA (Boberg et al. 2022)	C1: SU trocar system versus C2: Reusable trocar system versus C3: Mixed trocar systems for laparoscopic cholecystectomies	***Favours C2 (Reuseable):*** SU trocar system's impact on climate change was 379% higher than RU system's impact and 12% higher than the mixed system's impact [median difference of 446 kg CO_2_eq (413–483) and 55 kg CO_2_eq (25–87), respectively. Similar environmental impact of the mixed and single‐use trocar systems could be explained by the higher plastic weight of the single‐use trocar used in the mixed system compared to the trocars used in the single‐use system. Differences regarding effects on climate change robust in sensitivity analyses.	***Comparison: SU versus RU*** – Resources, Ecosystem quality, Human health, **findings favour RU**. ***Comparison: SU versus Mixed*** – Resources, Ecosystem quality findings = **No significant difference**. Human health = **favours mixed**. ***Cost:*** RU and mixed trocar systems approx. half as expensive as SU.
Le (2022), GE; LCA (Le et al. 2022)	C1: Reuseable duodenoscope versus C2: Reuseable duodenoscopes with disposable endcaps versus C3: SU duodenoscopes	***Favours C1 (Reuseable):*** SU releases 36.3–71.5 kg CO_2_eq, which is 24–47 times > an RD (1.53 kg CO₂eq) or an RD with a disposable endcap (1.54 kg CO₂ equivalent). Most climate change impact of SDs comes from manufacturing = 91%–96% GHG emission. Second‐highest contributor is disposal of SD = 1.8 kg CO₂eq/procedure and accounts for 3%–5% GHG emission. RDs: top contributor to GHG emission: electricity use during procedure (62%), RD cleaning and disinfection (26%). RDs with disposable endcaps perform similarly to traditional RDs in all categories, with the advantage of potentially reducing infections.	***SU performs most poorly**:* Non‐renewable resource, Ecosystem quality, Human Health. ***No sig. difference C1 versus C2:*** Non‐renewable resource use, Ecosystem quality. ***Favours C2:*** Human health.
Rizan (2022), GE; LCA (Rizan and Bhutta 2022a)	C1: SU versus C2: Hybrid surgical instruments used for Laparoscopic cholecystectomy (laparoscopic clip appliers, laparoscopic scissors, and ports)	***Favours C2 (Hybrid instruments):*** Carbon footprint/operation of laparoscopic hybrid instrument versus SU equivalent was 17% for clip applier (445 vs. 255 g CO₂eq), 33% scissors (378 vs. 1139 g CO₂eq), and 27% for four ports (933 g CO₂eq vs. 3495 g CO₂eq/operation). When combined, carbon footprint of hybrid versions of all 3 instrument types 24% of SU equivalents (1756 g CO₂eq vs. 7194 g CO₂eq), saving 5.4 kg CO₂eq (normalised results: normal activities of global average person over 6 h). Hotspot analysis: majority carbon footprint of hybrid instruments due to SU components (mean 62%, range 43%–79%), followed by decontamination of reusable components (mean: 37%, range 21%–56%). For all hybrid instruments, carbon footprint lower than SU equivalents when reusable component used more than twice. Impact on carbon plateaued at around 10 uses of reusable components, with little additional gain (< 1%) after using laparoscopic scissors 60 times, ports 70 times, and clip appliers 100 times. However, continued use of these saves additional carbon burden obtaining new instruments. When packaged and decontaminated separately, carbon footprint of hybrid laparoscopic clip applier increased 3.7‐fold to 1650 g CO₂eq per use. There were small accompanying increases for laparoscopic scissors (to 394 g CO₂eq per use, 4% increase) and ports (999 g CO₂eq per use, 7% increase), due to greater proportional weight in instrument set. Nevertheless, in this alternative model, carbon footprint of all hybrid instruments lower than SU equivalents (36% less for laparoscopic clip appliers, 65% less for laparoscopic scissors, and 71% less for ports). Carbon footprint of decontamination process 54% higher when Australian electricity modelled, which increased carbon footprint of hybrid instruments by 11%–30% but this lower (63%–77%) than SU equivalents. Shipping instead of air‐freight for international transport of SU instruments reduced carbon footprint by 22%–33% relative to baseline SU items, but hybrid baseline instruments remained lower than shipped SU equivalents: by 74% the clip applier, 55% scissors, 65% ports. Using 3xhybrid 5 mm ports and 1 × 1 mm port (635 g CO₂eq/operation) resulted in 32% carbon footprint reduction relative to base scenario hybrid port setup. Use of SU ports with this alternative port configuration associated with sixfold increase in carbon footprint versus hybrid use (3613 g CO₂eq), constituting 3% increase relative to base scenario SU port setup. Under consequential approach to LCA, carbon footprint of hybrid laparoscopic clip applier was 198 g CO₂eq (7% of SU equivalent of 2559 g CO₂eq), scissors 299 g CO₂eq (26% of SU equivalent of 1139 g CO₂eq), and for four hybrid ports was 614 g CO₂eq (18% SU equivalent of 3495 g CO₂eq). When combined under the consequentialist approach carbon footprint of hybrid versions of all 3 instrument types for operation = 15% that SU equivalents (1110 g vs. 7194 g CO₂eq), saving 6083 g CO₂eq.	***20 impact categories, 15 favour hybrid:*** Stratospheric ozone depletion, ozone formation: human health, ozone formation: terrestrial ecosystems, fine particulate matter, mineral resource depletion, acidification, freshwater eutrophication, land use, fossil fuel scarcity, water consumption, human carcinogenic toxicity, human non‐carcinogenic toxicity. Endpoint: resources, Endpoint: Ecosystem quality, ***4 favour SU*** (2 based on incomplete data): Ionising radiation, freshwater ecotoxicity, marine water ecotoxicity, marine eutrophication. For human health endpoint category: combination of hybrid laparoscopic clip appliers, scissors, and ports for single laparoscopic cholecystectomy saved estimated 1.13 e^−5^ DALYs.
Sherman (2018), GE; LCA (Sherman et al. 2018)	C1: Reusable versus SU/disposable laryngoscopes	***Favours C1 (Reuseable):*** Life cycle emissions from reusables largely due to reprocessing and thus depend on the level of cleaning utilised. Overall, majority of life cycle emissions that SUD components generate created during initial material manufacturing and device assembly. Reusable laryngoscopes produce far fewer environmental emissions. Most favourable scenario: reusable stainless‐steel handle treated to HLD standards. LLD of reusable handle produces 40% more GHG emissions (0.08 kg CO₂eq per use) and STZ nearly 400% more (0.23 kg CO₂eq) than HLD (0.06 kg CO₂eq). SUD generates approx. 25× more GHG emissions (1.41 and 1.60 kg CO₂eq for the plastic and metal SUD handles, respectively) than reusable handle treated with HLD. Most favourable scenario across all emissions categories: reusable steel tongue blade treated to the minimum HLD standards. Like results for handles, sterilising reusable blades increases GHG emissions by nearly 400% (0.22 kg CO₂eq) compared to HLD (0.06 kg CO₂eq/use). SUD options for blades generate 6–8× as much GHG emissions/use as reusable HLD option depending on whether SUD blade is made of plastic (0.38 kg CO₂eq) or metal (0.44 kg CO₂eq). Even if treated with STZ, reusable device generates 40%–50% fewer GHG emissions than SUD alternatives. The SUD tongue blades were the overall worst option under all scenarios, and metal was worse than plastic. SU handles become environmentally preferable if reusable device lifetime falls below 5 and 4 uses for plastic and metal SUDs, respectively. SU plastic blades become environmentally preferable if multi‐use device lifetime falls below 5 uses, and for SUD metal blades its 3 uses of the reusable. The total recycling scenario demonstrated marginal reductions in GHG emissions over the standard waste disposal scenario for SUDs and had no significant impact on reusable device emissions.	***9 Impact categories – All favour RU:*** Ozone depletion, smog, fine particulate matter, ecotoxicity, marine water ecotoxicity, acidification, marine eutrophication, human carcinogenic toxicity, human non‐carcinogenic toxicity, ***Cost: Favours RU.***
Schulte (2021), Cardiac; LCA (Schulte et al. 2021)	Comparison 1: Newly manufactured catheter versus Comparison 2: Remanufactured catheter	***Favours C2 (Remanufactured):*** Using remanufactured medical catheter has lower impact on global warming (0.87 kg CO₂eq/catheter) than virgin production route (1.75 kg CO₂eq/catheter). Production/processing of plastics for producing virgin catheter is most contributing to the GWI of using a newly manufactured catheter for SU (59.4%). Carbon footprint of plastic production/processing for a newly manufactured catheter (1.04 kg CO₂eq/catheter) greater than GWI of entire medical remanufacturing process (0.87 kg CO₂eq/catheter). In medical remanufacturing route, electricity consumption contributes most to GWI (34.5%), followed by waste treatment (32.0%) and packaging materials (18.2%). GWI of treatment similar for medical remanufacturing (0.28 kg CO₂eq/catheter) and new production (0.30 kg CO₂eq/catheter) because loss rate of collected but not‐remanufactured catheters in medical remanufacturing process (47.9%). Approx. 2× catheters must be collected for each remanufactured catheter.	***15 impact categories. 12 favour RU:*** Ozone depletion, ionising radiation, ozone formation: Terrestrial ecosystems, freshwater ecotoxicity, acidification, marine eutrophication, eutrophication: terrestrial, human non‐carcinogenic toxicity, resource use: energy carriers, resource use: metals and minerals, respiratory inorganics. ***2 favour virgin**:* Freshwater eutrophication, land use. ***1 no difference:*** Waste consumption.
Sorensen (2018), Respiratory; LCA^e^ (Sørensen and Grüttner 2018)	C1: SU flexible device for bronchoscopy (the Ambu aScope 4 broncho) versus C2: Reuseable bronchoscope	***Favours C1 (Single‐Use) or No difference:*** Using one set of protective wear per operation and the materials for cleaning and disinfection determine that reusable scopes have higher emissions of CO₂eq. Cleaning two or more reusable scopes per set of PPE makes the impacts comparable. Other aspects that may impaAmbu aScope 4 broncho, gives credit of 6% energy when incinerated but adds extra 21% emission of CO₂‐equivalents. Numbers similar for RB. Consequence for regions where incineration with energy recovery is not available is CO₂‐equivalent emissions will be 21% lower for aScope. In the same way, the numbers can be interpreted for RBs. Recycling of packaging materials from the Ambu aScope 4 broncho gives 1% crediting for CO₂‐equivalent emissions. Due to assumption none of PPE or auxiliary materials used for cleaning of RBs is recycled, there will be no crediting to consider. Result of the assessment highly depends on use of PPE and cleaning procedures.	***2 impact categories – both favour SU**:* Loss of scarce resources, resource use: energy carriers.
Leiden (2020), Orthopaedics and/or trauma; LCA (Leiden et al. 2020)	C1: Reuseable versus C2: Disposable surgery instrument set for spinal fusion surgery	***Favours C2 (Disposable):*** Application of disposable set of instruments = environmental advantage of approx. 45%–85% against reusable set in all impact categories. Main environmental impact of disposable set generated in production phase‐this share always higher compared to reusable set. Major environmental impacts result from sterilisation of reusable set, mainly due to energy use for washing and steam sterilisation. Transportation and disposal processes have minor impacts in both cases. Sensitivity analysis results: increasing no. of surgeries/year, has negligible effect on entire environmental impact. But changing logistics principle (from loaner to consignment system) and consequently dividing no. of sterilisation cycles in halves = serious reduction of environmental impacts. External Co gamma sterilisation further reduces environmental impact, but environmental impact still higher than for disposable set. Further required transport increases environmental impact and impact for washing and disinfection within hospital remains same.	Aggregated single‐score indicator depicts overall benefit of 75% for SU.
Stripple (2008), Urology; LCA (Stripple et al. 2008)	C1: TPU catheter versus C2: PVC catheter versus C3: Polyolefin‐based elastomer catheter	***Favours C3 (Polyolefin‐based catheter):*** Fossil CO₂ emissions: TPU has highest CO₂ emissions (47.6 kg CO₂) and new polyolefin‐based elastomer the lowest emissions (35.8 kg CO₂). PVC catheter = 40.3 kg CO₂. Regarding total energy use or CO₂, NO_*x*_ or SO_2_ emissions, polyolefin‐based elastomer catheter shows lowest environmental impact, followed by PVC catheter and the TPU catheter having the highest environmental impact.	***Eco‐indicator 99 model – summary findings:*** Compared to TPU, new polyolefin‐based elastomer shows lower environmental impact in all categories except ecotoxic emissions and extraction of minerals. Compared to PVC, polyolefin‐based elastomer shows a lower impact in six of nine categories. ***CM2 model – summary findings**:* New material shows an overall low environmental impact. Compared to TPU, polyolefin‐based elastomer has a lower or equivalent environmental impact in all impact categories. Compared to PVC, its impact is lower in 5 out of 10 impact categories. ***EPS 2000 model – summary findings:*** Results show highest environmental impact for TPU catheter, while the polyolefin‐based elastomer and the PVC catheters show almost equivalent environmental impact, with a small favour towards the PVC catheter. However, these final scores based on weighted values.

*Note:* Green shaded cell: Study appraised as Low risk of bias; Orange shaded cell: Study appraised as High risk of bias; Blue shaded cell: Study appraised as Medium risk of bias.

Abbreviations: BP, blood pressure; C, comparator; CO₂, carbon dioxide; DALY, disability adjusted life year; GHG, greenhouse gases; GWI, global warming impact; HLD, high‐level disinfection; IQR, interquartile range; LLD, low‐level disinfection; NOX, nitrogen oxide; PPE, personal protective equipment; PVC, polyvinyl chloride; RB, reusable broncoscope; RU, reuseable; SU, single use; SUD, single use disposable; TPU, themoplastic polyurethane.

^a^ Incorporates simplistic LCA methods.

^b^ Results queried by Rizan and Bhutta (2022b) – see Discussion of this report.

^c^ Davis et al. (2018).

^d^ Stated as LCA but incomplete impact assessment.

^e^ Using one set of protective wear/operation and materials for cleaning and disinfection determine reusable scopes have higher values of resource consumption. Cleaning two or more reusable scopes per set of PPE makes the impacts fairly comparable.

###### 5.5.3.1.1 Reuseable Equipment

Twelve studies appraised as predominantly High or Medium risk of bias used LCA or inventory analysis methods to explore carbon emissions associated with reuseable equipment. Five were conducted within urology (Baboudjian et al. 2022; Davis et al. 2018; Hogan et al. 2022; Kemble et al. 2023; Wombwell et al. 2023), four within gastroenterology (Boberg et al. 2022; Le et al. 2022; Rizan and Bhutta 2022a; Sherman et al. 2018), one within cardiology (Schulte et al. 2021), one within respiratory (Sørensen and Grüttner 2018), and one within orthopaedics and/or trauma (Leiden et al. 2020).

Overall, reduced carbon emissions were associated with the use of reuseable equipment when compared to single‐use within the four studies conducted in the gastroenterology speciality. One study compared the impact of the climate of a single‐use trocar system with a reuseable and or trocar systems for laparoscopic cholecystectomies (446 kg CO₂eq (413–483) and 55 kg CO₂eq (25–87), respectively) (Boberg et al. 2022). The second study compared carbon emissions associated with reuseable duodenoscopes with those which were reuseable but had disposable endcaps and those which were single‐use (1.53 kg CO₂eq vs. 1.54 kg CO₂eq vs. 36.3 kg CO₂eq) (Le et al. 2022). The third study reported that the carbon footprint for hybrid laparoscopic cholecystectomy surgical instruments was 24% of single use equivalents (saving 5.4 kg CO₂eq based on normal activities of global average person over 6 h) (Rizan and Bhutta 2022b). A single study conducted in the cardiac setting also reported reduced carbon emissions associated with remanufactured versus newly manufactured catheters (0.87 kg CO₂eq/catheter vs. 1.75 kg CO₂eq/catheter) (Schulte et al. 2021).

However, findings across studies were inconsistent for equipment used within urology settings. One study calculating carbon emissions associated with ureteroscopes indicates that the environmental costs between single and reuseable devices were comparable, with total carbon footprint of life cycle for reuseable versus disposable scopes reported as 4.47 kg CO₂/case and 4.43 kg CO₂/case, respectively (Davis et al. 2018). Three studies calculating carbon emissions associated with cystoscopes indicated single‐use equipment was associated with reduced carbon emissions when compared to reuseable (Baboudjian et al. 2022; Hogan et al. 2022; Wombwell et al. 2023). Two studies reported 33% (Baboudjian et al. 2022), and 36% lower carbon emissions (0.02 kg CO₂ vs. 1.18 kg CO₂) for single use equipment compared to reuseable (Wombwell et al. 2023). The other study reported the total median carbon footprint was significantly higher at 4.23 kg CO₂ (IQR: 4.22–4.24) per case for reusable flexible cystoscope (*p* < 0.0001) (Hogan et al. 2022). In contrast, another study indicated that reuseable cystoscopes were associated with reduced carbon emissions compared to single‐use devices (0.53 vs. 2.40 kg CO₂) (Kemble et al. 2023). Reduced carbon emissions associated with single‐use equipment were also highlighted in another two studies, the first indicated that reuseable bronchoscopes were associated with higher CO₂eq emissions where only one set of protective wear/operation was used, although cleaning two or more reuseable scopes per PPE set meant CO₂‐eq emissions were comparable with disposable scopes (Sørensen and Grüttner 2018). The second study reported that disposable surgical instrument sets within spinal fusion surgery had a 45%–85% environmental advantage over reuseable sets (Leiden et al. 2020). Authors of the former study were funded by a manufacturer of a single‐use bronchoscope (Sørensen and Grüttner 2018).

Queries have been raised by experts in LCA methods regarding the methods used in four of the above studies to calculate carbon emissions associated with reuseable equipment (Baboudjian et al. 2022; Hogan et al. 2022; Leiden et al. 2020; Sørensen and Grüttner 2018). Specific concerns relate to a lack of clarity regarding, or inappropriate, selection of characterisation factors, unequal comparisons between the quantities of materials in reuseable versus disposable groups and overestimation of carbon emissions associated with reprocessing of reuseable equipment (Rizan and Bhutta 2022a). Variations in carbon‐emission findings associated with different equipment types may be greatly impacted by the assumptions made regarding the composition of equipment, the electricity mix and variations in how reprocessing of reuseable equipment is conducted across different sites (Hogan et al. 2022; Leiden et al. 2020). The carbon emissions associated with the systems required to support these processes may not always be appropriately factored into LCA methodology. Thus, the current evidence base, particularly within urology, makes it difficult to determine whether reuseable or single‐use equipment is associated with reduced carbon emissions.

Findings from other impact categories reflected the direction of carbon emission findings reported in individual studies. In general, studies reporting in favour of reuseable equipment in terms of carbon emissions noted reduced impact (or little difference) in the majority of other environmental impact categories; and vice versa for studies reporting reduced carbon emissions associated with disposable equipment.

Two studies evaluated the impact of reuseable versus disposable equipment on costs within gastroenterology settings, both concluding that reuseable or hybrid equipment costs less than disposable (Boberg et al. 2022; Sherman et al. 2018).

###### 5.5.3.1.2 Equipment Composition

One LCA appraised as Medium risk of bias evaluated carbon emissions associated with catheters composed of three different types of plastic (TPU vs. PVC vs. Polyolefin‐based elastomer), with the lowest carbon emissions associated with the polyolefin‐based catheter (47.6 kg CO₂ vs. 40.3 kg CO₂ vs. 35.8 kg CO₂) (Stripple et al. 2008). Across other environmental impact categories, the polyolefin‐based catheter had lower environmental impacts compared to TPU and approximately equivalent impact compared to PVC, depending on the model used.

##### 5.5.3.2 Non‐LCA Studies

###### 5.5.3.2.1 Equipment Type

Four studies, representing three speciality groups (orthopaedic and trauma [Chan 2023], multiple [Field et al. 2023], and ophthalmology [Moussa et al. 2022, 2021]) evaluated carbon emissions associated with different types of equipment. Carbon emission savings were associated with pulelavage equipment used during joint replacement surgery which required less raw material to make (assuming 95% of cases eligible: saving of 4501.1 kg CO₂eq) (Chan 2023), low‐volume anaesthesia machines versus traditional machines (402.26 metric tons of CO₂/year, with 20 operating rooms each performing 5.5 cases/day) (Field et al. 2023), air versus tamponade gas (reduction of CO₂ emissions by 44.3%–56.6%, saving up to 716.5 tons CO₂ annually, assuming 30% retinal detachments suitable for air instead of gas tamponade) (Moussa et al. 2021), and 30 mL gas cannisters versus gas cylinders (gas cylinders 40 times higher emissions than cannisters; CO₂ emission ranged from a mean equivalent of 3.17 kg/patient using 30 ml canisters to 124.8 kg using cylinders metric tons) (Moussa et al. 2022).

Due to the heterogeneity between specialities and intervention types, no meaningful comparison can be made across the studies in this category. Please see Appendix 9, Product level – additional tables, for a description of these studies, carbon emission findings and other outcomes measured.

#### 5.5.4 Care Delivery

**Summary: Care Delivery**

Sixteen studies evaluated interventions which changed some aspect of how condition‐specific treatment or care was delivered. Six studies focused on changes to treatment modalities or regimens (Chan 2023; Connor, Mortimer, et al. 2011; Coombs et al. 2016; de Preux and Rizmie 2018; Frick et al. 2023; Langstaff 2023; Vaidya et al. 2022), six evaluated alterations to the treatment/clinical pathway (Burton 2022; Cooper 2022; Cooper et al. 2023; Leapman et al. 2023; Nielsen 2022; Phull et al. 2023; Zander et al. 2011), and four studies evaluated changes to the surgical equipment or approach used (Fuschi et al. 2023; Meiklejohn et al. 2023; Thiel et al. 2015; Woods et al. 2015). Specialities represented included oncology/radiation oncology (*n* = 5) (Coombs et al. 2016; Frick et al. 2023; Langstaff 2023; Vaidya et al. 2022; Woods et al. 2015), renal (*n* = 2) (Chen et al. 2017; Connor, Lillywhite, et al. 2011), urology (*n* = 1) (Fuschi et al. 2023), orthopaedic and/or trauma (*n* = 1) (Kodumuri et al. 2023), ENT (*n* = 1) (Meiklejohn et al. 2023), and gynaecology (*n* = 1) (Thiel et al. 2015). Five studies were informed by LCA methods, and are described further below (Connor, Lillywhite, et al. 2011; de Preux and Rizmie 2018; Fuschi et al. 2023; Leapman et al. 2023; Meiklejohn et al. 2023; Thiel et al. 2015).

**LCA studies (*n*** = **5):** One component analysis study appraised as Medium risk of bias reported that home haemodialysis HHD using standard machines, 3 nights a week for 7 h was most effective in terms of patient health benefits, carbon reductions and financial costs (Connor, Lillywhite, et al. 2011; de Preux and Rizmie 2018). This finding was supported by one controlled trial concluding that HHD was associated with reduced carbon emissions when compared to in‐centre haemodialysis (ICHD) (Chen et al. 2017). Due to the variation in types of intervention and speciality, no meaningful comparisons can be made across the remaining LCA studies.

**Non‐LCA studies (*n*** = **11):** Four studies evaluated the impact of altering the treatment regimen for patients undergoing cancer treatment (Coombs et al. 2016; Frick et al. 2023; Langstaff 2023; Vaidya et al. 2022). All studies indicated that reduced carbon emissions were associated with treatment schedules which reduced the number of times patients were required to travel to the hospital. Two studies considered patient clinical (Langstaff 2023; Vaidya et al. 2022), safety (Langstaff 2023), and/or accessibility outcomes (Vaidya et al. 2022), with one study considering service costs (Vaidya et al. 2022). These outcomes were presented using narrative or descriptive statistics and were all in favour of the intervention. One controlled trial evaluated the carbon emissions associated with ICHD versus HHD, concluding that the former was associated with reduced carbon emissions (Chen et al. 2017). Four studies indicated that interventions which changed the patient care pathway were associated with reduced carbon emissions within orthopaedics and/or trauma (Cooper 2022; Cooper et al. 2023), cardiology (Nielsen 2022), urology (Phull et al. 2023), and ENT specialities (Burton 2022). Carbon emission calculations were mainly based on the materials consumed as a result of providing care and/or travel. Changes to the care pathway for patients needing urgent cardiac treatment, which required care to be provided in more specialist centres, were associated with higher carbon emissions (Zander et al. 2011). Three studies indicated that care pathway interventions were associated with reduced service costs (Burton 2022; Cooper 2022; Cooper et al. 2023; Nielsen 2022). Patient outcomes were poorly reported. One retrospective database review study comparing surgical approaches to staging procedure for endometrial cancer found that robot‐assisted laparoscopy had the highest carbon footprint, with laparotomy the lowest (Woods et al. 2015). Laparotomy was also found to have the lowest energy consumption and was associated with the lowest environmental energy use (Woods et al. 2015).

##### 5.5.4.1 LCAs

###### 5.5.4.1.1 Treatment Pathway

One LCA appraised as Medium risk of bias was conducted within urology (Leapman et al. 2023), and evaluated an intervention examining how different combinations of prostate magnetic resonance imaging (MRI) and transrectal ultrasound guided prostate biopsy sampling could affect carbon emissions, indicated that systematic biopsy without the use of MRI produced the lowest carbon emissions of the four comparators being evaluated (bi‐parametric prostrate MRI with targeted/systematic biopsies: 70.5 kg CO₂eq, mpMRI with targeted biopsies cores only: 6.2 kg CO₂eq, systematic biopsy without MRI: 36.2 kg CO₂eq, mpMRI with systematic biopsy: 78.9 kg CO₂eq) (Leapman et al. 2023). No other outcomes were evaluated within this study.

###### 5.5.4.1.2 Treatment Regimen

One component analysis study appraised as Medium risk of bias evaluated the environmental impact and cost‐effectiveness of different haemodialysis regimens and place of delivery (Connor, Lillywhite, et al. 2011; de Preux and Rizmie 2018). In this study, the authors highlighted the tension between reducing carbon emissions through the provision of HHD by reducing patient travel and increasing carbon emissions through increasing the frequency and number of HHD treatments. They indicated that HHD using standard machines, 3 nights a week for 7 h was most effective in terms of patient health benefits, carbon reductions and financial costs. The carbon footprint associated with different treatment delivery regimens was as follows:

- ICHD – 3 days/week, 5 h/session: 3.8‐ton CO₂eq/patient/year.
- HHD – 4 days/week, 4 and a half hours/session: 4.3‐ton CO₂eq.
- HHD using standard equipment, 5 days/week, 4 h/session: 5.1‐ton CO₂eq.
- HHD using standard equipment, 6 days/week, 2 h/session: 5.2‐ton CO₂eq.
- HHD using standard equipment, 6 nights/week, 7 h/session: 7.2‐ton CO₂eq.
- HHD using standard equipment, 3 nights/week, 7 h/session: 3.9‐ton CO₂eq.
- HHD using N × Stage equipment, 5 and a half days/week, 3 h/session: 1.8‐ton CO₂eq.
- HHD using N × Stage equipment, 6 nights/week, 7 h/session: 2131 kg CO₂eq.

###### 5.5.4.1.3 Surgical Procedure

One inventory analysis appraised as Medium risk of bias (Fuschi et al. 2023), and two studies using LCA methods (critically appraised as being of Low [Thiel et al. 2015], and Medium risk of bias [Meiklejohn et al. 2023]) evaluated the impact of altering the types of surgical procedures used on environmental outcomes. These interventions included comparing robotic and laparoscopic surgical techniques for radical prostatectomy (carbon emissions higher for laparoscopic procedure at 12,946.73 g than robot assisted procedure at 9506.18 g) (Fuschi et al. 2023), different methods for conducting tonsillectomy (absolute values GHG emissions for cold, monopolar electrocautery, and coblation: 157.6, 184.5, and 204.7 kg CO₂eq per surgery, respectively (Meiklejohn et al. 2023), and different methods for undertaking hysterectomies (robotic hysterectomy largest impact on GHG emissions, with the upper range of the 90% confidence interval for GHG associated with a laparoscopic approach overlapping average GHG impact of robotic approach. Without anaesthetics, abdominal and vaginal hysterectomies emit significantly lower GHG (Thiel et al. 2015). Due to the variation in types of intervention and speciality, no meaningful comparisons could be made across these studies. Instead, the findings from the individual studies are summarised below in Table 6, with further detail in Appendix 10, Care delivery – additional tables.

**Table 6:** Care delivery – Summary of findings from LCA studies.

Study, speciality: Design (Comparators)	Carbon emission findings (based on summary of reported findings for each study)	Summary of other impacts											
		Ozone depletion	Smog	Respiratory/Fine particulate matter	Ecotoxicity	Acidification	Eutrophication	Human carcinogenic toxicity	Human non‐carcinogenic toxicity	Reuseable equipment	Operating time	Energy demand	Cost
Connor (2011a)^e^; de Preux (2018)^e^, Renal: CA (Home vs. In hospital maintenance HD – Diff. treatment regimens (Place of treatment/Machine type/freq. treatments/duration treatments (h)): C1: ICHD Standard 3 days/week, 4 h versus C2: HHD Standard 4 days/week, 4.5 h versus C3: HHD Standard 5 days/week, 4 h versus C4: HHD Standard 6 days/week, 2 h versus C5: HHD Standard 6 nights/week, 7 h versus C6: HHD Standard 3 nights/week, 7 h versus C7: HHD N × Stage 5.5/week, 3 h versus C8: HHD N × Stage 6 nights/week, 7 h) (Connor, Lillywhite, et al. 2011; de Preux and Rizmie 2018)	Most common form of dialysis in the United Kingdom (3× week ICHD, 7) = carbon footprint of 3818 kg CO₂eq/patient/year, with majority of emissions arising within medical equipment (37%), building energy use (21%) and patient travel (20%). Delivery of HHD using standard HD machines = release of 3901–7197 kg CO₂eq/pt/year depending on regime. Regime choice may have twofold impact on carbon footprint. Following reduction in patient travel emissions, clinically beneficial increase in dialysis treatment times (beyond 3× week ICHD) achieved without associated increase in overall carbon footprint through provision of HHD. 3× week nocturnal HHD offers 9hrs more dialysis/week than ICHD, with comparable carbon footprints (3901 and 3818 kg CO₂eq, respectively). Provision of 6x2hr HHD treatments/week = 5210 kg CO₂eq— 43,818 kg attributable to delivery of same total weekly treatment time by 3× week ICHD. Emissions from medical equipment supply chains = 37% of the emissions associated with ICHD. Re‐use of dialyzers over 10 treatments reduces carbon footprint of 3× week ICHD by 9.7%, from 3818 to 3448 kg CO₂eq/pt/year. Substantial carbon saving derives from reductions in supply chain emissions of the dialyzers (290 kg CO₂eq) and associated packaging (4 kg CO₂eq), and from reductions in waste management emissions (primarily reduction of kg CO₂eq in incineration emissions). Electricity consumption contributes significantly to carbon footprint of provision of HD using standard machines, representing 21% and 48% of emissions associated with ICHD and six nightly nocturnal HD, respectively. Newer HD technologies may offer solution. Provision of 3 h treatments/5.5 days/week using N × Stage equipment = carbon footprint of 1844 kg CO₂eq—<half that of 3× week ICHD. Six nightly nocturnal HHD using N × Stage equipment results in 2131 kg CO₂eq—< 1/3 emissions of comparable HHD regime using standard HD machine. Emissions attributable to patient undertaking 5.5 weekly dialysis but never travelling (1841 kg CO₂eq) almost identical to those of a patient undertaking 5.5× week dialysis and travelling in line with the assumptions made in study (1844 kg CO₂eq). Summary: As a result of reduction in patient travel emissions, clinically beneficial increase in dialysis treatment times (beyond that of 3× week ICHD) can be achieved without associated increase in carbon footprint through provision of HHD. 3× week nocturnal HHD offers 9 h more dialysis/week than ICHD, yet 2 regimes have comparable carbon footprints (3901 and 3818 kg CO₂eq, respectively). However, production of medical equipment is carbon intensive, and reduction in patient travel emissions is soon offset by increase in the frequency of HD treatments – provision of 6 × 2‐h HHD treatments/week = carbon footprint of 5210 kg CO₂eq—considerably 43,818 kg attributable to the delivery of the same total weekly treatment time by 3× week ICHD. Authors suggest rising uptake of HHD, in current form and using standard machines, likely to increase, rather than decrease, carbon footprint of HD programmes.												C6^f^
Fuschi (2023), Urology; Inventory analysis (Fuschi et al. 2023)	***Favours C2 (Robot assisted):*** CO₂ emissions resulting from production, disposal, and sterilisation of instruments overall higher for the instruments used in laparoscopic procedure (12,946.73 g) compared to robot‐assisted procedure (9506.18 g), with majority of the emissions coming from plastic (9083.30 g vs. 6481.80 g) and from composite fibre components (3019.63 g vs. 2157.63 g), the robot‐assisted procedure had higher emissions from metal components (866.76 g vs. 839.80 g). Total CO₂ emissions from energy consumption = 37,807.23 g for robotic procedure and 46,728.24 g for laparoscopic procedure. Total CO₂ emissions for robot‐assisted procedure = 47,313.414 g per procedure, of which 9506.18 g derived from instrument production, disposal, and sterilisation; whereas 37,807.23 g from energy consumption. Total laparoscopic CO₂ emissions = 59,674.96 g, with 12,946.72 g being derived from instrument use and 46,728.24 from energy consumption. Significant differences with lower CO₂ production obtained with robotic approach than laparoscopic, considering total CO₂ emissions, CO₂ derived from production, disposal and sterilisation, and energy consumption.												
Leapman (2023), Urology: LCAd (Different treatment pathways: C1; Bi‐parametric prostate MRI with targeted and systematic biopsies vs. C2: mpMRI with targeted biopsy cores only vs. C3: Systematic biopsy without MRI vs. C4: mpMRI with systematic biopsy)(Leapman et al. 2023)	***Favoured C3 (Systematic biopsy without MRI):*** (C1) bpMRI with targeted and systematic biopsies would result in 70.5 kg CO₂eq, a 10.7% reduction relative to mpMRI. Variation in emissions by biopsy strategy. (C3) A strategy of a 12‐core systematic biopsy without prostate MRI generated fewest emissions (36.2 kg CO₂eq), majority of which (33.0 kg CO₂eq, 91.3%) contributed by biopsy procedure itself and 3.2 kg CO₂eq (8.7%) from pathology analysis. (C2) Incorporation of prostate MRI increased estimated CO₂eq, primarily due to MRI step, and smaller contributions from additional biopsy core acquisition and processing. MRI with systematic biopsy sampling resulted in 78.9 kg CO₂eq, while approach of obtaining 2–5 MRI‐fusion cores alone without systematic biopsy = 6.2 kg CO₂eq.												
Meiklejohn (2023), ENT: LCA (C1: ME vs. C2: Coblation vs. C3: Cold excision without cautery) (Meiklejohn et al. 2023)	***No sig. difference between comparators:*** Life cycle impacts: Absolute values for GHG emissions for cold, ME, and coblation were 157.6, 184.5, and 204.7 kg CO₂eq per surgery, respectively. No statistically significant differences between techniques (all processes within the system boundaries included). Medications used for anaesthesia contributed most to GHG emissions, regardless of surgery technique. Subgroup analysis of the disposable items that differed between technique demonstrated a statistically significant difference in the GHG emissions attributable to disposable surgical items among the three different tonsillectomy techniques (*χ* ^2^ð^2^) = 9.4168, *p* = 0.009). A post hoc pairwise comparison based on the Wilcoxon rank sum test revealed that a statistically significant difference (*p* < 0.05) was observed between ME and cold technique, and between coblation and cold technique, with cold having reduced impact in both comparisons.	C3^a^		C3^a^^,^ b	><^a^	C3^a^	C3^a^	C3^a^	C3^a^	><	><		C3 [N]
Thiel (2015), Gyn: LCA (Type of hysterectomy – C1: Abdominal vs. C2: Vaginal vs. C3: Laparoscopic vs. C4: Robotic) (Thiel et al. 2015)	***Favours C1 and C2 (Abdominal/Vaginal)*** c: Robotic hysterectomy largest environmental footprint over other hysterectomy types in every impact category analysed. Upper range of laparoscopic hysterectomy's 90% confidence interval overlaps with average impacts robotic hysterectomies in every category. Error bars in GHG emissions largely influenced by anaesthetic choice, which varies based on anaesthesiologist preference and is not indicative of type of hysterectomy performed. Without anaesthetics, abdominal and vaginal hysterectomies emit significantly less greenhouse gases, with narrower confidence intervals, than laparoscopic and robotic hysterectomies. On average, anaesthetic gases contributed to a third of the greenhouse gas emissions of robotic and laparoscopic hysterectomies and two‐thirds of abdominal and vaginal hysterectomies. For abdominal and vaginal hysterectomy, anaesthetic use contributed to 98% of the ozone depletion potential. GHG emissions for vaginal hysterectomy from anaesthetics varied drastically between cases, from 0.001 kg CO₂eq/case to 505 kg CO₂eq/case.	C2 + C3	C1 HI^d^	C2 + C3	C1 HI^d^	C2 + C3	C1 HI^d^	C1 HI^d^	C1 HI^d^	><		C2 + C3^e^	

*Note:* Blue shaded cell: Appraised Medium risk of bias; Orange shaded cell: Study appraised as High risk of bias; Grey shaded cell: No data.

Abbreviations: C, comparator; CO_2_, carbon dioxide; CO_2_eq, carbon dioxide equivalent; GHG, greenhouse gas; HD, haemodialysis; HHD, home haemodialysis; HI, highest impact; h, hour; ICHD, in‐centre haemodialysis; ME, monopolar electrocautery; MRI, magenetic resonance imaging; N, number;

^a^ In relation to disposable instruments.

^b^ No significant difference observed between ME and Coblation technique for any impact category.

^c^ Without anaesthetics.

^d^ Significant overlap with laparoscopic, abdominal, and vaginal.

e Component analysis.

f Most effective (health benefits + carbon/financial costs).

##### 5.5.4.2 Non‐LCA

###### 5.5.4.2.1 Treatment Regimen

Studies using non‐LCA methodology explored the impact of different treatment regimens/schedules on carbon emissions within an oncology/radiation oncology (*n* = 4) (Coombs et al. 2016; Frick et al. 2023; Langstaff 2023; Vaidya et al. 2022), or renal setting (*n* = 1) (Chen et al. 2017).

Four studies evaluated the impact of altering the treatment regimen for patients undergoing cancer treatment (Coombs et al. 2016; Frick et al. 2023; Langstaff 2023; Vaidya et al. 2022). Study designs include an RCT (Coombs et al. 2016), a controlled trial (Frick et al. 2023), a before‐and‐after trial (Langstaff 2023), and a retrospective cohort (Vaidya et al. 2022). All studies indicated that the reduced carbon emissions were associated with treatment schedules which reduced the number of times patients were required to travel to the hospital. Patients treated with single‐dose intraoperative radiotherapy had lower CO₂ emissions than those receiving several weeks of external beam whole breast radiotherapy (24.7 kg [SE 5.4] vs. 111 kg [SE 8.6]) (Coombs et al. 2016). Total CO₂eq emissions for long‐ and short‐course radiation therapy were 665.3 and 149.9 kg CO₂eq per patient treatment course, respectively (*p* < 0.001), with a net difference of 515.4 kg CO₂eq (Frick et al. 2023). Administering photo biomodulation (PBM) to prevent/reduce oral mucositis in patients receiving radiotherapy over 30‐days, resulting in a total carbon saving of 2613.99 kg CO₂eq per year for 11 patients (42,774 kg CO₂eq per year among 180 patients) (Langstaff et al. 2023). By avoiding additional travel associated with external beam radiotherapy through receiving targeted intraoperative radiotherapy, one study estimated a carbon footprint reduction of 5.6 million kg CO₂ emissions (Vaidya et al. 2022).

Other outcomes measured in these studies, which reflect this finding, included savings to patient travel time (*n* = 2) (Coombs et al. 2016; Frick et al. 2023), distance (*n* = 3) (Coombs et al. 2016; Frick et al. 2023; Vaidya et al. 2022), or costs (*n* = 1) (Frick et al. 2023). Two studies considered patient clinical (Langstaff 2023; Vaidya et al. 2022), safety (Langstaff 2023), and/or accessibility outcomes (Vaidya et al. 2022), with one study considering service costs (Vaidya et al. 2022). These outcomes were presented using narrative or descriptive statistics, and most were in favour of the intervention.

One controlled trial evaluated the carbon emissions associated with ICHD versus HHD, concluding that the former was associated with reduced carbon emissions (Chen et al. 2017).

###### 5.5.4.2.2 Treatment Pathway

Five non‐LCA studies evaluated the effectiveness of changes to treatment/clinical pathways in reducing carbon emissions, one controlled trial was conducted within orthopaedics and/or trauma (Cooper 2022; Cooper et al. 2023), one before and after (Nielsen 2022), and one modelling study within cardiology (Zander et al. 2011), and one study each in urology (Phull et al. 2023), and ENT (Burton 2022), utilising retrospective database review and modelling study designs, respectively. Details regarding the care pathways evaluated can be found in Appendix 10, Care delivery – additional tables.

Care pathway interventions were associated with reductions in carbon emissions across three studies, and were mainly attributed to a reduced number of face‐to‐face visits. This followed introduction of a digital day‐case pathway for knee replacement (reduction of 119,381 kg CO₂ compared to face‐to‐face visits) (Cooper 2022), reduced hospital length of stay (LOS) following implementation of an early mobilisation programme (48.5 tonnes CO₂eq) (Nielsen 2022), or reduced patient travel after implementation of a new local anaesthetic pathway for patients with nose fracture (4137.26 kg CO₂eq/year) (Burton 2022). However, where changes to the care pathway required patients to travel via ambulance to specialist care centres, carbon emissions increased from 3.46 kg to 11.2 kg (Zander et al. 2011).

Carbon emission calculations were mainly based on the materials consumed as a result of providing care and/or travel (Cooper 2022; Cooper et al. 2023; Zander et al. 2011), with two studies considering energy consumption involved in delivering care (Burton 2022; Phull et al. 2023), and/or waste disposal (Phull et al. 2023). No studies considered carbon emissions associated with extraction/product manufacture or material transport. Comparisons between intervention and control groups for outcomes relevant to service use were mainly based on descriptive/narrative analysis and included the number of face‐to‐face visits (*n* = 1) (Cooper 2022), hospital LOS (*n* = 3) (Cooper et al. 2023; Nielsen 2022; Phull et al. 2023), and the number of physiotherapist appointments (*n* = 1) (Cooper et al. 2023). Outcomes favoured care pathway intervention over standard care pathways.

Three studies indicated that care pathway interventions were associated with reduced service costs (Burton 2022; Cooper et al. 2023; Nielsen 2022). Only one study, which evaluated the impact of a day‐case pathway versus inpatient care for patients undergoing transurethral bladder tumour surgery, evaluated any patient‐focused outcomes, with analysis based on descriptive/narrative statistics indicating that the day‐case pathway reduced the number of patient readmissions (Phull et al. 2023).

###### 5.5.4.2.3 Surgical Procedure

One retrospective database review study compared different surgical procedures for patients undergoing a staging procedure for endometrial cancer (robotically assisted laparoscopy, laparoscopy and laparotomy) (Woods et al. 2015). Robot‐assisted laparoscopy was found to have the highest carbon footprint (40.3 kg CO₂eq/patient), and laparotomy the lowest (22.7 kg CO₂eq/patient). Laparotomy was also found to have the lowest energy consumption and was associated with the lowest environmental energy use (Woods et al. 2015).

#### 5.5.5 Multiple Components

Seven studies evaluated interventions that included multiple components, representing two or more of the other four categories described above. Specialities represented included renal (*n* = 2) (Bendine et al. 2020; Hardy 2022), gastroenterology (*n* = 1) (Materacki 2023), oncology/radiation oncology (*n* = 1) (Cheung et al. 2023), radiology (*n* = 1) (Chuter et al. 2023), gynaecology (*n* = 1) (Thiel et al. 2018), and multiple (*n* = 1) (Rouviere et al. 2022). Three studies drew on LCA methods; two inventory analyses were appraised as High risk of bias (Rouviere et al. 2022; Thiel et al. 2018), and one inventory analysis was appraised as Medium risk of bias (Chuter et al. 2023). Heterogeneity across types of speciality and intervention precluded meaningful synthesis. An overview of the study characteristics and main findings relating to carbon emissions and other outcomes is provided in Appendix 11, Multiple components – additional tables.

## 6 Discussion

### 6.1 Summary of Main Results

This systematic review aimed to examine the effectiveness of interventions in reducing the carbon footprint within medical specialities in secondary healthcare and summarise how this evidence could inform the patient care pathway. Eighty‐eight studies met the eligibility criteria. We presented the evidence in an EGM, structured according to a secondary healthcare patient care pathway. Urology (*n* = 14), gastroenterology (*n* = 12). oncology/radiation oncology (*n* = 13) and renal (*n* = 11) were the most common specialities represented. Across different specialities, the majority of evidence was found in the first three stages of the patient care pathway (Initial assessment/diagnostic tests, initial treatment or follow‐up). The exception to this was the renal speciality, where most of the evidence was within the ‘ongoing care’ segment of the patient care pathway. This may reflect the ongoing treatment required by individuals receiving haemodialysis and thus the associated opportunities to conserve energy and resources. There was limited evidence within the ‘discharge’ segment of the care pathway across all specialities. Evidence relating to the wider healthcare setting was clustered within the gastroenterology (*n* = 5) and radiology specialities (*n* = 5).

Twenty‐eight studies used LCA‐informed methodology. Nine LCA studies were appraised as Low risk of bias (Baboudjian et al. 2022; Boberg et al. 2022; Holmner et al. 2014; McAlister et al. 2022; Rizan and Bhutta 2022; Schulte et al. 2021; Sherman et al. 2018; Thiel et al. 2015, 2023), 14 as Medium risk of bias (Chuter et al. 2023; Connor, Lillywhite, et al. 2011; de Ridder et al. 2022; Fuschi et al. 2023; Kemble et al. 2023; Le et al. 2022; Leapman et al. 2023; Leiden et al. 2020; Lopez‐Munoz et al. 2023; Meiklejohn et al. 2023; Sillcox, Gitonga, et al. 2023; Sørensen and Grüttner 2018; Stripple et al. 2008; Wombwell et al. 2023), and 5 as High risk of bias using the criteria detailed within the methods section above (Davis et al. 2018; Hogan et al. 2022; Rouviere et al. 2022; Thiel et al. 2018; Winklmair et al. 2023). Three of the five studies appraised as High risk of bias were inventory analysis (Davis et al. 2018; Hogan et al. 2022; Winklmair et al. 2023). Study characteristics associated with a High risk of bias appraisal rating included the following issues regarding the goal and scope of the studies: poor definition of functional unit used, poor description and/or justification of system boundaries and life cycle stages included in the analysis, calculations did not consider production, use/reuse and disposal of materials and energy. In terms of the inventory analysis, High risk of bias scores were associated with poor description of impact categories, categorisation method and software used and poor reporting of results in the context of the functional unit. Regarding interpretation of findings, studies appraised as High risk of bias did not contextualise their findings through the use of uncertainty and/or sensitivity analysis.

Interventions evaluated by the included studies were classified into one of five broad categories: ‘Accessing care’ (*n* = 29), ‘Setting’ (*n* = 20), ‘Product level’ (*n* = 16), ‘Care delivery’ (*n* = 16) and ‘Multiple components’ (*n* = 7). The two largest groups of evidence were for studies evaluating telehealth (*n* = 26) and reuseable equipment (*n* = 13) interventions. Telehealth interventions were predominantly evaluated using non‐LCA methodology (*n* = 23) and while carbon emissions favoured telemedicine interventions when compared to face‐to‐face care, these calculations often only considered patient‐travel saved and did not account for carbon emissions associated with the use of the digital pathway or other parts of the patient care pathway, such as the impact on primary care. In general, the majority of patient and cost outcomes evaluated favoured the telemedicine intervention, although most outcomes were based on descriptive or narrative analyses. These findings were reflected in the systematic review by Ravindrane and Patel (2022), which explored the environmental impact of telemedicine instead of face‐to‐face care in healthcare. They highlighted that the benefit of telemedicine in terms of carbon emission reduction was dependent on energy consumption of the telemedicine systems, number of patients, mode of transport used, and distance of travel avoided and indicated that improvements to modelling used within studies were needed, including the use of sensitivity analysis and transparent reporting of assumptions used (Ravindrane and Patel 2022). Lange et al. (2022) also highlighted the poor methodological quality of carbon emission methodology used in their review of telemedicine interventions within healthcare (Lange et al. 2022).

Interventions comparing carbon emissions associated with the use of reuseable versus disposable surgical equipment represented the largest group of studies utilising LCA methods. For studies within the gastroenterology speciality, reuseable equipment was associated with reduced carbon emissions. Within urology, this finding was reversed, with disposable instruments found to be associated with reduced carbon emissions. However, despite the quality of these studies being appraised as mainly High or Medium within this review, questions regarding the accuracy of use of characterisation factors, quantity of materials used in disposable versus reuseable equipment packs and how carbon emissions were assigned to the reprocessing stage of reuseable equipment mean confidence in this finding is uncertain (Brighton and Sussex Medical School, Centre for Sustainable Healthcare, and UK Health Alliance on Climate Change 2023; Rizan and Bhutta 2022). The latter finding contrasts with findings from two other systematic reviews, which indicate reuseable devices are associated with improved environmental outcomes (Drew et al. 2021; Siu et al. 2017), although limitations to the evidence base include methodological heterogeneity and lack of background life cycle inventory data for surgical inputs (Drew et al. 2021), and lack of cost‐comparison studies (Siu et al. 2017). The uncertainty regarding the beneficial effects of reuseable equipment on carbon emissions within urology arising from this review underscores the importance of considering the full product pathway within an LCA approach and ensuring the system boundaries for the change being considered reflect all parts of the patient care pathway and product life‐cycle. The composition of products evaluated and processes associated with, for example, transport, reprocessing of reuseable devices and waste disposal are highly context dependent, with alterations to these processes potentially having a huge impact on estimated carbon emission calculations (Hogan and Hennessey 2023; Leiden et al. 2020). Thus, it can be challenging to generalise findings across LCA studies, even when conducted in similar countries/health systems for the same type of intervention, and emphasises the importance of incorporating sensitivity analysis into LCAs. It also highlights the importance of considering how to reduce carbon emissions associated with the processes supporting the manufacture, transport and reprocessing of disposable and/or reuseable equipment as a target for future interventions. This is an alternative focus to comparing emissions associated with disposable versus reuseable equipment and promotes addressing carbon emissions associated with known ‘hot spots’ in the lifecycle of both types of product, such as manufacturing for disposable products and reprocessing for reuseable products. This may complement recommendations from the Green Surgery and MedTech Circular Economy reports to, among other actions, pursue the use of reuseable equipment to reduce carbon emissions and overcome challenges within supply chains, resource scarcity, healthcare disparities and waste production (Brighton and Sussex Medical School, Centre for Sustainable Healthcare, and UK Health Alliance on Climate Change 2023).

Finally, while waste management/reduction interventions were associated with reduced carbon emissions (*n* = 12), interventions were highly heterogeneous with limited consideration of patient or cost outcomes. Eight non‐LCA studies found reduced carbon emissions were associated with energy conservation interventions, the majority of which were conducted within radiology/radiotherapy settings and focused on the impact (or potential impact) of turning machines off when not in use.

### 6.2 Strengths and Limitations of This Review

We have conducted a comprehensive systematic review of the literature, which identifies and synthesises comparative studies evaluating interventions to reduce carbon emissions across nine specialities within secondary healthcare. We grouped these studies by broad intervention category to enable identification of carbon emission, patient, and cost outcomes relevant to specific interventions within each speciality, separating out evidence from studies which used LCA methods to calculate carbon emissions to highlight findings supported by the most methodologically robust evidence base. Our synthesis was informed by the critical appraisal of these LCA studies. Due to a lack of standardised appraisal tools and associated risk of bias thresholds, the critical appraisal scores awarded by reviewers were calibrated based on the set of studies included in the review. Further work to establish the validity of these thresholds is required. Unfortunately, there was a high degree of heterogeneity between types of intervention conducted within individual specialities, which made it challenging to identify interventions which were effective in reducing carbon emissions within similar and across different contexts. While any intervention with a direct link to the specialities indicated was eligible for inclusion in this review, there may be other interventions, such as the frequency with which blood pressure cuffs are sanitised (Sanchez et al. 2020), which may be relevant across multiple specialities but have been excluded from this review, as this has not been clearly stated. Thus, the evidence in this review may not reflect the full range of interventions available to clinicians within a particular speciality.

The number of studies including patient and cost outcomes alongside carbon emission calculations was also limited. This may reflect our inclusion criteria, which required studies to measure carbon emissions. Thus, unless related to one of our included studies, studies purely focused on patient outcomes or service costs would have been excluded. The paucity of studies reporting patient clinical outcomes and satisfaction from both intervention and control groups may also reflect that studies using a before‐and‐after design or conducting a retrospective database review relied on data recorded on electronic databases, where these outcomes may not be routinely recorded. Our inclusion criteria also required that included studies referenced a particular speciality, which may have resulted in the exclusion of otherwise relevant interventions, particularly within the ‘systemic interventions’ section of the care pathway.

Within studies drawing on an LCA approach, the lack of transparency in the reporting of methodological details raised issues of comparability and generalisability. The variability among LCA studies may be explained by data collection and calculation procedures, along with the researchers' assumptions and choices of background inventory databases. Although the Ecoinvent database was most commonly reported, there was a wide variety of secondary sources used by researchers, many of which were originally compiled for other purposes, ranging from government documents to other research papers and conference proceedings. In some instances, researchers reported using manufacturer details to calculate raw material composition of devices (e.g., Hogan et al. 2022; Kemble et al. 2023; Rizan and Bhutta 2022a, 2022b), while others could not access such data and based their calculations on available data for similar devices (e.g., Le et al. 2022). In addition, comparability was hindered by the lack of consistency in how studies defined and reported the system boundaries for the individual LCA studies. The difficulty in generalising results from the LCA was that the data could be specific to a particular context or intervention. The geographical setting of a study was important, particularly in relation to calculating the electricity supply. Studies undertaken in the United States, for example, assumed a US electricity supply with sources derived largely from fossil fuel (e.g., Leapman et al. 2023), and results based on these assumptions were not likely to apply to other countries with cleaner energy sources such as Sweden (e.g., Holmner et al. 2014).

Carbon emission calculations used within non‐LCA studies were typically narrow in scope, focusing on the use and/or reuse of products, with less consideration of other factors within the wider system, which may also influence carbon emissions of the intervention, for example, energy used by both health services and patients. The extent to which carbon‐emission calculations in non‐LCA studies considered emissions associated with the manufacture of equipment, vehicles or fuel, transport and/or waste management was also limited and dependent on the intervention in question. These issues were particularly evident for interventions such as telemedicine or remote delivery of care, where carbon‐emission calculations were typically based on non‐statistical comparisons of patient travel distance saved because of reduced number of visits to hospital, with less consideration of factors such as staff and patient energy use via heating, lighting and/or internet access. For studies focusing on waste management/recycling initiatives as part of local initiatives to reduce carbon emissions within specific NHS trusts, carbon‐emission calculations rarely consider emissions associated with the transport and recycling of waste, which would otherwise have been destroyed. However, where comparable interventions existed between the two groups of evidence, findings from non‐LCA studies generally reflected those in LCA studies.

We have presented all included studies within an interactive EGM, which displays the evidence relative to the patient care pathway for each speciality. This will enable evidence users to locate evidence relevant to their interests and requirements, and highlight where groups of evidence and gaps exist. The smaller quantity of evidence relating to the ‘Discharge’ part of the patient care pathway is likely influenced by the inclusion criteria for this review, which focused on interventions led by secondary healthcare. Thus, interventions such as self‐management or ongoing support within the community would not have been captured in the EGM. In addition, most interventions included in this review, and thus within the patient care pathway in the EGM, often reflect tangible changes that clinicians can make during their everyday practices at key patient care pathway. While more complex interventions, which changed the structure of patient care pathways and multi‐component interventions, were represented within this map, they were in the minority. This may reflect the increased difficulty in implementing and evaluating these types of intervention. Thus, while some of the interventions included in this review may correspond with known carbon hotspots (e.g., those targeting use of resources within surgical settings), others may indicate situations where clinicians feel they can quickly implement a change to address an area of high resource/energy consumption within their local service context.

## 7 Authors' Conclusions

This systematic review synthesises quantitative evidence evaluating the effectiveness of interventions intended to reduce carbon emissions within high‐volume specialities delivered within secondary healthcare. It highlights a highly heterogeneous evidence base and the methodological limitations associated with studies based on LCA and non‐LCA methods. While we identified several large clusters of studies evaluating similar interventions within the same speciality, future research needs to address these methodological limitations to support confident decision‐making within policy commissioning and clinical practice. Our EGM displays the included evidence according to individual speciality along the patient pathway, enabling evidence users to identify research which meets their requirements, as well as identifying potential gaps where further research may be required.

### 7.1 Implications for Research

– Existing research relating to carbon emissions reflects a narrow range of all the possible interventions/specialities available. Further research is needed to fill the gaps highlighted in the EGM, particularly evidence relating to the ongoing care or discharge of patients or relating to obstetric and respiratory specialities.
– Evidence generated using LCA methods is regarded as most robust for calculating carbon emissions associated with interventions, yet studies using these methods are underrepresented in the evidence base. Many of the studies stating they used full LCA methods were, in fact, inventory analyses. This may reflect the methodological challenges and specific skillset required to conduct this type of study. Future research needs to ensure individuals conducting LCA studies have the support and resources required to carry out this study within healthcare settings and report the conduct and findings in a way that maintains transparency on methodological and system boundaries.
– Studies based on LCA methodology may not always be appropriate, necessary, or possible to action within healthcare settings, particularly when it is reasonable to assume a change in carbon emissions between intervention and/or control is associated with a particular material or process within the care pathway (e.g., patient transport saved for telehealth interventions). However, the carbon‐emission calculations used in these studies should reflect all relevant parts of both the patient‐care and carbon‐emission pathways associated with the intervention. It may be useful to develop guidelines to support researchers in considering which factors they need to consider within individual speciality/intervention groups. Such guidelines, in turn, could be used by systematic reviews to critically appraise studies using non‐LCA methods.
– Closely tied to this is the need to consider patient clinical and satisfaction outcomes alongside carbon‐emission outcomes. This was a key issue raised by our PPIE collaborators and would ensure that carbon emissions associated with all stages of the patient care pathway are considered (e.g., visits to primary care clinicians to manage complications) and ensure that patient health is not adversely affected by the intervention implemented.
– Comparisons between intervention and control groups should be supported through statistical analysis to increase confidence in the reported direction of research findings.
– There is the opportunity to integrate patient and public involvement into the development and implementation of new interventions and/or carry out qualitative research to gather patient views of interventions, supported by a higher number of effectiveness studies with a paucity of patient satisfaction data, such as telehealth.
– It would also be beneficial for future research to explore patient, carer and clinician understanding, acceptance and views on the feasibility of implementing potential changes to the health system that aim to reduce carbon emissions, to support the implementation of successful carbon‐lowering interventions.
– There is a need to review existing research which evaluates carbon emissions, patient health outcomes and cost implications associated with interventions which support patients' transition between secondary and primary care.
– Regarding interventions with a telehealth component, future research needs to ensure the digital carbon footprint is fully considered, alongside ensuring the technology is used effectively to maximise patient outcomes and reduce cost across primary and secondary care.
– Future interventions need to target known carbon hotspots within individual specialities, while also assessing and addressing known issues within specific local contexts.
– Further research is required to establish a validated threshold to differentiate between LCA and non‐LCA studies at high, medium and low risk of bias.

### 7.2 Implications for Policy

– Our EGM provides a resource to identify where gaps in primary evidence exist on the patient care pathway, both within and across different specialities, making it a useful tool to inform commissioning of future research.
– The narrative synthesis considers the quality and quantity of evidence available to support the use of specific interventions to reduce carbon emissions within individual specialities. Our review highlights the larger groups of evidence available pertaining to the use of telehealth care and reuseable surgical equipment across different specialities, and its methodological limitations, which may influence the commissioning of future research and implementation of interventions within secondary healthcare.

### 7.3 Implications for Practice

– There is tentative evidence to indicate that interventions which reduce the distance patients need to travel to access care are associated with reduced carbon emissions. However, the impact on patient clinical outcomes and patient satisfaction is inconclusive and further research which addresses the methodological limitations highlighted above is required to increase confidence in reported findings.
– There is tentative evidence to indicate that reuseable surgical equipment is associated with reduced carbon emissions when compared with single‐use equipment within certain specialities. However, this is influenced by the composition of the instrument and how the reprocessing of reuseable units is carried out (e.g., number of units reprocessed at any one time and duration of reprocessing procedures).

## Author Contributions

Liz Shaw led the development of the interactive EGM and strategic planning for drafting the report and refining map categories. Noreen Orr led with formatting and generation of content for the EGM, with Simon Briscoe, G. J. Melendez‐Torres, Ruth Garside, Hassanat M. Lawal and Jo Thompson Coon contributing towards its development.

Liz Shaw, Noreen Orr, Hassanat M. Lawal and Simon Briscoe carried out screening, data extraction and quality appraisal. Noreen Orr led on quality appraisal. Liz Shaw, Noreen Orr and Hassanat M. Lawal carried out citation chasing.

Simon Briscoe designed and ran the search strategies and managed the bibliographic libraries.

Liz Shaw led on the drafting, assembly, and formatting of the final report. Noreen Orr drafted sections of the report and read, provided feedback on, edited and approved the final version of the report. Jo Thompson Coon, Simon Briscoe, Hassanat M. Lawal, Clara Martin‐Pintado, Xiaoyu Yan, G. J. Melendez‐Torres and Ruth Garside read, provided feedback on, edited, and approved the final version of the report.

Xiaoyu Yan provided expertise on LCA methods.

Ruth Garside provided overall project management. G. J. Melendez‐Torres, Hassanat M. Lawal, Jo Thompson Coon, Simon Briscoe and Liz Shaw contributed to the scoping process, refining of research questions and development of the protocol in collaboration with the protocol authorship team. Liz Shaw led on stakeholder engagement/PPI, with support from all authors.

- **Content:** All authors have experience in health service and social care research. Although no authors have direct experience in conducting or evaluating interventions to reduce carbon emissions within healthcare settings, stakeholders with such expertise were involved throughout the development of the EGM.
- **EGM Methods:** All authors have previously worked on evidence gaps maps and have prior experience in conducting all stages of a systematic review.
- **Information Retrieval:** Simon Briscoe has training and extensive experience in designing and implementing search strategies.
- **Statistical Analysis:** G. J. Melendez‐Torres and Jo Thompson Coon have experience undertaking statistical analyses within systematic reviews.

## Conflicts of Interest

Jo Thompson Coon is a member of the NIHR Health Technology Assessment General Funding Committee. The other authors declare no conflicts of interest.

## Plans for Updating the EGM

There are no current plans to update this EGM. However, the authors will consider updating the EGM in the future if relevant funding is available.

## Differences Between Protocol and Review

Due to the high number of eligible studies identified by our search and screening strategy, we made the pragmatic decision to prioritise the studies using LCA informed methods to evaluate the impact of interventions to reduce carbon emissions, for full data extraction and quality appraisal. Our approach, as detailed in the methods section above, meant all eligible studies could be considered within the synthesis while prioritising the most robust evidence to inform decision making. The data extraction items for studies using LCA and non‐LCA methods are summarised in Appendix 2 Data extraction items for included studies.

## Sources of Support

**Internal Sources**

Jo Thompson Coon is supported by the National Institute for Health and Care Research Collaboration South West Peninsula. This report is independent research supported by the National Institute for Health Research Applied Research Collaboration South West Peninsula. The views expressed in this publication are those of the author(s) and not necessarily those of the National Institute for Health Research or the Department of Health and Social Care.

**External Sources**

This is an independent review commissioned and funded by the NIHR Policy Research Programme [NIHR200695].

## Supporting information

NetZero Supp Materials 1 copy.

NetZero Supp Materials 2 copy.

NetZero Supp Materials 3 copy individual outcomes.

NetZero Supp Materials 4 copy methods.

Appendix.

Excluded_studies.

## Data Availability

Data is available upon reasonable request from the lead author.

## Footnotes

* Sillcox, Blaustein, et al. (2023) and Sillcox, Gitonga, et al. (2023) two papers reporting same study.
