# Supplementary material for: Effectiveness of Interventions to Reduce Carbon‐Emissions Within Secondary Healthcare: Systematic Review and Evidence and Gap Map
Source: Campbell Syst Rev. 2025 Dec 23;21(4):e70077. doi: 10.1002/cl2.70077 (PMC12723626; doi:10.1002/cl2.70077)
Supplement: Supplementary file 1 — NetZero Supp Materials 1 copy. [file CL2-21-e70077-s001.docx]

# Supplementary Materials 1: Overview of included studies

| **Study, Country, [Publication Status]** | **Title** | **Specialty** | **Aim** | **Study Design** | **Funding statement** | **Conflict of interest as reported in paper** |
| --- | --- | --- | --- | --- | --- | --- |
| Andrew 2020, Australia [JAP] | Telehealth model of care for routine follow up of renal transplant recipients in a tertiary centre: A case study | Renal | Describe telehealth model of care used to provide routine follow up to patients’ post-kidney transplantations | Observational: Retrospective review of database (CE data only) | The author(s) declared no potential conflicts of interest with respect to the research, authorship, and/or publication of this article | The author(s) received no financial support for the research, authorship, and/or publication of this article |
| Arndt 2023, Germany [JAP] | COVID-19 measures as an opportunity to reduce the environmental footprint in orthopaedic and trauma surgery | O/T | Compare estimated CO2 emissions in 6-month period when conducting video consultations (VC) with period of exclusive F2F consultation in outpatient clinic of orthopaedics and traumatology surgery | Experimental: Before and After | Funding by the University Hospital of Bonn | The authors declare the research was conducted in absence of any commercial or financial relationships that couldbe construed as a potential conflict of interest |
| Asghari 2020, Middle East Iran/France [JAP] | A green delivery-pickup problem for home haemodialysis machines;sharing economy in distributing scarce resources | Renal | Improve the classic pickup and delivery models, to make them more useful for decision-makers to enhance performance of sharing operations while serving customers with timely home health services and provides the individuals a compact source of income | Modelling | NR | NR |
| Baboudijian 2023, France [JAP] | Life Cycle Assessment of Reusable and Disposable Cystoscopes: A Path to Greener Urological Procedures | Urology | Provide the first rigorous life cycle assessment of reusable anddisposable flexible cystoscopes | LCA | The evaluation of the carbon footprint by the third-party company was funded by Ambu (Ballerup,Denmark) | Michael Baboudjian certifies that all conflicts of interest, including specific financial interests and relationships and affiliations relevant to the subject matter or materials discussed in the manuscript (eg, employment/affiliation, grants or funding, consultancies, honoraria, stock ownership or options, expert testimony, royalties, orpatents filed, received, or pending), are the following: None. The funder of the study had no role in study design, data collection, dataanalysis, data interpretation, or writing of the report |
| Benedine 2020, France [JAP] | Haemodialysis therapy and sustainable growth: a corporate experience in France | Renal | Describe data collection implemented in the NephroCare centres in France and changesobserved during a 13-year period regarding environmentalparameters | Observational: retrospective cohort | NR | None declared |
| Beswick 2016, USA [JAP] | Consultation via telemedicine and access to operative care for patients with head and neck cancer in a Veterans Health Administration population | Oncology | Evaluate telemedicine model that utilizes an audiovisual teleconference as a preoperative visit | Feasibility study | NR | NR |
| Betts 2023, UK [Non-Peer Reviewed Project report] | A greener endoscopy unit for royal Cornwall Hospital | GE | 1) Organize the unit and empower staff to recycle all sterile water bottles used daily. 2) Change test reporting system to reduce waste and low value use of admin staff time | Experimental: Before and After | NR | NR |
| Bird 2022, UK [Non-Peer Reviewed Project report] | Changing the 3 monthly blood test postage kits for patients on the renal transplant register | Renal | Measure environmental, social and financial benefits of a new postal system vs old postal system. | Modelling | NR | NR |
| Boberg 2022, Sweden [JAP] | Environmental impact of single-use, reusable, and mixed trocar systems used for laparoscopic cholecystectomies | GE | 1.Evaluate and compare the environmental impacts of a single-use, a mixed, and a reusable trocar system for laparoscopic cholecystectomy. 2. Assess the financial costs of respective trocar system | LCA | Boberg received funding from Lund University Agenda 2030 Graduate School and Bentzer received Swedish Government Funding (ALF) (grant number: 86626). The funders had no role in study design, data collection and analysis, decision to publish, or preparation of the manuscript | The authors have declared that no competing interests exist |
| Bond 2009, UK [JAP] | Tackling climate change close to home: mobile breast screening as a model | Radiology | Compare distances travelled by patients attending mobile breastscreening clinics vs distance they would travel if screening services were centralized | Observational: retrospective database review | NR | NR |
| Burton 2022, UK-Wales [Non-Peer Reviewed Project report] | Sustainable ENT: Fractured Nose Manipulation- Local Anaesthetic Pathway | ENT | Support this pathway to be retained post-Covid and spread to other hospitals in Wales and the UK, by • Analysing the social, financial and environmental impact of the new fractured nose manipulation LA pathway. • Comparing our new LA pathway to the fractured nose manipulation GA pathway• Embedding this change within the department by educating clinicians on the benefits of the new pathway • Capturing data on patient satisfaction for fractured nose manipulation under LA | Modelling | NR | NR |
| Buttner 2021, Germany [JAP] | Switching off for future—Cost estimate and a simple approach to improving the ecological footprint of radiological departments | Radiology | Investigate whether switching off workstations after core working hours can lower energy consumption considering ecological and economical aspects | Cross-sectional/ modelling | This research received no specific grant from any funding agency in the public, commercial, or not-for-profit sectors | Not applicable |
| Chambrin 2023, France [JAP] | Association Between Anesthesia Provider Education and Carbon Footprint Related to the Use of Inhaled Halogenated Anesthetics | Multiple: 4 hospitials with surgical s activity: O+T, ENT, Oph, visceral, urology, GC, neurosurgery, obstetrics, cardiology, liver, kidney, pancreatic, cardiac, GE, radiology | Assess whether implementing information campaigns was associated with a decrease in carbon footprint related to inhaled halogenated anesthetics | Retrospective cohort (using database) | S.deS. Received a fee from the Association Generale de l'Internat de Lyon (general association of the residents of Lyon) to perform the statistical analyses | The authors declare no conflicts of interest |
| Chan 2022, UK [Non-Peer Reviewed Project report] | Reducing carbon (CO2E) waste from pulse lavage systems used in joint replacement surgery, orthopaedic theaters | O+T | 1. Evaluate and compare carbon footprint of Ecopulse vs Pulsvac Plus 2. Evaluate and compare cost of Ecopulse vs Pulsvac Plus 3. Clinical evaluation of Ecopulse by surgeons | Experimental: Before and After | Products provided by De Soutter free of charge and no funding required | NR |
| Chen 2017, China [JAP] | The carbon footprints of home and in-centre peritoneal dialysis in China | Renal | Determine carbon footprints of differing modalities and treatment regimes used to deliver PD. Results represent first assessment on carbon footprint of PD patients in Chinese population, which might improve understanding of PD-associated GHG emissions and facilitate carbon reduction strategies at level of PD treatments | Experimental: CTr | NR | All the authors declare that they have no conflict  of interest |
| Cheung 2023, Canada [JAP] | Evaluating the Short-term Environmental andClinical Effects of a Radiation OncologyDepartment’s Response to the COVID-19 Pandemic | Oncology | Assess environmental effect of single radiation oncology department’s collective strategic changes implemented during the pandemic to reduce foot traffic to the hospital by identifying sources of CO2e from healthcare and patient perspective | Experimental: Retrospective before and after. Quality improvement | NR | None |
| Chuter 2023, UK [JAP] | Towards estimating the carbon footprint of external beam radiotherapy | Radiation oncology | Estimate carbon footprint of various components of RT at multiple centres and identify areas to focus future mitigation efforts. In addition to this, COVID-19 has resulted in routine protocol changes (e.g. increased use of hypofractionation and PPE) that may have altered the totalcarbon footprint for each patient. This study therefore additionally aims to quantify and compare the carbon footprint across multiple centres before and during the pandemic | Observational -retrospective | RC would like to acknowledge funding from the Northwest Greener NHS Innovation Fund and support of Cancer Research UK via funding to the Cancer Research Manchester Centre [CTRQQR-2021\100010]. MA acknowledges the support of the Engineering and Physical Research Council (Grant number EP/T028017/1). TM acknowledges the support of Christie Charity & Engineering and Physical Sciences Council [grant number EP/R023220/1] | The authors declare that they have no known competing financial interests or personal relationships that could have appeared to influence the work reported in this paper |
| Connor 2011a, UK [JAP] | The carbon footprints of home and in-centre maintenance haemodialysis in the United Kingdom | Renal | Determine carbon footprints of differing modalities and treatment regimens used to deliver maintenance HD, to inform carbon reduction strategies at the level of individual treatments and HD program | Component analysis | NR | NR |
| Connor 2011b, UK [JAP] | The follow-up of renal transplant recipients by telephone consultation: three years’ experience from a single UK renal unit | Renal | Follow-up to renal transplant recipients over a three-year period. Benefits of this service to patients, providers and the environment are outlined, existing literature on the provision of virtual care to patients with kidney disease is reviewed, and the possibilities of more widespread adoption are discussed | Observational: Cross-sectional | NR | NR |
| Connor 2019, UK [JAP] | Clinical, fiscal and environmental benefits of a specialist led virtual ureteric colic clinic: a report of a prospective study | Urology | Evaluate clinical, fiscal and environmental impact of a specialist-led acute ureteric colic VC pathway | Observational: Prospective cohort | Martin J. Connor is funded by the Wellcome Trust | Martin J. Connor is funded by the Wellcome Trust. All other authors have nothing to disclose |
| Coombs 2016, UK [JAP] | Environmental and social benefits of the targeted intraoperative radiotherapy forbreast cancer: data from UK TARGIT-A trial centres and two UK NHS hospitals offering TARGIT IORT | Oncology | Quantify journeys and CO2 emissions of women with breast cancer, treated with risk-adapted single-dose TARGIT vs several weeks’ course of EBRT treatment | Experimental: RCT | The TARGIT-A trial was supported by University College LondonHospitals (UCLH)/UCL Comprehensive Biomedical Research Centre, UCLHCharities, National Institute for Health Research Health TechnologyAssessment programme, Ninewells Cancer Campaign, National Health andMedical Research Council, and German Federal Ministry of Education andResearch | NJC, JMC, MiB, JST, FW, DJ, TC, MF, IP, NW and JSVdeclare the cost of travelling to some meetings where TARGIT treatment isdiscussed, from Carl Zeiss, the manufacturer of Intrabeam. JSV declareshonoraria when speaking about TARGIT at some meetings |
| Cooper 2022, UK [JAP] | Exploring the Impact and Acceptance of Wearable Sensor Technology for Pre- and Postoperative Rehabilitation in Knee Replacement Patients: A UK based pilot study | O+T | Understand if use of digital system could improve patient experience and efficiency of postoperative physiotherapy care provided, by: (1) exploring if wearable sensor would reduce number of F2F physiotherapy visits (2) measuring how well received and utilized messaging system was by patients and clinicians | Prospective, observational, single-arm feasibility study | See Cooper 2023 | See Cooper 2023 |
| Cooper 2023, UK [JAP] | The Economic Impact of a Pilot Digital Day-Case Pathway for Knee Arthroplasty in a U.K. Setting | O+T | Assess impact of implementing digital day-case pathway for knee replacement surgery at Calderdale and Huddersfield NHS Foundation trust | Experimental: Pilot controlled trial - CE data based on modelling | BPMpathway sensors provided free of charge courtesy of B. Braun Medical U.K., which distribute the BPMpathway. D.M.C. is employed by B. Braun, and G.W. was paid an honorarium fee by B. Braun to present this work on a B. Braunwebinar | Cooper: previously employed by B Braun as Lead medical science liaison. Hepworth: employed by B Braun as associate medical science liaison. Walsh: Payment for delivering an online presentation on the wearable at a BBraun meeting, $500 payment |
| Croghan 2021, Ireland [JAP] | Time, cost and carbon-efficiency: a silver of COVID era virtual urology clinics? | Urology | Evaluate impact of virtual clinics (VC) on travel time, monetary cost to patients and carbon emissions | Observational: Cross-sectional | NR | NR |
| Cunha Neves 2023, Portugal [JAP] | Targeted intervention to achieve waste reduction in gastrointestinal endoscopy | GE | Measure single unit’s waste carbon footprint and intervention towards a more sustainable endoscopy practice. Primary aims: Assess and compare waste carbon footprint and waste processing expenses induced by endoscopic procedures before and after intervention 2. Reorganise endoscopy unit to reduce and recycle endoscopic waste (intervention). Secondary aims: 1. Evaluate project’s impact on unit’s productivity and staff daily labour; 2. Assess waste carbon footprint of a single diagnostic upper endoscopy and a single diagnostic colonoscopy before and after intervention 3. Assess whether behavioural changes implemented still in practice 4 months after intervention | Experimental: Before and After | The authors have not declared a specific grant for this research from any funding agency in the public, commercial or not-for-profit sectors | None declared |
| Curtis 2021, UK [JAP] | Remote Clinics During Coronavirus Disease 2019: Lessons for a Sustainable Future | O+T | Establish whether non F2F clinics are sustainable according to “triple bottom line” framework, considering impact on patients, the planet, and financial cost | Observational: Retrospective cohort | All authors have declared that no financial support was received fromany organization for the submitted work | In compliance with the ICMJE uniform disclosure form, all authors declare thefollowing: Financial relationships: All authors declared they have no financial relationships at present or within the previous three years with any organizations that might have an interest in the submitted work. Other relationships: All authors declared there are no other relationships or activities that could appear to have influenced the submitted work. |
| Davis 2018, Australia [JAP] | Footprint in Flexible Ureteroscopy: A Comparative Study on the Environmental Impact of Reusable and Single-Use Ureteroscopes | Urology | Evaluate and compare environmental impact of SU and reusable flexible ureteroscopes | LCA: Incomplete impact assessment | NR | No competing financial interests exist |
| de Preux 2018, UK [JAP] | Beyond financial efficiency to support  environmental sustainability in economic evaluations | Renal | 1.Review the concepts of financial and environmental sustainability. 2. Discuss existing evidence of sustainable changes within this sector. 3. Propose a simple adaptation of the classic cost-effectiveness analysis to incorporate carbon foot printing to account for these external costs by using the case of in-centre versus home haemodialysis | Modelling | NR | NR |
| de Ridder 2022, Netherlands [JAP] | A New Method to Improve the Environmental Sustainability ofthe Operating Room: Healthcare Sustainability Mode andEffect Analysis (HSMEA) | Obstetrics | Describe a new method to effectively improve the environmental impact of operating rooms through a systematic approach. Primary research question: whether possible to transform the HFMEA into a tool for identifying and addressing the environmental impact of OR waste. Secondary research question: investigated whether application of this novel method, the HSMEA, could identify carbon hotspots in surgical waste and provide solutions for improvement | Healthcare Sustainability Mode and Effect Analysis and case study | This research did not receive any specific grant from funding agencies in the public, commercial, or not-for-profit sectors | The authors declare no conflict of interest |
| Dorrian 2009, UK [JAP] | Head and neck cancer assessment by flexible endoscopy and telemedicine | ENT | Feasibility study to establish whether ENT tele-endoscopy would be a suitable method of service delivery for patients in the Shetland Island | Observational: Prospective cohort | The Health Economics Research Unit isfunded by the Chief Scientist Office of the Scottish Government Health Directorate | NR |
| Field 2023, USA [JAP] | Environmental and Economic Impact of Using a Higher Efficiency Ventilator and Vaporizer During Surgery Under General Anesthesia: ARandomized Controlled Prospective Cohort | Multiple: General, GC Oph, Urology | Assess: a) whether low-volume anesthesia machines, such as the MQ, deliver volatile anesthetics more efficiently than traditional anesthesia machines, such as the GE b) whether this wasin a meaningful economic or environmentally conscious way | RCT | In compliance with the ICMJE uniform disclosure form, all authors declare the following: Payment/services info: This study was made possible by a grant from the Getinge Group (Grant number: IRB 2014-1248) | All authors have declared that they have no financial relationships at present or within the previous three years with any organizations that might have an interest in the submitted work. Otherrelationships: All authors have declared that there are no other relationships or activities that could appearto have influenced the submitted work |
| Filfilan 2021, France [JAP] | Positive environmental impact of remote teleconsultation in urology during the COVID-19 pandemic in a highly populated area | Urology | Assess environmental cost of teleconsultations vs F2F consultations in urology. | Observational: Cross-sectional | NR | The authors declare that they have no competing interest |
| Forner 2021, Canada [JAP] | Carbon footprint reduction associated with a surgical outreach clinic | Oncology | Estimate the carbon footprint savings of a head and neck surgery outreach clinic | Observational:Cross-sectional survey | This study was completed without funding | SMT is the attending surgeon of the head and neck surgical oncology outreach clinic and receives standard remuneration for patient care. Otherwise, the authors have no intellectual or financial conflicts of interest |
| Frick 2023, USA [JAP] | Effect of Radiation Schedule on TransportationRelated Carbon Emissions: A Case Study in RectalCancer | Oncology | Characterize the outcomes of a hypo fractionated radiation schedule for transportation-associated GHG emissions using rectal cancer as a case study | Experimental: CT | This work had no specific funding | All authors declare that they have no conflicts of interest |
| Fuschi 2023, Italy [Italy] | The impact of radical prostatectomy on global climate: a prospective multicentre study comparing laparoscopic versus robotic surgery | Urology | 1.Investigate environmental impact of minimally invasive surgery by comparing CO2 emissions of laparoscopic vs robot-assisted procedures, focusing on carbon footprint defined by GHG protocol and LCA to evaluate environmental impact of robot-assisted and laparoscopic instruments used for radical prostatectomy. 2.Evaluate life cycle cost analysis of surgical and anaesthetic products, considering the unit, decontamination, and disposal costs | LCA | NR | NR |
| Hardy 2022, UK [Non-Peer Reviewed Project report] | Review of the haemodialysis process in a single satellite dialysis unit with the aim to reduce carbon and waste | Renal | To reduce carbon and waste by:1. Reducing number of disinfections of dialysis machines to 1x24 hrs in staggered manner and replacing others with rinsing process. 2. When initial priming process of the dialysis machines complete, placing them in standby mode whilst waiting to connect patients to machine.3. Reducing number of pharmacy deliveries to satellite dialysis unit from weekly to biweekly. 4. Reviewing processes to enable usage of wastewater generated during water purification for dialysis. 5. Identifying potential ways of reducing wastage of canister acid 6. Recycling empty acid canisters | Modelling | NR | NR |
| Heye 2023, Switzerland [JAP] | Turn It Off! A Simple Method to Save Energy and CO2Emissions in a Hospital Setting with Focus on Radiology by Monitoring Nonproductive Energy-consuming Devices | Radiology | Identify idle energy-consuming imaging modalities and electronic devices in a hospital setting to reduce energy consumption and CO2 emissions | Observational: prospective cohort | NR | T.H. No relevant relationships. M.T.M.No relevant relationships. E.M.M. Institutional research support from Siemens Healthineers; residency program support from Bayer. J.V. No relevant relationships |
| Hogan 2022, NR [JAP] | The Carbon Footprint of Single-Use Flexible Cystoscopes Compared with Reusable Cystoscopes | Urology | Compare carbon footprint of single-use vs reusable flexible cystoscopes based on waste production and estimated carbon emissions | Prospective single-centre cohort study: controlled trial/Simplified LCA | NR | All authors confirm that they have no conflicts of interest to disclose |
| Holmner 2014, Sweden [JAP] | Carbon Footprint of Telemedicine Solutions - Unexplored Opportunity for Reducing Carbon Emissions in the Health Sector | Hand and plastic surgery, ENT | Evaluate potential of telemedicine services based on videoconferencing (VC) technology to reduce travellingand carbon emissions in healthcare sector | Modelling: Inventory analysis | This work was partly undertaken within the Umea˚ Centre for Global Health Research, with support from FAS, the Swedish Council for Working Life andSocial Research (grant no. 2006-1512). Also supported in part by funding from the Swedish International Development Cooperation Agency (SIDA). The funders had no role in study design, data collection and analysis, decision to publish, or preparation of the manuscript | ClimAdapt, LLC is a sole proprietorship consulting company working on health and global change issues. Clients are predominantlyinternational organizations, such as WHO and UNEP, and national institutions, such as Health Canada. Dr. Kristie Ebi conducts research on the impacts of and adaptation to climate change, including on extreme events, thermal stress, foodborne safety and security, and vectorborne diseases. Her work focuses onunderstanding sources of vulnerability and designing adaptation policies and measures to reduce the risks of climate change in a multi-stressor environment.Hence, the authors can confirm that this does not alter their adherence to all PLOS ONE policies on sharing data and materials. |
| Jiang 2021, USA [JAP] | Teleoncology for Veterans: High Patient Satisfaction Coupled With Positive Financial and Environmental Impacts | Oncology (including medical, surgical or radiation oncology) | 1.Use COVID-19pandemic as natural experiment to better understand tele oncology’s potential to facilitate VHA-based care, and determine viability of moving forward with this modality, and its role in a disproportionately rural population. 2. Assess the satisfaction and perspective of Veterans with cancer receiving teleoncology care to determine willingness to engage with technology. 3.Generate estimates of both private and social, financial, and environmental impacts of tele oncology to inform policy trade-offs in the future | Observational: Retrospective cohort | Garth W. StrohbehnPatents, Royalties, Other Intellectual Property: Co-inventor of a filed patentheld by the University of Chicago covering the use of low-dose tocilizumab in the treatment of viral infections. Nithya Ramnath Research Funding: Merck, Clovis Oncology. No other potential conflicts of interest were reported | The following represents disclosure information provided by authors of this manuscript. All relationships are considered compensated unless otherwise noted.Relationships are self-held unless noted. I= Immediate Family Member, Inst= My Institution. Relationships may not relate to the subject matter of this manuscript.For more information about ASCO’s conflict of interest policy, please refer to www.asco.org/rwc or ascopubs.org/op/authors/author-center. Open Payments is a public database containing information reported by companies about payments made to US-licensed physicians |
| Kemble 2023, USA [JAP] | Environmental impact of single‐use and reusable flexible cystoscopes | Urology | Assess and compare carbon footprint of SU and reusable flexible cystoscopes throughout the lifecycle of production and clinical use.Awareness of the environmental costs of these high-volume devices may help guide policy decisions and promote environmental stewardship in urology | Inventory Analysis | NR | The authors declare no conflicting interests. Brian R. Matlaga and Jared S. Winoker are consultants for Boston Scientific Corporation, unrelated to this work |
| King 2023, UK [JAP] | Towards NHS Zero: greener gastroenterology and the impact of virtual clinics on carbon emissions and patient outcomes. A multisite, observational, cross-sectional study | GE | Calculate true reduction in carbon emission resulting from transition to virtual consultations (VC) during global pandemic and assess safety of these appointments when compared with traditional F2F consultations | Observational: Retrospective cross-sectional | The authors have not declared a specific grant for this research from any funding agency in the public, commercial or  not-for-profit sectors | None declared |
| Klein 2023, Germany [JAP] | A New Approach to the Improvement of Energy Efficiencyin Radiology Practices | Radiology | Examine ways to improve energy efficiency in radiology by using regenerative and energy-friendly technology in construction and operation of two radiological facilities. | CTr | NR | The authors declare that they have no conflict of interest |
| Kodumuri 2022, UK [Non-Peer Reviewed Project report] | SusQI project report: The upper “hand” of sustainability:  Reducing the carbon footprint in hand surgery | O+T | Reduce carbon footprint of carpel tunnel surgery by; 1. adapting a “lean and green theatre set up” by reducing number of single use instruments, trays and drapes used per procedure. 2. transforming to a “Green CTR patient pathway” by utilizing a minor procedure room (instead of theatre) and therefore bypassing the pre-theatre ward admission process | Experimental: Before and After | NR | NR |
| Kodumuri 2023, UK [JAP] | Reducing the carbon footprint in carpaltunnel surgery inside the operatingroom with a lean and green model: acomparative study | O+T | 1) Determine the carbon footprint of a carpal tunnel release. 2) Construct and implement the lean and green model for the operation. Financial costs associated with both models were determined according to our quality improvement methods. 3) Comment in semiquantitative fashion on environmental, financial and social impacts of the study | Experimental: Before and After | The authors received no financial support for the research, authorship, and/or publication of this article | The authors declare no potential conflicts of interest with respect to the research, authorship, and/or publication of this article |
| Lambert 2023, Canada [JAP] | Impact of Cancer-Related Virtual Visits on Travel Distance,Travel Time, and CO2 Emissions during theCOVID-19 Pandemic in Manitoba, Canada | Oncology | Describe patterns of visit types (in-person vs virtual) over time during the pandemic at CancerCareManitoba, and the impact of virtual visits on hypothetical travel distance, travel time, and CO2 emissions generated by travel | Observational: retrospective database review | This work was supported by a research grant from Research Manitoba and the CancerCare Manitoba Foundation (2020 to 2021, funding reference number 4459) and the Canadian Institutes of Health Research (2022 to 2024, funding reference number 179890) | The authors declare no conflict of interest. The funders had no role in the design of the study; in the collection, analyses, or interpretation of data; in the writing of the manuscript; or in the decision to publish the results |
| Langstaff 2023, UK [Non-Peer Reviewed Project report] | PMB: Using light therapy for oral mucositis, palliative care team | Oncology | Evaluate clinical, social, financial and environmental impacts of PBM as supplemental treatment for prevention and/or reduction of oral mucositis for base of tongue and tonsil oncology patients undergoing radical radiotherapy +/ chemotherapy | Experimental: Before and After | NR | NR |
| Le 2022, USA [JAP] | Environmental and health outcomes of single-use versus reusable duodenoscopes | GE | Perform exploratory LCA comparing environmental and human health effects of single vs reuseable duodenoscopes | LCA | NR | NR |
| Leapman 2023, USA [JAP] | Environmental Impact of Prostate Magnetic Resonance Imaging and Transrectal Ultrasound Guided Prostate Biopsy | Urology | Estimate environmental impacts of prostate MRI and prostate biopsy | LCA: Incomplete impact assessment | None | Michael S. Leapman certifies that all conflicts ofinterest, including specific financial interests and relationships and affiliations relevant to the subject matter or materials discussed in the manuscript (eg,employment/affiliation, grants or funding, consultancies, honoraria, stock ownership or options, expert testimony, royalties, orpatents filed, received, or pending), are the following: None |
| Lewis 2009, UK [Non-Peer Reviewed Project report] | Use of videoconferencing in Wales to reduce carbon dioxide emissions, travel costs and time | Oncology | Evaluate environmental impact of using videoconferencing (VC) vs meeting in person | Observational: Cross-sectional | NR | NR |
| Lopez-Munoz 2023, Spain [JAP] | Life cycle assessment of routinely used endoscopic instruments and simple intervention to reduce our environmental impact | GE | 1.Determine endoscopic instrument composition, LCA, and assess a sustainability proposal based on a mark on instruments that identifies parts that can be safety recyclable or a ‘green mark’, to understand environmental impact of daily practice.2. Evaluate sustainability and composition environmental impact of commonly used endoscopy instruments (biopsy forceps, polypectomy snares and haemostatic clips) from four different manufacturers, quantifying the parts that could be recycled. Primary outcome: determination of endoscopic instrument composition and environmental impact with LCA of total number of biopsy forceps, polypectomy snares and haemostatic clips used during one-week period. Secondary outcome: perform prospective intervention based on a green mark to evaluate differences in carbon footprint | LCA: Incomplete impact assessment | The authors have not declared a specific grant for this research from any funding agency in the public, commercial or not-for-profit sectors | None declared |
| Materacki 2023, UK [Non-Peer Reviewed Project report] | Scoping for change – adopting greener practice in endoscopy in Gloucestershire, Endoscopy team | GE | 1.Establish a multi-professional green endoscopy working group in Gloucestershire.2. To make at least one change to improve sustainability in endoscopy in Cheltenham General Hospital and measure its environmental (CO2e), financial and social impact. | Experimental: Before and After | NR | NR |
| McAlister (2022), Australia [JAP] | The carbon footprint of hospital diagnostic imaging in Australia | Radiology | Estimate carbon footprint of five common imaging modalities within an Australian public hospital setting: CT; MRI; US; CXR; and MCXR in a “cradle to grave” life cycle assessment | LCA: Incomplete impact assessment | The funding sources played no role in the study design, data collection, data analysis, interpretation, or the writing of the manuscript | Scott McAlister was funded by a National Health andMedical Research Council of Australia (NHMRC) PhDscholarship, and from the Healthy Urban Environments(HUE) Collaboratory of the Maridulu Budyari GumalSydney Partnership for Health, Education, Researchand Enterprise MBG SPHERE.Alexandra Barrett is funded by a National Health andMedical Research Council of Australia (NHMRC) Centre of Research Excellence Grant, No 1004136. Kate Charlesworth was funded from the Healthy Urban Environments (HUE) Collaboratory of theMaridulu Budyari Gumal Sydney Partnership forHealth, Education, Research and Enterprise MBG SPHERE |
| McCarthy 2014, Ireland [JAP] | "EcoRadiology"--pulling the plug on wasted energy in the radiology department | Radiology | Perform an energy audit of department to identify where savings could be made. Re-audited energy use 18 months after an educational session within the department | Experimental: Before and After | NR | NR |
| McLachlan 2021, New Zealand [JAP] | An NP-led pilot telehealth  programme to facilitate  guideline-directed medical  therapy for heart failure  with reduced ejection  fraction during the  COVID-19 pandemic | Cardiology | Using latest decision pathway for optimisation of heart failure treatment, aim to facilitate titration while limiting in-person clinic visits by using patient self-monitoring with a package that included funded home BP monitors and electronic scales alongside NP-led telephone support for patients with HFrEF | Experimental: Before and After | NR | Dr Lund reports other from Amgen inc and personal fees from Novartis outside the submitted work |
| Meiklejohn 2023, USA [JAP] | Environmental impact of adult tonsillectomy: life cycle assessment and cost comparison of techniques | ENT | Quantify and compare cost and environmental impact of different techniques for adult tonsillectomysurgery, and identify target areas for impact reduction. We report the first formal evaluation of thecomprehensive environmental impact of an otolaryngologic surgery, using LCA to compare three commonlyused surgical techniques for adult tonsillectomy. Hypothesized that cold technique would have reduced costand environmental impact, across all categories of environmental impact, compared to either ME or Coblation | LCA | NR | NR |
| Miah 2019, UK [JAP] | A prospective clinical, cost and environmental analysis of a clinician-led virtual urology clinic | Urology | Quantify the clinical, financial, and environmental benefits of virtual urology clinic | Observational: Prospective cohort | NR | NR |
| Milne 2010, UK [Non-Peer Reviewed Project report] | Green nephrology: retro-fit of heat exchangers to haemodialysis machines- case study and how-to guide | Renal | Investigate potential costs and benefits of retro-fitting heat exchangers to their existing Braun Dialog+ haemodialysis machines | Modelling | NR | NR |
| Milne 2023, UK [Non-Peer Reviewed Project report] | Retro-fit of Heat Exchangers to Haemodialysis Machines - Case Study and How to Guide | Renal | Investigate the possibility of retro-fitting heat exchangers to their existing machines | Modelling | NR | NR |
| Mojdehbakhsh 2021, N [USA] | A quality improvement pathway to rapidly increase telemedicine services in a gynaecologic oncology clinic during the COVID-19 pandemic with patient satisfaction scores and environmental impact | Gyn oncology | 1. Convert at least 50% of all outpatient clinical encounters to telemedicine, and 2. Have 100% documentation of telemedicine consent in providers’ notes, both within one week of initial intervention.3. Elicit patient feedback regarding this new type of encounter and determine potential interpersonal impact on patient care. 3. Calculate CO2 emissions prevented from patient travel to demonstrate important secondary effect of transitioning to telemedicine | Observational: Cross-sectional | NR | The authors declare that they have no known competing financial interests or personal relationships that could have appeared to influence the work reported in this paper |
| Moussa 2021, UK [JAP] | Environmental effect of fluorinated gases in vitreoretinal surgery: a multicentre study of 4,877 patients | Oph | Investigate the direct contribution to carbon emissions of fluorinated gases used in VR procedures utilizing gas tamponade and assess respective carbon footprint of the three different gas delivery systems | Retrospective, continuous, comparative multicentre study | There are no external funders that have played a role in study design, data collection and analysis, decision to publish, or preparation of the manuscript | All authors have no conflict of interest in the production of this manuscript |
| Moussa 2022, UK [JAP] | Environmental effect of air versus gas tamponade in management of rhegmat-ogenous retinal detachment VR surgery: A multicentre study of 3,239 patients | Oph | Report the potential reduction of carbon emissions by utilising AT instead of fluorinated gases in management of RRDs. Compare the CO2 emissions produced at two large tertiary referral VR centres where RRD are exclusively repaired using fluorinated gases to a tertiary VR centre that employs AT in selected RRD cases | Retrospective, continuous, comparative multicentre study | GM: University of Birmingham - supplying open access fees. No the funders had no role in study design, data collection and analysis, decision to publish, or preparation of the manuscript | The authors have declaredthat no competing interests exist |
| Muschol 2022, Germany [JAP] | Economic and Environmental Impact of Digital Health App Video Consultations in Follow-up Care for Patients in Orthopaedic and Trauma Surgery in Germany: Randomized Controlled Trial | O+T | Provide the first health economic analysis comparing telemedicine in follow-up of patients in orthopaedic and trauma surgery with knee and shoulder disorders, with conventional F2F examinations in German clinic | RCT | NR | None declared |
| Natale 2022, UK [JAP] | Tele-consultation versus traditional clinical assessment of patients undergoing circumcision: A retrospective cohort study | Urology | Determine whether standalone teleconsultation is an effective alternative to F2F assessment ofpatients requiring circumcision. Determine environmental and efficiency benefits of service alteration | Observational: Retrospective cohort | The author(s) received no financial support for the research, authorship, and/or publication of this article | The author(s) declared no potential conflicts of interest with respect to the research, authorship, and/or publication of this article |
| Nielson 2022, UK [Non-Peer Reviewed Project report] | Pioneering Early Mobilisation in a Cardiac Intensive Care unit: a Sustainable Healthcare Initiative | Cardiology | NR | Experimental: Before and After | NR | NR |
| Owens 2023, UK [Non-Peer Reviewed Project report] | Collaborative report of paper lite and contrast recycling projects, endoscopy team | GE | 1. Reduce printing and paper use in the Endoscopy department by transitioning to electronic working 2. To redirect Contrast waste from sharps (incineration) disposal to be recycled | Modelling | NR | NR |
| Patel 2023, USA [JAP] | Estimated Carbon Emissions Savings With Shifts From In-Person Visitsto Telemedicine for Patients With Cancer | Oncology | Assess carbon savings from telemedicine visits | Observational: Cross-sectional | Dr Gonzalez reported receiving personal fees from Sure Med Compliance and personal fees from Elly Health outside the submitted work | Dr Rollison reported service on the board of directorsfor NanoString Technologies outside the submitted work. Dr Spiess reported service as vice-chair of the NationalComprehensive Cancer Network Bladder and Penile Cancer Panel, president of the Global Society of RareGenitourinary Tumors, and panel member of the American Society of Clinical Oncology and European Association of Urology. No other conflicts were reported |
| Phull 2023, UK [JAP] | Potential Carbon Savings with Day-case Compared to Inpatient Transurethral Resection of Bladder Tumour Surgery in England: A Retrospective Observational Study Using Administrative Data | Urology | Investigate the estimated difference in carbon footprint between day case and inpatient TURBT surgery inEngland | Observational: Retrospective Review of Data | Funding/Support and role of the sponsor: None | William K. Gray certifies that all conflicts of interest, including specific financial interests and relationships and affiliations relevant to the subject matter or materials discussed in the manuscript (eg, employment/affiliation, grants or funding, consultancies, honoraria,stock ownership or options, expert testimony, royalties, or patents filed, received, or pending), are the following: None |
| Richards 2022, UK [JAP] | Virtual Arthroplasty Follow-Up: Better for theTrust, Patients, and the Planet | O+T | Examine outcomes of shifting part of arthroplasty follow-up service to virtual system called VARF, and the benefits for the trust, patients, and planet. This system aimed to maintain or improve patient experience, reduce F2F attendance, improve cost efficiency, and reduce cost to the environment without adverse impact on the quality of care | Observational: retrospective cohort | All authors have declared that no financial support was received from any organization for the submitted work | All authors have declared that they have no financial relationships at present or within the previous three years with any organizations that might have an interest in the submitted work. All authors have declared that there are no other relationships or activities that could appear to have influenced the submitted work |
| Rizan 2022, UK [JAP] | Environmental impact and life cycle financial cost of hybrid (reusable/single‑use) instruments versus single‑use equivalents in laparoscopic chole-cystectomy | GE | Compare environmental and financial life cycle cost of currently available hybrid instruments for laparoscopic cholecystectomyand compare these to single-use equivalents | Modelling; LCA | This work was funded by Surgical Innovations Ltd. who manufacture hybrid laparoscopic instruments. The company played no part in scientific conduct, analysis, or writing of this manuscript | NR |
| Rouviere 2022, France [JAP] | Ecoresponsible actions in operating rooms: A health ecological and economic evaluation | Multiple: Neuro surgery, GE, oto-laryngology, oph, O+T, plastic, vascular, GC, urology and digestive surgery) and anaesthesia | Assess ecological and economic impacts of sustainable actions targeting medical devices designed by a multidisciplinary working group and implemented in 24 operating rooms of a University Hospital over one year | LCA and prospective pilot study | ANFH (approved by the French Ministry of Health) supported the Primum Non Nocere® agency costs for the ecological impact analysis | All the authors have declared no potential conflicts of interest regarding this study |
| Sanchez 2020, France [JAP] | Environmental and economic comparison of reusable and disposable bloodpressure cuffs in multiple clinical settings | Multiple inc. ICU | Assess environmental and economic impacts of reusable and disposable BP cuffs | Modelling; LCA | Financial support Dept. of Civil and Environmental Engineering, Northeastern University | The authors declare that they have no known competing financial interests or personal relationships that could have appeared to influence the work reported in this paper |
| Schulte 2021, Germany [JAP] | Combining Life Cycle Assessment and Circularity Assessment to Analyse Environmental Impacts of the MedicalRemanufacturing of Electrophysiology Catheters | Cardiology | Analyze environmental consequences of electrophysiology catheters considering two modelling perspectives, the implementation of LCA, including a cut-off approach and combining LCA and a circularity indicator measuring multiple life cycles | LCA | This research and APC was funded in part by the Fraunhofer-Gesellschaft with sponsoredresearch agreement with industry partner Vanguard AG, Germany | The data for the provision of a catheter through medical remanufacturing was gathered from the company Vanguard AG in Germany. However, the Vanguard AG had no role in the design of the study, the analyses, and the interpretation of data or in the writing of the manuscript. The authors declare no conflict of interest |
| Sherman 2018, USA [JAP] | Life Cycle Assessment and Costing Methods for Device Procurement: Comparing Reusable and Single-Use Disposable Laryngoscopes | GE | Provide quantitative comparisons of environmental impacts and total cost of ownership among laryngoscope options, which can aid procurement decision making to benefit facilities and public health | LCA | J.D.S. was supported by an Anaesthesia Patient Safety Foundation award. L.A.R. was supported by a Provost’s award for undergraduate | The authors declare no conflicts of interest |
| Sillcox 2023a, USA [JAP] | Telemedicine Use Decreases the Carbon Footprint of the Bariatric Surgery Preoperative Evaluation | GE | Hypothesized that telemedicine would  decrease carbon emissions, improve patient compliance with appointments, and decrease overall preoperative evaluation time to surgery | Observational: retrospective review | NR | The authors declare no competing interests |
| Sillcox 2023b, USA [JAP] | The environmental impact of surgical telemedicine: life cycle assessment of virtual vs. in‑person preoperative evaluations for benign foregut disease | GE | Aimed to estimate environmental impact of implementing telemedicine visits for evaluation of benign foregut disease at their institution during the Covid-19 pandemic | Inventory analysis | NR | Drs. Sillcox, Meiklejohn, Wright, Oelschlager, Bryant, Tarefder and Zhu, Baraka Gitonga, and Zafrul Khan have no conficts of interest to disclose |
| Sorensen 2018, Denmark [JAP] | Comparative Study on Environmental Impacts of Reusable and Single-Use Bronchoscopes | Respiratory | Evaluate CO2-equivalent emissions and resource consumption from SU bronchoscope like Ambu® aScopeTM 4 broncho vs those for materials used to clean flexible RBs | LCA (although only considered 2 impact categories) | This study has been funded and by Ambu a/s | The authors have no competing interests |
| Stripple 2008, Sweden [JAP] | Development and environmental improvements of plastics for hydrophilic catheters in medical care: environmental evaluation | Urology | Analyse environmental performance of three different plastic materials for urinary catheters: a bulk plastic material (PVC), a high quality performance plastic (TPU) and a newly developed plastic material based on the experiences from the present environmental evaluations (a polyolefin-based elastomer) | LCA | Financial support for the study described in this paper was provided by IVL Swedish Environmental Research Institutethrough the Swedish Environmental Protection Agency (Naturva˚rdsverket) and by Astra Tech AB. The latter has also provided valuable technical background information | NR |
| Thiel 2015, USA [JAP] | Environmental Impacts of Surgical Procedures: Life Cycle Assessment of Hysterectomy in the United States | GC | Analyse life cycle impacts of a single surgical procedure, using four different surgical methods. Because OR is most resource-intensive area of a hospital, understanding environmental impact of surgery is critical to understanding healthcare-related emissions in general | LCA | Financial support for the data collection and management ofthis project came from Grant Number ULI RR024153 from theNational Centre for Research Resources (NCRR), acomponent of the National Institutes of Health (NIH), andNIH Roadmap for Medical Research. Support for graduatestudent researchers came from Award No. 050434 from theNational Science Foundation (NSF) Integrative GraduateEducation and Research Traineeship (IGERT) | The authors declare no competing financial interest |
| Thiel 2018, USA [JAP] | Strategies to Reduce Greenhouse Gas Emissions from Laparoscopic Surgery | GC | 1.Determine carbon footprint of various sustainability interventions used for laparoscopic hysterectomy. 2.Identify and model the impact of sustainability interventions in the OR | Inventory analysis | NR | NR |
| Thiel 2023, USA [JAP] | Telemedicine and the environment: life cycle environmentalemissions from in-person and virtual clinic visits | Multiple: Psychiatry, medical specialties, pain manage-ment, GE, oncology, oph, plastic surgery, O+T, oto-laryngology | Determine environmental emissions associated with in-person and virtual clinic visits | LCA | This work was funded by the Sean N. Parker Centre for Allergy and Asthma at Stanford University | The authors declare no competing financial or non-financial interests. Unrelated to this publication, author C.L.T. is a consultant through Clinically Sustainable Consulting LLC, which has contracts with Becton Dickinson, Philips, Stryker, and Zabble, Inc |
| Thota 2020, USA [JAP] | Telehealth Is a Sustainable Population Health Strategy to Lower Costs and Increase Quality of Health Care in Rural Utah | Oncology | Can telehealth between a tertiary cancer centre and rural health systems improve access to cancer care, decrease financial burdens, save time for patients with cancer living in rural Utah, and support local health delivery systems? | Observational: retrospective cohort | NR | No potential conflicts of interest were reported |
| Tselapedi-Sekeitto 2023, Canada [JAP] | Telemedicine as an environmental ally - The social, financial, and environmental impact of virtual care in otolaryngology clinic | ENT: Oto-laryngology | Investigate patients' satisfaction, travel cost, productivity loss, and CO2 emissions involved with synchronous virtual care and in-person assessments in rhinology and sleep apnoea clinics | Prospective comparative study | This research did not receive any specific grant from funding agencies in the public, commercial, or not-for-profit sectors. | None declared |
| Udayaraj 2019, UK [JAP] | Establishing a tele-clinic service for kidney transplant recipients through a patient-codesigned quality improvement project | Renal | Adopted a Quality Improvement approach with iterative PDSA cycles to test the introduction of a tele-clinic service. Project aimed to reduce number of patients not attending face-to-face clinics by offering a tele-clinic option | Observational: Before and After, Iterative PDSA cycles | We would like to thank the West of England Academic Health Sciences Network for their quality improvement expertise and for funding this project | AP reports personal fees from Vifor Fresenius Renal Pharma, personal fees from Bayer GmBH outside the submitted work |
| Vaidya 2022, Multi-country [JAP] [Linked to Coombs] | Global adoption of single-shot targeted intraoperative radiotherapy (TARGIT-IORT) for breast cancer-better for patients, better for healthcare systems | Oncology | TARGIT-IORT delivers radiotherapy targeted to the fresh tumour bed exposed immediately after lumpectomy for breast cancer. TARGIT-A trial found TARGIT-IORT to be as effective as whole-breast radiotherapy, with significantly fewer deaths from non–breast cancer causes. This paper documents its worldwide impact and provides interactive tools for clinicians and patients | Observational: retrospective cohort | The TARGIT-A trial was initiated by an academic insight and collaboration with the industry was solely for the development of the device. The study was sponsored by University College London Hospitals (UCLH)/UCL Comprehensive Biomedical Research Centre. Funding was provided by UCLH Charities, National Institute for HealthResearch (NIHR) Health Technology Assessment programme (HTA 07/60/49), Ninewells Cancer Campaign, National Health and Medical Research Council, and German Federal Ministry of Education and Research (BMBF) FKZ 01ZP0508. The infrastructure of the trial operations office in London, UK, was supported by core funding from Cancer Research Campaign (now Cancer Research UK) when the trial was initiated. In the extended follow-up of the TARGIT-A trial (TARGIT-Ex; funded by the HTA programme of the National Institute for Health Research, Department of Health and Social Care in the UK, HTA 14/49/13), we are continuing the follow up of TARGIT-A trial patients in the UK by direct patient contact and via UK national databases. | JV has received a research grant from Photoelectron Corp (1996–99) and from Carl Zeiss for supporting data management at the University of Dundee (Dundee, UK, 2004–2008) and has received honorariums. JV and JT received funding from HTA, NIHR, Department of Health and Social Care for some activities related to the TARGIT trials. MBa was briefly on the scientific advisory board of Carl Zeiss and was paid consultancy fees |
| Vo 2023, USA [JAP] | Reuse of shipping materials in the intravitreal bevacizumab supply chain: feasibility, cost, and environmental impact | Oph | Analyse the feasibility, environmental impact, and cost of reusing shipping materials for intravitreal injection medications vs wasting coolers and cold packs after SU | Observational: cohort | The authors report no financial support for this study | The authors declared no potential conflicts of interest with respect to the research, authorship, and/or publication of this article |
| Winklmair 2023, Austria [JAP] | Potential environmental effect of reducing the variation of disposable materials used for cataract surgery | Oph | 1.Evaluate variability in cataract package composition used throughout Austria. 2. Provide recommendations for more sustainable cataract package compositions and the potentially achievable effect on CO2, reduction. 3. Assess current state of waste separation in Austrian cataract surgery and the possible CO2, reduction | Inventory analysis | Funded by the Austrian Society of Ophthalmology and the Association of Austrian Eye Surgeons | O. Findl is a scientific advisor to Alcon Laboratories,Inc., Beaver-Visitec Intemational, Carl Zeiss Meditec AG, Croma Pharma GmbH, and Johnson & Johnson Vision. M. Amon is a consultant for Alcon Laboratories, inc., Bausch & Lomb, Inc., Geuder AG, Johnson & Johnson Vision, Morcher GmbH, Rayner Intraocular Lenses Lid., and Carl Zeiss Meditec AG. None of the other authors has anyfinancial or proprietary interest in any material or method mentioned |
| Wombwell 2023, Australia [JAP] | Are single-use flexible cystoscopes environmentally sustainable? A lifecycle analysis | Urology | Compare carbon footprint of single-use Ambu® aScope™ 4 Cysto System (Ambu®) with reusable Olympus CYF-VH flexible video-cystoscope (Olympus) | Inventory analysis | The author(s) received no financial support for the research, authorship, and/or publication of this article | The author(s) declared no potential conflicts of interest with respect to the research, authorship, and/or publication of this article |
| Woods 2015, USA [JAP] | Carbon footprint of robotically-assisted laparoscopy, laparoscopy and laparotomy: A comparison | Oncology | Quantify and compare the total greenhouse gas emissions/ ’carbon footprint’ attributable to three surgical modalities | Observational: retrospective database review | NR | The authors declare no potential conflicts of interest |
| Woolen 2023, USA, Germany, Switzerland [JAP] | Ecodesign and Operational Strategies to Reduce the Carbon Footprint of MRI for Energy Cost Savings | Radiology | Determine energy, cost, and carbon savings that could be achieved through different scanner power management strategies | Retrospective cohort | The study was sponsored by an academic-industry partnership. The University of California San Francisco (San Francisco, Ca), Siemens Healthineers (Erlangen, Germany), Siemens USA (Washington, DC), and Siemens Smart Infrastructure (Zug, Switzerland) cosponsored the work. Funding from Siemens was provided to provide MRI machines with power meters and install power monitoring software to collect data from the meters, and funding for investigator effort and data analysis was provided by University of California San Francisco | S.A.W. supported by funding from UCSF, Siemens (grant no. C00236773), and American Roentgen Ray Society ScholarshipAuthors who were not employees of Siemens had complete control of data measurement and analysis and vendor-specific information that might present a potential conflict of interest for authors who were employees of Siemens.S.A.W. Howard S. Stern research grant from the Society of Abdominal Radiology investigating low-field strength prostate MRI in patients with hip implant; grant from Siemens Healthineers. A.E.B. Program manager at UCSF Radiology for institutional collaboration with Siemens Healthineers. A.J.M. Grant from Siemens Healthineers. R.K. Employee of Siemens Healthcare GmbH in Germany and responsible for Sustainability Program of Healthineers Modalities; stock options in Siemens Healthineers. V.L. Employed by Siemens. J.F. Employed by Siemens. C.E. Board membership from Academy of Radiology; Research Board member, University City Science Center; stock options in Siemens Healthineers. C.P.H. No relevant relationships. V.D. Employed by Siemens Medical Solutions; stock in Siemens Medical Solutions |
| Yong 2022, UK [JAP] | Rationalising the use of specimen pots following colorectal polypectomy: a small step towards greener endoscopy | GE | Determine whether combining multiple small colorectal polyps within a single specimen pot reduces carbon footprint without any deleterious clinical impact | Observational: retrospective | The authors have not declared a specific grant for this research from any funding agency in the public, commercial or not-for-profit sectors | None declared |
| Zander 2011, UK [JAP] | Changes in travel-related carbon emissions associated with modernization of services for patients with acute myocardial infarction: a case study | Cardiology | Assess carbon footprint of different healthcare service models for patients with acute STEMI. | Modelling | No external funding or sponsorship was received for this study | The work in part relates to part-fulfilment (dissertation) of the M Phil in Public Health course of the Department of Public Health and Primary Care, University of Cambridge, by A. Zander |
| AT=Air Tamponade, CE=Carbon Emission, CO2=Carbon Dioxide, CT=Computerised Tomography, CTr=Controlled Trial, CTR=Carpel Tunnel Release, ENT=Ear, Nose, Throat, F2F=Face to Face, GA=General Anaesthetic, GC=Gynaecology, GE=Gastroenterology, GHG=Greenhouse Gas, ICU=Intensive Care Unit, JAP=Peer reviewed journal article, LA=Local Anaesthetic, LCA=Life Cycle Assessment, MRI=Magnetic Resonance Imaging, MXR=Mobile X-Ray, NR=Not Reported, Oph=Ophthalmology, O+T=Orthopaedics and Trauma, PMB=Photo-biomodulation therapy, PD=Peritoneal Dialysis, PDSA=Plan Do Study Act, PPE=Personal Protective Equipment, PVC=Polyvinyl Chloride, RCT=Randomized Controlled Trial, STEMI=ST segment elevation myocardial infarction, SU=Single Use, TPU=Thermoplastic polyurethane, TURBT=Trans Urethral Resection of Bladder Tumour, UK=United Kingdom, US=Ultrasound, USA=United States of America, VC=Video/virtual Conferencing, XR=X-Ray | | | | | | |

# References

1. Andrew N, Barraclough KA, Long K, Fazio TN, Holt S, Kanhutu K, et al. Telehealth model of care for routine follow up of renal transplant recipients in a tertiary centre: A case study. Journal of telemedicine and telecare. 2020;26(4):232-8.

2. Arndt E-M, Jansen TR, Bojko J, Roos JJ, Babasiz M, Randau TM, et al. COVID-19 measures as an opportunity to reduce the environmental footprint in orthopaedic and trauma surgery. Frontiers in surgery. 2023;10:959639.

3. Asghari M, Al-e-Hashem S. A green delivery-pickup problem for home hemodialysis machines; sharing economy in distributing scarce resources. Transportation Research Part E-Logistics and Transportation Review. 2020;134.

4. Baboudjian M, Pradere B, Martin N, Gondran-Tellier B, Angerri O, Boucheron T, et al. Life Cycle Assessment of Reusable and Disposable Cystoscopes: A Path to Greener Urological Procedures. European Urology Focus. 2022.

5. Bendine G, Autin F, Fabre B, Bardin O, Rabasco F, Cabanel JM, et al. Haemodialysis therapy and sustainable growth: a corporate experience in France. Nephrology, dialysis, transplantation : official publication of the European Dialysis and Transplant Association - European Renal Association. 2020.

6. Beswick DM, Vashi A, Song Y, Pham R, Holsinger FC, Rayl JD, et al. Consultation via telemedicine and access to operative care for patients with head and neck cancer in a Veterans Health Administration population. Head Neck-J Sci Spec Head Neck. 2016;38(6):925-9.

7. Betts. A GREENER ENDOSCOPY UNIT FOR ROYAL CORNWALL HOSPITAL. 2022 [

8. Bird. Changing the 3 month blood test postage kits for patients on the renal transplant register 2022 [

9. Boberg L, Singh J, Montgomery A, Bentzer P. Environmental impact of single-use, reusable, and mixed trocar systems used for laparoscopic cholecystectomies. PLoS ONE. 2022;17(7 July):e0271601.

10. Bond A, Jones A, Haynes R, Tam M, Denton E, Ballantyne M, et al. Tackling climate change close to home: mobile breast screening as a model. Journal of Health Services Research & Policy. 2009;14(3):165-7.

11. Burton. Sustainable ENT: Fractured Nose Manipulation - Local Anaesthetic Pathway. 2022 [

12. Buttner L, Posch H, Auer T, Jonczyk M, Fehrenbach U, Hamm B, et al. Switching off for future-Cost estimate and a simple approach to improving the ecological footprint of radiological departments. European Journal of Radiology Open. 2021;8:100320.

13. Chambrin C, de Souza S, Gariel C, Chassard D, Bouvet L. Association Between Anesthesia Provider Education and Carbon Footprint Related to the Use of Inhaled Halogenated Anesthetics. Anesthesia and Analgesia. 2023;136(1):101-10.

14. Chan. Reducing carbon (CO2E) waste from pulse lavage systems used in joint replacement surgery, orthopaedic theaters 2023 [

15. Chen M, Zhou R, Du C, Meng F, Wang Y, Wu L, et al. The carbon footprints of home and in-center peritoneal dialysis in China. International Urology and Nephrology. 2017;49(2):337-43.

16. Cheung R, Ito E, Lopez M, Rubinstein E, Keller H, Cheung F, et al. Evaluating the Short-term Environmental and Clinical Effects of a Radiation Oncology Department's Response to the COVID-19 Pandemic. International Journal of Radiation Oncology Biology Physics. 2023;115(1):39-47.

17. Chuter R, Stanford-Edwards C, Cummings J, Taylor C, Lowe G, Holden E, et al. Towards estimating the carbon footprint of external beam radiotherapy. Phys Medica. 2023;112:8.

18. Connor A, Lillywhite R, Cooke MW. The carbon footprints of home and in-center maintenance hemodialysis in the United Kingdom. Hemodialysis International. 2011;15(1):39-51.

19. Connor A, Mortimer F, Higgins R. The follow-up of renal transplant recipients by telephone consultation: Three years experience from a single UK renal unit. Clinical Medicine, Journal of the Royal College of Physicians of London. 2011;11(3):242-6.

20. Connor MJ, Miah S, Edison M, Brittain J, Kondjin Smith M, Hanna M, et al. Clinical, fiscal and environmental benefits of a specialist led virtual ureteric colic clinic: a report of a prospective study. BJU international. 2019.

21. Coombs NJ, Coombs JM, Vaidya UJ, Singer J, Bulsara M, Tobias JS, et al. Environmental and social benefits of the targeted intraoperative radiotherapy for breast cancer: Data from UK TARGIT-A trial centres and two UK NHS hospitals offering TARGIT IORT. BMJ Open. 2016;6(5):e010703.

22. Cooper. Exploring the Impact and Acceptance of

Wearable Sensor Technology for Pre- and Postoperative Rehabilitation in Knee

Replacement Patients: A U.K.-Based Pilot Study. 2022.

23. Cooper DM, Bhuskute N, Hepworth C, Walsh G. The Economic Impact of a Pilot Digital Day-Case Pathway for Knee Arthroplasty in a U.K. Setting. JB & JS open access. 2023;8(1).

24. Croghan SM, Rohan P, Considine S, Salloum A, Smyth L, Ahmad I, et al. Time, cost and carbon-efficiency: a silver of COVID era virtual urology clinics? Annals of the Royal College of Surgeons of England. 2021;103(8):599-603.

25. Curtis A, Parwaiz H, Winkworth C, Sweeting L, Pallant L, Davoudi K, et al. Remote Clinics During Coronavirus Disease 2019: Lessons for a Sustainable Future. Cureus. 2021;13(3):e14114.

26. Davis NF, McGrath S, Quinlan M, Jack G, Lawrentschuk N, Bolton DM. Carbon footprint in flexible ureteroscopy: A comparative study on the environmental impact of reusable and single-use ureteroscopes. Journal of Endourology. 2018;32(3):214-7.

27. de Preux L, Rizmie D. Beyond financial efficiency to support environmental sustainability in economic evaluations. Future Healthcare Journal. 2018;5(2):103-7.

28. de Ridder EF, Friedericy HJ, van der Eijk AC, Dankelman J, Jansen FW. A New Method to Improve the Environmental Sustainability of the Operating Room: Healthcare Sustainability Mode and Effect Analysis (HSMEA). Sustainability. 2022;14(21).

29. Dorrian C, Ferguson J, Ah-See K, Barr C, Lalla K, van der Pol M, et al. Head and neck cancer assessment by flexible endoscopy and telemedicine. Journal of Telemedicine and Telecare. 2009;15(3):118-21.

30. Field RR, Calderon M-DC, Ronilo SM, Ma M, Maxwell H, Mensah P, et al. Environmental and Economic Impact of Using a Higher Efficiency Ventilator and Vaporizer During Surgery Under General Anesthesia: A Randomized Controlled Prospective Cohort. Cureus. 2023;15(5):e39534.

31. Filfilan A, Anract J, Chartier-Kastler E, Parra J, Vaessen C, de La Taille A, et al. Positive environmental impact of remote teleconsultation in urology during the COVID-19 pandemic in a highly populated area. Progres En Urologie. 2021;31(16):1133-8.

32. Forner D, Purcell C, Taylor V, Noel CW, Pan L, Rigby MH, et al. Carbon footprint reduction associated with a surgical outreach clinic. Journal of Otolaryngology-Head & Neck Surgery. 2021;50(1).

33. Frick MA, Baniel CC, Qu V, Hui C, Brown E, Chang DT, et al. Effect of Radiation Schedule on Transportation-Related Carbon Emissions: A Case Study in Rectal Cancer. Advances in Radiation Oncology. 2023;8(5):101253.

34. Fuschi A, Pastore AL, Al Salhi Y, Martoccia A, De Nunzio C, Tema G, et al. The impact of radical prostatectomy on global climate: a prospective multicentre study comparing laparoscopic versus robotic surgery. Prostate Cancer and Prostatic Diseases. 2023.

35. Hardy. Review of the haemodialysis process in a single satellite dialysis unit with the aim to reduce carbon and waste 2022 [

36. Heye T, Meyer MT, Merkle EM, Vosshenrich J. Turn It Off! A Simple Method to Save Energy and CO2 Emissions in a Hospital Setting with Focus on Radiology by Monitoring Nonproductive Energy-consuming Devices. Radiology. 2023;307(4):e230162.

37. Hogan D, Rauf H, Kinnear N, Hennessey DB. The Carbon Footprint of Single-Use Flexible Cystoscopes Compared with Reusable Cystoscopes. Journal of Endourology. 2022;36(11):1460-4.

38. Holmner A, Ebi KL, Lazuardi L, Nilsson M. Carbon Footprint of Telemedicine Solutions - Unexplored Opportunity for Reducing Carbon Emissions in the Health Sector. Plos One. 2014;9(9).

39. Jiang CY, Strohbehn GW, Dedinsky RM, Raupp SM, Pannecouk BM, Yentz SE, et al. Teleoncology for Veterans: High Patient Satisfaction Coupled With Positive Financial and Environmental Impacts. JCO Oncology Practice. 2021;17(9):E1362-E74.

40. Kemble JP, Winoker JS, Patel SH, Su ZT, Matlaga BR, Potretzke AM, et al. Environmental impact of single-use and reusable flexible cystoscopes. BJU International. 2023;131(5):617-22.

41. King J, Poo SX, El-Sayed A, Kabir M, Hiner G, Olabinan O, et al. Towards NHS Zero: greener gastroenterology and the impact of virtual clinics on carbon emissions and patient outcomes. A multisite, observational, cross-sectional study. Frontline Gastroenterology. 2022.

42. Klein H-M. A New Approach to the Improvement of Energy Efficiency in Radiology Practices. Ein neuer Ansatz zur Verbesserung der Energieeffizienz in radiologischen Versorgungseinheiten. 2023;195(5):416-25.

43. Kodumuri. Reducing the carbon footprint in carpal tunnel surgery inside the operating room with a lean and green model: a comparative study. . 2022.

44. Kodumuri P, Jesudason EP, Lees V. Reducing the carbon footprint in carpal tunnel surgery inside the operating room with a lean and green model: a comparative study. The Journal of hand surgery, European volume. 2023:17531934231176952.

45. Lambert P, Musto G, Thiessen M, Czaykowski P, Decker K. Impact of Cancer-Related Virtual Visits on Travel Distance, Travel Time, and Carbon Dioxide (CO2) Emissions during the COVID-19 Pandemic in Manitoba, Canada. Curr Oncol. 2023;30(7):5973-83.

46. Langstaff. Photobiomodulation Therapy (PBM): Using light therapy for oral mucositis, 2023 [

47. Le NNT, Hernandez LV, Vakil N, Guda N, Patnode C, Jolliet O. Environmental and health outcomes of single-use versus reusable duodenoscopes. Gastrointestinal Endoscopy. 2022;96(6):1002-8.

48. Leapman MS, Thiel CL, Gordon IO, Nolte AC, Perecman A, Loeb S, et al. Environmental Impact of Prostate Magnetic Resonance Imaging and Transrectal Ultrasound Guided Prostate Biopsy. European Urology. 2023;83(5):463-71.

49. Leiden A, Cerdas F, Noriega D, Beyerlein J, Herrmann C. Life cycle assessment of a disposable and a reusable surgery instrument set for spinal fusion surgeries. Resources, Conservation and Recycling. 2020;156:104704.

50. Lewis D, Tranter G, Axford AT. Use of videoconferencing in Wales to reduce carbon dioxide emissions, travel costs and time. J Telemed Telecare. 2009;15(3):137-8.

51. Lopez-Munoz P, Martin-Cabezuelo R, Lorenzo-Zuniga V, Vilarino-Feltrer G, Tort-Ausina I, Vidaurre A, et al. Life cycle assessment of routinely used endoscopic instruments and simple intervention to reduce our environmental impact. Gut. 2023:E329544.

52. Materacki. Scoping for change - adopting greener practice in endoscopy in Gloucestershire, Endoscopy team 2023 [

53. McAlister S, McGain F, Petersen M, Story D, Charlesworth K, Ison G, et al. The carbon footprint of hospital diagnostic imaging in Australia. The Lancet Regional Health - Western Pacific. 2022;24:100459.

54. McCarthy CJ, Gerstenmaier JF, O' Neill AC, McEvoy SH, Hegarty C, Heffernan EJ. "EcoRadiology"--pulling the plug on wasted energy in the radiology department. Academic radiology. 2014;21(12):1563-6.

55. McLachlan A, Aldridge C, Morgan M, Lund M, Gabriel R, Malez V. An NP-led pilot telehealth programme to facilitate guideline-directed medical therapy for heart failure with reduced ejection fraction during the COVID-19 pandemic. N Z Med J. 2021;134(1538):77-88.

56. Meiklejohn DA, Khan ZH, Nunez KM, Imhof L, Osmani S, Benavidez AC, et al. Environmental Impact of Adult Tonsillectomy: Life Cycle Assessment and Cost Comparison of Techniques. The Laryngoscope. 2023.

57. Miah S, Dunford C, Edison M, Eldred-Evans D, Gan C, Shah TT, et al. A prospective clinical, cost and environmental analysis of a clinician-led virtual urology clinic. Ann R Coll Surg Engl. 2019;101(1):30-4.

58. Milne. Green Nephrology: Retro-fit of heat cxchangers to haemodialysis machines - Case study and how-to guide: he Campaign for Greener Healthcare; 2010 [

59. Milne. Retro-fit of Heat Exchangers to Haemodialysis Machines - Case Study and How to Guide. : The Campaign for Greener Healthcare; 2023 [

60. Mojdehbakhsh RP, Rose S, Peterson M, Rice L, Spencer R. A quality improvement pathway to rapidly increase telemedicine services in a gynecologic oncology clinic during the COVID-19 pandemic with patient satisfaction scores and environmental impact. Gynecologic Oncology Reports. 2021;36:100708.

61. Moussa G, Andreatta W, Ch'Ng SW, Ziaei H, Jalil A, Patton N, et al. Environmental effect of air versus gas tamponade in the management of rhegmatogenous retinal detachment VR surgery: A multicentre study of 3,239 patients. PLoS ONE. 2022;17(1 January):e0263009.

62. Moussa G, Ch'ng SW, Park DY, Ziaei H, Jalil A, Patton N, et al. Environmental effect of fluorinated gases in vitreoretinal surgery: a multicenter study of 4,877 patients. American journal of ophthalmology. 2021.

63. Muschol J, Heinrich M, Heiss C, Hernandez AM, Knapp G, Repp H, et al. Economic and Environmental Impact of Digital Health App Video Consultations in Follow-up Care for Patients in Orthopedic and Trauma Surgery in Germany: Randomized Controlled Trial. J Med Internet Res. 2022;24(11):11.

64. Natale J, Pascoe J, Horn C, Coode-Bate J, Dickinson A. Teleconsultation versus traditional clinical assessment of patients undergoing circumcision: A retrospective cohort study. Journal of Clinical Urology. 2022.

65. Neves JAC, Roseira J, Queiros P, Sousa HT, Pellino G, Cunha MF. Targeted intervention to achieve waste reduction in gastrointestinal endoscopy. Gut. 2022.

66. Nielson. Pioneering Early Mobilisation in a Cardiac Intensive Care unit: a Sustainable Healthcare Initiative. . 2022.

67. Owens. COLLABORATIVE REPORT OF PAPER LITE AND CONTRAST RECYCLING PROJECTS,

ENDOSCOPY TEAM. 2023.

68. Patel KB, Gonzalez BD, Turner K, Alishahi Tabriz A, Rollison DE, Robinson E, et al. Estimated Carbon Emissions Savings With Shifts From In-Person Visits to Telemedicine for Patients With Cancer. JAMA network open. 2023;6(1):e2253788.

69. Phull M, Begum H, John JB, van Hove M, McGrath J, O'Flynn K, et al. Potential Carbon Savings with Day-case Compared to Inpatient Transurethral Resection of Bladder Tumour Surgery in England: A Retrospective Observational Study Using Administrative Data. European Urology Open Science. 2023;52:44-50.

70. Richards JD, Stoddart M, Bolland B. Virtual Arthroplasty Follow-Up: Better for the Trust, Patients, and the Planet. Cureus. 2022;14(11):e31978.

71. Rizan C, Bhutta MF. Environmental impact and life cycle financial cost of hybrid (reusable/single-use) instruments versus single-use equivalents in laparoscopic cholecystectomy. Surgical Endoscopy. 2022;36(6):4067-78.

72. Rouviere N, Chkair S, Auger F, Alovisetti C, Bernard MJ, Cuvillon P, et al. Ecoresponsible actions in operating rooms: A health ecological and economic evaluation. International Journal of Surgery. 2022;101:106637.

73. Sanchez SA, Eckelman MJ, Sherman JD. Environmental and economic comparison of reusable and disposable blood pressure cuffs in multiple clinical settings. Resources, Conservation and Recycling. 2020;155:104643.

74. Schulte A, Maga D, Thonemann N. Combining Life Cycle Assessment and Circularity Assessment to Analyze Environmental Impacts of the Medical Remanufacturing of Electrophysiology Catheters. Sustainability. 2021;13(2).

75. Sherman JD, Raibley LAt, Eckelman MJ. Life Cycle Assessment and Costing Methods for Device Procurement: Comparing Reusable and Single-Use Disposable Laryngoscopes. Anesthesia and analgesia. 2018;127(2):434-43.

76. Sillcox R, Blaustein M, Khandelwal S, Bryant MK, Zhu J, Chen JY. Telemedicine Use Decreases the Carbon Footprint of the Bariatric Surgery Preoperative Evaluation. Obesity Surgery. 2023.

77. Sillcox R, Gitonga B, Meiklejohn DA, Wright AS, Oelschlager BK, Bryant MK, et al. The environmental impact of surgical telemedicine: life cycle assessment of virtual vs. in-person preoperative evaluations for benign foregut disease. Surgical Endoscopy and Other Interventional Techniques. 2023.

78. Sorensen. Comparative Study on Environmental Impacts of Reusable and Single-Use Bronchoscopes. . 2018.

79. Stripple H, Westman R, Holm D. Development and environmental improvements of plastics for hydrophilic catheters in medical care: an environmental evaluation. Journal of Cleaner Production. 2008;16(16):1764-76.

80. Thiel CL, Eckelman M, Guido R, Huddleston M, Landis AE, Sherman J, et al. Environmental impacts of surgical procedures: Life cycle assessment of hysterectomy in the United States. Environmental Science and Technology. 2015;49(3):1779-86.

81. Thiel CL, Mehta N, Sejo CS, Qureshi L, Moyer M, Valentino V, et al. Telemedicine and the environment: life cycle environmental emissions from in-person and virtual clinic visits. Npj Digital Medicine. 2023;6(1).

82. Thiel CL, Woods NC, Bilec MM. Strategies to Reduce Greenhouse Gas Emissions from Laparoscopic Surgery. American journal of public health. 2018;108(S2):S158-S64.

83. Thota R, Gill DM, Brant JL, Yeatman TJ, Haslem DS. Telehealth Is a Sustainable Population Health Strategy to Lower Costs and Increase Quality of Health Care in Rural Utah. JCO oncology practice. 2020;16(7):e557-e62.

84. Tselapedi-Sekeitto. Telemedicine as an environmental ally - The social, financial, and environmental impact of virtual care in the otolaryngology clinic. . Am J Otolaryngol. 2023;44(2).

85. Udayaraj UP, Watson O, Ben-Shlomo Y, Langdon M, Anderson K, Power A, et al. Establishing a tele-clinic service for kidney transplant recipients through a patient-codesigned quality improvement project. BMJ open quality. 2019;8(2):e000427.

86. Vaidya JS, Vaidya UJ, Baum M, Bulsara MK, Joseph D, Tobias JS. Global adoption of single-shot targeted intraoperative radiotherapy (TARGIT-IORT) for breast cancer-better for patients, better for healthcare systems. Frontiers in Oncology. 2022;12:786515.

87. Vo LV, Mastrorilli V, Muto AJ, Emerson GG. Reuse of shipping materials in the intravitreal bevacizumab supply chain: feasibility, cost, and environmental impact. International Journal of Retina and Vitreous. 2023;9(1):34.

88. Winklmair N, Kieselbach G, Bopp J, Amon M, Findl O. Potential environmental effect of reducing the variation of disposable materials used for cataract surgery. Journal of Cataract and Refractive Surgery. 2023;49(6):628-34.

89. Wombwell A, Holmes A, Grills R. Are single-use flexible cystoscopes environmentally sustainable? A lifecycle analysis. Journal of Clinical Urology. 2023.

90. Woods DL, McAndrew T, Nevadunsky N, Hou JY, Goldberg G, Yi-Shin Kuo D, et al. Carbon footprint of robotically-assisted laparoscopy, laparoscopy and laparotomy: A comparison. International Journal of Medical Robotics and Computer Assisted Surgery. 2015;11(4):406-12.

91. Woolen SA, Becker AE, Martin AJ, Knoerl R, Lam V, Folsom J, et al. Ecodesign and Operational Strategies to Reduce the Carbon Footprint of MRI for Energy Cost Savings. Radiology. 2023;307(4):e230441.

92. Yong KK, He Y, Cheung HCA, Sriskandarajah R, Jenkins W, Goldin R, et al. Rationalising the use of specimen pots following colorectal polypectomy: a small step towards greener endoscopy. Frontline Gastroenterology. 2022.

93. Zander A, Niggebrugge A, Pencheon D, Lyratzopoulos G. Changes in travel-related carbon emissions associated with modernization of services for patients with acute myocardial infarction: A case study. Journal of Public Health. 2011;33(2):272-9.
