# Supplementary material for: Effectiveness of Interventions to Reduce Carbon‐Emissions Within Secondary Healthcare: Systematic Review and Evidence and Gap Map
Source: Campbell Syst Rev. 2025 Dec 23;21(4):e70077. doi: 10.1002/cl2.70077 (PMC12723626; doi:10.1002/cl2.70077)
Supplement: Supplementary file 2 — NetZero Supp Materials 2 copy. [file CL2-21-e70077-s002.docx]

# Supplementary Materials 2: Methods for calculating carbon emissions - LCA studies

| **Study** | **Use of a lifecycle informed perspective** | **LCA or Inventory analysis methods** | **Standards / reference data informing carbon emission model (including dates)** | **Method of collecting/calculating carbon emission data** | **Method of analysis** | **Functional Unit (as stated in paper)** | **Boundaries of system being studied** | **Stages covered by system** | **Statement of representativeness of data** |
| --- | --- | --- | --- | --- | --- | --- | --- | --- | --- |
| Baboudijian 2023 | Y | LCA | The LCA of consumables used during the reprocessing of reusable cystoscopes was evaluated using generic data from the Ecoinvent v3.5 database. The life cycle inventory of the Scope was provided by the manufacturer (these data, particularly the raw materials used, are not developed in the study due to company confidentiality rules). | For reusable scopes, an internal audit was conducted to quantify and characterise the products and materials used in the reprocessing of reusable cystoscopes. For both scenarios (reprocessing consumables and aScope), transportation distances of devices to health care facilities were estimated at 500 km. After reaching the end of their useful lives, both single-use cystoscopes and consumables, including their packaging, entered waste management and were burned entirely. Waste management modelling was performed using the French model.^a^ The main environmental impact category studied was climate change caused by greenhouse gas emissions. Four other standard environmental and human health impact categories were considered: mineral resource depletion, ecotoxicity, acidification, and eutrophication. Carbon impact category calculation: Climate change (kgCO2 eq). The calculation method used for this indicator was based on the results of the work of the Intergovernmental Panel on Climate Change, which evaluated the global warming potential of several substances that may be emitted to the air. For the same substance, global warming potentials can vary according to the time horizon considered. The authors chose radiative forcing with a 100-yr horizon, which is the most commonly used time scale in life cycle analyses. Data for each impact category were reported as a mean value. | Simapro v9.3.3 (Amersfoort, the Netherlands). A Monte Carlo analysis random number sampling, was used to account for the inherent uncertainty in life cycle inventory data and the variability in material and energy consumption for each type of flexible cystoscope, enabling a better understanding of the range of potential environmental impacts from a typical procedure. For all impact categories, the standard deviation and 95% confidence intervals, calculated using the Monte Carlo analysis, were presented alongside the mean values. | The functional unit corresponded to the specific set for high-level disinfection, including four pairs of nitrile gloves, one pair of neoprene gloves, an apron, a wipe, a soft brush, a sterile swab, three sterile syringes, two transport (Hanova) bags, a camera cover, a sterile cover, three peracetic acid canisters, and a sterile water canister. | For the aScope, the complete lifespan of the scope was evaluated, including raw material, material formulation, component production, product assembly, distribution, transportation after use, and final disposal. For reusable cystoscopes, raw material extraction, production and assembly, distribution, maintenance and repair, collection, and end-of-life treatment were not taken into account due to the lack of specific data provided by companies; therefore, the evaluation was limited to their reprocessing and sterilisation using a model consisting of standard high-level disinfection with peracetic acid. | 'Cradle-to-grave' life cycle analysis - extraction, production, distribution, transportation after use, final disposal, and reprocessing and sterilisation. | **Geographical:** For both scenarios (reprocessing consumables and aScope), transportation distances of devices to health care facilities were estimated at 500 km. **Temporal:** The authors chose radiative forcing with a 100-yr horizon, which is the most commonly used time scale in life cycle analyses. **Technical:** Limited to flexible cystoscopes used within cystoscopy. The life cycle of a single-use cystoscope may vary among manufacturers, and the results cannot be transposed to all commercially available flexible disposable cystoscopes, or to rigid cystoscopes. |
| Boberg 2022 | Y | LCA | LCA was in accordance with the international standard ISO 14044 guideline for conducting an LCA. Data collection included reference to the database ecoinvent v3.6, ecoinvent Centre. | Landskrona Hospital was used as the index hospital in the analysis and all hospital related data, such as product storage information, the model of autoclave and washer disinfector, and waste treatment practice was collected from this institution. Data describing the specifics of the trocars, processes at the hospital and the hospital’s waste management system were collected using questionnaires sent to the practitioners, the trocar distributors or manufacturers, and the supplier of sterilisation machines. Data on some of the plastic materials were neither provided by the manufacturer nor included in the ecoinvent v3.6 database. To assess the impact of assumptions concerning these materials, the authors performed two sensitivity analyses in which all plastic materials were assumed to be the common plastics polycarbonate or high-density polyethylene. In the sterilisation process, trocars in the reusable system represented 2% of a fully loaded autoclave and about 8% of a fully loaded washer-disinfector, and inputs to SimaPro were allocated based on the trocars share of a fully loaded machine. In the mixed system the reusable trocars represented 1.5% of a fully loaded autoclave and around 6% of a fully loaded washer-disinfector. To assess a situation in which the machines were not fully loaded, the authors increased the allocation by two and five times the original allocation in sensitivity analyses. Transportation distances for the trocars from the manufacturer (Ireland, GB, Germany, Netherlands) to Landskrona (Sweden) were estimated using Google Maps, assuming use of the fastest route. Transport by road was modelled as a lorry weighing 16–32 metric tons with a Euro Class 5 engine which represents a large share of the European transportation fleet. Transport by boat was modelled as freight by sea on ferry. Transport from raw material supplier to manufacturer was not modelled as the raw material impacts include average global transport. One of the manufacturers had air freight as an alternative and so a sensitivity analysis was performed using air freight as main mode of transportation. The individual trocar package for new trocars was modelled based on data from the manufacturer of the trocars in the reusable trocar system. The sterilisation wrap, used in the sterilisation process and for the storage of the reusable trocars in between surgeries was modelled based on information from the index hospital. The sterilisation process was modelled with water from well (tap water), deionized water, detergent (alkylbenzene sulfonate), average wastewater treatment for Europe, and a Swedish, mainly renewable, electricity-mix consisting of 39% hydropower, 39% nuclear power, 12% wind, 10% thermal impact of the electricity-mix we performed two sensitivity analyses using either a largely coal dependent electricity mix as exemplified by the Polish electricity-mix consisting of 72% coal and oil, 16% natural gas, 10% renewable or a European standard market electricity mix consisting of 46% coal and oil, 25% natural gas, 13% renewable energy, 12% nuclear, 3% hydropower. At the end-of-life, all trocars were assumed to be incinerated whereas paper and plastics from instrument packaging and sterilisation wraps were considered to be recycled. | Monte Carlo simulations were used to estimate differences between trocar systems. Monte Carlo simulation, which uses randomly selected input data within the uncertainty range for each parameter in the model, was used to simulate the 2.5th and 97.5th percentiles (iterations = 1000). Monte Carlo simulations were performed using SimaPro software version 9.1.1.1. Inferential statistics were not used to compare the systems. Instead, dependent (paired) simulation in the Monte Carlo runs was used to assess the certainty of differences between the products. This means that the same sample of input data was used for shared processes in the different systems. Data were presented as median and the 2.5th to 97.5th percentiles. Differences between the systems for which the 2.5th to 97.5th percentiles did not cross 0 were considered to reflect true differences. The environmental impacts on 15 midpoint impact categories were estimated: mineral extraction, non-renewable energy, global warming, aquatic eutrophication, aquatic acidification, land occupation, terrestrial acidification and nutrification, terrestrial ecotoxicity, aquatic ecotoxicity, respiratory organics, ozone layer depletion, ionizing radiation, respiratory inorganics, non-carcinogens, and carcinogens. The integrated downstream effect of these impacts was characterised into the four endpoint categories resources, climate change, ecosystem quality, and human health using the IMPACT 2002+ methodology to get a holistic understanding of the overall environmental impact of the different product systems. The unit for the resource endpoint is MJ Primary, referring to the total amount of extracted non-renewable energy. The unit for the climate change endpoint is kg CO2eq 100, referring to the climate effect of CO2 emitted into the air over 100 years. The unit for ecosystem quality was PDF m2 yr, referring to potentially disappeared fraction of species over a certain area during a certain time. The unit for human health was DALY, referring to disability adjusted life years per person and year. | 500 laparoscopic cholecystectomies (based on one large reusable trocar being used approximately 500 times during its ten-year lifetime at Landskrona Hospital). As reusable trocars can break and have a shorter lifetime, or, in some cases be used more than 500 times, two sensitivity analyses were performed where the functional unit was changed to 250 and 750 surgeries. | System boundaries for both single-use and reusable trocars were set to include raw material and fibre production, for both the production of trocars and the production of their packaging. Waste scenarios and energy savings due to the recycling of packaging materials were included in the assessment. Similarly, transport from the manufacturer to the hospital and from hospital to waste management facilities were included. Only environmental impact directly connected to the products’ life cycles was accounted for. Material and processes needed to produce the machines used in sterilisation process (autoclave and washer-disinfector) fell outside the system boundaries. | 'Cradle to grave' - extraction of raw material, manufacturing, the use phase, and waste management. | **Geographical:** Swedish setting. **Temporal:** The unit for the climate change endpoint is kg CO2eq 100, referring to the climate effect of CO2 emitted into the air over 100 years. **Technical:** Validity of study findings is uncertain for systems containing trocars other than those analysed in this study. |
| Chuter 2023 | y | Inventory analysis | Research papers. Climate Change 2013: The Physical Science Basis. Contribution of Working Group I to the Fifth Assessment Report of the Department of Business, Energy and Industrial Strategy 2020-2021 | **Patient travel:** To estimate emissions resulting from patient travel centres 1, 2 and 4, postcodes from patient records were extracted and all travel was assumed to be by car. A route planning tool was used to estimate a realistic road travel route, based on average off-peak driving. Centre 3 used a slightly different methodology, where a patient questionnaire of 99 patients was used instead of patient notes to estimate the distance travelled by patients. For comparison with other centres emissions from patient travel by car was calculated, but mode of transport was also used to calculate travel emissions in this urban area**. Linac energy consumption:** direct measurements were made at Centre 1 using preinstalled power meters on three linacs. The power meters displayed a running total of energy used by the linac, and so by monitoring this value before and after delivering a patient treatment, the energy used could be calculated. The power consumption was measured for 20 prostate and 20 breast patients. For Centre 1 the measured treatment power was also compared to the theoretical treatment power based on a manufacturer value of 18 kVA and 0.9 power factor, giving 16.2 kW. This value was multiplied by the beam-on time to give the power in kWh for each patient and the difference between this and the measured value calculated. As linacs also consume energy when in an idle state (i.e. when they are not treating), idle energy measurements were also taken using the same power meters used for the treatment power measurement. It was assumed that the linacs were turned on at 6 am and turned off at 8 pm, with only two out of the 10 linacs on between these times at weekends. The average value was subtracted from treatment energy measurements. To ensure that idle energy use was still accounted for in overall emissions, annual idle energy use was calculated and split equally among all fractions delivered annually, resulting in an additional emission value per fraction. Some variability between different linacs was to be expected, so measurements were taken across all three available linacs at Centre 1, with a sub-sample being repeated on all three to ensure they were comparable with one another. **Power consumption:** Centres 3 and 4 used a Fluke 1738 3-phase power logger (Everett, Washington, USA) to collect linac energy data. The same portable device was used at both centres which samples data at 10.24 kHz recording every second for each power phase. An energy study was conducted with current loops and voltage probes attached to each of the 3 phases and a neutral. Fluke Energy Analyse Plus software was used to extract summed data over all three phases, including the average apparent power and average apparent energy per second. Apparent power and energy values were analysed instead of active power and energy, so the full demand from the utility is used in calculations, therefore no ‘power factor’ has been taken into account in calculations. This data logger was attached to a Varian TrueBeam in Centre 3 for one week, and to an Elekta Versa HD in Centre 4 for one week. The apparent power data over the week were used to compare to manufacture published information. The apparent energy was used to calculate the energy consumed during one fraction of a conventional prostate treatment through time correlation of Fluke data and treatment data, identifying 20 prostate patients in each centre for analysis. The time selected per treatment was approximated based on beam on/off times, not the treatment appointment time slot which would be longer than this to allow for patient set up and imaging. The energy per fraction was then scaled to the treatment course and BEIS conversion factors for energy use on the national grid were used to convert to emissions. As the linac still consumes power in an idle state a fraction of this should be considered for every patient’s treatment as done in Centre 1. The idle power for each fraction was also removed from the measured treatment energy to indicate the additional energy required for a treatment. This was performed by calculating the energy consumed over the ‘treatment’ time slot using the average measured apparent power in the idle state. **Pre-treatment imaging:** Patient notes were used to identify the number of pre-treatment scans (CT and MRI) that patients received. Values for energy use per scan taken from literature were then used alongside BEIS conversion factors for energy use of the national electricity grid to convert to CO2e emissions. Sulphur hexaflouride: To quantify the emissions due to SF6 lost from linacs at centre 1, periodic weight measurements of SF6 tanks during routine maintenance of linacs were utilised. These tanks are used to re-pressurise SF6 within the feeding waveguide of linacs, which takes place during routine maintenance. By analysing the lost mass of SF6 in the container over time, the rate of SF6 leakage from the linac could then be estimated. Data for ten linacs were available with between 9 and 3 years of measurements for each linac, taken from engineering service records. A weighted average of leakage data to account for different number of fractions treated on each linac was then taken and then leakage split equally among all fractions delivered annually, resulting in a leakage value per fraction. **Personal protective equipment:** Emission contributions from the use of PPE were quantified using values taken from literature. The types of PPE considered included face masks, surgical gloves and single use aprons. Estimated numbers were provided by radiographers, determining how many of each item were used per radiographer and per patient. | **Conversion factors:** Miles travelled - Average petrol car, 0.281 kgCO2e/mile [14] and public transport, 0.0754 kgCO2e/mile. Number of CT/MRI scans: 1.2 kWh for a CT and 19.9 kWh for an MRI [7]. UK electricity grid, 0.233 kgCO2e/kWh. **Linac energy-treatment:** UK electricity grid, 0.233 kgCO2e/kWh. **Linac power-idle:** UK electricity grid,  0.233 kgCO2e/kWh. SF6 leakage: IPCC GWP100 = 23,507. **PPE:** Gloves = 0.026 kg | One completed treatment course for one patient, with the carbon footprint (unless otherwise stated) being  reported per patient. | The point of referral for radiotherapy until the first follow up appointment and included the following aspects of the RT pathway for The Christie (Centre 1, main centre): (i) patient travel to and from the hospital, (ii) pre-treatment imaging (CT and MRI), (iii) energy used by the linac during treatment and when idle between treatments, (iv) SF6 gas leakage and personal protective equipment. | Use/reuse, patient travel, PPE waste. | **Technical:** travel contribution to CE was based on based on assumptions on the mode of transport and travel route. It was assumed that all patients travelled by an average petrol car meaning that this is not an accurate measurement of the true carbon footprint of travel. Centre 3 used a different approach to estimating the distance travelled by patients (questionnaire) which enabled a more accurate determination of the carbon footprint of patient travel. It was also assumed that they travelled from home rather than from work. As all four centres had different geographical patient catchment areas, there would be a large difference in the use of public and active transport, leading to variation in the carbon footprint of travel. **Power consumption of the linacs:** Values for energy use per scan were taken from literature (Heye et al, 2020) and were then used alongside BEIS conversion factors for energy use of the national electricity grid to convert to CO2e emissions. The apparent power data over the week were used to compare to manufactured published information. Previously literature had used these values for calculations of power in different machine states. A recent measurement of linac power has been performed through measuring the power used per MU. However, this did not capture power in different states and assumptions were made for the power factor. This is therefore the first publication with detailed linac power values in a clinical setting. |
| Connor 2011a | Y | Component analysis | Followed principles and definitions defined within the Publicly Available Specification for the measurement of GHG emissions from goods and services (PAS2050). Department for Transport. National travel survey: 2006.(1)** The Health and Social Care Information Centre, National Kidney Care Audit Patient Transport Survey Report.2010. (2)** 2009 Guidelines to Defra/DECC’s GHG Conversion Factors for Company Reporting. (3)** Inventory of Carbon and Energy (ICE) database version 1.6a.(4) Manufacturer information** - Market Review: Home Haemodialysis Services. CEP 10061. March 2010.(5)** Informal reports - Ansell (2008);(6) U.S. Renal Data System. U.S. Renal Data System, USRDS 2007Annual Data Report: Atlas of End-Stage Renal Disease in the United States(7). **. Data provided by Dorset County Hospital Estates Department. | **Assumptions:** an in-centre dialysis facility consists of 15 stations and runs 3 shifts per day to full capacity, thereby providing ICHD to 45 patients each day. All patients receive HD rather than hemodiafiltration, and that no unforeseen clinical or technological complications arise during the provision of HD. **Travel & Transport:** The modality and distance of both outward and return journeys were analysed for 11,211 different patients. Staff travel emissions were apportioned on the basis of the number of patients to whom they would provide care during a working day. | **Electricity consumed by water treatment and storage systems in ICHD facilities:** A volume of purified water is stored for use during ‘‘loop disinfections.’’ The energy required to heat this water from room temperature to, and maintain it at, 60 C, as well as the energy required to heat it from 60 C to 90 C before each disinfection cycle, was calculated using the formula Energy Used=C x M x DT, where: C= specific heat capacity of water (J/g/K); M=the mass of water being heated (g); and DT=the change in temperature required (1K), on the assumption that the process is 100% efficient. The activity data were then apportioned according to the number of HD treatments to which they contribute. | NR: The carbon footprints attributable to the provision of maintenance HD to a single patient.* | Approach (i) individual components of the process of providing repeated treatment sessions of HD (including dialysis access surgery) and, (ii)) the impact of these components in terms of building energy use, travel and transport, and procurement (including waste). **Travel & Transport:** included travel of employees to and from their normal place of work and of ICHD patients to and from point of service use. **Procurement:** as direct and indirect emissions were included, those emissions relating to the procurement of the consumable products required to provide a HD treatment were included; namely, the procurement of medical equipment, paper and office supplies, laundry services, construction, water, sanitation products (including chemicals for the external decontamination of HD machines), and the collection, treatment and disposal of waste. The inclusion of the emissions arising from the installation and maintenance construction work undertaken to provide appropriate dialysis environments was considered necessary to accurately compare the ICHD and HHD modalities. **Universal exclusions:** the following sources of emissions were considered to lie outside of the boundary of this study; the production of machinery used repeatedly; pharmaceuticals; human inputs into the processes; food and beverages; staff and patient training; water other than that used in HD treatments; heating and lighting; business services; and immaterial emissions sources (those anticipated to be <1% of total footprint). **Machinery:** the emissions attributable to the production of HD machines were excluded. The emissions attributable to the production of HD machines were excluded. The emissions related to the consumable items of medical equipment (those with an anticipated life-span of 1 year) used within HD machines were included. Included building energy use, water consumption, only those treatments for which the dialysate had been derived from mains water were included in the determination of water consumption. Because the recycling of domestic waste is not undertaken within the boundary of a HD service, the carbon recovered was realised outside of the boundary of this study. However, the DEFRA emissions factors for recycling to this waste were applied (rather than to consider the disposal of this waste to have no impact upon the overall carbon footprint of HD), in order to maintain consistency in the approach to waste management. The extent of manufacturing waste, and what happened to it (re-use, recycling or disposal), was excluded from this study. | Procurement, use/reuse, travel, waste. | **Technical**: NxStage equipment. Data provided by UK distributors for NxStage. Electricity consumption of HD machines - data measured directly within UK for ICHD, data were recorded for 3 standard HD machines in common use (Fresenius 5008, Fresenius, Sutton-in-Ashfield, UK; Gambro AK200S, Gambro, Huntington, UK; and B-Braun Dialog1machines, B-Braun, Sheffield, UK) and averages taken. For patients using standard HD machines, dialysate flow rates have been assumed to be 300 mL/min for those undertaking nocturnal treatments and 600 mL/min for all other treatments. For each HD modality and regime, the volume of pure water required to provide 1 HD treatment has been determined by measuring the volumes required for each of the stages of simulated treatments using the aforementioned machines. Maintenance construction costs were determined from data provided by Dorset County Hospital Estates Department for ICHD, and from estimated data for HHD. **Geographical:** The electricity emission factors used in this study are based on the United Kingdom grid average mix of different types of generation, and the contribution of energy consumption to the emissions of HD treatments may therefore vary internationally. The source of activity data for patient travel was intended to provide the most accurate model of the travel emissions  of a patient undertaking ICHD in the United Kingdom but significant differences might exist between regions or internationally. |
| Davis 2018 | Y | Inventory analysis | Carbon Emissions Calculator ICAO; ETD4; Stutz (2010) Carbon Footprint of a typical business laptop from Dell;(8) Juerg (2009) Plastic bags and plastic bottles CO2 emissions during their lifetime. Time for change;(9) Steel's contributions to a low carbon future world stell position paper. Africa (Lond) 2014 Dayaratne et al. (2015) Carbon footprint reduction. A critical study of rubber production in small and medium scale enterprises in Sri Lanka. .(10) | Data were obtained on manufacturing of single-use and reusable flexible ureteroscopes, and from typical uses obtained with a reusable scope, including repairs, replacement instruments, and ultimate disposal of either type of ureteroscope. Solid waste generated (kg) and energy consumed (kWh) during each case were quantified and converted into equivalent mass of carbon dioxide (kg of CO2) released. Standardized carbon footprint protocol guidelines were used to determine the mass of CO2/kg emitted during the manufacturing process for single-use and reusable flexible ureteroscopes. The carbon footprint of reusable flexible ureteroscopes was calculated using previously validated models by obtaining data on manufacturing, sterilisation, repackaging, repair and solid waste disposal. Repair costs were calculated as a percentage of the components that failed resulting in reparation of the scope. |  | For reuseable scope: 180 endourologic cases per scope. FU=1kg/180. | NR | Manufacturing of single-use and reusable flexible ureteroscopes, repairs, replacement instruments, and ultimate disposal for reusable scopes. | **Technical:** reusable flexible ureteroscopes typically have 16 uses before repairs are required and approximately 180 uses before decommissioning (life cycle of 180 uses and 11 repairs). Sterilisation machine: Olympus ETD4 - uses 9.2kW per cycle=each cycle takes 70MIN and sterilises 2 scopes= 7.9Kw/hour=7.9kg CO2. |
| de Ridder 2022  Healthcare Sustainability Mode and Effect Analysis | Y | Healthcare Sustainability Mode and Effect Analysis. | Research papers (e.g. DeRosier et al 2022;(11) MacNeil et al, 2017).(12) DEFRA. Guidelines to Defra/DECC’s GHG Conversion Factors for Company Reporting: Methodology Paper for Emission Factors.**(13) | Draws on Failure Mode and Effect Analysis risk assessment tool used in manufacturing industry: purpose is to prospectively identify the ways in which a process may fail (failure mode) and examine their consequences (effect analysis). To apply this method to healthcare processes for improving patient safety, the Healthcare FMEA (HFMEA) was created (DeRosier et al, 2002). The HFMEA is a structured step-by-step method characterised by the following key elements: (i) Multidisciplinary team: subject matter experts, advisor (to keep the team on target) and team leader (skills in group processes); (ii) Flowchart: provides insight into the process, keep the team on track and allow for timely completion; (iii) Hazard analysis: list all possible failure modes for each subprocess, determine their severity and probability and calculate a hazard score; (iv) Decision tree: determine if further action is needed (critical?, control measure available?, detectable hazard?); (v) Triage function: focus on critical and relevant parts of the process. This condenses into 5 analytical steps: 1) topic definition, 2) team assembly, 3) process flowchart, 4) hazard analysis, 5) actions and outcome measures. | From baseline data, hotspots of environmental burden could be identified. In the HSMEA, the environmental hazard of all sub-steps (items that are disposed of) identified in Step 3 were quantified in Step 4 by means of the amount of waste (in kg) produced, and the carbon footprint of the waste stream it was disposed of through (kg CO2 equivalents). In order to calculate the carbon footprint, DEFRA greenhouse gas life cycle conversion factors for waste disposal were used | NR | The DEFRA greenhouse gas life cycle conversion factors for waste disposal take into account greenhouse gas emissions generated both upstream (production, transport) and downstream (transport, disposal). All waste generated during the C-section was not included e.g. anaesthetic waste was not taken into account. Waste from anaesthetic practice contributes substantially to Operating Room waste. | Production, transport*, disposal. | NA |
| Fuschi 2023 | Y | Inventory analysis | Used existing databases or ones developed specifically for the purpose which came from associations, authorities (governments and European Commission) and research bodies. LCA was modelled as per ISO 14044 Guidelines. | The first step was to separate the contributing components into the scope of emission. **Scope 1** emissions involved the CO2 used during the surgical procedures necessary for insufflation. A typical CO2 cylinder used in the operating room contains compressed gas. Using the ideal gas law, 1 mol of any gas occupies 22.4 L at 1 atm. Because 1 mol of CO2 weighs 44 g, there are 1.5∙10-4 metric tons of CO2 in one cylinder. To estimate the operative time per cylinder, the authors calculated the operative times for both robot-assisted and laparoscopic radical prostatectomy procedures. **Scope 2** involves the indirect emissions that result from the generation of electricity, disposal of single-use devices, and heating, cooling, production, and sterilisation of instruments. The environmental impact of instrument manufacturing was approximated using global average metalworking processes for all metal components of instruments, and injection moulding processes for the plastic components of instruments. All reusable components were decontaminated and reused 15 times, with energy and material inputs for decontamination being modelled using data extrapolated from the university department. Raw material composition of both single-use and reusable instruments: as most of the instruments utilised are composites, the plastic, metal, and composite fibre components were weighed to evaluate the CO2 emissions resulting from their production, sterilization, and disposal. All anaesthesiologic materials utilized during the procedures were weighed to calculate the CO2 emissions. The CO2 emissions from electricity usage, single-use device disposal, heating, cooling, and production were calculated considering, for each group, the mean result from the database of patients who underwent minimally invasive radical prostatectomy (Table 1) surgical procedure time, anaesthesia duration, length of stay, days spent in postoperative intensive care unit, and the need for conversion to open surgery. For each procedure, the disposable material and amount of CO2 used was reported, and the quantity of fluids infused and dispersed. Furthermore, all disposable materials were weighed, and the CO2 consumption necessary for disposal was evaluated. Additionally, an estimate of the energy consumption required during the surgical procedure and hospital stay (heating and cooling) was calculated. | Simapro v9.3.3 (Amersfoort, the Netherlands) software was used to perform the LCA. | Surgical procedure* | 'Door to door' analysis. Manufacturing, sterilisation, single-use device disposal, use of anaesthesiologic materials, energy usage and energy consumption during surgical procedure and hospital stay. | Extraction, material formulation, component production, product assembly, distribution. | **Technical:** Estimates of CE were modelled from existing databases or those developed specifically for the purpose. |
| Hogan 2022 | Y | Inventory analysis. This study reviewed the carbon footprint solely and did not address the overall environmental impact, which includes full life cycle analysis assessment of impacts ("flows") to and from nature, before, during, and after product use. It did not report on the volume of toxic waste produced in relation to the cystoscopes themselves. | Environmental Protection Agency (EPA). Inventory of US Greenhouse Gas Emissions and Sinks 1990-2019. Research paper. See Davis 2018 for references for standardised CO2 protocol.(14) Detailed data were obtained from the Ambu research and development team outlining the flexible cystoscope. | Weights were recorded using digital scale (g). Device packaging items disposed in household waste bin were weighed separately to items used in contact with patients disposed in clinical waste. Solid waste generated during each case were calculated and converted into equivalent mass of carbon dioxide (kg CO2) released based on its mode of disposal. To assess CO2/Kg of raw material produced by equipment, a standardised CO2 protocol was followed. This was 6kg of CO2 per kg of plastic, 1.16kg of CO2/kg of rubber, 1.8kg CO2/kg of steel and 150kg of CO2 per kg of electronics. Data from Ambu research on the flexible cystoscope showed that it comprised plastic (92.6%), steel (4%), electronics (1.8%) and rubber (1.6%). The manufacturing cost of a flexible cystoscope was calculated as 8.51kg of CO2 per 1 kg of cystoscope. A calculation was performed based on one imperial tonne (1016kg) of waste incinerated, producing 1246kg of CO2, meaning each 1kg waste incinerated produces 1.23kg of CO2. The CO2 footprint of transport of single use flexible cystoscopes was calculated based on average of 26.5g of CO2 produced per tonne-kilometre in dry freight over 10,791km distance from manufacturing factory in Penang, Malaysia to Cork, Ireland. This amounted to 285.96kg of CO2 per tonne per journey and each 170g cystoscope (including packaging) producing 0.049kg of CO2. | Data analysis was performed using Stata Statistical Software: Release 17. A Shapiro-Wilk test was used to test for normality. All data were non-normally distributed, so Mann-Whitney U test was used to compare median values. Two-sided p-value 0.05 was considered statistically significant. | Per case* | Repairs and maintenance for the reusable cystoscope were not included. | Manufacture, transport, sterilisation, landfill and incineration. | **Geographical:** data were limited to a single centre and may not be similar to the waste produced by other centres for the same procedure. The data on which the carbon footprint outcomes were from online sources, although this was similar to other studies reviewed in this field. |
| Holmner 2014 | Y | Study adopted the form of a simplified, streamlined life cycle inventory, with the aim to evaluate the most important aspects of telemedicine with respect to CO2 emissions. | Used Conference Proceedings, research papers and LCA & Product Carbon Footprint ESPRIMO E9900. | The study builds on results from life cycle inventories on hardware and software required to connect the patient and the specialist, via videoconferencing. The authors largely adopted the strategy described in Ong et al. that takes into account end-point devices, such as computers, monitors, cameras, local area network (LAN) components and video codes used to compress and decompress digital video signals, as well as the costs for Internet traffic, although technically, data is transmitted using the dedicated hospital network.(15) The method was modified to fit the device setups and assumptions on life length and used rates of the equipment. Travel distances were estimated as the distance from the town closest to the patient’s place of residence to Umea, where the university hospital was located. Access to information on the technological set-ups used for individual appointments was only available for the hand and plastic surgery unit, and therefore, a realistic upper and lower emission limit based on the following scenarios were estimated. These scenarios were applied to both clinics. Upper bound scenario: The upper bound calculations were based on the scenario for treatment in the primary health centre, but with the addition of a second 46’’ screen in the videoconference room of the specialist. Lower bound scenario: The lower bound calculations were based on the scenario for treatments provided in the patients’ home; two standard desktop PCs with use rates of 780 hours (patient) and 7,300 hours (specialist) and a bandwidth of 512 kbps. Regarding carbon costs of Internet traffic, the same assumptions apply as for the upper bound scenario to avoid underestimating the carbon costs. The patient's home LAN was likely to be of significantly lower complexity than the hospital LAN and both power consumption and embodied energy costs are likely to be significantly lower. Nonetheless, the authors chose to apply the same estimates as the higher bound scenario, with the only exception that the use rate applied to the patient scenario is 780 hours, similar to the other end-point devices. |  | One hour (typical meeting duration) | Patient travel and patient and staff technology use. | Transportation after use, and final disposal. | LCA data on videoconferencing peripherals and transport are sometimes rough estimates and not always completely up to date. **Temporal:** the hourly carbon cost of a meeting was calculated by adding the cost of operating the equipment for one hour to the CE generated throughout all other lifecycle phases amortised over the whole length of the equipment. **Geographical:** a generalized conversion factor of 0.6 kCO2e/kWh was used to convert the results to carbon dioxide equivalents, based on the methods developed by Ong and Malmodin, because the processes of designing, manufacturing, using, and disposing of the technology may differ across locations and countries. The authors believe that they may have overestimated the carbon cost of the use phase because northern Sweden has access to environmentally friendly electricity in the form of hydropower and wind power. |
| Kemble 2023 | Y | Inventory analysis | Institutional device database, manufacturing data and research papers. | Single use (SU) cystoscope manufacturing data were obtained from the manufacturers and confirmed using previously published estimates (Davis et al, 2018).(14) Manufacturing carbon footprint of SU cystoscopes was calculated using the equivalent kg of CO2 manufacturing cost of each material comprising a SU AmbuaScopeTM 4 Cysto device, using a standardised CO2 protocol.(8, 9, 14, 16)Reusable cystoscope data were obtained from an institutional device performance database. The per-case carbon footprint for SU cystoscopes were calculated: manufacturing cost of cystoscope; manufacturing cost of packaging; mass of solid waste; manufacturing sterilisation; and transportation costs. The per-case carbon footprint for reusable cystoscopes were calculated: manufacturing cost; reprocessing (energy consumption of reprocessor); repackaging; reprocessing personal protective equipment; repair cost; and mass of solid waste. **Transportation costs:** calculated by using 26.5 g CO2/ton-km of shipping cost (Shiptraffic.net) |  | Per case | **Scope of LCA:** manufacture, packaging, sterilisation of device before shipping, transportation, and solid waste disposed after use. **Reusable cystoscopes:** manufacturing costs, reprocessing and repackaging between uses, personal protective equipment (PPE) used during reprocessing, periodic repair costs due to device damage sustained during clinical use, and the solid waste disposed after the device is irreparably damaged and permanently removed from clinical use. | Lifecycle began at manufacture and concluded at disposal. | **Transportation:** costs were calculated by using 26.5g CO2/ton-km of shipping cost (Hopkin, 2008). The distance of 13,289 nautical miles from the Ambu factory port in Malaysia to the port of Duluth, Minnesota, was used (Shiptraffic.net). **Detergents used in reprocessing:** the environmental impact of detergents used in reprocessing was not included as there have been uncertainties in the carbon footprint models for detergents (de Koning et al, 2009). Authors concluded that including the environmental cost of detergent to the model was unlikely to impact the results significantly. |
| Le 2022 | Y | LCA - The primary outcome of the LCA was carbon dioxide emissions (kg CO2 equivalent), complemented by 22 other environmental indicators; and the secondary outcome was impact on human health. | SimaPro 9.1.1 software (Amersfoort, Netherlands) and the Ecoinvent 3.8 cutoff database (Zurich, Switzerland. Data from manufacturers. Used the ReCiPe 2016 life cycle impact assessment method (Hierarchist version) (Huijbregts et al, 2017).(17) | The weights of the duodenoscopes were obtained from the manufacturers and were 1.49 kg for the RDs and .69 kg for the SD. The composition of the RDs was assumed to be 90% plastic, 4% steel, 4% electronics, and 2% rubber by weight. These values were based on available data for a similar medical device, the ureteroscope. Because of the lack of data on the composition of SDs and similar disposable medical devices, a lower bound scenario and an upper bound scenario was modelled for the SD. The upper bound SD scenario would have the same mass of electronics as the RD, whereas the lower bound SD scenario would have the same percentage of electronics as the RD. This is a key parameter because the electronics account for over 95% of the impacts of duodenoscope manufacturing, and although the SD might have fewer complex electronics compared with the RD, the percentage of reduction in the weight of the electronics was likely lower than the percentage of reduction of the other components, leading to an effective composition within the above-described upper and lower bounds. The model was designed to provide a range to account for changes in future design from not just 1 but a variety of SD manufacturers. The other components of the SD (plastic, steel, and rubber) were assumed to follow the same ratios as in the RDs. The intraprocedure electricity used for the RD was calculated based on the manufacturer’s manual, assuming the same amount of energy was used across all scenarios. The 2 RDs were assumed to be reused 125 times per year for 5 years, thus a total number of 625 reuses, whereas the 5-g endcap was replaced after each use. This was based on the procedural volume of an average-sized community hospital. Using a kilowatt meter, the authors prospectively recorded the average energy consumption of reprocessing 10 RDs used on 10 consecutive patients who underwent ERCP at All Saints Hospital (Racine, Wisc, USA) from February to March 2020. The endoscope reprocessor used was Advantage Plus (Medivators, Minneapolis Minn, USA), and the detergents used in reprocessing the RDs (Intercept [Medivators], Rapicide high-level disinfectant [Medivators], and Prolystica 2X concentrate enzymatic presoak and cleaner [Steris, St Charles, Mo, USA) were also accounted for. | The SimaPro 9.1.1 software (Amersfoort, Netherlands) and the Ecoinvent 3.8 cutoff database (Zurich, Switzerland) were used to estimate the midpoint impact on 23 different aspects of human and environmental health, such as particulate matter emission, carcinogen emission, effects on aquatic and terrestrial ecosystems, GHG emission, fossil resource consumption, water consumption, and land use. These midpoint impact results were then used to calculate the duodenoscopes’ endpoint impact using the ReCiPe 2016 life cycle impact assessment method (Hierarchist version). This method considers three endpoint impact categories: human health, expressed as disability-adjusted life years; ecosystem quality, expressed as the number of potentially lost species integrated over time (species per year); and non-renewable resource use, expressed as the extra cost for future mineral and fossil resource extraction (in U.S. dollars, reference year 2013). To facilitate the comparison of these impact categories, the results were normalized by dividing them by the total impact each person produces a year in each category globally. **Measuring toxic impact of chemicals in cleaning products:** The fate of chemicals in the duodenoscope cleaning products after passing through the wastewater treatment plant was further modelled using the EPI Suite 4.11 and USEtox 2.12 packages. EPI Suite estimated estimate the fraction of the cleaning products’ chemicals that reaches freshwater ecosystems after sewage water is processed at wastewater treatment plants. USEtox was used to evaluate the toxic impact of the cleaning products’ chemicals on freshwater ecosystems. | 1 ERCP procedure | 'Cradle to grave' environmental and human health burdens of SDs and RDs. | Production, transportation, disposal and electricity use of RDs and SDs, and high-level disinfection of RDs. | **Technical:** The model was based on key assumptions and the authors could only approximate the material composition and manufacturing energy of the RDs and SDs using the ureteroscope as a surrogate device, because they were unable to obtain empirical data on the duodenoscope’s composition or the energy involved in assembling its components. Steps were taken to avoid favouring RDs and the model favoured SDs in two ways: RDs were compared with the best-case scenario of SDs and the risk of serious infection from RDs was included, whereas the risk of SDs was assumed to be zero. |
| Leapman 2023 | Y | Inventory Analysis: does not consider impacts other than carbon. | LCA interface software SimaPro version 8.5.2.3 (Pre Consultants, Amersfoort, The Netherlands). Life cycle inventory database Ecoinvent 3.3. Environmental Protection  Agency’s life cycle impact assessment method TRACI 2.1 version 1.04 (Tool for Reduction and Assessment of Chemicals and Other Environmental Impacts). Chemical Life Cycle Collaborative (CLiCC; University of California, Santa Barbara, CA, USA) Life Cycle Impact Assessment Estimate tool. Environmental Protection Agency’s Greenhouse Gas Equivalencies Calculator. Research papers. ISO 14040. | Primary study endpoint was GHG emissions in units of kg CO2 equivalents (CO2e), as calculated using LCA interface software SimaPro version 8.5.2.3 (Pre Consultants, Amersfoort, Conversions of GHG emissions from kg CO2e into tangible units of measurement (such as kg of coal) was performed using the Environmental Protection Agency’s Greenhouse Gas Equivalencies Calculator. For each process step, data were collected on the physical resources used and their treatment or end of life pathways. A materials inventory included all single-use and disposable supplies used in each process step, primary material types, the weight of each material (measured directly with a scale), estimated lifespans of reusable supplies based on manufacturer specifications, and end-of-life disposal methods. Disposal methods included regular landfill waste (sometimes referred to as “white bag”), biohazardous solids (“red bag”), sharps, paper recycling, and, in the laboratory, drain and chemical wastes. Reuse processes including laundering (semi-industrial washer and drier) and sterilisation in central sterile (machine washer and autoclave) were calculated using energy consumption estimates from machine specifications and typical loading patterns for each machine. The commuting travel of staff was estimated using the total number of staff on hand in each process step, an assumed roundtrip distance of 25 km travel, an assumed mode of transit (where 80% of staff travel by car, 15% by bus, and 5% by bike), and the duration of the process step divided over an 8-hour workday. Travel of patients was also assumed to be 25 km roundtrip, occurring once each in both process steps 1 and 2. Energy use from the Heating, Ventilation, and Air Conditioning (HVAC) system, lighting, and equipment plug loads was estimated using the duration of each process step and an average power consumption of each component. Power estimates were gathered from either equipment specifications or previous published work by author group. | Estimates for five strategies used in contemporary care were generated. The baseline scenario was a MRI-fusion biopsy including separate targeted (two to seven, assuming the possibility of more than one lesion) and systematic biopsy (12) cores, for a possible 14–19 samples. Additional scenarios included mpMRI with two to five targeted biopsy samples only (scenario 1), systematic 12-core biopsy without prostate MRI (scenario 2), mpMRI with 12-core systematic biopsy only (scenario 3), and biparametric MRI with targeted and systematic biopsy, 14–19 samples (scenario 4). The authors estimated population-level reductions in the use of a prostate biopsy that may be contributed by the use of prebiopsy MRI as a triage tool. The primary analysis was conducted using a 28% incidence of negative MRI reported in the PRECISION trial (Kasivisvanathan et al, 2018).(18) In these scenarios, a biopsy was omitted after negative MRI and only targeted biopsies were obtained. Sensitivity analyses were conducted at lower (5%) and upper (50%) boundaries of negative MRI findings based on varying reports within the literature. The authors assumed equivalent cancer detection using biparametric MRI and mpMRI. Sensitivity analyses were performed to assess variation in the results when changes are made to model assumptions. These included variation in the number of single-use disposable supplies consumed, longer or shorter lifespans (0.5 or 1.5 assumed average value) for reusable supplies, and variation in waste disposal through a biohazardous treatment pathway. They also assessed the effect of different commuting patterns, including longer average commutes or travel conducted entirely by car or entirely by bicycle, as well as by national energy grids. Finally, they assessed variation due to changes in energy use from more or less intense power consumption of the equipment and heating, ventilation, and air conditioning (HVAC) systems. Owing to the dominant role of energy, they estimated that emissions would differ by energy grid mix. In Sweden, a country with a greater share of low-carbon electricity, they estimated that total emissions for MRI and prostate biopsy would be 38.2 kg CO2e, – 53% of the US baseline estimate. Conversely, in countries with more carbon-intensive energy sources such as Australia, the total emissions would be 124.5 kg CO2, +54% relative to the US estimate. | A single prostate biopsy procedure and anatomic pathology analysis with prostate MRI obtained beforehand | The prostate biopsy pathway was divided into three process steps (Figure 1): (1) prebiopsy prostate MRI, (2) a TRUS biopsy in an outpatient clinical setting, and (3) pathologic processing of biopsy specimens in a clinical laboratory. For each process step, the following were considered: materials (includ. single use and disposable supplies), disposal, laundering, sterilisation and energy consumption, commuting travel of staff, patient travel, and energy usage (heating, ventilation, air conditioning). The scope was limited to the diagnostic procedure itself and did not incorporate the environmental emissions from prior workup, procedural complications, and prostate cancer detection. Capital equipment (other than use-phase energy), including upstream and downstream emissions of MRI, ultrasound, image registration, and laboratory process steps were regarded as out of scope. | 'Cradle to grave’ - materials, energy, sterilisation, patient and staff travel. | **Geographical:** In this study, standard energy mix in the Northeastern USA was assumed, with sources derived predominately from fossil fuel. In areas with more or less clean energy sources, results are likely to vary. The LCA methodology was sensitive to numerous assumptions regarding energy estimates, transit pathways, and resources that were directly observed in a single institution. Although the authors accounted for variations in these assumptions using sensitivity analyses, differences in MRI, biopsy, and pathology analysis approaches between institutions may exist. **Technical:** study focused on the TRUS guided approach but modifications to the procedure (e.g. transperineal approach) are now preferred, as per the European Association of Urology Guidelines. |
| Leiden 2020 | Y | LCA | Software tool Umberto NXT was used and the Ecoinvent 3.1 database was the main life cycle inventory database used. | **Reuseable:** Depending on the requirements of the lumbar surgery, only a part of the set, which encompasses six boxes including R® eleven trays with several instruments, screws and rods, was applied. The total weight was 45.5 kg per set. It is used for five years and discarded through a solid waste incineration process at the end of life. It was assumed that the conventional set is used for 60 lumbar fusion surgeries per year. Hence, 300 surgeries can be realised throughout the lifetime of one reusable set. To fulfil the functional unit of one surgery, only a three-hundredth of the weight of the reusable set is taken into account for production and disposal processes, but the total weight for the use phase. According to experience, 10% of the instruments are lost per year. Beyond that, a loaner system for providing the surgical instruments was assumed for the base scenario. Thus, the distributor provides the complete set of surgical instruments, which must be sterilised in hospital before the surgery. After the surgery, it is cleaned and sterilised again and sent back to the distributor. There, the set is checked and complemented before returning it to hospital.  **Single use:** The Neo Pedicle Screw System from Neo Medical SA comprised one package with few instruments, one package with two rods and two packages with each two screws, nuts, screw extenders, and screw drivers. All parts of this single-use set are applied for a one level lumbar fusion surgery. The  total weight is 2.0 kg per set. After manufacturing and packaging, the 60Co set is gamma-sterilised, transported to the central distribution point, Frankfurt, and delivered to the hospitals. Here the whole set is used once for a one level lumbar fusion surgery. Screws and rods are implanted, packaging and instruments are discarded and incinerated as solid waste. | To conduct the LCA, the software tool Umberto NXT was used and the Ecoinvent 3.1 database. For critical processes, for example, the energy demand for steam sterilisation, power measurements were conducted. The selected impact categories were the cumulative energy demand (CED), abiotic depletion potential (ADP), global warming potential (GWP), acidification potential (AP) and particulate matter (PM). A single score indicator was used (ReCiPe Endpoint), which aggregated 18 different impact categories contributing to human health, ecosystem quality and resource availability. The use of individual impact categories and endpoints allowed for a comprehensive analysis to ensure that the aggregated characteristic of endpoints did not hide specific negative impacts. All score indicators were calculated by the corresponding life cycle impact assessment methods (CML 2001 and ReCiPe 2008) integrated into Umberto NXT software.  **Sensitivity analysis** was performed. Firstly, the number of usage cycles was increased from 60 to 100 times per year or rather from 300 to 500 surgeries in 5 years. And secondly, the application was changed from the loaner system to a consignment of the instruments by the hospital. Hence, instead of a double passage of sterilisation before and after the lumbar surgery and repeated transportation between distributor and hospital, it was now assumed that the reusable set of surgery instruments was delivered to the hospital once and remained there. Only spare parts were delivered in addition. The efforts regarding sterilisation and transportation were considerably reduced. Only an initial transport from distributor to hospital was required and only one sterilisation process per surgery was needed (see Figure 5). Thirdly, it was assumed that the surgical instruments were only cleaned and disinfected in the hospital and the final sterilisation is done in an external 60Co gamma sterilisation facility. The in-house cleaning and disinfection was still required to ensure the hygienic safety standards as the instruments were contaminated with blood and tissue after surgeries. It was assumed that the external 60Co gamma sterilisation was located next to the distribution point so that no additional transport were required. A scenario in which only used instruments were reprocessed was not considered as such a procedure would be accepted by health authorities and was not covered by the instructions for use. Damaged packages jeopardize the sterility for all instruments. | The specific set of surgical instruments for the realisation of single level lumbar fusion surgery (one surgery) including the implantation of four screws and two rods by means of a set of surgical instruments. | Scope encompasses raw material extraction and production of the instrument sets, spare parts and packages, transportation, sterilisation, use in hospital and final disposal (see Figure 1). | Production, transport, sterilisation, use/reuse, disposal. | **Geographical:** The production site of the reusable set was Indiana/USA and the production site of the disposable set was Switzerland. Both are transported to the distribution.  point in Frankfurt/Germany and distributed to hospitals in Germany. It was assumed that patients travel to a centrally located city within their metropolitan area for a spinal fusion. Based on eleven German metropolitan areas, in which 71 % of the German population live, an average distance from Frankfurt was calculated. The distance to Frankfurt, calculated with the route planning function of Google Maps, was weighted with the share of population living in a specific metropolitan area. The average of all eleven weighted distances was 3136 km, which represents the distance surgical instruments have to travel by lorry from the central distribution point Frankfurt. **Technical:** Data collection for washing and steam sterilisation was specific to a German hospital. |
| López-Muñoz 2023 | Y | LCA | EF was estimated using a free LCA software, OpenLCA V.1.11.0 (GreenDelta GmbH, Germany). Databases for lifecycle inventory analysis used include ecoinvent V.2.2, Agribalyse V.3.0 and EF Secondary Data sets V.EF 2.0. Impact assessment method applied was EF (midpoint indicator). | Carbon footprint was assessed as kilogram of CO2 equivalent (CO2 -eq) released, a common measure of global warming potential (GWP) from an LCA (manufacture, transportation, use and end of life) of each instrument to quantify total carbon footprint. The GHG emissions (eg, carbon dioxide, methane and nitrous oxide) across life cycle stages were converted into CO2-eq using an LCA ‘cradle to grave’ model. Laboratory detailed calculation of weight and composition of endoscopy instruments allowed the authors to precisely determine what kind of material components manufacturers use for production. GHG emissions derived from production of forceps, snares and clips from companies A–D were calculated. Therefore, most sustainable instruments were identified through LCA software. Several assumptions were made to estimate carbon emissions deriving from transportation. Based on manufacturing sites from different companies and ship-to-party, most frequent international routes were assumed. Emissions from shipping by cargo container for transoceanic routes and diesel lorry for continental ones were calculated. Since the databases used do not consider manufacturing and assembly steps (injection, extrusion and lamination), they were not included in the calculations, even though their environmental impact falls around 15% of the total. As single-use equipment was required to be processed via high temperature incineration, end-of-life emissions were estimated according to recent data of waste streams in the literature. The incineration of general BMW was estimated as 1.074kg CO2-eq/kg13 for non-plastics and 6kg CO2-eq/kg for plastics. The procedure was assessed across several environmental impact categories (ionising radiation, ozone depletion, human toxicity cancer/not cancer effects and acidification). Material-specific global average environmental impact of raw material extraction, production, and transportation to the ‘end user’ (in this case the manufacturer) of instruments and packaging was determined through matching materials identified with the closest processes within Ecoinvent v3.6, or where unavailable in Industry data v.2.0 (both databases integrated within SimaPro). Environmental impact of instrument manufacture was approximated using global average metal working processes for all metal components of instruments and injection moulding processes for plastic components of instruments (as modelled in Ecoinvent v3.6). The mode and distance of international transportation from site of manufacture to the UK was determined through discussion with instrument suppliers (Supplementary Table 1), and 80 km of travel by road using a heavy goods vehicle both within country of origin and in the UK, with the first and last 8 km at either end of this journey by courier, was assumed. All reusable components were assumed to be decontaminated and re-used 500 times, in accordance with manufacturer guidance on typical usage, with energy and material inputs for decontamination modelled using our own data presented elsewhere. | Comparison of means among groups was done using one-way analysis of variance or its corresponding non-parametric (Kruskal-Wallis) test, with a two-sided p value of <0.05 indicating statistical significance. Comparisons of proportions among groups were made with the χ2 test. All statistical analyses were performed using SigmaPlot V.12.5 (Systat Software GmbH, Erkrath, Germany). | Total number of biopsy forceps, polypectomy snares and haemostatic clips used during the one-week period* | The scope of the LCA analysis includes extraction of material and energy resources, manufacturing, transport between sites in the production process to the hospital and disposal at end of life. Since the databases used did not consider manufacturing and assembly steps (injection, extrusion and lamination), they were not included. Transportation from extraction of raw materials to manufacturing sites and BMW from hospitals to incinerators were not taken into account. When assessing end-of-life emissions, LCA software databases which include information about emissions derived from incineration of different materials (polymers and metals) could not be found. Consequently, incineration had to be estimated according to literature references. | Extraction of material and energy resources, manufacturing, transport between sites in the production process to the hospital and disposal at end of life. Cradle-to-grave carbon emissions (manufacturing, transportation and incineration) were estimated for every instrument and represented as kg CO2 -eq. | **Technical:** focused on commonly used disposable endoscopy instruments, a certain part of the overall endoscopy carbon footprint. **Geographical:** the assumption was that all instruments were sent to high-temperature incineration but regulations can vary by country with different waste management policies. |
| McAlister 2022 | Y | Inventory Analysis. Process-based LCA to undertake both attributional (ALCA) and consequential (CLCA) analyses to provide complementary perspectives of the carbon impacts of imaging. In the case of imaging, ALCAs determine what share of the total impact from operating a given modality can be attributed to a single scan (mean impact), helping to identify the sources and magnitude of operating diagnostic imaging as a whole. By contrast, CLCAs only model the changes, such as a scanner moving from standby to active, that result from undertaking one additional (or fewer) scans within an already operational system. | Ecoinvent 3.5 database (ecoinvent, Zuricg, Switzerland). Australian electricity generation mixes and emissions factors (Appendix p. 4). LCA was conducted in accordance with the International Organization for Standardization (ISO) standard 14040:2000. | To calculate power usage for CT, MRI, US and CXR, power usage was divided into two states: active power (including positioning a patient or scanner, and imaging the patient), and standby power for all other times. Two distinct standby power states were identified for CT, MRI and US imaging devices; passive standby, when the scanner was in a deep low-power mode overnight, and active standby, for the time the imager was in-between patients during operational hours (Appendix p. 3). For the ALCA, the average power consumed by each modality was estimated as the sum of the average active power (per minute for US, CT, and MRI, or per image for CXR) added to its respective proportion of standby power on a per minute basis, including computers and monitors. For the CLCA, power usage of scanners was estimated as being the difference between the average active power and active standby power per minute. The impacts of computers and monitors were not included in the CLCA analysis, as these were running regardless of an image being ordered. As the MCXR operates on battery power, two charging modes were identified: active charging, when the mobile unit was first plugged back into the charger after performing imaging, and trickle charging when the unit’s battery was fully charged. For the ALCA, power usage per MCXR was estimated as the sum of the total power divided by the total number of MCXRs performed. All data were collected between 10 February 2021 and 30 August 2021. The electrical power consumed by each modality was collected, as was the weight and composition of materials used by patients and staff such as sheets, gloves, gowns, contrast, needles and syringes (consumables) (Appendix p 1). These materials were weighed with a Satrue KA-1000 scale (resolution 0¢5g). Usage of consumables was estimated by the radiographers undertaking the scans and X-rays. Three-phase electrical power (400 volts) was measured sequentially during the collection period for each of the CXR, CT and MRI imagers (1¢5 Tor) for a period of approximately two weeks by a Hioki PW3365-20 power meter (Hioki, Nagano Japan), using a sampling rate of one sample every 15 s. Single phase electrical power (230 volts) measured by a Watts Up Pro power meter (Vernier, Oregon USA) connected to Logger Pro 3¢14¢1¢0 (Vernier) with a sample obtained every 30 s for a period of two weeks for US, and for four days for MCXR. Power consumption of computers and monitors in the imaging control rooms was obtained from manufacturers’ published data. | Impact assessment was modelled using the ReCiPE 2016 (H) impact assessment model (Huijbregts et al, 2016).(17) | The functional unit of the ALCA is a CT, MRI, US, CXR or MCXR, in an Australian public hospital setting, while the functional unit of the CLCA is an additional CT, MRI, US, CXR or MCXR, in an Australian public hospital setting. | Capital infrastructure, including the manufacture of scanners, was excluded as environmental impacts are amortised over long periods of time and are typically small. The energy used by both the hospital’s HVAC (heating, ventilation, and air conditioning) system and the room-specific air conditioning energy required to maintain the CT and MRI scanner rooms at 18°C were excluded, so as to make the results generalisable irrespective of geographic location and time of year. The environmental impact of the manufacture of radiological equipment was excluded due to the amortised impact per scan being known to be very small, and because it cannot be estimated with precision without detailed manufacturer’s data about the weights and composition of all of a scanner’s components (generally not available for commercial in confidence reasons). However, the issue was explored by using publicly available data (appendix p. 7), together with the usual duration of a machine life of 10−15 years to estimate this impact. (See Figure 1) | Resource extraction, manufacturing, electricity, waste disposal. | **Geographical & Temporal:** The decision to exclude the energy used by both the hospital’s HVAC (heating, ventilation, and air conditioning) system and the room-specific air conditioning energy required to maintain the CT and MRI scanner rooms at 18°C, was to make the results generalisable irrespective of geographic location and time of year. This was based the authors’ previous study in which they calculated the HVAC energy requirements for a hospital ward in two different geographic and climatic locales, with the study showing that HVAC energy varied greatly due to both location and time of year. That the authors only investigated a single scanner of each modality in a public hospital setting in one country is a limitation. Large differences in scanner energy consumption have been reported, due to both differences in actual scanners as well as usage patterns. Consequently, absolute levels of emissions at other sites may differ. This study, however, does show their expected relative magnitude. The authors recommend that further studies be undertaken to measure a range of scanners of varying ages, sizes, and manufacturers, as well as in different settings. |
| Meiklejohn 2023 | Y | Hybrid LCA (incorporates both process LCA and Economic Input-Output LCA) | Ecoinvent database (version 3 part 1). EIO-LCA was used for processes or products in which the primary constituents could not be identified and/or the corresponding unit production processes were not available in Ecoinvent. The price paid by the hospital for the equipment and medications was matched with the quantity used in each procedure, and this was used to determine the associated impact. Several North American Industry Classification Systems (NAICS) were used for EIO-LCA, for example,  325412 Pharmaceutical Preparation Manufacturing, and 339112 Surgical and Medical Instrument Manufacturing, etc. The US 2002 producer price model was used for the EIO-LCA, thus, all the dollar amounts paid by the hospital were converted to 2002-dollar amounts. Operating Room Ventilation Systems Best Practices Guide was used to calculate operating room energy (Jarvis & Vahabi, 2017).(19) US Department of Energy benchmarks were used to calculate natural gas usage (Tip Sheets by Sysytem. Energy.gov). Environmental impacts were calculated using the Tool for the Reduction and Assessment of Chemical and other Environmental Impacts, TRACI 2.1 software for both process LCA and EIO-LCA. | **Surgical processes and procedures:** Data were obtained from 15 cases of adult tonsillectomy that were prospectively randomised for surgical technique: cold excision without cautery. After conclusion of the surgery but prior to turnover and cleaning of the room, the entire room was inspected by study staff and the trash inspected and sorted. All disposable items were tabulated, including items opened in error or opened and unused. Disposable items were identified using purchasing and supply chain data for manufacturer, location of origin, and materials composition. All items, including packaging materials, were weighed and their volume measured using volume displacement cylinders. Where possible, the primary constituents of each disposable item and packaging material were identified. Stainless steel surgical instruments were assumed to have 300 uses in their lifetime. Operating time was determined by electronic medical record review of 36 consecutive unassisted tonsillectomy procedures, including the 15 study cases and an additional 21 historical cases from all three techniques. **Anaesthesia processes and equipment:** For the 15 study cases, the Anaesthesia practitioner recorded all supplies used to complete the anaesthetic. After the conclusion of surgery and prior to turnover, the room was inspected by study staff and the trash inspected and sorted. All disposable items were tabulated, including items opened in error or unused items, and measured as described above. **Operating room energy:** The electrical power and natural gas used for OR heating, ventilation, and air conditioning (HVAC) were calculated using equipment data, operating time, and the Operating Room Ventilation Systems Best Practices Guide where appropriate. Electrical power was tabulated from digital meters and multiplied by operating time to quantify consumption for all other electrical devices. **Sterile processing and laundry:** Parameters for sterile processing equipment were obtained from equipment vendors and sterile processing staff. Natural gas usage for steam production was determined using US Department of Energy benchmarks with modifications made based on local conditions. Environmental impacts were assessed by aggregating the combined impacts from raw material extraction, production, usage, and disposal. All single-use equipment was assumed to be disposed, as the facility did not recycle OR waste. | Typically, a standard number of reusable and disposable items are used during tonsillectomy surgeries. In cases where these numbers were not fixed (e.g. need to open additional suture material for haemostasis in cold technique), a probability was more appropriate. Monte-Carlo Simulation (MCS) was used to account for the data variability in the analysis. Random numbers were chosen from a uniformly distributed probability distribution. For anaesthetic medications, normally distributed probability distributions were adopted based on the Anderson-Darling test. For each type of tonsillectomy surgery, a total of 100,000 MCSs were conducted. Differences between techniques in environmental impacts were compared using one-way Analysis of Variance (ANOVA). Because of the large number of identical inputs between the techniques, a nonparametric Kruskal-Wallis test and post hoc pairwise comparison based on the Wilcoxon rank sum test were used to compare disposable equipment between techniques (see Methods in the Supplement). Student’s t-test was used to compare operating times. Software used included Matlab for MCS and R for statistical analysis. | One tonsillectomy, spanning the duration of the surgical procedure, and included all materials and processes necessary for procedure completion. | System boundaries included raw material extraction and processing for manufacturing and production of equipment and materials used in the OR as well as their disposal, and materials extraction and processing for fuels and electricity used to power OR processes as well as for cleaning and sterilisation of equipment and linens (see Fig. 1). All three scopes of GHG emission categories were included as defined by the GHG Protocol 41 (see Methods in the Supplement). All pre- and postoperative processes and the production and disposal of durable OR equipment were excluded, apart from surgical instruments. Excluded from the study scope were patient and staff travel to and from the hospital, pre- and postoperative processes and factors, and manufacture and maintenance of non-surgical durable equipment such as computers, anaesthesia ventilator, light sources for headlight, sterile processing equipment, etc. | Cradle to grave - raw material extraction and manufacture, energy usage, sterilisation, landfill, autoclave, incineration. | **Technical**: EIO-LCA was used for processes or products in which the primary constituents could not be identified and/or the corresponding unit production processes were not available in Ecoinvent. The price paid by the hospital for the equipment and medications was matched with the quantity used in each procedure, and this was used to determine the associated impact. **Geographical:** Several North American Industry Classification Systems (NAICS) were used for EIO-LCA, for example, 325412 Pharmaceutical Preparation Manufacturing, and 339112 Surgical and Medical Instrument Manufacturing. Natural gas usage for steam production was determined using US Department of Energy benchmarks. Cost and energy consumption related to laundry were calculated based on energy and cost data from the hospital and the contracted laundry facility, and calculated per surgery based on kilograms of laundry soiled per surgical case. The cost and kgCO2-eq produced of electrical power and natural gas were obtained from utility companies and the University Utilities Division. **Temporal**: In this study, US 2002 producer price model was used for the EIO-LCA, thus, all the dollar amounts paid by the hospital were converted to 2002 dollar amounts. |
| Rizan 2022 | Y | LCA | Used SimaPro v9.10 (PRé Sustainability, Amersfort, Netherlands). Ecoinvent v3.6, or where unavailable in Industry data v.2.0 (both databases  integrated within SimaPro). Information from suppliers. Used ReCiPe v1.1 Midpoint Hierarchist | Hybrid instruments were supplied by Surgical Innovations Ltd. (Leeds, UK) and Microline Surgical Inc. (Beverly, USA). Equivalent single-use instruments were identified from the catalogue of the UK National Health Service (NHS) Supply Chain. The raw material composition of each instrument and associated primary packaging was determined using information provided by manufacturers through personal correspondence, or expert knowledge where manufacturers were unable to provide sufficient detail. Weight of component materials was determined using Fisherbrand FPRS4202 Precision balance scales (Fisher Scientific, Loughborough, UK). All reusable components were assumed to be decontaminated and re-used 500 times, in accordance with manufacturer guidance on typical usage, with energy and material inputs for decontamination modelled using author data (presented elsewhere). The metrics of the washer/disinfector and sterilisation cycle varies according to a number of factors, including the loading of machines, electricity source, and constitution of instrument sets. Reusable components of hybrid instruments were assumed to be integrated into a general laparoscopic set, as is common practice. The typical weight of instruments on a general laparoscopic set was 730g (data from the local hospital) and the weight of hybrid reusable components was 594g. Thus, hybrid instruments within a set comprise 45% of total weight (594/1324 g), and the authors also apportioned 45% of environmental harm from decontamination of the set to the reusable components of hybrid instruments. At the end of their life, all items were assumed to be disposed of as clinical waste via high-temperature incineration. | The ReCiPe v1.1 Midpoint Hierarchist method (integrated within SimaPro) was used to characterise emissions and to combine these into environmental impacts. This method evaluates eighteen midpoint impact categories (each relating to a single environmental problem): global warming, stratospheric ozone depletion, ionising radiation, ozone formation (on human health, and terrestrial ecosystems), fine particulate matter formation, terrestrial acidification, eutrophication (freshwater and marine), ecotoxicity (terrestrial, freshwater, and marine), human toxicity (carcinogenic and non-carcinogenic), land use, resource scarcity (mineral and fossil), and water consumption. A deeper analysis of global warming impact was performed and hotspot analysis was conducted to determine processes contributing most to carbon footprints. The authors used the ReCiPe v1.1 Endpoint Hierarchist method to aggregate midpoint impact categories to calculate endpoint factors for damage to human health, the natural environment, and resource scarcity. Finally, they used ReCiPe v1.1 Hierarchist normalisation factors to compare total midpoint and endpoint impacts to mean average contributions to each of those impacts from a global average person’s daily routine activities. | The number of the three types of instruments - laparoscopic clip appliers, laparoscopic scissors, and ports (small diameter 5 mm ports, and large diameter 10–11 mm) - typically required to perform one laparoscopic cholecystectomy: two small diameter ports, two large diameter ports, one laparoscopic scissor, and one laparoscopic clip applier. | Other reusable instruments and consumables used to perform a laparoscopic cholecystectomy were beyond the scope of this analysis. | 'Cradle to grave’ - raw material extraction, manufacture, transport, and disposal, plus decontamination for reusable components. | **Technical:** Sensitivity analyses conducted by modelling five different scenarios to determine sensitivity of results to allocation methods and key assumptions. (i)the impact of altering the number of uses of instruments; (ii) decontamination with the clip applier decontaminated separately in a flexible double wrapped polyethylene pouch; (iii) the impact of switching the electricity source for decontamination to that typical of Australia, a country using a lower proportion of renewable energy; (iv) impact of changing overseas transport of single-use instruments to shipping by sea, with distances determined using the online Pier2Pier tool and alternative road distances using Google maps; (v) the carbon footprint of using three 5 mm ports and one 10/11 mm port, as this is a commonly used alternative port configuration for laparoscopic cholecystectomy. |
| Rouviere 2022 | Y | LCA | Manufacturer's data sheets for material composition of all studied devices. Supplier information. | The weight of each small device or packaging was measured using the same calibrated scale, and the weight of garbage bins was measured with a dedicated scale. Data on the consumption of water, electricity and chemical products necessary for the washers and autoclaves were collected through the suppliers. The before and after scenario waste management also was evaluated. | The ecological impact was evaluated using the LCA method with the SimaPro® v9.2.0.1 software. The results obtained by the LCA method were then summarised and classified in seven categories: global warming (climate change impact) (kg eq CO2), occupied land (m2 year crop eq), human toxicity and environmental toxicity (aquatic and terrestrial ecosystems) (kilo gram equivalent of 1,4 dichlorobenzene; kg eq 1,4-DCB), depletion of mineral resources (non-renewable resources) (kilogram of copper equivalent; kg eq Cu), depletion of fossil resources (non-renewable resources) (kilogram of oil equivalent; kg eq Oil), and depletion of water resources (m3 of water). | Thirteen functional units prior to intervention ('before scenario') which changed after the intervention ('after scenario'). Prior to intervention (changed after intervention): A. Waste reduction actions: (i) & (ii) Individual packaging for each sterile medical device (SMD); (iii) Anaesthesia mask with plastic hook; (iv) Redon drain: Pre-mounted needle on the Redon drain systematically wasted (ICWb) during robotic surgery; (vi) Laryngoscope blade: Single-use laryngoscope blades; (x) Irrigation fluid: Evacuation of surgical fluids to the ICW with a classic system using flexible bags; (xiii) Surgical kits: Using double packaged surgical kits. B. Waste sorting actions: (v) Aluminium blisters: Aluminium blisters wasted in NICW; (vii) no recycling channel in OR; (viii) Metal waste recycling in SPD: Defective SMD not eligible for repair wasted in NICW; (ix) Use of triclosan coated surgical sutures: Widespread use of triclosan-coated sutures; (xii) Recycling of ES wires: ES wires wasted in NICW. C: Eco-responsible purchasing action: The final score when referencing a medical device was based on the technical (60%) and economic (40%) scores. | Manufacturing processes were not considered due to a lack of information. | NR clearly for all functional units. Needle/blade/suture composition, use/reuse, sterilisation/disinfection, waste/disposal. | NR |
| Sanchez 2020 | Y | LCA | Material and process-specific life cycle inventory data were matched with unit processes from the US-EI database, which includes USLCI and Ecoinvent v2.2 data adjusted for the US energy system. Transport packaging was assumed to be recycled at US average rates for each material (USEPA, 2014). Specific LCI database entries that were used were listed in the Supporting Information for each BP cuff (Tables S3-S4) and each cleaning product (Tables S3-S5). | The composition of each BP cuff was determined through general information from manufacturer specifications and physical testing. Each cuff was disassembled into individual pieces and then weighed on a milligram scale. Material types were determined through individual labels and published product descriptions, expert elicitation, and polymer burn tests (see Table 1 for material composition of BP cuffs). Specific entries from life cycle inventory databases were matched with each component (see Supporting Information Tables S1-S2), enabling modelling of the supply chain of each specified material. The two cuffs have similar masses, despite being composed of a number of different materials. The total masses of the reusable and disposable BP cuff are 73g and 66g, respectively, including packaging. BP cuffs were individually wrapped, packed 50 per box and then two boxes per case. Associated plastic film, boxboard and corrugated cardboard packaging were proportionally allocated to each cuff. How each BP cuff was used, reused, and discarded was expected to have the highest variability of any phase of the product life cycle, given the number of administrative, operational, and behavioural factors at play in any given clinical setting. A range of use and cleaning scenarios were modelled to represent a diversity of clinical settings, normalised to one day in each setting. **1. Outpatient:** This scenario considered a single examination room with 20 patient encounters per day. Therefore, 20 disposable cuffs were compared to 1 reusable cuff-day (where there is a single shared cuff). **2. Ambulatory procedure:** A typical ambulatory patient encounter could involve 3 separate locations, each with a stationary BP cuff, such as the pre-procedure holding area, the operating room, and the postanaesthetic care unit. This scenario considered 10 patient encounters per day. Therefore, 10 disposable cuffs were compared to 3 reusable cuff days where there was one shared cuff in each of the three locations. **3. Inpatient:** **Regular ward:** a hospital ward with 20 patients was assumed, with an average stay of 5 days. In this scenario, the impacts and costs of the disposable cuffs must also be amortised over their useful lifetimes, as they stayed with each individual patient for more than one day. This setting required: (1 disposable BP cuff/patient) × (20 patients / 5 days) = 4 disposable cuffs per day. The regular ward was assumed to have a single reusable BP cuff on a mobile cart that was shared among patients, with each patient having their blood pressure measured 4 times per day. Therefore, 4 disposable BP cuffs were compared to 1 reusable cuff day**. 4. ICU:** An ICU ward with 20 patients was assumed, with an average stay of 5 days. Unlike the other settings where reusable cuffs were routinely shared, each patient in the ICU was assumed to have a dedicated reusable BP cuff due to the need for more frequent measurements. Therefore, this setting required 4 disposable BP cuffs (as with the Regular Ward) compared to 20 reusable cuff-days. **Cleaning:** a ¼ of a wipe was allocated to the BP cuff itself. Each wipe was modelled as 1 g of cotton substrate with active ingredient n-alkyl dimethyl ethylbenzyl ammonium chloride (21 mg) and isopropyl alcohol (2.3 g), based on product labels and the product safety data sheet (PDI Super Sani-Cloth). Packaging and transport of the sanitising wipes are also included, with 160 wipes per bottle and the HDPE bottle shipped 1200 km from Illinois. Decontamination was modelled in all scenarios, occurring once every five days, in addition to the routine LLD with chemical wipes per day or per encounter. The enzyme bath was assumed to be filtered water, with 20 devices per bath, using an enzymatic detergent at 1 fluid ounce per gallon of water, with one gallon of water assumed for rinsing. Typically, dedicated reusable cuffs are only used for ICU patients. However, this management strategy could also reduce the number of cleanings in other settings, and so was modelled as alternative scenarios. Research has noted lack of routine cleaning of dedicated BP cuffs in multi-day inpatient settings to be a potential source of re-infection (de Gialluly et al., 2006).(20) Scenarios were modelled whereby all dedicated BP cuffs in use more than 24 h—whether reusable or disposable—were cleaned daily. At end of product life, both types of BP cuffs and sanitising wipes were assumed disposed of as standard municipal solid waste (not bio-hazardous) and then either landfilled or incinerated. | LCA modelling was performed using the commercial LCA software package SimaPro 8.1 (PRé Consultants, Amersfoort, Netherlands). Environmental impact calculations were run using the US EPA’s impact assessment model TRACI 2.1, covering a standard set of environmental and health considerations (Bare, 2011).(21) Attrition and sensitivity analysis: the baseline analysis assumed that clinicians keep the reusable BP cuffs in use for the full three years suggested by the manufacturer. However, it may be that the reusable BP cuffs are discarded far earlier than this, which means that the environmental impacts of producing the cuff, and its purchasing costs, will be allocated over a smaller number of days, thus increasing the environmental emissions and cost results. The authors conducted a sensitivity analysis on attrition rates to find the ‘break-even’ lifetime of a reusable BP cuff such that its effective daily costs would be equal to those of a disposal BP cuff. Disposable BP cuffs also may be misplaced or discarded prematurely; however, one per patient was assumed for all modelling scenarios. | Providing blood pressure readings for a clinic room or ward, under four different health care delivery scenarios. | No recycling scenario was considered (except for packaging), as recycling is uncommon (though possible) given the types of plastics and mixed materials employed in the BP cuffs (Anon, 2019e). Certain indirect or infrastructure inputs, including room lighting and HVAC energy and materials, staff commuting, administration, restocking, and construction of the hospital/clinic itself were not included, as they were judged to differ only modestly between the two blood pressure cuffs, and outside the scope of this study. | Cradle to grave - materials and manufacturing, packaging, transportation, use/reuse, cleaning, disposal. | **Temporal:** The cost and environmental impacts associated with manufacturing and transporting the reusable cuff must be amortised over the lifetime of the cuff, which was taken to be three years, as described in manufacturer specifications. (Anon, 2019c) **Waste Management:** Rather than considering a single, site-specific end-of-life route, landfill and incineration options were considered for all clinical care scenarios in order to make the study applicable to a wide range of setting. **Geographical:** There was also data uncertainty associated with some of the modelling parameters, stemming in part from the national averages used in the background life cycle inventory data. For example, emissions due to electricity vary widely across different regions of the U.S., and yet national average emissions were applied throughout the study. For this reason, the absolute environmental impact results are less meaningful than the relative results. **Technical:** There is also uncertainty associated with the BP cuff materials. Material quantities were measured directly for the standard adult cuff, but the flame test used to ascertain material types can be imprecise. For this reason, the exact polymer blends or composite materials were grouped into general material categories (details in the Supporting Information). Finally, there is uncertainty around infection control practices for the different alternatives analysed. LCA studies are predicated on the assumption of functional equivalence of different product options, and for this study, this equivalence extends to infection control achieved through cleaning or utilising disposable items. Due to the lack of controlled data on nosocomial infections, uncertainty remains about this infection control equivalence between disposable and reusable BP cuffs. |
| Schulte 2021 | Y | LCA: follows a 'supporter perspective' for modelling comparative LCAs of remanufactured and newly-manufactured products to provide information on short-term impacts. Also analysed long-term impacts using a modelling approach including a circularity metric. Authors propose that LCA and Circular Economy (CE) indicators could be combined to compare newly-manufactured catheters from single-use and remanufactured catheters that could be used multiple times. The circularity of remanufactured products can vary between 1 and infinity. A circularity of 1 describes an entirely linear system; a circularity of infinity means a fully circular production system without losses. E.g. For the linear production system, 0% of the non-remanufactured and linear-used catheters are collected, which corresponds to a circularity of 1; and for the circular production system, it is assumed that 100% of the catheters to be remanufactured are collected and stored separately so that no losses occur here. Circularity can be measured by the average number of product systems in which a resource is involved before products or materials leave as non-recoverable waste. This circular production system can then be compared to a linear production system, where newly-manufactured catheters are used once and treated as waste without a remanufacturing process. The results of the circularity indicator can be used in combination with the LCA results of the 'supporter perspective' to analyse the environmental impacts of remanufacturing catheters multiple times (circular  used catheters) in comparison to disposal of those catheters after single-use (linear-used catheters). | LCA conducted according to ISO standards 14040 and 14044. Impact assessment conducted according to the recommendations of the environmental footprint (EF) 3.0 method published by the European Joint Research Centre (JRC). | For primary data collection, if data ranges were given, average values calculated. Process steps, final inspection, sterile packaging and final packaging assumed to be identical to medical remanufacturing route expect for transport distance to customer, as no further data available. Two transportation processes modelled for transport of newly manufactured catheter to Germany. 1) average sea route from California to Germany (approx. 16000km) assumed and final transport to customer was carried out with a small truck. Transportation distance to customer within Germany adapted from remanufacturer to customer (Average value 79.1km). Some simplifications were made in relation to the provision of materials for virgin catheter production which consisted of a plug, a handle, a curvature, a loop, and a shaft – e.g. The incorporated filler of polyurethane in the curvature and loop (barium sulfate) was not known and the modelled curvature and loop was assumed to contain polyurethane exclusively. This simplified assumption should not influence the LCA result because the curvature and loop correspond in total to 0.7 wt.% of the catheter. The plastic of the catheter shaft (PEBAX®) is a specific block copolymer obtained by polycondensation of a polyamide (e.g., PA6, PA11, PA12) with an alcohol termination polyether (Polytetramethylene glycol or polyethylene glycol PEG). As no LCA dataset was available for the production of PEBAX®, the material was modelled by two possible preliminary products, namely polyethylene glycol (PEG) and polyamide 6 (PA6). The shaft of the catheter had a small sleeve at the top (7.5 × 10−3 g), which is made of polyether ether ketone (PEEK). The data for the provision of a catheter through medical remanufacturing was gathered from Vanguard AG in Germany. The investigated remanufacturing process guaranteed the safety and functionality of its remanufactured medical products in full compliance with the specifications of the original products and with CE-certified products also in compliance with the requirements of the Medical Device Directive 93/42/EEC. A few assumptions were made to model the medical remanufacturing process. The German electricity mix was chosen to provide the electricity demand for medical remanufacturing because the company is located in Germany. The pre-used catheters are collected at hospitals in reusable polypropylene boxes. However, the reusable boxes’ production was neglected because the estimated number of reuses is higher than 250. The primary packaging at the collection point in hospitals was not considered for medical remanufacturing, as the plastic bag with the original label was attributed to the virgin production. The detergents and disinfectants for remanufacturing as well as for sterile packaging were modelled by their identifiable main components (>10.0%) according to the available safety data sheets on the internet. However, the ingredient glutaral in the disinfectant Neodischer Endo Sept GA was represented by a proxy dataset for steam cracking products, as no specific LCA dataset for the production process of glutaral was available in the GaBi database. | Impact assessment covered the following life cycle impact categories: Acidification (terrestrial and freshwater), cancer human health effects, climate change, ecotoxicity freshwater, eutrophication (freshwater, marine, terrestrial), ionizing radiation, land use, non-cancer human health effects, ozone depletion, photochemical ozone formation, resource use (energy carriers and minerals and metals), respiratory inorganics and water scarcity. Remanufacturing was expected to influence mainly the two impact categories concerning global warming and abiotic resource use so the analysis focuses on (i) Climate change (determination of the GWI) and (ii) Resource use, mineral and metals (determination of the abiotic resource use. | Provision of an electrophysiological diagnostic catheter for single-use. | For the newly-manufactured catheter: system boundaries comprised the provision of raw materials for the catheter production, followed by the process steps of final inspection, sterile packaging, final packaging, transportation to client, use of catheter and waste treatment of disposed catheter after single use. Manufacturing/provision of chemicals and materials, required energy, transport processes, water and packaging materials to distribute catheter were within scope. Medical remanufacturing route: system boundaries comprised collection of catheters at hospital in Germany, transportation of remanufacturing site, prefabrication process, medical remanufacturing, final inspection, sterile packaging, final packaging, transportation to the hotel and use of catheter. Prefabrication process included goods receipt, decontamination and identification of products. Medical remanufacturing process included disassembly, assembly, cleaning, disinfection and test. Final inspection and sterile packaging step included packaging in sterilisation pouches and sealing of sterilisation pouches, assignment of sterile packaged products to the sterilisation process as well as the sterilisation process and final visual inspection. All rejected catheters that failed identification, functional tests or failed sterile packaging were included and treated as municipal waste. Environmental impacts that occurred due to using catheter i.e. electricity needed for cardiac ablation, were not considered as no data were available. It can be assumed to be same for both catheter life-cycles, because application, including functionality and quality, were assumed to be equivalent. | Cradle to grave - virgin catheter. Remanufactured catheters considered as burden-free products - assessment begins with collection of pre-used catheters | **Geographical:** Virgin catheters assumed to be produced in USA and used in Germany. Hence study considers German conditions regarding waste management system. Accordingly disposed catheter treated as municipal waste. Since virgin catheter manufactured in US, US provider datasets were applied if available e.g. US electricity mix chosen for production and processing of virgin plastic for catheter components. If US specific data were missing, providers were selected according to countries or regions with similar production conditions e.g. EU/industrialised countries. **Technical:** datasets used for materials discussed in methods for calculation. Due to missing data, e.g., the proper concentration of the filler of polyurethane or the weight of the shaft sleeve, the uncertainties in terms of completeness and reliability are assumed to be higher for the virgin manufactured catheter. Furthermore, missing US-specific datasets might cause a higher uncertainty in the field of geographic correlation for the virgin production route. The impact results of the virgin production route could tend to be higher, but the results of the medical remanufacturing route are assumed to be more certain due to primary data. |
| Sherman 2018 | Y | LCA & LCC analysis. | Laryngoscope materials, energy and process inputs were matched with background life cycle inventory data from the Ecoinvent v2.2 database and adjusted for the US energy system (US-EI database, Earthshift, Huntington, VT). US Environmental Protection Agency's Tool for the Reduction and Assessment of Chemical and other | Material composition of all components under investigation was determined through a combination of manufacturer specifications, deconstruction, and density testing. The mass of each material was measured using a microgram scale. Data collection specific to Yale-New Haven Hospital (YNHH) included device transportation distance, and washer- and autoclave-associated energy, water, and chemical requirements for reprocessing. **Rated Lifetimes**: The reusable stainless steel laryngoscope handle was rated for 4000 uses, whereas the SUD alternatives were rated for 1 use (Table 1). To compare devices, 1/4000th of the manufacturing, transportation, and disposal impacts of a reusable handle plus 1 reprocessing cycle were therefore compared to the manufacturing, transportation, and disposal impacts of 1 SUD alternative. Reusable handle light bulbs and hinge pins become worn over time, especially as the level of disinfection was increased, and were periodically replaced. Refurbishment was conservatively estimated once every 40 uses. The reusable tongue blade comprised a stainless steel blade and a removable fibre-optic light conduit pipe. The steel blade had no rated limit; however, 4000 uses were assumed per lifetime. The removable fibre-optic light pipe was rated for 500 uses (Table 1). Total results for these components were similarly scaled to a single use and reprocessing cycle to compare to 1 SUD alternative. **Transportation and Packaging:** Transportation distances of devices to Yale-New Haven Hospital (YNHH) were determined through distributing company information on final manufacturing locations. Overseas transportation was assumed by cargo ship from the country of origin. North American transportation was assumed by truck to distribution centres and then to New Haven, CT. Bulk shipment considered 20 units per cardboard box, evenly attributed. All new handles and blades were separately packaged by the manufacturer in plastic film and paper. CSS-reprocessed reusable devices were repackaged at YNHH in peel-packs equivalent in materials, size, and weight to the original individual packaging. Thus, packaging was attributed to each SUD blade and SUD handle and to each use of the reusable tongue blade and reusable handle if treated by either HLD or STZ. LLD of the handle was historically performed in the operating room and without repackaging. **Use:** The rigid reusable stainless steel handle was powered by 2 alkaline C-batteries. These batteries were used until the laryngoscope light source appears weak or spent. Battery inputs were allocated proportionally to reflect conservative replacement every 40 uses. Similar use/reuse of 2 alkaline C-batteries was assumed for the SUD metal handle. The SUD plastic laryngoscope handle was powered by 3 embedded button-sized lithiumion batteries, discarded within the handle after a single use. **Reprocessing:** The Centers for Disease Control (CDC) requires that noncritical devices (those contacting intact skin) undergo a minimum of LLD, whereas sem- critical devices (those contacting mucous membranes or broken skin) undergo a minimum of HLD. Laryngoscope tongue blades were uniformly classified as semi-critical. For laryngoscope handles, there was inconsistent classification by professional bodies as either noncritical or semi-critical, and the CDC defers to manufacturer instructions for use (IFUs). Thus, reusable tongue blades and handles were both evaluated under HLD and STZ cleaning scenarios, and the reusable handle was also evaluated under the LLD scenario. CSS energy, chemical, and water requirements were determined through washer (Getinge 8666; Getinge Group, Englewood, CO) and autoclave (Getinge 833HC, Englewood, CO) specifications and were apportioned to each device assuming a full load (180 and 240 devices per tray, respectively). One quarter of a chemical wipe was allocated per handle LLD, as it was observed that a single cloth was used to clean additional surfaces (Supplemental Digital Content, Materials, http://links.lww.com/AA/C146). SUD handles and blades were assumed treated with HLD as part of the manufacturing process per original package labelling. **Disposal/End of Life:** After reaching the end of their useful lives, both SUD and reusable laryngoscope handles and blades, including packaging, entered waste management. Waste management modelling was performed using US average rates of recycling plastics (6%) and metals (30%–70%), while remaining solid waste is either landfilled (80%) or incinerated (20%). | LCA modelling was performed using the commercial software package, SimaPro 8.1 (Amersfoort, the Netherlands). Impact assessment was performed using the US Environmental Protection Agency's Tool for the Reduction and Assessment of Chemical and other environmental Impacts (TRACI) method. The primary environmental impact category of interest was global warming caused by greenhouse gas (GHG) emissions, expressed in carbon dioxide equivalents (CO2-eq). In addition to global warming, nine other standard environmental and human health impact categories were considered (measured in equivalents of respective reference compound). It was assumed that the values for device component weights and component contract costs applied to the entire population of devices (reflecting expected consistency in manufacturing). Therefore, neither sample-based statistical tests nor statistical analysis of model parameter uncertainty were appropriate. To test uncertainty in the results due to modelling assumptions, sensitivity analysis was undertaken through applying an alternate 100% recycling scenario to reflect health care facilities or communities that have aggressive recycling programs, and allowing the model to vary reprocessing parameters of time and device attrition to calculate break-even scenarios between reusable and SUD option. | 1 handle and 1 blade for a single patient encounter. | **SUD:** Raw materials, Laryngoscope materials production, laryngoscope manufacturing, packaging, transport/distribution, USE, SUD+ Packaging, solid waste management. **Reuseable Laryngoscope:** Raw materials, materials production+chemicals/fibres production, manufacturing of scope+clearning supply, fuel/electricity, reuseable | 'Cradle to grave' - extraction of material and energy resources, manufacturing, packaging, transportation, cleaning scenarios, and final disposal. LCC - procurement, reprocessing, refurbishment, and waste disposal, reflecting facility total cost of ownership (see Figure 1). | **Technical:** Data for reusable components were scaled per use based on rated lifetimes of each component, plus 1 cleaning, and were then compared to SUD alternatives **Geographic:** Waste management modelling was performed using US average rates of recycling plastics (6%) and metals (30%–70%), while remaining solid waste is either landfilled (80%) or incinerated (20%). The total recycling scenario demonstrated marginal reductions in GHG emissions over the standard waste disposal scenario for SUDs and had no significant impact on reusable device emissions (Figure 3). Total recycling of laryngoscope materials had no significant impact on costs at YNHH. Laryngoscope materials and energy and process inputs were matched with background life cycle inventory data from the ecoinvent v2.2 database adjusted for the US energy system. |
| Silcox 2023b | Y | Inventory analysis. LCA used to quantify the current usage and carbon footprint of telemedicine. | Ecoinvent database. Research paper (Holmner et al, 2014).(22) Information provided by a leading computer manufacturer's website. When a specific product’s LCA was not available, economic input–output LCA utilising known economic information to estimate associated emissions was used. A Monte Carlo Analysis (MCA) was used with 10,000 simulations to account for variability in the data. | Computer usage per telemedicine visit was determined as follows. The environmental impact for the production and end-of-life disposal of the computer devices was determined (leading manufacturer’s website). The total environmental impact of the manufacturing and end-of-life disposal of the devices used for telemedicine was then divided by the lifespan of the devices to determine the per-hour environmental impact of computer usage. This quantity was then multiplied by the telemedicine visit time to determine the device’s per visit environmental impact. The authors assumed that patients used their devices for five hours per week for three years, and the providers used their computers for five hours per day for four years. **Network data usage calculations:** Authors assumed that teleconference calls were routed through a single server. The environmental impact was calculated by adding internet usage and server usage. The GHG emission per kWh of electricity use was taken to be 0.6 kg CO2 -eq, the energy density of the internet was taken to be 0.64 kW/GB, the internet transfer rate was taken to be 5.5. Mbps and the wattage of the server was taken to be 0.6 kWh/server. **In person calculations:** round-trip travel distance data were collected retrospectively from a representative sample of patient visits in the third quarter of 2020. These data were fitted to several distributions to find the best-fit distribution, which produced a minimum error. It was found that a log-normal distribution best described the travel distance data. It was assumed that patients would travel the on- way distance to the clinic by car if the distance was less than 300 miles and by air, if the distance was 300 miles or greater. Based on the data from Holmner et al. (2014) and ecoinvent database, the authors determined that per kilometer of travel, a car produces 0.5 kgCO2-eq and a kilometer traveled by air produces 0.0245 kgCO2-Eq. Additional inputs for in-person visits included disposable items (e.g., paper handouts, exam table paper sheets, hand sanitiser) and durable medical equipment (DME, e.g., blood pressure cuff, weight scale, pulse oximeter, digital thermometer). | When a specific product’s LCA was not available, economic input–output LCA utilising known economic information to estimate associated emissions was used. Using these inputs, a Monte Carlo Analysis (MCA) was used with 10,000 simulations to account for variability in the data. MCA generates simulations based on a distribution of known data points. MCA was also used to simulate low and high endpoints of possible GHG emissions for various inputs to generate upper and lower bounds scenarios. | One preoperative visit | Scope of the LCA for telemedicine visits was defined as the full-patient visit from the start of the virtual encounter to its completion. Inputs used to determine emissions included the equipment used for video conferencing, energy requirements for broadband internet, and the length of the visit. For inperson visits, the scope encompassed patient travel to and from clinic as well as products used during the visit. Facilities-related inputs were excluded, such as hospital building electricity use, heating, ventilation, and cooling, as well as clinic electricity use as these are a part of the larger hospital structure and are not altered by telemedicine vs. in-person appointments. | Production, manufacturing, network usage and disposal (includ. disposable items and durable medical equipment used on in-person visit). | Assumptions made to define inputs. **Temporal:** assumed that the telemedicine conference call times followed a normal distribution; assumed that the patients used their devices for 5hrs per week for three years, and the providers use their computers for 5hrs per day for four years. **Technical:** assumed that patients used any of the following devices for their telemedicine session: desktop, laptop, tablet, or phone. The provider was assumed to have used a desktop computer (observed as standard during telemedicine visits). **Geographical:** round-trip travel distance data were collected retrospectively from a representative sample of patient visits in the third quarter of 2020. It was assumed that patients would travel the one-way distance to the clinic by car if the distance was less than 300 miles and by air, if the distance was 300 miles or greater. |
| Sorensen 2018 | Y | 'Simplified' LCA (only 2 impact categories measured (i) Greenhouse gas emissions (expressed as CO2 eq) & (ii) loss of scarce resources). This simplified methodology assessed the ‘embodied energy’ of the materials included in a product or cleaning operation, and the additional energy used to manufacture and use the product. The methodology also assessed the potential embodied energy recovered by recycling of the materials at end-of-life or the energy recovered by incineration of the product at end-of life. | American National Standards ST91: 2015 Flexible and semirigid endoscope processing in health care facilities prepared by the Association for the Advancement of Medical Instrumentation. The approach is similar to that using the ISO 14040/44 standards for LCAs but is not fully compliant with the standard, as the LCA technique has been used to produce information on only part of the lifecycle for the reusable bronchoscopes (RBs). | Ambu A/S collected the data used for the analysis of cleaning and disinfection of RBs. Rigshospitalet, the University Hospital of Copenhagen, Denmark, collected samples of the materials used for protective wear. The reprocessing procedure of a RB and materials used at the University hospital were in accordance with the American National Standards ST91: 2015 Flexible and semirigid endoscope processing in health care facilities prepared by the Association for the Advancement of Medical Instrumentation. Data for consumptions for washing and drying of RBs originated from data sheets for the cleaning systems from three different suppliers. Ambu A/s provided the data on material composition and amounts for the Ambu ® aScopeTM 4 broncho (FORCE Technology, 2017). Weighing of the materials was done on a Mettler Toledo PG5002-S Delta Range with a resolution of 10 mg. | Analysis focused on materials and energy used for the cleaning and sterilisation of RBs. **PPE:** changed between each reprocessing cycle and when moving from the decontaminated area to the clean area. **Cleaning & Sterilisation:** three disinfectant wipes were included; 40 ml of Sekusept for pre-cleaning and as an intercept detergent (Rapicide A and B) for use in the automated bronchoscope reprocessor; isopropyl alcohol 70% is included for disinfection. The end-of-life fate included in this analysis assumed recycling of all recyclable materials and incineration with energy recovery of auxiliary materials. The assessment assumed that the typical disposal pattern for disposal of the single-use bronchoscopes was incineration together with the inner packaging due to hygiene requirements. The consequence was that the heat value of the plastic and paper/cardboard would be credited in the assessment. The metals would be lost in the ash. The reference system included the use of RBs until discarding them. After cleaning, an RB must be brought from a washer to a dryer/storage cabinet in a clean environment with the operator wearing one set of protective equipment such as an apron, protective shoes, gloves, etc. (see table 1). After using the RBs many times (number of times unknown) they were discarded. | Results for RBs based on only one bronchoscope being cleaned per cleaning operation and thereby using one set of PPE per RB* | Material and energy use for producing scopes; materials used and the composition of the PPE and washing agents used for reprocessing the RBs; energy used for washing and drying the RBs. The materials contributions and their end-of life fate was not considered and analysis did not include manufacturing of the screen needed to use the Ambu® aScopeTM 4 broncho. | **Reuseable scopes:** Material and energy used for producing PPE; material and energy used for producing reuseable scopes not included in assessment, but included for disposable scopes; washing, drying and storing, incineration of material waste for energy recovery, material waste for reuse. **Disposable scopes:** Material and energy use for producing scopes; incineration of material waste for energy recovery/material waste for reuse. | **Technical:** The monitoring of materials included in the analysis for cleaning a RB was based on current practice at Rigshospitalet, the University Hospital of Copenhagen, Denmark. The procedure was comparable to that of the American National Standards ST91: 2015 Flexible and semirigid endoscope processing in health care facilities prepared by the Association for the Advancement of Medical Instrumentation. |
| Stripple 2008 | Y | LCA and a system perspective. Three different LCA models were designed for the raw catheters of the three different plastic materials. Evaluation and valuation of the results have been performed both with a direct inventory data comparison, such as a comparison of energy use and formed emissions, and by using the following four impact assessment methods. (i) A comparison after classification and characterisation in line with the environmental product declaration (EPD) system. (ii) An impact assessment according to the Eco-indicator 99 system. (iii) An impact assessment according to the CML 2 system. (iv) An impact assessment according to the EPS 2000 system. | Used weighting methods which are only included in the EN ISO 14044:2006 standard as an option and not recommended for comparative studies. In this regard, the ISO standard was not followed. | The most common catheter size was selected for the analysis; a 40 cm long, Nelaton tip, charrière 12. Typical catheter use during treatment was five catheters per day, which amounted to 1825 catheters in one year. Accordingly, the model covered the life cycle of 1825 catheters. LCA models for the raw catheters of the three different plastic materials were developed. An internal independent expert review has been performed by Lars-Gunnar Lindfors (IVL) of the LCA models. In the review report it was concluded that the LCA models were developed according to good practice and that the results were balanced and supported by the models. The different plastic materials also had different densities, which gave the catheters different weights. Two different waste handling methods were considered; incineration (40% of the plastics) and landfill (60% of the plastics). This can reflect an average of the present situation in the OECD countries. For the landfill process calculations, an infinite time period was used, i.e. the material in the landfill was broken down completely and all the emissions released which can take several 100 years. Recycling of plastic materials from medical devices is not possible, due to the risk of infection. For destruction of medical devices, incineration is preferred. The energy released during waste incineration or the production and combustion of methane from landfills is, as an example, treated as the corresponding gain in energy resource use and emissions when the same amount of energy is supplied by a fuel oil boiler. For electric power production, an OECD power production mix was used. Transport distances related to the application were assumed to be equal for the different materials and carried out by heavy diesel trucks. However, for specific inventory data such as production of different plastic materials, specific data for electric power production and transports were used. | The first impact assessment method to be applied was a pure classification and characterisation in line with the EPD system. The Eco-indicator 99 hierarchist impact assessment method - normalisation and weighting methods were applied and the weighting method used was based on the panel procedure with the following weighting factors: human health 40%, ecosystem quality 40% and resource use 20%. No standard weighting method has been developed for the CML 2 method. However, Huppes et al (2007) have developed a weighting method that can be used with the CML 2 method.(23) This weighting method is based on a panel procedure developed in the Netherlands. This weighting method does not include resource use. A similar weighing approach as in Eco-indicator 99 has been used. In this case, the weighting factors have been scaled so the resource use has a weighting of 20% and the remaining, existing weighting stands for 80%. | The actual function of a hydrophilic urinary catheter is to be part of a urological treatment and to be the tool in catheterisations. With single-use catheters, one catheter is used for each catheterisation. One catheter is thus identical to one catheterisation, and the amount of catheters can thereby be used as a measure of the functional unit. In this case, the functional unit for the entire model was chosen as the treatment of a patient with catheters during one year. | Focused only on the selection of plastic materials for the uncoated catheters that are later coated to become hydrophilic (the catheter tube and the connector are assumed  to be of the same type of material). Extraction of raw materials, production of the plastic material, production of the raw catheter, transports, waste handling and energy recycling of the plastic materials. Other processes, i.e. packaging, hydrophilic coating and sterilisation were considered equal for the different materials and are not considered. | LCA system - extraction of raw materials, production of the plastic material, production of the raw catheter, transports, waste handling and energy recycling of the plastic materials. | **Geographic:** Two different waste handling methods were considered; incineration (40% of the plastics) and landfill (60% of the plastics). This can reflect an average of the present situation in the OECD countries. **Technical:** The data module for incineration (Simonson et al, 2000) covered incineration in a municipal solid waste (MSW) incinerator. The data were based on modern OECD standard equipment with well-controlled exhaust gas cleaning equipment. Controlled HCl emissions were assumed with an emission level equivalent to 3% of total formed HCl. The incinerator was also assumed to be connected to a district heating system for energy recovery. The inventory data describing the system in terms of resource use, emissions, etc. were relatively comprehensive and included many different parameters with different environmental impacts. In this study, normalisation was carried out from a Western European perspective. |
| Thiel 2015 | Y | Hybrid LCA framework developed for analysing infant birthing procedures by incorporating process LCA data and Economic Input Output LCA (EIO-LCA) data. | Research papers (e.g. Shrake et al, 2012; Campion et al, 2012).(24, 25) Carnegie Mellon University Green Design Institute. Economic input-output life cycle assessment (EIO-LCA) US 2002 (428) model. http://www.eiolca.net AICS (North American Industry Classification System) sector 339112 Surgical and Medical Instrument Manufacturing, and sector 562000: Waste Management and Remediation Services. | Waste audits and site assessments were conducted. Detailed waste audits of 62 cases of hysterectomy were conducted (15 each abdominal, vaginal, and robotic, and 17 laparoscopic). The audits involved data collection from individual patients’ medical cases. Waste audits were conducted over the course of 1 year, with the target goal of auditing the waste from at least 15 of each type of hysterectomy so that variability in material use could be included in the Monte Carlo Analysis. Immediately following the surgery, the municipal solid waste (MSW) and recycling was collected, labelled with the case identification number, and moved to a secure storage location for sorting and weighing. Regulated Medical Waste (RMW), which undergoes autoclaving prior to landfilling, was estimated by quantifying the type of “peel packs” or package labels found  in the MSW. Chemo/Pathogenic waste was calculated using uterine weights, as described in the patient records. Quantities of anaesthesia and abdominal insufflation were calculated from patient records. Unit processes were assigned to each data, with preference given first to U.S. based databases, i.e., USLCI29 and then the most robust database, i.e., ecoinvent. Impacts due to the transportation of material wastes were calculated using distances from the hospital to the landfill and recycling facilities based on waste hauling quantity data provided by Magee’s facility management. Impacts due to production and disposal of reusable linens were allocated based on the estimated lifespan of each linen type. Reusable stainless steel instruments were estimated to have a lifespan of 300 uses, based on a study of reusable surgical instruments (McGain et al, 2012). Previous literature was used to supplement gathered data on the sterilisation process for reusable materials and linens. Characterisation factors for the global warming potential of anaesthetic gases were taken from previous literature. | Monte Carlo simulations were used to quantify the variability and uncertainty in emissions for each component of a hysterectomy. Environmental impacts from the inputs and outputs of the four types of hysterectomy were calculated using TRACI 2.1 for both process LCA and EIO-LCA. Unit conversion was necessary to match the EIOLCA results in impact categories Acidification, Carcinogenics, NonCarcinogenics, and EcoToxicity with the process LCA results (Supporting Information). Embodied energy, or a summation of all energy used during the material’s life cycle, was calculated using cumulative energy demand (CED) version 1.08 developed by ecoinvent version 2.0 and PRéConsultants for process LCA and the energy analysis function found on the EIO-LCA online tool. Monte Carlo Analysis (MCA), or random number sampling, was used to account for the uncertainty inherent in life cycle inventory data and the variability of material and energy consumption for each type of hysterectomy at the hospital. Distributions of individual material processes in each type of hysterectomy were calculated from waste audit data using the Anderson−Darling test. The resulting distribution was calculated from 100 000 random samplings. The 5th, 50th, and 95th percentiles, as well as the means and standard deviation for all impact categories, were reported for each hysterectomy as a whole. The impacts due to recycling, because they were negative, were not included within the MCA, but were incorporated as averages in post-MCA results. MCA results are included as error bars in result figures. | One hysterectomy | Boundaries encompass the raw material extraction, production, use, and end-of-life of the processes and products required to perform each type of hysterectomy from the moment the patient enters the OR to the moment she leaves the OR. Products and processes encompass energy and materials associated with surgical instruments and sterilisation. Not included: manufacturing of building materials and use of hot water and chemical manufacturing associated with drugs/anaesthesia and making of/use of cleaning products. | Production, use/reuse, end of life. | **Technical:** All database selections were determined by comparing the physical description and application of the material to the unit process description. Certain unit processes were modified based on literature to more accurately reflect the product or process being represented. The USLCI electricity process was modified to match the energy mix of Pennsylvania for 2012.31. Disposable gowns, drapes, and bluewrap from the OR are a type of polypropylene fabric also known as spunbond-meltblown-spunbond or SMS PP. As this material makes up a large portion of a hysterectomy’s waste stream by weight, the USLCI unit process for PP production was modified to include the manufacture of the textile beyond pelletisation of the plastic. **Temporal:** All monetary values were converted from 2012 U.S. dollars to 2002 dollars, the basis for the most recent EIO-LCA model. |
| Thiel 2018 | Y | Hybrid LCA framework | Research papers (e.g. Thiel at al, 2015). Carnegie Mellon University Green Design Institute. Economic input–output life cycle assessment. 2013. the Ecoinvent life cycle inventory database. SimaPro PhD7.3.3 by Pré Consultants. Impact assessment method TRACI 2.1 from the US Environmental Protection Agency. | **Anaesthetics**: The baseline calculation was an average of the combination of anaesthetic approaches used in each of the 17 laparoscopic hysterectomies from the original study (Thiel et al, 2015).(26) To model anaesthetic interventions, the authors assumed an average anaesthetic duration of 150 minutes and calculated the average GHGs resulting from the use of desflurane alone (intervention A1), desflurane with N2O (A2), sevoflurane with N2O (A3), and sevoflurane alone (A4). It should be noted that N2O, in these cases, was used only for an average of 16 minutes during the 150-minute surgery. They also modelled the GHG emissions from the replacement of all inhaled anaesthetics with propofol (A5), although in the original study, propofol was used only during vaginal hysterectomy. **Surgical Materials:** These GHG estimates from the Economic Input Output Life Cycle Assessment database, which estimated the emissions on the basis of the price, in this case $0.21 per pound of regulated medical waste. Cotton towels (M3) were assumed to have a 10-use life span, and third-party linen laundering was assumed to be the sterilisation pathway. Although life spans may be shorter or longer, this was the life span hospital staff estimated. The estimates of energy and detergent use were from Thiel et al (2015).(26) Reusable gowns and laparotomy drapes (M4) had an estimated life span of 75 uses and were sterilised between cases with laundering, drying, and autoclaving cycles, per manufacturer recommendations. In the estimates of emissions, the sterilisation process was assumed to be in-house (therefore, there are no off-site transportation emissions). Re-processable SUDs (M5) were identified as the surgical instruments UPMC’s current third-party re-processor can accept. These include endoshears (Medtronic, North Haven, CT), Carter-Thomason CloseSure System (Medline, Mundelein, IL), Versa-Port plus v2, 5 to 12 millimeter (Medtronic), LigaSure blunt tip laparoscopic sealer–divider 5 millimeter blunt tip laparoscopic sealer (Medtronic), LigaSure (Medtronic), and LigaSure Vessel Sealing 5 millimeter (Medtronic). Emissions from reprocessing were estimated using values from previous literature. A panel of 3 practicing gynaecologists at UPMC determined a list of the bare essentials of surgery which included a uterine manipulator, a monopolar shears, a vessel sealer, a grasper, laparoscopic suturing equipment, suture, ports, and an insufflator. The environmental impacts from these single-use instruments were calculated using their purchase prices and the Economic Input Output Life Cycle Assessment database. The authors assumed these supplies were single-use disposables (although reusable supplies do exist for some of these items) and that the original disposable custom pack (with single-use surgical supplies for laparoscopic hysterectomy) was still in use. The gynaecologist panel reported using this minimal supply set in at least one third of their laparoscopic cases. **Energy:** From the17 cases surveyed in Thiel et al (2015),(26) the authors estimated the average duration of a laparoscopic hysterectomy at 170 minutes, with 8 kilowatt-hours of electricity use from OR equipment and machines, 0.5 kilowatt-hours from lighting, and 37 kilowatt-hours from the heating, ventilation, and air-conditioning system. This heating, ventilation, and air-conditioning system also used natural gas, averaging 2.2 cubic meters of gas per average case. Low-use periods (E1) for the ORs include nights (estimated at 8 hours) and weekends (56 hours) for a total of 96 hours per week (56% of the week) in energy-saving mode. The American Society of Heating, Refrigerating and Air-Conditioning Engineers advocate this approach to cooling off during OR low-use periods. UPMC uses the Pennsylvania power generation mix, which at the time of the 2014 study (Thiel et al, 2015) was 73% coal based. The proposed electrical power (available through PG&E Corp Energy Company, San Francisco, CA; E2) is 0.00% coal, 2.20% oil, 35.70% nuclear, and 62.10% hydro, with GHGs averaging 0.02 kilograms CO2 per kilowatt-hour.(26) | The life cycle GHGs or carbon footprint of these interventions was calculated using the same hybrid life cycle assessment framework from Thiel et al (2015) including obtaining data from Economic Input Output Life Cycle Assessment, the Ecoinvent life cycle inventory database, SimaPro PhD 7.3.3 by Pré Consultants, and the impact assessment method TRACI 2.1 from the US Environmental Protection Agency. | NR | NR | Use/reuse, disposal (not for all interventions). | **Geographical:** UPMC uses the Pennsylvania power generation mix, which at the time of the 2014 study (Thiel et al, 2015) was 73% coal based. The electrical power mix proposed (available through PG&E Corp Energy Company, San Francisco, CA; E2) is 0.00% coal, 2.20% oil, 35.70% nuclear, and 62.10% hydro, with GHGs averaging 0.02 kilograms CO2 per kilowatt-hour. The GHGs were calculated using one study location and differences between facilities may exist which could result in different percentages of savings in carbon footprint when enacted. **Technical:** The list of proposed interventions is not exhaustive and does not include, for example, minimising leaks or poor flowrate in anaesthetic delivery, installing anaesthetic gas-capturing devices, reformulating the disposable custom pack of surgical supplies, switching to other reusable supplies such as hard plastic or stainless steel wash bins, utilising reusable canisters for surgical instrument trays, or scheduling surgeries more efficiently to reduce OR idle time. Only the affects of drugs inhaled by patients were included and other pharmaceuticals may also affect the GHG footprint. |
| Thiel 2023 | Y | LCA | LCI database Ecoinvent v3.82. US Environmental Protection Agency’s TRACI 2.1 v1.06/ US 2008 (Tool for Reduction and Assessment of Chemicals and Other Environmental Impacts). LCA was conducted in 4 steps, according to ISO 14040 standards. | **In person visits:** patient transport to and from clinic; energy used in heating, ventilation, and air conditioning (HVAC) and lighting the exam room; and the supplies used, and waste generated, including PPE worn by clinicians (assumed to be one surgical mask per 10 patient visits), one paper-based exam table cover, one pump of hand sanitiser, and a sanitising wipe. **Virtual visits:** electricity needed to power a cellular phone for a call or the power needed for running video conference software; the electricity use of the clinician, who (it is assumed) joins the virtual visit through a desktop computer in the clinic with HVAC and lighting but the size of space and the energy usage is difficult to determine**. Calculations excluded:** energy needs of clinicians' space and energy needs of patient's space; commuting of staff for either visit, given the difficulties of allocating a staff member’s entire commute to a single visit. This is especially challenging for the virtual visit, where a clinician may not be commuting at all. All virtual visits were assumed to be appropriate, meaning that they did not convert to an in-person visit. Data were collected on the number of on the number of in-person, phone, and video visits across SHC, by department, from 2019 through 2021. These data included the number of visits to each department and the duration of each visit in minutes (see SI Table 2). The average distance between the patient’s ZIP code and the ZIP code of the clinic visited was calculated. Any one-way distance greater than 250 miles (402 km) was assumed to be taken via airplane, while anything less than this value was assumed to be travelled by passenger car. The round-trip distance per patient was used to estimate emissions.  A list of basic supplies typically found in more patient visits was generated by the study team. These included one surgical mask, worn by the care provider for an estimated 10 cases; a serving of hand sanitiser; tissue to cover the exam table; and a sanitising wipe to clean the exam room after the patient’s visit. The material components of each product were directly measured or estimated from literature. All products were assumed to be manufactured in China and shipped approximately 3000 km by boat. A 40 km distance for distribution by freight truck was estimated before arrival at the clinic. All products were disposed in a sanitary landfill approximately 40 km from the clinic by truck. Distances varied by the exact clinic location so an average was assumed.  SHC’s engineering team provided record of electricity, gas, steam, and chilled water consumption across SHC’s clinics in 2020. These values were divided by the total surface area of the clinical space represented in the data and the number of minutes in a year (assumed to be 525,600 though this may have artificially reduced the actual energy intensity of a clinical visit, as it assumed lights and equipment were drawing electrical power after hours. This ‘energy intensity’ was then multiplied by the floor area of an exam room, assumed to be a 10' × 10' 9.3m2), space (or and the duration of the clinic visit, as captured in the medical records. As some buildings consumed varying quantities of energy, these ranges were included in one of the sensitivity analyses. | The LCA 9.3.0.240 v3.82 software SimaPro was used to estimate the life cycle emissions of clinic visits. The amount of GHGs NOT emitted as a result of virtual visits were estimated by estimating the GHGs of these visits, had they been in person. This would therefore add transportation of the patient, following the assumptions for distance and mode, supplies production and disposal, and a shift in the amount of electricity used during the visit, which was done by assuming the number of minutes on the phone or video call would instead be the number of minutes spent consuming the average energy in a 10′ × 10′ exam room. | One clinic visit, either in-person or virtually. | In-person visits: patient transport, energy, supplies used, waste generated. Virtual visits: electricity, disposal and transport. | Production, use, disposal and transport. | **Technological:** Exact data were not collected on the mode or distance travelled by patients. The average distance between the patient’s ZIP code and the ZIP code of the clinic visited was calculated. Any one-way distance greater than 250 miles (402 km) was assumed to be taken via airplane, while anything less than this value was assumed to be travelled by passenger car. |
| Winklmair 2023 | Y | Inventory analysis | Grey literature (manufacturer information). Fifth Assessment Report (Fontaras & Samaras, 2010).(27) | The different materials of the individual cataract package components of 3 Austrian hospitals (Hanusch Krankenhaus Wien, Barmherzige Briider Wien and Privatklinikk Hochrum) were separated (for example, plastic from paper packaging), weighed, and categorised based on their properties. Grey literature was searched for manufacturer information on the product components and clear information on material was unobtainable, an assessment was made based on specific characteristics. In uncertain cases, the worst material from an eco-balance perspective was used for the life-cycle analysis calculation. Based on these data, the global warming potential (GWP) of each product is calculated using the GaBi Product Sustainability and Performance software. The environmental effects assessed using this software underlie the Environmental Footprint 3.0 (EF 3.0) methodology, developed by the European Commission. In this report, the focus was on the carbon footprint and the respective EF 3.0 category “EF 3.0 Climate Change—total” considering the effects on climate change from fossil greenhouse gas emissions and removals, biogenic methane emissions, and carbon emissions from land use and land use change. The EF 3.0 Climate Change—total was based on the current IPCC characterisation factors taken from the Fifth Assessment Report (Fontana & Samaras, 2010) for a 100-year time frame (GWP100), which is currently the most used metric for climate change analyses.(27) | Cataract package sales lists were provided by the 3 main Austrian cataract package suppliers (estimated 94% market share in 2021). For the analysis, the products were divided into categories of surgical gowns, cannulas, syringes, postoperative eye protection, surgical trays/bowls, knives, fluid management (stick swabs, compresses, cotton balls), drapes/covers, and packaging for ease of reference. A mean weight and carbon emissions of each product type was calculated, which was then multiplied by the sales figures of the cataract packages from the 3 suppliers. This resulted in a representative weight and CO, balance representing 94% of all cataract packages used throughout Austria. Particular attention was paid to the quantities and size variations between the varying cataract package compositions. | NR | Some disposables (e.g. cassette and tubing) and external factors, such as hospital electricity consumption (scope 2 emissions), transport of staff and patients to and from the hospital, pharmaceuticals used before and in the operating room (OR), and other consumption outside the cataract OR, were not included in the emission calculation. Included cataract package materials used to calculate weight and greenhouse gas were: Products - Knives (Phaco knives, Paracentesis knives), Cannulas (18-30 gauge), Fluid management (stick swabs, compresses, cotton balls), Surgical gowns (M/L/XL), Postoperative eye cover, Surgical trays/bowls, Syringes, Drapes and covers (Body drape, Back table cover/wrapping drape, armrest covers, Multipurpose drape if listed separately). Packaging: Packaging Phaco and Paracentesis knife, Cannula cover/packaging, Bag for swabs, compresses, syringes etc, Cataract kit bag with product list. Excluded data: (products and associated packaging (not separately listed): surgical gloves and ophthalmic viscoelastic device, Medication and infusion solution with cutlery, Phaco cassettes and tubing, Bag for swabs, compresses, syringes etc, Individually occurring single-use surgical instruments (disposable clamps, forceps, manipulator, chopper, shears, hooks etc). | N/A | **Technical:** Where it was not possible to obtain clear information on the materials, an assessment was made based on specific characteristics. In uncertain cases, the worst material from an eco-balance perspective was used for the life-cycle analysis calculation. **Geographical:** the Environmental Footprint 3.0 (EF 3.0) methodology, developed by the European Commission underpin the software used in the analysis. |
| Wombwell 2023 | Y | Inventory analysis | Research papers. Standardised CO2 Protocol (Hogan et al, 2022). Soluscope: Series 3 User Manual. | A simplified lifecycle analysis was performed to compare the carbon footprint of the Ambu aScopeTM 4 Cysto System with Olympus CYF-VH reusable flexible cystoscope. Detailed manufacturing information was obtained from the sustainability team at Ambu®, outlining the composition of the flexible cystoscope. This information was used to calculate the manufacturing carbon footprint of the cystoscopes, following a standardised CO2 protocol (Hogan et al, 2022). The respective amount of CO2 per kilogram produced from manufacturing the raw materials included: 6 kg of CO2/kg of plastic, 1.16 kg of CO2/kg of rubber, 1.8 kg CO2/kg of steel and 150 kg of CO2/kg of electronics. The Ambu® cystoscope weighs 159 g, and the composition is 95.3% plastic, 3.3% steel, 1.1% electronics and 0.4% rubber. In total, 7.4 kg of CO2 is produced per kilogram of Ambu® cystoscope manufactured, meaning that the total CO2 produced per cystoscope manufactured was 1.18 kg CO2. The Ambu® cystoscope is manufactured in Penang, Malaysia and requires transportation to Sydney, Australia, via shipping freight. On average, the CO2 produced by shipping freight is 26.5 g of CO2 per tonne-kilometre. The journey is 9540km, which equates to 252 kg of CO2/tonne. The total weight of the shipped product (including its packaging) is 353g; therefore, the CO2 produced by transporting each cystoscope is 0.09kg CO2. The Olympus cystoscope weighed 0.870 kg, thereby giving a manufacturing footprint of 0.02 kg CO2. The average lifecycle of the Olympus cystoscope obtained from Olympus, which is 7 years, was included in the manufacturing carbon footprint calculations alongside the 3920 average uses per lifecycle. A detailed breakdown of the reusable scope into its component parts was unobtainable from Olympus so the calculation was based on the average manufacturing cost per kilogram of cystoscope manufactured as above. The expected footprint from cleaning and sterilising the Olympus cystoscopes using the Soluscope 3 automatic endoscope re-processor was calculated, including the use of a new set of personal protective equipment (PPE) for each scope, needing to be worn by the technician sterilising the equipment. The carbon footprint of a single-use gown is 0.9 kg CO2 and a pair of sterile gloves is 0.05 kg CO2 – as per the protocol in the centre, a new pair of gloves and gown must be used for each cystoscope sterilised. A power meter was used to obtain an accurate power consumption per cycle of re-processing. The ultimate disposal of the scopes was added to give a total carbon footprint; cystoscopes cannot be recycled in Australia and must be disposed of as medical waste, requiring incineration and ultimately landfill. |  | A power meter was used to obtain an accurate power consumption per cycle of re-processing. | Olympus did not provide a detailed breakdown of the reusable scope into its component parts and so the calculation was based on the average manufacturing cost per kilogram of cystoscope manufactured as above. The chemical waste generated from the re-processing of the reusable cystoscope was not included, nor was the manufacturing, transport and solid waste footprint of the Soluscope 3 automatic endoscope re-processor. | Manufacturing, transportation, cleaning/sterilisation, reprocessing, disposal. | **Technical & Geographical:** Due to Australian environmental regulations, cystoscopes cannot be recycled and must be disposed of as medical waste, requiring incineration and landfill. |

^a^No detail is given re this in the paper. From google, it would seem to be a circular economic model..*Assumed as not clearly reported in paper. ** No longer available online.

# References for datasets cited in included studies

1. Transport Df. National travel survey: 2006. National Statistics, 2007. (Transport statistics bulletin). In: Transport Df, editor. 2006.

2. Centre THaSCI. National Kidney Care Audit Patient Transport Survey Report. 2010. 2010.

3. Defra/DECC. 2009 Guidelines to Defra/DECC’s GHG Conversion Factors for Company Reporting. In: DECC D, editor. 2009

4. Bath IoCaEIUo. Inventory of Carbon and Energy (ICE) database version 1.6a. . 2009.

5. purchasing Cfeb. Market Review: Home Haemodialysis Services. CEP 10061.

6. Ansell D. Missing facts, different countries. BMJ. 2008;336(7637):172-.

7. U.S. Renal Data System. U.S. Renal Data System U. Annual Data Report: Atlas of End-Stage Renal Disease in the United States. . Bethesda; 2007.

8. Stutz M. Carbon Footprint of a Typical Business Laptop From Dell 2010.

9. Juerg. Plastic bags and plastic bottles - CO2 emissions during their lifetime. Time for change 2009 [Available from: <https://timeforchange.org/plastic-bags-and-plastic-bottles-co2-emissions-during-their-lifetime/>.

10. Dayaratne SP, Gunawardana KD. Carbon footprint reduction: a critical study of rubber production in small and medium scale enterprises in Sri Lanka. Journal of Cleaner Production. 2015;103:87-103.

11. DeRosier J, Stalhandske E, Bagian JP, Nudell T. Using health care failure mode and effect analysis™: the VA National Center for Patient Safety’s prospective risk analysis system. The Joint Commission journal on quality improvement. 2002;28(5):248-67.

12. MacNeill AJ, McGain F, Sherman JD. Planetary health care: a framework for sustainable health systems. The Lancet Planetary Health. 2021;5(2):e66-e8.

13. Defra/DECC. Guidelines to Defra/DECC’s GHG Conversion Factors for Company Reporting: Methodology Paper for Emission Factors. 2011.

14. Davis NF, McGrath S, Quinlan M, Jack G, Lawrentschuk N, Bolton DM. Carbon footprint in flexible ureteroscopy: A comparative study on the environmental impact of reusable and single-use ureteroscopes. Journal of Endourology. 2018;32(3):214-7.

15. Ong D, Moors T, Sivaraman V, editors. Complete life-cycle assessment of the energy/CO2 costs of videoconferencing vs face-to-face meetings. 2012 IEEE Online Conference on Green Communications (GreenCom); 2012: IEEE.

16. association W. Steel's contribution to a low carbon fture and climate resilient societies: worldsteel position paper. 2014.

17. Huijbregts MA, Steinmann ZJ, Elshout PM, Stam G, Verones F, Vieira M, et al. ReCiPe2016: a harmonised life cycle impact assessment method at midpoint and endpoint level. The International Journal of Life Cycle Assessment. 2017;22:138-47.

18. Kasivisvanathan V, Rannikko AS, Borghi M, Panebianco V, Mynderse LA, Vaarala MH, et al. MRI-targeted or standard biopsy for prostate-cancer diagnosis. New England Journal of Medicine. 2018;378(19):1767-77.

19. Jarvis I VF. Operating room ventilation systems best practices guide for energy efficiency, health and safety. 2017.

20. De Gialluly C, Morange V, De Gialluly E, Loulergue J, Van der Mee N, Quentin R. Blood pressure cuff as a potential vector of pathogenic microorganisms a prospective study in a teaching hospital. Infection Control & Hospital Epidemiology. 2006;27(9):940-3.

21. Bare J. TRACI 2.0: the tool for the reduction and assessment of chemical and other environmental impacts 2.0. Clean Technologies and Environmental Policy. 2011;13:687-96.

22. Holmner A, Ebi KL, Lazuardi L, Nilsson M. Carbon Footprint of Telemedicine Solutions - Unexplored Opportunity for Reducing Carbon Emissions in the Health Sector. Plos One. 2014;9(9).

23. Huppes G, Davidson M, Kuyper J, Van Oers L, de Haes HU, Warringa G. Eco-efficient environmental policy in oil and gas production in The Netherlands. Ecological Economics. 2007;61(1):43-51.

24. Shrake SO, Thiel CL, Landis AE, Bilec MM. Life cycle assessment as a tool for improving service industry sustainability. IEEE Potentials. 2012;31(1):10-5.

25. Campion N, Thiel CL, DeBlois J, Woods NC, Landis AE, Bilec MM. Life cycle assessment perspectives on delivering an infant in the US. Science of the total environment. 2012;425:191-8.

26. Thiel CL, Eckelman M, Guido R, Huddleston M, Landis AE, Sherman J, et al. Environmental impacts of surgical procedures: Life cycle assessment of hysterectomy in the United States. Environmental Science and Technology. 2015;49(3):1779-86.

27. Fontaras G, Samaras Z. On the way to 130 g CO2/km—Estimating the future characteristics of the average European passenger car. Energy Policy. 2010;38(4):1826-33.

# References for studies included in review

1. Andrew N, Barraclough KA, Long K, Fazio TN, Holt S, Kanhutu K, et al. Telehealth model of care for routine follow up of renal transplant recipients in a tertiary centre: A case study. Journal of telemedicine and telecare. 2020;26(4):232-8.

2. Arndt E-M, Jansen TR, Bojko J, Roos JJ, Babasiz M, Randau TM, et al. COVID-19 measures as an opportunity to reduce the environmental footprint in orthopaedic and trauma surgery. Frontiers in surgery. 2023;10:959639.

3. Asghari M, Al-e-Hashem S. A green delivery-pickup problem for home hemodialysis machines; sharing economy in distributing scarce resources. Transportation Research Part E-Logistics and Transportation Review. 2020;134.

4. Baboudjian M, Pradere B, Martin N, Gondran-Tellier B, Angerri O, Boucheron T, et al. Life Cycle Assessment of Reusable and Disposable Cystoscopes: A Path to Greener Urological Procedures. European Urology Focus. 2022.

5. Bendine G, Autin F, Fabre B, Bardin O, Rabasco F, Cabanel JM, et al. Haemodialysis therapy and sustainable growth: a corporate experience in France. Nephrology, dialysis, transplantation : official publication of the European Dialysis and Transplant Association - European Renal Association. 2020.

6. Beswick DM, Vashi A, Song Y, Pham R, Holsinger FC, Rayl JD, et al. Consultation via telemedicine and access to operative care for patients with head and neck cancer in a Veterans Health Administration population. Head Neck-J Sci Spec Head Neck. 2016;38(6):925-9.

7. Betts. A GREENER ENDOSCOPY UNIT FOR ROYAL CORNWALL HOSPITAL. 2022 [

8. Bird. Changing the 3 month blood test postage kits for patients on the renal transplant register 2022 [

9. Boberg L, Singh J, Montgomery A, Bentzer P. Environmental impact of single-use, reusable, and mixed trocar systems used for laparoscopic cholecystectomies. PLoS ONE. 2022;17(7 July):e0271601.

10. Bond A, Jones A, Haynes R, Tam M, Denton E, Ballantyne M, et al. Tackling climate change close to home: mobile breast screening as a model. Journal of Health Services Research & Policy. 2009;14(3):165-7.

11. Burton. Sustainable ENT: Fractured Nose Manipulation - Local Anaesthetic Pathway. 2022 [

12. Buttner L, Posch H, Auer T, Jonczyk M, Fehrenbach U, Hamm B, et al. Switching off for future-Cost estimate and a simple approach to improving the ecological footprint of radiological departments. European Journal of Radiology Open. 2021;8:100320.

13. Chambrin C, de Souza S, Gariel C, Chassard D, Bouvet L. Association Between Anesthesia Provider Education and Carbon Footprint Related to the Use of Inhaled Halogenated Anesthetics. Anesthesia and Analgesia. 2023;136(1):101-10.

14. Chan. Reducing carbon (CO2E) waste from pulse lavage systems used in joint replacement surgery, orthopaedic theaters 2023 [

15. Chen M, Zhou R, Du C, Meng F, Wang Y, Wu L, et al. The carbon footprints of home and in-center peritoneal dialysis in China. International Urology and Nephrology. 2017;49(2):337-43.

16. Cheung R, Ito E, Lopez M, Rubinstein E, Keller H, Cheung F, et al. Evaluating the Short-term Environmental and Clinical Effects of a Radiation Oncology Department's Response to the COVID-19 Pandemic. International Journal of Radiation Oncology Biology Physics. 2023;115(1):39-47.

17. Chuter R, Stanford-Edwards C, Cummings J, Taylor C, Lowe G, Holden E, et al. Towards estimating the carbon footprint of external beam radiotherapy. Phys Medica. 2023;112:8.

18. Connor A, Lillywhite R, Cooke MW. The carbon footprints of home and in-center maintenance hemodialysis in the United Kingdom. Hemodialysis International. 2011;15(1):39-51.

19. Connor A, Mortimer F, Higgins R. The follow-up of renal transplant recipients by telephone consultation: Three years experience from a single UK renal unit. Clinical Medicine, Journal of the Royal College of Physicians of London. 2011;11(3):242-6.

20. Connor MJ, Miah S, Edison M, Brittain J, Kondjin Smith M, Hanna M, et al. Clinical, fiscal and environmental benefits of a specialist led virtual ureteric colic clinic: a report of a prospective study. BJU international. 2019.

21. Coombs NJ, Coombs JM, Vaidya UJ, Singer J, Bulsara M, Tobias JS, et al. Environmental and social benefits of the targeted intraoperative radiotherapy for breast cancer: Data from UK TARGIT-A trial centres and two UK NHS hospitals offering TARGIT IORT. BMJ Open. 2016;6(5):e010703.

22. Cooper. Exploring the Impact and Acceptance of

Wearable Sensor Technology for Pre- and Postoperative Rehabilitation in Knee

Replacement Patients: A U.K.-Based Pilot Study. 2022.

23. Cooper DM, Bhuskute N, Hepworth C, Walsh G. The Economic Impact of a Pilot Digital Day-Case Pathway for Knee Arthroplasty in a U.K. Setting. JB & JS open access. 2023;8(1).

24. Croghan SM, Rohan P, Considine S, Salloum A, Smyth L, Ahmad I, et al. Time, cost and carbon-efficiency: a silver of COVID era virtual urology clinics? Annals of the Royal College of Surgeons of England. 2021;103(8):599-603.

25. Curtis A, Parwaiz H, Winkworth C, Sweeting L, Pallant L, Davoudi K, et al. Remote Clinics During Coronavirus Disease 2019: Lessons for a Sustainable Future. Cureus. 2021;13(3):e14114.

26. Davis NF, McGrath S, Quinlan M, Jack G, Lawrentschuk N, Bolton DM. Carbon footprint in flexible ureteroscopy: A comparative study on the environmental impact of reusable and single-use ureteroscopes. Journal of Endourology. 2018;32(3):214-7.

27. de Preux L, Rizmie D. Beyond financial efficiency to support environmental sustainability in economic evaluations. Future Healthcare Journal. 2018;5(2):103-7.

28. de Ridder EF, Friedericy HJ, van der Eijk AC, Dankelman J, Jansen FW. A New Method to Improve the Environmental Sustainability of the Operating Room: Healthcare Sustainability Mode and Effect Analysis (HSMEA). Sustainability. 2022;14(21).

29. Dorrian C, Ferguson J, Ah-See K, Barr C, Lalla K, van der Pol M, et al. Head and neck cancer assessment by flexible endoscopy and telemedicine. Journal of Telemedicine and Telecare. 2009;15(3):118-21.

30. Field RR, Calderon M-DC, Ronilo SM, Ma M, Maxwell H, Mensah P, et al. Environmental and Economic Impact of Using a Higher Efficiency Ventilator and Vaporizer During Surgery Under General Anesthesia: A Randomized Controlled Prospective Cohort. Cureus. 2023;15(5):e39534.

31. Filfilan A, Anract J, Chartier-Kastler E, Parra J, Vaessen C, de La Taille A, et al. Positive environmental impact of remote teleconsultation in urology during the COVID-19 pandemic in a highly populated area. Progres En Urologie. 2021;31(16):1133-8.

32. Forner D, Purcell C, Taylor V, Noel CW, Pan L, Rigby MH, et al. Carbon footprint reduction associated with a surgical outreach clinic. Journal of Otolaryngology-Head & Neck Surgery. 2021;50(1).

33. Frick MA, Baniel CC, Qu V, Hui C, Brown E, Chang DT, et al. Effect of Radiation Schedule on Transportation-Related Carbon Emissions: A Case Study in Rectal Cancer. Advances in Radiation Oncology. 2023;8(5):101253.

34. Fuschi A, Pastore AL, Al Salhi Y, Martoccia A, De Nunzio C, Tema G, et al. The impact of radical prostatectomy on global climate: a prospective multicentre study comparing laparoscopic versus robotic surgery. Prostate Cancer and Prostatic Diseases. 2023.

35. Hardy. Review of the haemodialysis process in a single satellite dialysis unit with the aim to reduce carbon and waste 2022 [

36. Heye T, Meyer MT, Merkle EM, Vosshenrich J. Turn It Off! A Simple Method to Save Energy and CO2 Emissions in a Hospital Setting with Focus on Radiology by Monitoring Nonproductive Energy-consuming Devices. Radiology. 2023;307(4):e230162.

37. Hogan D, Rauf H, Kinnear N, Hennessey DB. The Carbon Footprint of Single-Use Flexible Cystoscopes Compared with Reusable Cystoscopes. Journal of Endourology. 2022;36(11):1460-4.

38. Holmner A, Ebi KL, Lazuardi L, Nilsson M. Carbon Footprint of Telemedicine Solutions - Unexplored Opportunity for Reducing Carbon Emissions in the Health Sector. Plos One. 2014;9(9).

39. Jiang CY, Strohbehn GW, Dedinsky RM, Raupp SM, Pannecouk BM, Yentz SE, et al. Teleoncology for Veterans: High Patient Satisfaction Coupled With Positive Financial and Environmental Impacts. JCO Oncology Practice. 2021;17(9):E1362-E74.

40. Kemble JP, Winoker JS, Patel SH, Su ZT, Matlaga BR, Potretzke AM, et al. Environmental impact of single-use and reusable flexible cystoscopes. BJU International. 2023;131(5):617-22.

41. King J, Poo SX, El-Sayed A, Kabir M, Hiner G, Olabinan O, et al. Towards NHS Zero: greener gastroenterology and the impact of virtual clinics on carbon emissions and patient outcomes. A multisite, observational, cross-sectional study. Frontline Gastroenterology. 2022.

42. Klein H-M. A New Approach to the Improvement of Energy Efficiency in Radiology Practices. Ein neuer Ansatz zur Verbesserung der Energieeffizienz in radiologischen Versorgungseinheiten. 2023;195(5):416-25.

43. Kodumuri. Reducing the carbon footprint in carpal tunnel surgery inside the operating room with a lean and green model: a comparative study. . 2022.

44. Kodumuri P, Jesudason EP, Lees V. Reducing the carbon footprint in carpal tunnel surgery inside the operating room with a lean and green model: a comparative study. The Journal of hand surgery, European volume. 2023:17531934231176952.

45. Lambert P, Musto G, Thiessen M, Czaykowski P, Decker K. Impact of Cancer-Related Virtual Visits on Travel Distance, Travel Time, and Carbon Dioxide (CO2) Emissions during the COVID-19 Pandemic in Manitoba, Canada. Curr Oncol. 2023;30(7):5973-83.

46. Langstaff. Photobiomodulation Therapy (PBM): Using light therapy for oral mucositis, 2023 [

47. Le NNT, Hernandez LV, Vakil N, Guda N, Patnode C, Jolliet O. Environmental and health outcomes of single-use versus reusable duodenoscopes. Gastrointestinal Endoscopy. 2022;96(6):1002-8.

48. Leapman MS, Thiel CL, Gordon IO, Nolte AC, Perecman A, Loeb S, et al. Environmental Impact of Prostate Magnetic Resonance Imaging and Transrectal Ultrasound Guided Prostate Biopsy. European Urology. 2023;83(5):463-71.

49. Leiden A, Cerdas F, Noriega D, Beyerlein J, Herrmann C. Life cycle assessment of a disposable and a reusable surgery instrument set for spinal fusion surgeries. Resources, Conservation and Recycling. 2020;156:104704.

50. Lewis D, Tranter G, Axford AT. Use of videoconferencing in Wales to reduce carbon dioxide emissions, travel costs and time. J Telemed Telecare. 2009;15(3):137-8.

51. Lopez-Munoz P, Martin-Cabezuelo R, Lorenzo-Zuniga V, Vilarino-Feltrer G, Tort-Ausina I, Vidaurre A, et al. Life cycle assessment of routinely used endoscopic instruments and simple intervention to reduce our environmental impact. Gut. 2023:E329544.

52. Materacki. Scoping for change - adopting greener practice in endoscopy in Gloucestershire, Endoscopy team 2023 [

53. McAlister S, McGain F, Petersen M, Story D, Charlesworth K, Ison G, et al. The carbon footprint of hospital diagnostic imaging in Australia. The Lancet Regional Health - Western Pacific. 2022;24:100459.

54. McCarthy CJ, Gerstenmaier JF, O' Neill AC, McEvoy SH, Hegarty C, Heffernan EJ. "EcoRadiology"--pulling the plug on wasted energy in the radiology department. Academic radiology. 2014;21(12):1563-6.

55. McLachlan A, Aldridge C, Morgan M, Lund M, Gabriel R, Malez V. An NP-led pilot telehealth programme to facilitate guideline-directed medical therapy for heart failure with reduced ejection fraction during the COVID-19 pandemic. N Z Med J. 2021;134(1538):77-88.

56. Meiklejohn DA, Khan ZH, Nunez KM, Imhof L, Osmani S, Benavidez AC, et al. Environmental Impact of Adult Tonsillectomy: Life Cycle Assessment and Cost Comparison of Techniques. The Laryngoscope. 2023.

57. Miah S, Dunford C, Edison M, Eldred-Evans D, Gan C, Shah TT, et al. A prospective clinical, cost and environmental analysis of a clinician-led virtual urology clinic. Ann R Coll Surg Engl. 2019;101(1):30-4.

58. Milne. Green Nephrology: Retro-fit of heat cxchangers to haemodialysis machines - Case study and how-to guide: he Campaign for Greener Healthcare; 2010 [

59. Milne. Retro-fit of Heat Exchangers to Haemodialysis Machines - Case Study and How to Guide. : The Campaign for Greener Healthcare; 2023 [

60. Mojdehbakhsh RP, Rose S, Peterson M, Rice L, Spencer R. A quality improvement pathway to rapidly increase telemedicine services in a gynecologic oncology clinic during the COVID-19 pandemic with patient satisfaction scores and environmental impact. Gynecologic Oncology Reports. 2021;36:100708.

61. Moussa G, Andreatta W, Ch'Ng SW, Ziaei H, Jalil A, Patton N, et al. Environmental effect of air versus gas tamponade in the management of rhegmatogenous retinal detachment VR surgery: A multicentre study of 3,239 patients. PLoS ONE. 2022;17(1 January):e0263009.

62. Moussa G, Ch'ng SW, Park DY, Ziaei H, Jalil A, Patton N, et al. Environmental effect of fluorinated gases in vitreoretinal surgery: a multicenter study of 4,877 patients. American journal of ophthalmology. 2021.

63. Muschol J, Heinrich M, Heiss C, Hernandez AM, Knapp G, Repp H, et al. Economic and Environmental Impact of Digital Health App Video Consultations in Follow-up Care for Patients in Orthopedic and Trauma Surgery in Germany: Randomized Controlled Trial. J Med Internet Res. 2022;24(11):11.

64. Natale J, Pascoe J, Horn C, Coode-Bate J, Dickinson A. Teleconsultation versus traditional clinical assessment of patients undergoing circumcision: A retrospective cohort study. Journal of Clinical Urology. 2022.

65. Neves JAC, Roseira J, Queiros P, Sousa HT, Pellino G, Cunha MF. Targeted intervention to achieve waste reduction in gastrointestinal endoscopy. Gut. 2022.

66. Nielson. Pioneering Early Mobilisation in a Cardiac Intensive Care unit: a Sustainable Healthcare Initiative. . 2022.

67. Owens. COLLABORATIVE REPORT OF PAPER LITE AND CONTRAST RECYCLING PROJECTS,

ENDOSCOPY TEAM. 2023.

68. Patel KB, Gonzalez BD, Turner K, Alishahi Tabriz A, Rollison DE, Robinson E, et al. Estimated Carbon Emissions Savings With Shifts From In-Person Visits to Telemedicine for Patients With Cancer. JAMA network open. 2023;6(1):e2253788.

69. Phull M, Begum H, John JB, van Hove M, McGrath J, O'Flynn K, et al. Potential Carbon Savings with Day-case Compared to Inpatient Transurethral Resection of Bladder Tumour Surgery in England: A Retrospective Observational Study Using Administrative Data. European Urology Open Science. 2023;52:44-50.

70. Richards JD, Stoddart M, Bolland B. Virtual Arthroplasty Follow-Up: Better for the Trust, Patients, and the Planet. Cureus. 2022;14(11):e31978.

71. Rizan C, Bhutta MF. Environmental impact and life cycle financial cost of hybrid (reusable/single-use) instruments versus single-use equivalents in laparoscopic cholecystectomy. Surgical Endoscopy. 2022;36(6):4067-78.

72. Rouviere N, Chkair S, Auger F, Alovisetti C, Bernard MJ, Cuvillon P, et al. Ecoresponsible actions in operating rooms: A health ecological and economic evaluation. International Journal of Surgery. 2022;101:106637.

73. Sanchez SA, Eckelman MJ, Sherman JD. Environmental and economic comparison of reusable and disposable blood pressure cuffs in multiple clinical settings. Resources, Conservation and Recycling. 2020;155:104643.

74. Schulte A, Maga D, Thonemann N. Combining Life Cycle Assessment and Circularity Assessment to Analyze Environmental Impacts of the Medical Remanufacturing of Electrophysiology Catheters. Sustainability. 2021;13(2).

75. Sherman JD, Raibley LAt, Eckelman MJ. Life Cycle Assessment and Costing Methods for Device Procurement: Comparing Reusable and Single-Use Disposable Laryngoscopes. Anesthesia and analgesia. 2018;127(2):434-43.

76. Sillcox R, Blaustein M, Khandelwal S, Bryant MK, Zhu J, Chen JY. Telemedicine Use Decreases the Carbon Footprint of the Bariatric Surgery Preoperative Evaluation. Obesity Surgery. 2023.

77. Sillcox R, Gitonga B, Meiklejohn DA, Wright AS, Oelschlager BK, Bryant MK, et al. The environmental impact of surgical telemedicine: life cycle assessment of virtual vs. in-person preoperative evaluations for benign foregut disease. Surgical Endoscopy and Other Interventional Techniques. 2023.

78. Sorensen. Comparative Study on Environmental Impacts of Reusable and Single-Use Bronchoscopes. . 2018.

79. Stripple H, Westman R, Holm D. Development and environmental improvements of plastics for hydrophilic catheters in medical care: an environmental evaluation. Journal of Cleaner Production. 2008;16(16):1764-76.

80. Thiel CL, Eckelman M, Guido R, Huddleston M, Landis AE, Sherman J, et al. Environmental impacts of surgical procedures: Life cycle assessment of hysterectomy in the United States. Environmental Science and Technology. 2015;49(3):1779-86.

81. Thiel CL, Mehta N, Sejo CS, Qureshi L, Moyer M, Valentino V, et al. Telemedicine and the environment: life cycle environmental emissions from in-person and virtual clinic visits. Npj Digital Medicine. 2023;6(1).

82. Thiel CL, Woods NC, Bilec MM. Strategies to Reduce Greenhouse Gas Emissions from Laparoscopic Surgery. American journal of public health. 2018;108(S2):S158-S64.

83. Thota R, Gill DM, Brant JL, Yeatman TJ, Haslem DS. Telehealth Is a Sustainable Population Health Strategy to Lower Costs and Increase Quality of Health Care in Rural Utah. JCO oncology practice. 2020;16(7):e557-e62.

84. Tselapedi-Sekeitto. Telemedicine as an environmental ally - The social, financial, and environmental impact of virtual care in the otolaryngology clinic. . Am J Otolaryngol. 2023;44(2).

85. Udayaraj UP, Watson O, Ben-Shlomo Y, Langdon M, Anderson K, Power A, et al. Establishing a tele-clinic service for kidney transplant recipients through a patient-codesigned quality improvement project. BMJ open quality. 2019;8(2):e000427.

86. Vaidya JS, Vaidya UJ, Baum M, Bulsara MK, Joseph D, Tobias JS. Global adoption of single-shot targeted intraoperative radiotherapy (TARGIT-IORT) for breast cancer-better for patients, better for healthcare systems. Frontiers in Oncology. 2022;12:786515.

87. Vo LV, Mastrorilli V, Muto AJ, Emerson GG. Reuse of shipping materials in the intravitreal bevacizumab supply chain: feasibility, cost, and environmental impact. International Journal of Retina and Vitreous. 2023;9(1):34.

88. Winklmair N, Kieselbach G, Bopp J, Amon M, Findl O. Potential environmental effect of reducing the variation of disposable materials used for cataract surgery. Journal of Cataract and Refractive Surgery. 2023;49(6):628-34.

89. Wombwell A, Holmes A, Grills R. Are single-use flexible cystoscopes environmentally sustainable? A lifecycle analysis. Journal of Clinical Urology. 2023.

90. Woods DL, McAndrew T, Nevadunsky N, Hou JY, Goldberg G, Yi-Shin Kuo D, et al. Carbon footprint of robotically-assisted laparoscopy, laparoscopy and laparotomy: A comparison. International Journal of Medical Robotics and Computer Assisted Surgery. 2015;11(4):406-12.

91. Woolen SA, Becker AE, Martin AJ, Knoerl R, Lam V, Folsom J, et al. Ecodesign and Operational Strategies to Reduce the Carbon Footprint of MRI for Energy Cost Savings. Radiology. 2023;307(4):e230441.

92. Yong KK, He Y, Cheung HCA, Sriskandarajah R, Jenkins W, Goldin R, et al. Rationalising the use of specimen pots following colorectal polypectomy: a small step towards greener endoscopy. Frontline Gastroenterology. 2022.

93. Zander A, Niggebrugge A, Pencheon D, Lyratzopoulos G. Changes in travel-related carbon emissions associated with modernization of services for patients with acute myocardial infarction: A case study. Journal of Public Health. 2011;33(2):272-9.
