# Supplementary material for: Effectiveness of Interventions to Reduce Carbon‐Emissions Within Secondary Healthcare: Systematic Review and Evidence and Gap Map
Source: Campbell Syst Rev. 2025 Dec 23;21(4):e70077. doi: 10.1002/cl2.70077 (PMC12723626; doi:10.1002/cl2.70077)
Supplement: Supplementary file 5 — Appendix. [file CL2-21-e70077-s006.docx]

# Appendices

## 1 Search strategies

### Bibliographic database searches

Database: MEDLINE
Host: Ovid
Issue: 1946 to July 11, 2023
Date Searched: 12^th^ July 2023
Searcher: SB
Hits: 3052
Strategy:

1. ((carbon or CO2 or CO2eq) adj3 (emission* or footprint or impact* or output or green* or sustainab*)).tw.
2. (greenhouse adj1 (effect or gas*)).tw.
3. (("life cycle" or lifecycle) adj1 (analys?s or assessment*)).tw.
4. ((climate or environment*) adj1 (footprint or impact* or sustainab*)).tw.
5. Carbon Footprint/
6. Greenhouse Gases/
7. Air Pollutants, Occupational/
8. Global Warming/
9. environmental indicators/
10. or/1-9
11. (gastro* or gastric*).tw.
12. (gi adj1 (disease* or disorder*)).tw.
13. ((bowel or gi or intesti* or liver or stomach) adj1 (assessment* or biops* or bleeding or cirrhosis or disease* or disorder* or failure or illness* or cancer* or carcinoma* or tumour* or tumor* or neoplasm*)).tw.
14. ("abdominal adhesion*" or appendicitis or "barrett's esophagus" or "celiac disease" or "colon polyps" or "crohn's disease" or "esophageal varices" or "pancreatic insufficiency" or gallstones or gastritis or h?emorrhoid* or hernia* or colitis or pancreatitis or "peptic ulcer*" or "stomach ulcer*" or proctitis or pylori).tw.
15. Gastroenterology/
16. exp Gastrointestinal Diseases/
17. (appendectomy or colonoscop* or duodenoscop* or endoscop* or gastroscop* or colectomy).tw.
18. exp Endoscopy, Gastrointestinal/
19. manometry.tw.
20. exp Manometry/
21. (barium adj1 (enema or swallow)).tw.
22. (cholangiography or cholangiopancreatography).tw.
23. exp Cholangiography/
24. (esophagogram or electrogastrogram).tw.
25. exp Digestive System Surgical Procedures/
26. exp digestive system neoplasms/
27. or/11-26
28. 10 and 27
29. (orthopedic* or orthopaedic* or musculoskeletal).tw.
30. Orthopedics/
31. exp Musculoskeletal System/
32. ((ankle or bone or cervical or elbow or femoral or finger or hand or hip or joint or knee or neck or shoulder or spine) adj2 (break or broken or fracture*)).tw.
33. (bone adj1 (cancer* or carcinoma* or tumour* or tumor* or neoplasm*)).tw.
34. exp Fractures, Bone/
35. ("carpal tunnel" adj2 (syndrome or release)).tw.
36. Carpal Tunnel Syndrome/
37. osteoporosis.tw.
38. exp osteoporosis/
39. ((ankle or bone or cervical or elbow or femoral or finger or hand or hip or joint or knee or neck or shoulder or spine) adj2 (arthroplast* or arthroscop* or implant* or reconstruction or repair or replacement)).tw.
40. (cementoplasty or diskectomy or "fracture fixation" or "intervertebral disc chemolysis" or laminectomy or laminoplasty or "orthopedic manipulation posterior cruciate ligament reconstruction" or "ulnar collateral ligament reconstruction" or viscosupplementation).tw.
41. exp Orthopedic Procedures/
42. (trauma adj1 (care or "life support" or medic*)).tw.
43. Trauma Centers/
44. exp bone neoplasms/
45. or/29-44
46. 10 and 45
47. (cardiolog* or cardiovascular).tw.
48. exp Cardiology/
49. Cardiology Service, Hospital/
50. ((cardiac or heart) adj3 (arrest or attack* or disease* or disorder* or defect* or dysfunction or failure or sarcoma or transplant*)).tw.
51. "myocardial infarction".tw.
52. ((aortic or artery or arterial) adj1 disease).tw.
53. "intermittent claudication".tw.
54. aneurysm.tw.
55. hypertension.tw.
56. exp Hypertension/
57. exp Heart Diseases/
58. (angioplasty or "arterial switch operation" or "artificial heart valve" or "heart valve replacement" or atherectomy or "cardiac valve annuloplasty" or cardiomyoplasty or "heart bypass" or "heart massage" or "heart valve prosthesis implant*" or "maze procedure" or revascularization or vascularization or "norwood procedures" or "pericardial window techniques" or pericardiectomy or pericardiocentesis).tw.
59. exp Cardiac Surgical Procedures/
60. exp Angioplasty/
61. stent*.tw.
62. exp Stents/
63. exp cardiac imaging techniques/
64. exp heart neoplasms/
65. or/47-64
66. 10 and 65
67. ophthalm*.tw.
68. Ophthalmology/
69. (cataract* or glaucoma).tw.
70. "macular degeneration".tw.
71. "diabetic retinopathy".tw.
72. exp Eye Diseases/
73. ((eye* or retina*) adj3 (care or detachment or disease or disorder* or cancer* or carcinoma* or tumour* or tumor* or neoplasm*)).tw.
74. "intravitreal injection*".tw.
75. Intravitreal Injections/
76. (blepharoplasty or dacryocystorhinostomy or "eye enucleation" or "eye evisceration" or "filtering surgery" or sclerostomy or trabeculectomy or iridectomy or "light coagulation" or "laser coagulation" or "orbit evisceration" or "corneal transplant*" or "radial keratotomy" or "lens implant*" or "posterior capsulotomy" or scleroplasty or "scleral buckling" or vitrectomy).tw.
77. exp Ophthalmologic Surgical Procedures/
78. exp eye neoplasms/
79. or/67-78
80. 10 and 79
81. ((lung or pulmonary or respiratory) adj3 (care or disease* or disorder* or cancer* or carcinoma* or tumour* or tumor* or neoplasm*)).tw.
82. Pulmonary Medicine/
83. ("chronic obstructive pulmonary disease" or COPD).tw.
84. lung diseases, obstructive/
85. exp pulmonary disease, chronic obstructive/
86. ((emergency or hospital or medical or therap*) adj3 oxygen).tw.
87. (bronchoscop* or "endobronchial ultrasound" or bullectomy or "chest drain" or "lung transplant*").tw.
88. exp Respiratory Therapy/
89. bronchoscopy/
90. exp Diagnostic Techniques, Respiratory System/
91. (asthma or inhaler*).tw.
92. Asthma/
93. respiratory tract neoplasms/
94. or/81-93
95. 10 and 94
96. nephrolog*.jn,tw.
97. Nephrology/
98. (renal or kidney*).jn,tw.
99. ((renal or kidney) adj3 (acute or chronic or disease* or "end stage" or failure or injury or injuries or transplant* or cancer* or carcinoma* or tumour* or tumor* or neoplasm*)).tw.
100. nephropathy.tw.
101. exp Kidney Failure, Chronic/
102. exp Kidney Diseases/
103. exp Renal Replacement Therapy/
104. ((kidney or renal) adj3 (therap* or replacement or transplant*)).tw.
105. (dialysis or h?emodialysis or h?emofiltration or h?emoperfusion or lithotripsy).tw.
106. exp Carcinoma, Renal Cell/
107. or/96-106
108. 10 and 107
109. ("critical care" or "intensive care" or intensivist* or icu).jn,tw.
110. (serious adj1 (accident* or injur* or infect*)).tw.
111. exp Critical Care/
112. or/109-111
113. 10 and 112
114. exp Gynecologic Surgical Procedures/
115. (colposcopy or colpotomy or "culdoscopy dilatation and curettage" or "endometrial ablation" or hysterectomy or hysteroscopy or ovariectomy or salpingectomy or salpingostomy or "tubal sterilization" or "uterine artery embolization" or "uterine myomectomy" or vulvectomy).tw.
116. 114 or 115
117. 10 and 116
118. exp Urologic Surgical Procedures/
119. (cystectomy or cystoscopy or cystotomy or "kidney Transplant*" or nephrectomy or nephroureterectomy or nephrolithotomy or nephrotomy or nephrostomy or "transurethral resection" or ureteroscopy or "urinary diversion" or ureterostomy or "male circumcision" or orchiectomy or orchiopexy or "penile Implantation" or prostatectomy or vasectomy or vasovasostomy).tw.
120. 118 or 119
121. 10 and 120
122. exp Otorhinolaryngologic Surgical Procedures/
123. (adenoidectomy or laryngectomy or laryngoplasty or laryngoscopy or rhinoplasty or "neck dissection" or "auditory brain stem implant*" or "cochlear implant*" or "endolymphatic shunt" or "labyrinth fenestration" or mastoidectomy or "middle ear ventilation" or myringoplasty or "ossicular replacement" or "transtympanic micropressure treatment" or tympanoplasty or pharyngectomy or pharyngostomy or tonsillectomy or tracheostomy or tracheotomy).tw.
124. 122 or 123
125. 10 and 124
126. exp chemoprevention/
127. exp chemoradiotherapy/
128. exp chemotherapy, adjuvant/
129. exp consolidation chemotherapy/
130. (chemotherapy or chemoprevention or chemoradiotherapy or radiotherapy).tw.
131. oncolog*.tw.
132. exp Medical Oncology/
133. antineoplastic*.tw.
134. exp Antineoplastic Agents/
135. or/126-134
136. 10 and 135
137. obstetric*.tw.
138. exp obstetrics/
139. ((oxytocin or labo?r) adj3 induc*).tw.
140. ("electro f?etal monitoring" or "continuous EFM").tw.
141. (amniotomy or enema or epidural or episiotomy).tw.
142. ("artificial rupture" adj2 membranes).tw.
143. ("cervical cerclage" or colposcop* or colpotomy or culdoscop* or fetoscop* or hysteroscop* or hysterotomy or "umbilical cord clamp*").tw.
144. abortion*.tw.
145. (terminat* adj2 pregnancy).tw.
146. exp Pregnancy Complications/
147. exp Obstetric Surgical Procedures/
148. or/137-147
149. 10 and 148
150. (radiolog* or radiotherap*).tw.
151. exp Radiology/
152. (angiography or "CT scan" or echocardiogram or "electrocardiogram" or "magnetic resonance imag* MRI" or "PET scan" or tomography or ultrasound or "x ray").tw.
153. (CT adj2 (micro or "high resolution" or "volumetric quantitative")).tw.
154. ((medical or fluoroscopic*) adj2 imag*).tw.
155. exp Diagnostic Imaging/
156. or/150-155
157. (health or hospital* or medical or medicine or pharmaceutical).tw.
158. exp Hospitals/
159. Hospital Medicine/
160. or/157-159
161. 10 and 156 and 160
162. 28 or 46 or 66 or 80 or 95 or 108 or 113 or 117 or 121 or 125 or 136 or 149 or 161
163. limit 162 to (english language and yr="2008 -Current")

Database: Environment Complete
Host: EBSCO
Issue: n/a
Date Searched: 12^th^ July 2023
Searcher: SB
Hits: 2753
Strategy:

1. TI ( healthcare or "health care" or "health service*" or "health system" or hospital* or medical or medicine or pharmaceutical ) OR AB ( healthcare or "health care" or "health service*" or "health system" or hospital* or medical or medicine or pharmaceutical )
2. DE "MEDICINE"
3. S1 OR S2
4. TI (climate or environment* N2 (footprint or impact* or sustainab*) ) OR AB (climate or environment* N2 (footprint or impact* or sustainab*) )
5. TI ( ("life cycle" or lifecycle) N0 (analys?s or assessment*) ) OR AB ( ("life cycle" or lifecycle) N0 (analys?s or assessment*)
6. TI ( greenhouse N0 (effect or gas*) ) OR AB ( greenhouse N0 (effect or gas*) )
7. TI ( (carbon or CO2) N2 (emission* or footprint or impact* or output or sustainab*) ) OR AB ( (carbon or CO2) N2 (emission* or footprint or impact* or output or sustainab*) )
8. DE "HOSPITAL waste disposal"
9. DE "CARBON emissions"
10. S4 OR S5 OR S6 OR S7 OR S8 OR S9
11. S3 AND S10

### Web searches

HLCA Categories searched 26^th^ September 2023

| **Specialty** | **HLCA category** | **Results (n)** | **Studies (n)** | **Not in BD results (n)** |
| --- | --- | --- | --- | --- |
| Cardiology | Cardiology | 6 | 6 | 1 (thesis) |
| Gastroenterology | Gastroenterology | 6 | 6 | 1 (letter) |
| Ophthalmology | Ophthalmology | 11 | 11 | 0 |
| Orthopaedics and trauma | Orthopaedics | 5 | 5 | 0 |
| Renal | Renal medicine | 6 | 6 | 0 |
| Respiratory | Respiratory medicine | 10 | 10 | 2 |
| Obstetrics, | Obstetrics and gynaecology | 8 | 8 | 0 |
| Radiology | Radiology | 4 | 4 | 1 (thesis) |
| Oncology | Oncology | 1 | 1 | 0 |
| *High volume low complexity surgery, including:* |  |  |  |  |
| Ear, nose and throat | ENT | 1 | 1 | 0 |
| Gynaecology | See above | See above | See above | See above |
| Urology | Urology | 8 | 8 | 0 |

Google Search. Searched 19^th^ September 2023

“carbon emissions” (health OR hospital)

About 45,800,000 results (0.35 seconds)

19^th^ September 2023

400 with repeated results showing

188 without repeated results

### Websites

- Centre for Sustainable Healthcare <https://sustainablehealthcare.org.uk/>
- Current Awareness Service for Health <https://cash.libraryservices.nhs.uk/>
- European Centre for Environmental and Human Health <https://www.ecehh.org/>
- Health Care Without Harm <https://noharm-europe.org/>
- IHSCM – Greener care special interest group <https://ihm.org.uk/special-interest-groups/greener-care/>
- Green Health Wales <https://greenhealthwales.co.uk/>
- Greener NHS <https://www.england.nhs.uk/greenernhs/>
- Sustainable Healthcare Networks Hub <https://networks.sustainablehealthcare.org.uk/T>

## 2 Data extraction items for included studies

| **Study details** | **Sample characteristics** | **Intervention/Control Characteristics** | **Carbon emission calculation methods** | **PROGRESS-PLUS (detail relevant to below categories extracted)** | **Other outcomes measured** | **Carbon emission findings** |
| --- | --- | --- | --- | --- | --- | --- |
| First author, date of study | Setting | Intervention/first comparator category and name | LCA informed – Y/N | Place of residence | Method of data collection | Summary of main findings (cut and paste) |
| Title of article | Specialty | Description | If LCA – LCA/Inventory analysis? | Race/ethnicity/culture /language | Outcome measure name |  |
| Publication type | Clinician characteristics | Aim | Standards/reference data used to inform CE calculations | Occupation | Outcome measure category |  |
| Country | Total No. patients (intervention/control) | Details of treatment pathway | Type of CE (Scope 1, 2, 3) (non-LCA studies) | Gender/Sex | Rater |  |
| Income | Dropouts | How accessed | Method of calculating carbon emission data | Religion | Blinded (Y/N) |  |
| Aim of study | Patient characteristics (Procedure, mean age, | Materials required to deliver | Functional unit (as stated) | SES | Analysis method |  |
| Summary of findings (from abstract) | Recruitment method | Procedures | System boundaries | Social capital | Narrative summary of findings |  |
| Study design | Allocation procedure | Who delivered, mode of delivery, frequency/duration, tailored to patient needs, modifications | Stages of system | Personal characteristics associated with discrimination |  |  |
| Funding statement | Inclusion criteria | Recipients | Statement of representativeness of data | Features of relationships |  |  |
| Conflict of interest |  | Adherence/fidelity assessed – if Y, findings extracted |  | Time-dependent relationships |  |  |

Blue shaded cell=data collected from non-LCA studies. CE=Carbon Emissions, LCA=Life Cycle Assessment, N=No, No=Number, SES=Socioeconomic status, Y=Yes

## 3 Critical appraisal criteria applied to LCA studies

| **Criteria** |
| --- |
| **Phase 1: Goal & scope (12 points)** |
| Study goal is clearly stated, including the study's rationale (1), intended application, and/or intended audience (1) |
| Lifecycle assessment method is clearly stated (1) |
| Functional unit is clearly defined and measurable (1), justified (1), and consistent with the study's intended application (1) |
| The system to be studied is adequately described with clearly stated system boundaries (1), lifecycle stages (1), and appropriate justification of any omitted stages (1) |
| The system covers production (1), use/reuse (1) and disposal (1) of materials and energy (half mark if only for energy and vice versa) |
| **Phase 2: Inventory analysis (7 points)** |
| The data collection process is clearly explained, including the source(s) of foreground material weights and energy values (1); the source(s) of reference data (e.g. inventory database (1); and what data are included (e.g. production and disposal of unit processes (1) |
| Representativeness of the data is discussed (1), differences in electricity generating mix are accounted for (1), and the potential significance of exclusions or assumptions is addressed (1) |
| Allocation procedures, where necessary, are described and appropriately justified (1) |
| **Phase 3: Impact assessment (6 points)** |
| Impact categories (1), characterisation method (1), and software used (1) are documented transparently |
| Results are clearly reported in the context of the functional unit (1) (0.5 if graphically, 0 if only normalized results reported) |
| A contribution analysis is performed and clearly reported (1), and hotspots are identified (1) |
| **Phase 4: Interpretation (10 points)** |
| Conclusions are consistent with the goal and scope (1) and supported by the impact assessment results (1) |
| Results are contextualized through the use of sensitivity analysis (1) and uncertainty analysis (1) |
| Limitations are adequately discussed (1), and the potential impact of omissions or assumptions on the study's outcomes are described (1) |
| The assessment has been critically appraised (i.e. peer review if journal article or independent, external critical review if report/thesis; 1) |
| Source(s) of funding (1) and any potential conflict(s) of interest are disclosed (1), and are unlikely to be a source of bias (1) |

* Numbers in brackets show points assigned for each item.

## 4 Patient care pathway

**
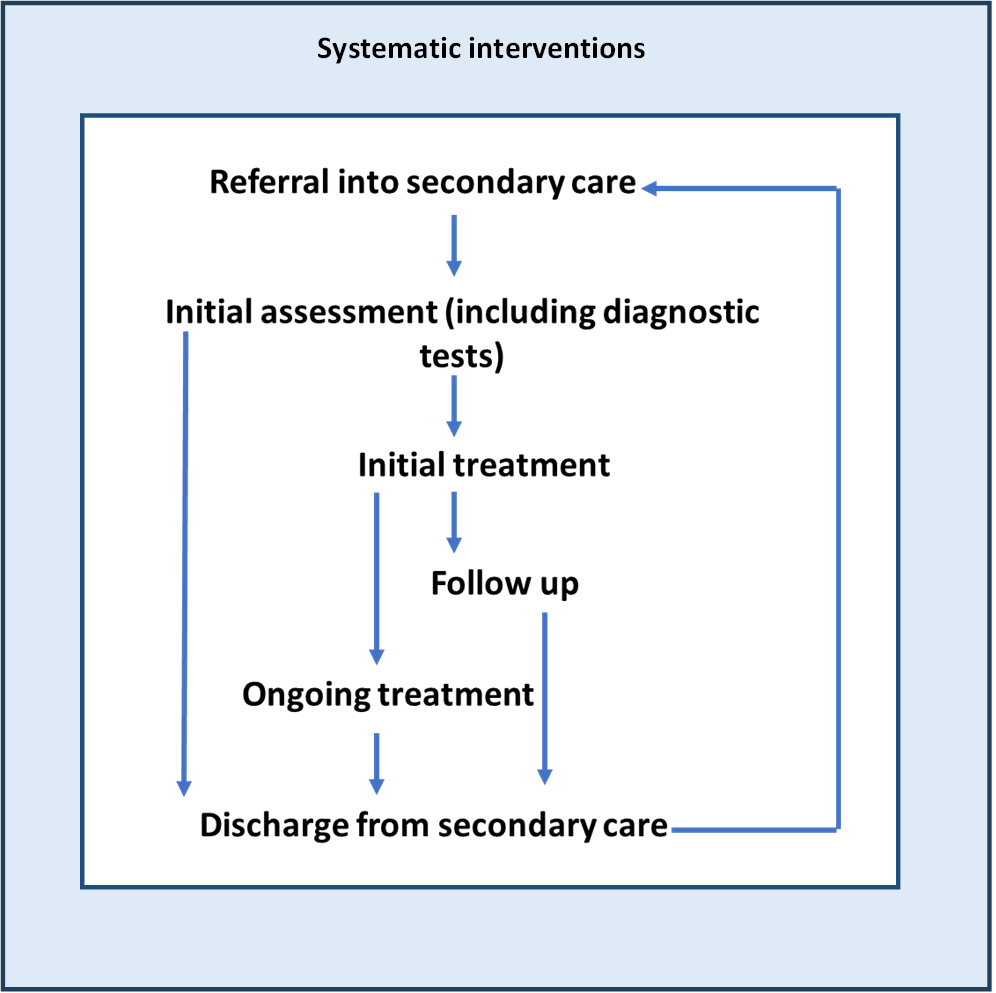
**

## 5 Description of interventions evaluated by LCA studies

| **Specialty** | **Study: Setting** | **Intervention/ Comparator** | **Intervention aim** | **Intervention/Comparator description** |
| --- | --- | --- | --- | --- |
| **Product level: Reuseable equipment** | | | | |
| **Urology** | Baboudijian 2023: NR(Baboudjian et al., 2022) | C1 Disposable cystoscope, C2 aS4C reusable cystoscope | NR | ***C1*** Disposable cystoscope: Ambu (Ballerup, Denmark), ***C2*** aS4C reusable cystoscope (aScope) |
|  | Davis 2018: Austin Hospital, Melbourne(Davis et al., 2018) | C1 Single use flexible ureteroscope, C2 Reusable flexible ureteroscope | NR | ***C1*** Single use flexible ureteroscope (LithoVue, Boston Scientific), ***C2*** Reusable flexible ureteroscope (Olympus Flexible Video scope or URV-F) |
|  | Hogan 2022: NR(Hogan et al., 2022) | C1 Single use FLEXIBLE cystoscope, C2 Reuseable flexible Cystoscope | For cystoscopy | ***C1*** Ambu aScope 3 Cysto. Packaged into punch, inner box, and outer box and sterilized in bulk using ethylene oxide according to EN ISO 11135 and EN 551-1. ***C2*** Olympus SD Flexible Cysto-Nephro videoscope (CYF-VA2). Sterilized between each use, starting with preclean immediately after cystoscopy, then sterilisation in an EndoThermo Disenfectors endoscopic reprocessing machine. They are then repacked in a vacuum sealed plastic container to allow storage for 72hrs. Average lifespan: 7 years |
|  | Kemble 2023: Urology(Kemble et al., 2023) | C1 Single-use flexible cystoscope, C2 Reuseable cystoscopes | Lower initial acquisition costs, no reprocessing, and greater portability | A SU flexible cystoscope was recently introduced with the proposed advantages of lower initial acquisition costs, no reprocessing, and greater portability to enable use in diverse and resource-limited clinical settings. While SU cystoscopes may have reduced initial costs compared to the purchase of a fleet of reusable devices, there are conflicting reports as to whether exclusive use of SU cystoscopes is cost-saving in the long term. Purported economic benefits aside, other non-economic aspects of SU cystoscopes relevant to urology practices are not well characterized |
|  | Wombwell 2023: Urology department(Wombwell et al., 2023) | C1 Single use flexible cystoscope: Ambu® aScope™ 4 Cysto System | Reduce environmental impact | Flexible cystoscopy is a commonly used procedure in urological practice to endoscopically assess the urethra and bladder for a multitude of conditions. Single-use cystoscopes also overcome issues of the infrastructure required for re-processing and sterilising scopes as they are sterile and portable |
| **Gastroenterology** | Boberg 2022: Skåne University Hospital, Lund(Boberg et al., 2022) | C1 Mix of reusable and single use-trocars, C2 single use trocars] | NR | Trocars for laparoscopic cholecystectomies ***C1***-one system was a mix of reusable and single-use trocars (Helsingborg Hospital. The mixed system used a single-use trocar 5–12 mm, a reusable trocar 10 mm, and a reusable trocar 5 mm. ***C2*** - one system consisted only of single-use trocars |
|  | Le 2022:NR(Le et al., 2022) | Duodenoscope C1 Single use, C2 Reusable with disposable endcaps, C3 conventional reusable | Compare the environmental and human health burden of SDs and RDs | Duodenoscopes ***C1 -*** Boston Scientific Exalt Model D, ***C2*** - An RD with disposable endcaps (TJF-Q190V; Olympus). ***C3 -*** A conventional RD (Olympus TJF-Q180V). |
|  | Rizan 2022: Operating room(Rizan & Bhutta, 2022a) | Laparoscopic cholecystectomy [C1 hybrid instruments, C2 disposable instruments] | Reduce environmental impact | We included in our analysis three types of instrument routinely used in laparoscopic cholecystectomy: laparoscopic clip appliers, laparoscopic scissors, and ports (small diameter 5 mm ports, and large diameter 10–11 mm ports). These instruments have both disposable and hybrid versions available on the market |
|  | Sherman 2019: Yale New Haven Hospital(Sherman et al., 2018) | Laryngoscopy [C1 reuseable equipment, C2 disposable equipment] | Reduce environmental impact and costs | NR |
| **Orthopaedics and Trauma** | Leiden 2020; Germany(Leiden et al., 2020) | C1 Disposable surgery instrument set, C2 Reuseable instrument set | Compares environmental impact of a reusable and a disposable spinal fusion instruments set | ***C1***, the Neo Pedicle Screw System from Neo Medical SA is used. It consists of one package with few instruments, one package with two rods and two packages with each two screws, nuts, screw extenders, and screw drivers. All parts of this single-use set are applied for a one level lumbar fusion surgery. The total weight is 2.0 kg per set. After manufacturing and packaging, the set is 60Co gamma-sterilized, transported to the central distribution point Frankfurt and delivered to the hospitals. Here the whole set is used once for a one level lumbar fusion surgery. Screws and rods are implanted, packaging and instruments are discarded and incinerated as solid waste. The disposable system is a new development and served in modular packages, which clearly focusses on reducing the number of required instruments for the surgery and therefore allows using less instruments. ***C2***, the Viper 2 surgical instruments and implants set from DePuy Synthes: VIPER®, 2019 used. It encompasses six boxes including eleven trays with several instruments, screws and rods (left side of Fig. 2). Depending on the requirements of the lumbar surgery, only a part of the set is applied. The total weight is 45.5 kg per set. It is used for five years and discarded through a solid waste incineration process at the end of life. It is assumed that the conventional set is used for 60 lumbar fusion surgeries per year. Hence, 300 surgeries can be realized throughout the lifetime of one reusable set |
| **Cardiology** | Schulte 2021: NR(Schulte et al., 2021) | C1 Remanufactured electrophysiology catheter C2 Virgin electrophysiology catheter | Reduce environmental impact | Cardiac reuseable equipment: catheter. The electrophysiological diagnostic catheter is mainly used in cardiac ablations procedures, a procedure to alleviate or eliminate specific cardiac arrhythmia forms |
| **Respiratory** | Sorensen 2018: NR(Sørensen & Grüttner, 2018) | C1 Reuseable bronchoscope, C2 | Reduce environmental impact | After cleaning, ***C1*** must be brought from a washer to a dryer/storage cabinet [16] in a clean environment with the operator wearing one set of protective equipment such as an apron, protective shoes, gloves, etc. (see table 1). After using the RBs many times (number of times unknown) they are discarded ***C2*** Single-use bronchoscopes are assumed to be used similarly to the RBs, then discarded afterwards |
| **Product level: Equipment type** | | | | |
| **Urology** | Stripple 2008: NR(Stripple et al., 2008) | C1 TPU catheter, C2 polyolefin-based elastomer catheter, C3 PVC catheter | Reduce environmental impact | ***C1*** -TPU is chemically less homogenous and can be considered more as a group of plastics, ***C2 -*** A new polymer material was developed, analysed and implemented in the urinary catheter production process. This new plastic material is principally based on different bulk polyolefins and styrene block copolymer ***C3*** - A urinary catheter is used as a standardized treatment method for intermittent emptying of the bladder, e.g. for patients suffering from urine retention. The product in this study is a single-use hydrophilic catheter used in hospital medical care and for home treatment of patients. The main function of the product, besides the medical treatment, is to offer patients a comfortable therapy, efficient treatment and a safe product. The urinary catheter consists of a catheter tube and a connector that can be connected to a urine collection bag. The tube and the connector are welded together. The physical geometry of the product and the surface structure are of great importance for the product’s functionality. The catheters are produced with different diameters (charrie`re 06-24) and different lengths (15, 20, 30 and 40 cm) to fit varying patient requirements. The charrie`re number is three times the outer diameter of the catheter tube, measured in millimetres. The two most common catheter tips are Nelaton and Tiemann....The PVC plastic used is of a plasticized type and the PVC polymer is produced with suspension polymerization. Different plasticizers can be used, but DEHP is the most common and best evaluated plasticizer for PVC and thus assumed in this study |
| **Accessing care** | | | | |
| **Hand and plastic surgery** | Holmner 2014: Patient’s home/ primary health centre(Holmner et al., 2014) | C1 Telehealth, C2 Physical visit | Reduce carbon emissions | ***C1 -*** Appointments included follow-ups, interventions, consultations, and assessments of various conditions, such as amputations of one or more fingers, osteoarthritis, flexor tendon injuries, radius fractures, finger fractures, and ligament injuries. Hand/Plastic surgery: 81 appointments conducted in the patient’s home using a PC or tablet computer, 157 at the closest primary health centre using standard videoconferencing equipment. Speech unit: patient home or closest primary health centre. ***C2*** - Telemedicine appointments were compared with care-as usual scenarios that require the patient travel to the hospital for a face-to-face visit |
| **Gastroenterology** | Sillcox 2023b: NR(Sillcox et al., 2023b) | C1 Telehealth, C2 F2F | NR | NR |
| **Multiple** | Thiel 2023: NR(Thiel et al., 2023) | C1 Telehealth, C2 F2F | Reduce carbon emissions/ increase accessibility | ***C1*** - For the virtual visit, the patient connects with a single clinician either by video conferencing or by telephone without video. ***C2*** - For in-person visits, patients must travel to the clinic, where they wait in a waiting area and are then escorted to a private exam room. Prior to their doctor’s visit, a nurse will often have the patient complete a digital questionnaire and (additionally, in office), will collect some data on the patient’s health, including blood pressure readings, height, and weight, depending on the specialty |
| **Care delivery** | | | | |
| **Renal** | Connor 2011a: Patient's home/Clinic(Connor et al., 2011a) | Treatment modality - Dialysis regimen | To deliver maintenance hemodialysis (HD), | Modality/Machine type/frequency of treatments/duration of treatments (h): ***C1*** - ICHD Standard 3 d a wk 4,  ***C2*** - HHD Standard 4 d a wk 4.5 ***C3*** - HHD Standard 6 d a wk 2, ***C3*** - HHD Standard 5 d a wk 4, ***C4*** - HHD Standard 6 nights a wk 7, ***C5*** - HHD Standard 3 nights a wk 7, ***C6*** - HHD NxStage 5.5 d a wk 3, ***C7*** - HHD NxStage 6 nights a wk 7 |
| **Urology** | Fuschi 2023: Urology clinic(Fuschi et al., 2023) | Surgical procedure [C1 Standard v C2 robotic assisted laparoscopy] | To estimate the CO2 consumption/production and evaluate all the instruments used during a standard laparoscopic or robot-assisted radical prostatectomy with or without lymphadenectomy for prostate tumors | A standardized surgical technique was used for both the robot-assisted and laparoscopic approaches, and the procedures were performed by the same team of expert surgeons. The patient was placed in a supine position with abdominoperineal disinfection and sterile placement of an 18-ch Foley catheter Rectal probe was inserted for the hydropneumatic rectal test at the end of procedure. We then proceeded with a supraumbilical incision and the introduction of a Verres needle for the induction of pneumoperitoneum to 12 mmHg with a standard CO2 insufflator with no AirSeal. The patient was then placed in the Trendelemburg position (25°). Two single use trocars (12 mm) and two trocars (5 mm) were used in the laparoscopic approach; four multi-use robotic trocars of 8 mm, one of 12 mm, and one of 5 mm were used in the robotic approach. A disposable single-use aspirator and multiuse forceps were used by the surgeon at the operating table during the robotic procedure. During laparoscopic procedures, a LigaSure vessel sealing system by Medtronic and a second multiuse forceps were also used. A hemostatic section of the lateral prostatic peduncles was made using medium or large hem-o-locks. Closure of the dorsal venous plexus of Santorini was made using a barbed V-Loc 3.0 suture. An endobag was used to remove the surgical specimens. Vesical-urethral anastomosis with the modified Van Velthoven technique was performed with Strata fix 3.0, and a second definitive 18-ch Foley catheter was placed in the bladder at the end of the anastomosis |
|  | Leapman 2023: tertiary care center located in the Northeastern USA(Leapman et al., 2023) | Treatment modality - Diagnosis pathway [C1 bpMRI with targeted and systemic biopsies, C2 mpMRI with targeted biopsy cores only, C3 Systematic biopsy without MRI, C4 mpMRI with systematic biopsy, C5 mpMRI with targeted & systematic biopsies (baseline)] | Reduce low-value clinical care. Reduce environmental pollution | We estimated the environmental impacts associated with reducing the overall number and varying the approach of a prostate biopsy by using MRI as a triage strategy or by omitting MRI. ***C5*** - The prostate biopsy pathway was divided into three process steps, as shown in Figure 1: (1) prebiopsy prostate MRI, (2) a TRUS biopsy in an outpatient clinical setting, and (3) pathologic processing of biopsy specimens in a clinical laboratory. For ***C1*** , we assumed shorter durations of active and standby time as well as the omission of MRI contrast and associated materials. For ***C2***, we explored biopsy sampling strategies including combined systematic and MRI-ultrasound fusion biopsies: targeted biopsy cores only. For ***C3***, we explored biopsy sampling strategies including combined systematic and MRI-ultrasound fusion biopsies, MRI-ultrasound fusion alone, and systematic biopsy alone, systematic biopsy without MRI. For ***C4***, we explored biopsy sampling strategies including combined systematic and MRI-ultrasound fusion biopsies: systematic biopsy with MRI |
| **Ear, Nose and Throat** | Meiklejohn 2023: Operating room - University of New Mexico Hospital(Meiklejohn et al., 2023) | Surgical procedure - tonsillectomy, without adenoidectomy or other procedures, | To quantify cost & environmental impact of techniques for Otolaryngology surgery, and identify areas to maximally reduce this impact | ***C1*** monopolar electrocautery, ***C2*** coblation, ***C3*** cold excision without cautery |
| **Gynaecology** | Thiel 2015: Magee Women’s Hospital (Magee) of the University of Pittsburgh Medical Center (UPMC)(Thiel et al., 2015) | Surgical procedure [C1 Abdominal, C2 Vaginal, C3 Laparoscopic, C4 Robotic | Complete hysterectomy | Method to perform hysterectomy |
| **Multiple** | | | | |
| **Radiology/ Radiotherapy** | Chuter 2023: Christie Centre; Mount Vernon Cancer Centre, Guys and St Thomas, South West Wales Cancer Center(Chuter et al., 2023) | Setting, Care delivery radiotherapy [C1-SABR (Stereotactic ablative radiotherapy) protocol, C2 - Before intervention] | Limit footfall and reduce infection risk | ***C2*** - For all centres, the prostate dataset consisted of 10 patients treated with 60 Gy in 20 fractions ***C1*** - two patients treated with SABR technique (36.25 Gy in 5 fractions): this reflected the implementation of a SABR protocol used to treat approximately 5% of these patients to limit foot-fall and therefore infection risk during COVID. The COVID breast dataset consisted of 10 breast patients receiving ultra-hypofractionated RT (26 Gy in 5 fractions) |
| **Multiple^a^** | Rouviere 2022: 24  OR (among which 4 ambulatory rooms), 3 preoperative rooms, 3 post anaesthesia care units, and SPD(Rouviere et al., 2022) | Waste management, anesthesia, surgical equipment, purchasing | Reduce environmental and economic impact | The sustainable actions concerning SMD were implemented in the 24 OR. Waste reduction actions (Specialty: Neurosurgery, digestive, gynecological): custom brain surgery pack, custom coelioscopy pack. Change of anaesthesia face masks to version without plastic hook (Specialty: Anesthesia), Redon drain without premounted needle for robotic urological surgery (Speciality: Urology), change from single use to reusable laryngoscope blades (Speciality: Anesthesia), implementation of a moveable irrigation fluid recovery system for wastewater (Specialty: urological/orthopedic surgery), single pack surgical kits (Specialty: Urology, some general surgeries). Waste sorting actions: recycling aluminum blisters of surgical sutures (All surgical specialties), optimising selective waste sorting in OR, metal waste recycling at SPD, rationalisation of use of triclosan coated surgical sutures (Specialty: all surgical), recycling of ES wires. Eco-responsible purchasing action: creating sustainable development questionnaire for medical device suppliers (Surgical medical devices referenced in hospital database). Training or information on the action was given during its implementation. Thirteen actions were evaluated: seven concerned waste reduction, five concerned waste sorting, and one concerned eco-responsible purchases. Seven actions concerned all the hospital OR, one concerned both OR and SPD, one concerned neurosurgery, one concerned coelioscopic surgery, one concerned urological robotic surgery, and one concerned the urological and orthopedic surgery departments |
| **Gynaecology** | Thiel 2018: Operating room - Magee-Womens Hospital University of New Mexico Hospital(Thiel et al., 2018) | Product level - Anasthesia[C1 Desflurane alone, C2 desflurane with N2O, C3 sevoflurane with N2O, C4 sevoflurane alone, C5 propofol only | Reduce carbon emissions | Desflurane is 2500 times more potent than is CO2. N2O, at 310 times the heat-trapping potential of CO2, is used as a carrier gas in conjunction with the use of either sevoflurane or desflurane although it can be safely excluded from surgery.28Sevoflurane, with 130 times the heat-trapping potential of carbon dioxide (CO2) on a 100-year time scale, is environmentally preferable to desflurane Propofol is an injectable anesthetic with limited GHG emissions, with impacts mainly from its production and delivery, and is sometimes used as the primary anesthetic for hysterectomy |
|  |  | Setting - Recycling [C1 Maximize recycling, C2 Maximize regulated medical waste, C3 Reusing cotton OR towels, C4 Switch to reusable linens, C5 Reprocess SUDs where possible, C6 Minimal instruments hysterectomy, C7 minimal materials and maximum reuse] |  | We identified recycling potential (***C1)*** in the initial study; these include spunbondmeltblown-spunbond plastics (drapes and gowns), hard plastic basins, metals and glass from pharmaceutical vials, and paperboard or paper used in packaging. ***C2*** At the time of the original study, UPMC was sorting most of their surgical waste away from red bag or hazardous waste. At UPMC, non-hazardous, or white bag, waste is sent to a sanitary landfill, and regulated medical waste is autoclaved before landfilling, adding extra treatment and emissions to the end-of-life scenario. We created the maximizing regulated medical waste intervention to determine the effect of this regulated medical waste diversion policy relative to disposing of surgical waste completely via the red bag treatment path. ***C3*** We assumed cotton towels to have a 10-use life span, and we assumed third-party linen laundering to be the sterilization pathway. Although life spans may be shorter or longer, this was the life span hospital staff estimated. Our estimates of energy and detergent use were from the original study. ***C4*** Reusable gowns and laparotomy drapes have an estimated life span of 75 uses and are sterilized between cases with laundering, drying, and autoclaving cycles, per manufacturer recommendations. In our estimates of emissions, we assumed that the sterilization process is conducted in-house (therefore, there are no off-site transportation emissions). ***C5,*** We identified reprocessable SUDs as the surgical instruments UPMC’s current third-party reprocessor can accept. These include endoshears (Medtronic, North Haven, CT), Carter-Thomason CloseSure System (Medline, Mundelein, IL), Versa-Port plus v2, 5 to 12 millimeter (Medtronic), LigaSure blunt tip laparoscopic sealer–divider 5 millimeter blunt tip laparoscopic sealer (Medtronic), LigaSure (Medtronic), and LigaSure Vessel Sealing 5 millimeter (Medtronic). We estimated emissions from reprocessing using values from previous literature.2 ***C6*** A panel of 3 practicing gynecologists at UPMC determined a list of the bare essentials of surgery; these include a uterine manipulator, a monopolar shears, a vessel sealer, a grasper, laparoscopic suturing equipment, suture, ports, and an insufflator. We calculated the environmental impacts from these single-use instruments using their purchase prices and the Economic Input Output LCA database.1 We assumed these supplies were single-use disposables (although reusable supplies do exist for some of these items) and that the original disposable custom pack (with single-use surgical supplies for laparoscopic hysterectomy) was still in use. Our gynecologist panel reported using this minimal supply set in at least one third of their laparoscopic cases. **C7** Combination of the following interventions: Bare minimum materials, Reusable linens and towels, Maximized recycling, Reprocessing was not available for items on the bare minimum list |
|  |  | Setting - Energy consumption [C1 Occupancy sensors installed for off-hours C2 Switch to maximum renewable energy C3 Combo: occupancy sensors and low-carbon energy grid mix] |  | ***C1*** installing occupancy sensors to minimize electricity and energy use during nonoperative or low-use times, Weather conditions, occupancy, equipment and OR size remain unchanged Does not include 20-min room turnover between cases HVAC settings in “energy saving” mode include a 40% reduction in air changes per hour (from 20 to 12) and a 15% reduction in temperature set point (from 20°C to 17°C). These are the lowest-energy operating conditions for the ORs at Magee, and may not be optimal for all HVAC designs25. ***C2*** switching to a low-carbon electricity source. Proposed energy mix (available through PG&E Corp Energy Company) is 2.2% oil, 35.7% nuclear, and 62.1% hydro, with GHGs averaging 0.05 lbs CO2 per kWh26 Total consumed kWh/h remained the same in each hysterectomy. ***C3*** Combination of C1+C2 |
|  |  | Waste management, product level, energy conservation |  | Optimized: ideal green hysterectomy: Combination: Sevoflurane only+Minimum materials+Maximum reusable materials+Maximum recycling+Occupancy sensors for low-energy ORs in off-hours+ Low-carbon electricity grid mix |
| **Setting** | | | | |
| **Multiple** | de Ridder 2022: Leiden University Medical centre(de Ridder et al., 2022) | C1 Multiple C2 Usual care | Reduce carbon footprint of caesarean section procedures | ***C2*** - As illustrated in case study: Preparation room: four categories of products enter 1) Instrument tray (contains reusable surgical instruments that are used during the procedure. Originates from the Central Sterile Supply Department within the hospital and is packed in polypropylene blue wrap), 2) Prepack (Custom pack used for every C-section. This is a pre-packed tray with sterile disposable items assembled especially for a specific kind of surgery), 3) Individually wrapped reusable products 4) Individually wrapped disposable products. Individually wrapped disposable and reusable products can be collected separately when requested by the surgeon. Packaging and other waste disposed of in preparation room in 3 waste streams 1) Residual waste (8): incinerated, 2) Paper waste (11): Incinerated - collected separately but treated aws MSW by waste handling company, 3) Plastic foils (3): recycled. Reuseable and disposable products used during surgery enter operating room. The disposables are disposed in 2 waste streams after use: 1) Residual waste (19): incinerated, 2) Regulated medical wase (1) incinerated at different waste incineration plant.  ***C1*** - 1) A multidisciplinary team is assembled ensuring diverse expertise and unbiased outcomes. The team leader oversees meetings and guides the process, aiming for 6-10 members for balanced input and effective discussion over 4-6 sessions. 2) The process flowchart is developed to provide a comprehensive understanding of waste generation, identifying sub-steps and waste streams through graphical representation. 3) Hazard analysis quantifies environmental risks associated with waste disposal, utilizing DEFRA greenhouse gas conversion factors and a decision tree to streamline the analysis process. 4) Action and outcome measures focus on sustainable solutions for waste reduction, applying the principles of 'reduce,' 'reuse,' 'recycle,' 'rethink,' 'refuse,' and 'refrain' to mitigate environmental impact and ensure stakeholder safety. 5) Calculations for carbon footprint and sustainability interventions are conducted using a spreadsheet, facilitating data entry and comparison between baseline and revised scenarios |
| **Gastroenterology** | López-Muñoz 2023: Hospital (Lopez-Munoz et al., 2023) | C1 Multiple: Reuseable equipment, recycling C2 No recycling | Evaluate composition & environmental impact of commonly used endoscopy instruments (biopsy forceps, polypectomy snares and haemostatic clips) from four different manufacturers, quantifying the parts that could be recycled | Biopsy forceps, polypectomy snares and haemostatic clips from four different manufacturers (A, B, C and D) were selected: biopsy forceps (A, B and C), polypectomy snares (A, B and D) and haemostatic clips (A and B). All instruments were analysed after the endoscopic procedure, adding a mark on the instruments to identify parts not in contact with the endoscope, outside the working channel, which could be recyclable. Our hypothesis to develop a sustainability intervention is based on one simple proposal: some parts of the instrument may not be considered as BMW. Parts of the instrument body and the handle are not in contact with patient fluids or secretions. Our proposal consists in taking apart the instrument after the procedure (upper from the mark), sending the handle and part of the body to recycle and the rest (in contact with the working channel of the endoscope) to BMW management. An experiment was conducted in our daily practice to mark the proximal part of the instrument body not in contact with the working channel. Marking of the sheath was made during 30 consecutive diagnostic endoscopic procedures to determine this contact mark for gastroscopy and colonoscopy. Mean, median, range and SD of distance from the instrument tip to the marked point of the instrument body were calculated. Although the device has not been inside the endoscope, it would still be in contact with the hands of the endoscopist and the assistant, with multiple passes. To reduce the potential risk contamination, 5cm away from the contact mark with the working channel was considered safe and marked as our recyclable mark or green mark (figure 1). After the procedure, in the same endoscopy room, instruments were cut into pieces with a wire cutter by the endoscopist |
| **Radiology/ Radiotherapy** | McAlister 2022: Hospital, Australia(McAlister et al., 2022) | Energy conservation | Support the more appropriate use of imaging | Different imaging modalities: ***C1*** Chest X-Ray, ***C2*** Ultrasound, ***C3*** Mobile chest x-ray scanner was located in the intensive care unit, St George's Hospital, Sydney, ***C4*** Computerised tomography, ***C5*** Magnetic resonance imaging (MRI) at Footscray Hospital in Melbourne, Australia |
| **Opthalmology** | Winklmair 2023: 3 Austrian hospitals^b^ (Winklmair et al., 2023) | Waste management [C1 Recycling  C2 100% incineration] | Compare material composition and carbon emission | Variability in cataract package composition across 3 Austrian hospitals considered. Compared environmental effect of recycling all technically recyclable materials |
| ^a^All surgical specialties (neurosurgery, otolaryngology, ophthalmology, orthopaedic, plastic, vascular, gynaecology, urology, and digestive surgery) and anesthesia were included in the study to involve all the professionals working in the OR and SPD. ^b^Hanusch Krankenhaus Wien, Barmherzige Briider Wien and Privatklinikk Hochrum. bpMRI=biparametric MRI, C=Comparator, DEHP=di(2-ethylhexyl) phthalate, F2F=Face to face, HD=Home Hemodialysis, ICHD=In-centre hemodialysis, mpMRI=multiparameter MRI, MRI=Magnetic Resonance Imaging, OR=Operating room, SD=Standard deviation, SPD=Sterile Processing Department, PVC=Polyvinylchloride, RD=Reuseable duodenoscope, TPU=Thermoplastic polyurethane | | | | |

## 6 Specialty specific findings and link to online interactive EGM

The map is intended as an interactive resource and we suggest that readers navigate the evidence and gap map, accessed here [https://eppi.ioe.ac.uk/cms/Portals/35/Maps/carbon-emissions-healthcare.html](https://eur03.safelinks.protection.outlook.com/?url=https%3A%2F%2Feppi.ioe.ac.uk%2Fcms%2FPortals%2F35%2FMaps%2Fcarbon-emissions-healthcare.html&data=05%7C02%7CE.H.Shaw%40exeter.ac.uk%7C63ed912b1fcb4de9fbf108dc78afadf7%7C912a5d77fb984eeeaf321334d8f04a53%7C0%7C0%7C638517943139935654%7CUnknown%7CTWFpbGZsb3d8eyJWIjoiMC4wLjAwMDAiLCJQIjoiV2luMzIiLCJBTiI6Ik1haWwiLCJXVCI6Mn0%3D%7C0%7C%7C%7C&sdata=kCaWvy0%2Fod%2Fgd6TKgaQE1YOxj4QNEfnRtHT4hqGiT6E%3D&reserved=0) and browse publications of interest.

## Broad intervention: Accessing care

### Telemedicine: Non-LCA studies

Six observational studies were conducted within oncology/radiation oncology services.(Beswick et al., 2016; Jiang et al., 2021; Lambert et al., 2023; Lewis et al., 2009; Patel et al., 2023; Thota et al., 2020) Studies showed the beneficial effects of telemedicine in reducing carbon emissions, however all studies based their carbon emission calculations solely on patient travel saved. Other outcomes categories considered included patient costs saved (n=4),(Beswick et al., 2016; Jiang et al., 2021; Lambert et al., 2023; Thota et al., 2020) patient time saved (n=3),(Beswick et al., 2016; Jiang et al., 2021; Lambert et al., 2023) clinician travel saved (n=1),(Lewis et al., 2009) time from evaluation/referral to treatment (n=1),(Beswick et al., 2016) patient petrol saved (n=1),(Patel et al., 2023) costs to services (n=1),(Lewis et al., 2009) and environmental costs (n=1).(Lambert et al., 2023) All outcomes favoured the telehealth intervention, with the exception of time from referral to surgery, which favoured face-to-face care in one study.(Beswick et al., 2016) The majority of comparisons between intervention and control groups were calculated using descriptive statistics and narrative techniques, with the exception being the calculation of patients costs within one study.(Lambert et al., 2023) Poor access to, or difficulty using, technology impacted patient satisfaction,(Jiang et al., 2021) with higher rates of telehealth utilisation associated with longer travel times, male gender and higher age.(Lambert et al., 2023) Descriptive statistics in two studies highlight the cost savings for services associated with the telehealth interventions.(Miah et al., 2019; Natale et al., 2022) Only one study statistically compared attendance/cancellation rates between telehealth and face-to-face control groups, demonstrating no significant difference between groups.(Natale et al., 2022) This study also demonstrated no significant difference in cancellation rates in patients aged above 50 years of age.(Natale et al., 2022)

Five observational studies were conducted within urology services.(Connor et al., 2019; Croghan et al., 2021; Filfilan et al., 2021; Miah et al., 2019; Natale et al., 2022) All studies reported carbon-emission reductions in relation to intervention implementation, however only one of these studies went beyond basing these calculations on patient travel data to also include patient and staff energy use.(Filfilan et al., 2021) Narrative/descriptive findings indicated the patient benefits associated with the telehealth intervention included travel saved (n=3),(Connor et al., 2019; Croghan et al., 2021; Miah et al., 2019), time saved (n=2),(Croghan et al., 2021; Filfilan et al., 2021), and reduced costs (n=2). (Croghan et al., 2021; Filfilan et al., 2021)

Four studies were conducted within the orthopaedics and/or trauma speciality, two observational,(Curtis et al., 2021; Richards et al., 2022) and two utilising an experimental comparative study design.(Arndt et al., 2023; Muschol et al., 2022) Two of these studies incorporated measures of power and/or technology use into their carbon emission calculations, alongside impact of patient travel saved.(Arndt et al., 2023; Richards et al., 2022) All four studies reported in favour of the intervention reducing carbon emissions. One study reported no statistically significant difference in patient satisfaction, adverse events or accessibility between individuals receiving a telehealth intervention vs those receiving face-to-face care,(Curtis et al., 2021) with another reporting no statistically significant difference in rates of patient attendance/cancellation or work absence.(Muschol et al., 2022) Two studies reported greater patient time saved for individuals receiving telehealth interventions.(Arndt et al., 2023; Curtis et al., 2021) Greater carbon reductions were found for individuals living further away from service.(Arndt et al., 2023) Access issues highlighted that half of the individuals completing the virtual care intervention were dependent on an escort,(Arndt et al., 2023) and that those with a higher level of disability were more likely to be unsatisfied with telehealth services.(Richards et al., 2022) However, the influence of patient age on patient satisfaction varied across studies. One study reported older patients being more likely to have difficulties accessing a virtual care intervention,(Curtis et al., 2021) whilst another reported a weak predictive value of greater distance travelled and age with increased overall satisfaction.(Richards et al., 2022) One study highlighted that the costs of follow-up appointments were greatest for unemployed patients.(Muschol et al., 2022)

Three observational studies were conducted within the renal speciality.(Andrew et al., 2020; Connor et al., 2011b; Udayaraj et al., 2019) All reported in favour of telehealth interventions (vs face-to-face care) in reducing carbon emissions, although these calculations were solely based on patient travel saved. Other outcomes measured relied on descriptive or narrative comparisons between intervention and control groups. The majority reported improved scores in the intervention group in relation to: patient travel distance saved (n=3),(Andrew et al., 2020; Connor et al., 2011b; Udayaraj et al., 2019) patient time saved (n=1),(Andrew et al., 2020) attendance/cancellations (n=1),(Udayaraj et al., 2019) patient costs saved (n=1),(Andrew et al., 2020) and service costs saved (n=1).(Udayaraj et al., 2019)

Two observational studies were conducted within gastroenterology,(King et al., 2023; Sillcox et al., 2023a), one of which incorporated emissions associated with software and infrastructure use into their carbon emission calculations.(King et al., 2023) Both reported reduction of carbon emissions associated with telehealth vs face-to-face visits, although one study noted no significant difference when adjusting for number of appointments and no significant difference between non-tertiary and tertiary delivery sites.(King et al., 2023) A statistically significant difference was found between intervention and control groups in favour of the telehealth intervention for patient travel distance saved,(Sillcox et al., 2023a) There was no statistically significant difference between groups regarding time from referral to surgery.(Sillcox et al., 2023a) Measures of attendance/cancellation demonstrated conflicting findings, either favouring face to face appointments,(47) of the telehealth intervention.(Sillcox et al., 2023a) Findings relating to adverse events differed between studies, with one reporting no statistically significant difference between groups,(Sillcox et al., 2023a) and one reporting no statistically significant difference between groups for 90 day admission/mortality rate, but favoured face-to-face care regarding number of blood test requests after appointment.(King et al., 2023)

Two studies were conducted within ENT services, one was observational,(Dorrian et al., 2009) and the other was a prospective comparative study.(Tselapedi-Sekeitto et al., 2023) Both based their carbon emission calculations on patient or staff travel saved, reporting reductions in carbon emissions due to a telehealth (vs face-to-face) intervention. One study reported no statistically significant difference in patient satisfaction between the two groups and provided descriptive statistics which indicated greater patient travel saved within the intervention group.(Tselapedi-Sekeitto et al., 2023) The other study provided descriptive statistics to indicate reduced time from referral to initial consultation in the telehealth group, and a statistically significant difference in favour of face-to-face care regarding service costs.(Dorrian et al., 2009)

One before and after study conducted within the cardiology speciality,(McLachlan et al., 2021) and one observational study conducted within gynaecological services,(Mojdehbakhsh et al., 2021) reported reduced carbon emissions within the telemedicine group. Neither study reported comparative data for any other outcome measure, aside from patient travel saved – which favoured the telehealth condition.(Mojdehbakhsh et al., 2021)

## Product Level

### Reuseable equipment: LCA studies

Five studies were conducted within urology,(Baboudjian et al., 2022; Davis et al., 2018; Hogan et al., 2022; Kemble et al., 2023; Wombwell et al., 2023) with the majority (n=4) comparing carbon emissions associated with the use of reuseable versus disposable cystoscopes.(Baboudjian et al., 2022; Hogan et al., 2022; Kemble et al., 2023; Wombwell et al., 2023) One of these was an LCA appraised as Low risk of bias,(Baboudjian et al., 2022) two studies were Medium risk of bias inventory analyses,(Kemble et al., 2023; Wombwell et al., 2023) and one was a High risk of bias study based on simplified LCA methodology.(Hogan et al., 2022) Three of the studies indicated that single-use devices were associated with reduced carbon emissions when compared to reuseable devices,(Baboudjian et al., 2022; Hogan et al., 2022; Wombwell et al., 2023) however the results of the High risk of bias study based on simplified-LCA methodology(Hogan et al., 2022) have been queried by Rizan and Bhutta (2022),(Rizan & Bhutta, 2022b) who raised concerns that the carbon emissions attributed to the reprocessing of reuseable cystoscope and manufacturing of a single use cystoscope were incorrect, due to an over-estimation in carbon emissions associated with the reprocessing of reuseable devices and incorrect use of the characterisation factor from a referenced study.(Davis et al., 2018) Rizan et al (2022) provided amended figures that reuseable cystoscopes are associated with reduced carbon emissions when compared to single use.(Rizan & Bhutta, 2022b) Hogan et al (2023) stand by their initial calculations, citating variation in reprocessing times and fuel mix across different contexts and attributing the difference in the characterisation factor they utilised, compared to those cited in Davis et al, to the different composition of cystoscopes vs ureteroscopes.(Davis et al., 2018; Hogan & Hennessey, 2023)

One Medium risk of bias inventory analysis also indicated that reuseable cystoscopes were associated with lower carbon emissions than single-use.(Kemble et al., 2023) Four impact categories were evaluated by the Low risk of bias study using LCA methods. Single use devices were associated with reduced environmental impact within Mineral resource depletion and Acidification impact categories, whilst no difference in environmental impact was observed within the categories Ecotoxicity and Eutrophication.(Baboudjian et al., 2022) One study indicated reduced environmental impact for solid waste for disposable devices.(Hogan et al., 2022) One inventory analysis appraised as High risk of bias compared reuseable with disposable flexible ureteroscopes, indicated no significant difference in carbon emissions associated with disposable versus reuseable ureteroscopes.(Davis et al., 2018)

Four studies were conducted within gastroenterology,(Boberg et al., 2022; Le et al., 2022; Rizan et al., 2020; Sherman et al., 2018) with equipment including trocar systems (n=1),(Boberg et al., 2022) duodenoscopes (n=1),(Le et al., 2022) laparoscopic surgery equipment (n=1),(Rizan & Bhutta, 2022a) and laryngoscopes (n=1).(Sherman et al., 2018) All studies were appraised as being as “High” (n=3),(Boberg et al., 2022; Rizan et al., 2020; Sherman et al., 2018) or “Medium” (n=1) quality,(Le et al., 2022) and indicated reduced carbon emissions associated with reuseable or hybrid instruments when compared with single use. All the impact categories included in the study evaluating single use vs reuseable laryngoscopes favoured reuseable equipment.(Sherman et al., 2018) Findings for the other impact categories for other equipment types were more varied, although the majority of environmental impacts associated with reuseable and hybrid equipment were either reduced, or not significantly different, when compared with single-use equipment.(Boberg et al., 2022; Le et al., 2022; Rizan et al., 2020) Two studies evaluated the impact of reuseable vs disposable equipment on cost, both concluding that reuseable or hybrid equipment cost less than disposable.(Boberg et al., 2022; Sherman et al., 2018)

One LCA appraised as Low risk of bias calculated the carbon emissions associated with newly-manufactured catheters vs remanufactured catheters within a Cardiology setting(2021) (Sanchez et al., 2020)reporting reduced carbon emissions associated with reuseable equipment. In contrast, one LCA appraised as Medium risk of bias evaluating single-use vs reuseable bronchoscopes reported that carbon emissions were reduced for single-use equipment or did not differ significantly between groups, depending on quantity of PPE and cleaning procedures used for reuseable bronchoscopes.(Sørensen & Grüttner, 2018) However, these findings have been queried by another study which highlights that whilst Sorensen and Grüttner (2008) acknowledge reuseable bronchoscopes are associated with reduced carbon emissions when compared with disposable bronchoscopes, and when two or more bronchoscopes are reprocessed together, they omitted this from main analysis.(Rizan & Bhutta, 2022b) The majority of the other impact categories evaluated for the first study favoured reuseable equipment,(Sanchez et al., 2020; Schulte et al., 2021) whilst the two impact categories associated with the study evaluating reuseable vs single-use bronchoscopes favoured single-use.(Sørensen & Grüttner, 2018)

## Setting

### Waste management non-LCA studies

Two of the four studies conducted within gastroenterology were before and after studies,(Betts, 2022; Neves et al., 2022) one was a modelling study,(Owens, 2023) and one was a retrospective observational study.(Yong et al., 2022) All interventions focused on methods of reducing waste associated with conducted endoscopies, including water bottle recycling,(Betts, 2022) improving waste segregation and recycling within endoscopy rooms,(Neves et al., 2022) reducing paper waste associated with patient information leaflets, questionnaires and reports and contrast,(Owens, 2023) and reducing number of plastic pots used for polyp removal.(Yong et al., 2022) Carbon reduction calculations focused mainly on carbon emissions associated with reducing quantity of waste created and/or disposed, without consideration of carbon emissions associated with recycling processes.(Betts, 2022; Neves et al., 2022; Owens, 2023; Yong et al., 2022) and hence indicated that waste management interventions were associated with carbon emission reductions. The only outcomes consistently measured across these studies were quantity of waste reduction (n=3),(Betts, 2022; Neves et al., 2022; Yong et al., 2022) and service costs (n=3),(Betts, 2022; Neves et al., 2022; Owens, 2023) all of which indicated beneficial effects of the intervention. Other outcomes assessed included patient clinical outcomes,(Betts, 2022; Neves et al., 2022) patient satisfaction,(Owens, 2023) clinician satisfaction,(Neves et al., 2022) social sustainability,(Betts, 2022) and fidelity to clinical process.(Yong et al., 2022) However, no comparative data was available for the majority of these outcomes, and thus did not support evaluation of the impact of the intervention.

Aims of other studies within this category included one modelling study estimating the impact of changing the composition of patient blood-testing kit for renal transplant/dialysis patient,(Bird, 2022) one retrospective cohort study evaluated an educational intervention focusing on reducing use of inhaled halogenated anaesthetic gases in individuals undergoing organ transplants, one before and after study evaluated an intervention aimed at reducing waste and materials associated with carpel tunnel surgery and an observational cohort study investigated the impact of reusing shipping materials used to package materials for intravitreal injections.(Vo et al., 2023) All were associated with reduced carbon emissions when compared with standard practice. System components/stages included within carbon emission calculations were often focused on one or two states e.g. use/reuse or waste disposal,(Chambrin et al., 2023; Kodumuri et al., 2023; Kodumuri, 2022) but two studies expanded on this to include all three stages and/or transport.(Bird, 2022; Vo et al., 2023) Other outcome measures included patient satisfaction (n=1), patient clinical outcomes (n=1),(Bird, 2022) clinician satisfaction (n=1),(Bird, 2022) cost saved,(Bird, 2022; Chambrin et al., 2023; Kodumuri et al., 2023; Kodumuri, 2022; Vo et al., 2023) waste reduced,(Kodumuri et al., 2023; Kodumuri, 2022; Vo et al., 2023) all of which favoured the waste reduction intervention.

## Care Delivery

### Treatment pathway-non-LCA studies

One controlled-trial within the orthopaedic and/or trauma speciality,(Cooper et al., 2022; Cooper et al., 2023) demonstrated “incremental” reductions in carbon emissions resulting from establishing a day-case treatment pathway for patients undergoing knee arthroplasty, which included changes to in-hospital care and a remote monitoring package. Carbon reductions were mainly associated with reductions in face-to-face contact. including number of face-to-face visits, hospital length of stay (LOS), service costs and number of physio appointments favoured the intervention group.

## 7 Accessing care - overview of non-LCA studies

**Overview of non-LCA studies – Telehealth interventions**

| **Specialty** | **Author, date: Country [Setting]** | **Aim** | **Study design: Intervention vs Comparator [Participants N]** | **Specific health condition: Feature** | **Intervention description** | **Carbon emission calculation methods** | **Boundary of system evaluated [Unit: CE Scope]** | **Carbon Emission findings** | **PROGRESS-PLUS** | **Patient satisfaction** | **Patient travel distance saved** | **Patient costs saved** | **Patient time saved** | **Time to referral** | **Attendance/Cancellation** | **Adverse event/Patient safety** | **Cost to service/staff** | **Other outcomes** | **Outcomes with no comparative data** |
| --- | --- | --- | --- | --- | --- | --- | --- | --- | --- | --- | --- | --- | --- | --- | --- | --- | --- | --- | --- |
| **Cardiology** | McLachlan 2021: New Zealand [HF service at Counties  Manukau District Health Board](McLachlan et al., 2021) | Using latest decision pathway for optimisation of HF treatment aims: facilitate titration, limiting F2F visits by using patient self-monitoring with package including funded home BP monitors, electronic scales & NP-led phone support | Experimental: Before and After: C1 Telehealth vs C2 F2F [52] | HF N(%) Of 50 patients: New diagnosis of HFrEF: (76%), History of CVD: 21 Hypertension: 34 Atrial fibrillation/flutter: 12 Obstructive sleep apnea: 6, T2DM (44%), non-concordance: 8, smoking: 10, Harmful alcohol use: 8, HbA1c (mmol/l): Mean 64 (43–100), BMI (kg/m2 ):Mean 32 (range 18–59), CKD (eGFR <50ml/min/1.73m2 ): 11 IICD in situ: 4 | Patients taught to identify fluid congestion/monitor vital signs with NP-led telephone support. Team introduced process. Self-help material included visual scale+book "Living Well with Heart Failure," for monitoring symptoms/vital signs. BP monitors+ electronic scales provided following practical demonstration. Booked fortnightly TC from NP/CNS agreed. Clinical support/guidance available from consultant cardiologist. F2F option if required. Each patient met HF member supporting them at beginning of trial. Some patients preferred email contact/text, TC most common. Up-titration facilitated by ePrescription+eLabform process | Accessibility benefits to patient from VC: distance travelled from patient’s home address to outpatient department (Google Maps). Petrol costs: Standard car petrol use. Travel time: off peak traffic volumes for conservative estimate time saved. Data collected included no.contacts | Patient travel [$/ patient. Total CO2: 3] | During trial period, 216 contacts made: 129 (60%) telephone and 87 (40%) face to face. By eliminating travel need estimated saved on average 2.12hrs and 73.6km/patient travel costs: $2,908 during pilot ($58.17/patient). Total CO2 emissions reduced by 607kg, which would require 27.9 medium-sized trees to absorb within 1yr | ***Gender*** Male: 38 (76%), ***Mean age*** all: 58.9yrs, ***Nationality*** Maori: 12 (24%), Pacific Islanders: 17 (34%), Others: 21 (42%). | NCD |  |  |  |  |  | NCD |  |  | PU, Cl.E./PH, AE/ PSa, AC |
| **Renal** | Andrew 2020: Australia [Royal Melbourne Hospital, renal transplant unit](Andrew et al., 2020) | Describe telehealth care model used to provide routine follow up to patients’ post-kidney transplant | Observ.: Retrospective review of database (CE data only): C1-Telehealth vs C2-F2F [45]^a^ | Kidney transplant: NR | Clinicians access Health Direct Videocall via web-link or desk-top icon on outpatient clinic room computers, patients access from any device with microphone & webcam. Sometimes, reviews conducted along with patient’s local dialysis facility, GP or nurse; other patients connect directly from chosen place. F2F reviews interspersed with telehealth, frequency depending on duration post-transplant+patient factors. Referrals predominantly internal+informal, offered to patients at physician’s discretion. Referral triage- assessment of: patient’s ability to self-monitor BP, HRa+weight, involvement of local dialysis facility, GP or nurse, technology access and internet reliability, distance from hospital or individual circumstances warranting telehealth | Telehealth patient and appointment data collated/ analysed using Microsoft Excel 2010. Distance between hospital and longitude/latitude of each postcode calculated using the Geo-Coded National Address File.^1^ Estimates CO2 emissions calculated based on United States Environmental Protection Agency calculation of 404g of CO2 emitted/mile (251g/km) for average passenger vehicle.^2^ Patient location data plotted on map, with size of marker proportional to frequency of appointments/ postcode represented | Patient travel [Tonnes CO2 equivalents: 3] | Estimated reduction GHG emissions: 51 tonnes CO2 eq | ***Place of residence:*** Approx. half all RMH kidney transplant recipients live in regional areas | NCD | C1>C2 (N) | C1>C2 (N) | C1>C2 (N) |  |  |  |  |  | PU |
| **Renal** | Connor 2011b: UK [The University Hospital of Coventry and Warwick-shire renal service: telephone clinic](Connor et al., 2011b) | Follow-up to renal transplant recipients over a 3yr period, outlines benefits of service to patients, providers, and environment, discuss provision of virtual care to patients with kidney disease and possibilities of more widespread adoption | Observ.: Cross-sectional: C1- Service delivery/ Telehealth vs C2 -F2F [30] | Kidney failure: Renal transplant patients attending 2xfollow-up TC | Service offered to patients at physician’s discretion. Most patients well known to department, all stable graft function. Patients receive quarterly clinic appointments-one remains F2F. Patient ringS through to clinician at time in appointment letter, which requests provide weight/BP readings. As with F2F consultations, blood tests undertaken beforehand; patients may attend family practice, city centre phlebotomy service, or local hospitals. 15min allocated/consultation. Clinic letters copied to patient, along with necessary prescriptions, blood test form, and next appointment details. Annual F2F consultation allows for physical examination (including urinalysis) | Data collected prospectively from 30 patients attending 2 consecutive telephone clinics. Each patient’s return journey length calculated from postcode using Google Maps. Calculated using DEFRA conversion factors specific to transport modality used to attend local clinic.17 Mean value of 8.05 kg CO2 equivalents (kgCO2eq) identified. Reduced physician travel across 2 sites to outlying clinics based on 20 clinics across two sites, assuming physician return journeys from site by car | Patient travel, clinician travel [kg CO2eq: 3] | Annual 350 TCs: estimated reduction in GHG emissions of 2,818 kgCO2eq. Reduced physician travel to outlying clinics: estimated annual total annual reduction GHG emissions of ([10x0.20487x2x 20.4]+[10x 0.20487x2x 36.2]) 231.8 kgCO2eq (where 20.4 and 36.2 are return distances to outlying clinics, in km, and 0.20487 is conversion factor for average-sized car. Further potential, carbon savings result from reductions in building energy use (e.g. Lighting/heating hospital waiting room) and staff commuting (e.g. outpatient nurses and reception staff). Annual carbon saving estimated: >three tonnes CO2 eq (Sufficient to fill 3xlarge detached houses) | NR |  | C1>C2 (N) |  |  |  |  |  |  |  |  |
| **Renal** | Udayaraj 2019: UK [North Bristol NHS Trust Renal Unit](Udayaraj et al., 2019) | Test introduction of tele-clinic service to reduce no. patients not attending F2F  clinics | Observ.: before and after, Iterative PDSA cycles: C1 – Telehealth vs C2 - F2F [185]^b^ | Kidney transplant: NR | PDSA cycles to test introduction of tele-clinic service. Codesigned the service with patients and developed prototype delivery model and tested over 2xPDSA improvement ramps containing multiple PDSA cycles to embed the model into routine service delivery | Miles calculated based on patient survey (Miles travelled to face-to face appointments combined with transport used, 57.7% response rate) | Patient travel [kg CO2: 3] | Among survey respondents: average distance travelled by patients to F2F appointments=36.4 miles. Tele-clinic saved 3527m of motorized travel in total=Saving of 1035 kgCO2. Actual reduction in travel distance and CO2 emissions will be higher as response rate to patient survey only 57.7% | NR | NCD | C1>C2 (N) |  |  |  | C1>C2 (N)) | NCD | C1>C2 (N)^C^ |  | AC, BTC, AD, PSn, UA, ISU |
| **Orthopaedics (and Trauma)** | Arndt 2023: Germany [Outpatient clinic of O+T surgery, German University Hospital](Arndt et al., 2023) | Compare estimated CO2 emissions in 6-months conducting VC with period of exclusive F2F outpatient clinic | Experimental: Before and After: C1 Telehealth vs C2 F2F [52]^d^ | Spinal surgery, joint surgery, paediatric orthopaedics, and accident surgery: Spinal surgery, joint surgery, paediatric orthopaedics, and accident surgery. Median age 51 [2-7-87.9yrs] | VC in the outpatient clinic | GHG emissions were assessed with CO2 calculations based on data from the German FEA. Avg emissions for car journeys were estimated at 143 g CO2eq/ person-km. Reduction of GHG emissions also considered emissions during VC and power consumption of data centres. Fiber optic technology was found to be the most environmentally friendly= 2 g CO2eq/hr of video streaming compared to 90 g CO2eq/hr for 3G networks. IF widespread VDSL connections in German homes, a 1-hour VC session= 4 g CO2eq | Use/reuse (power consumption), patient travel [g CO2eq: 3] | Significant difference between groups favouring intervention for CO2 reduction (p < 0.001). (Referring to Federal Environment Agency, implementation VC: reduction in GHG missions over 0.5 tons CO2eq for respondents VS patients traveling by car. Time patient and doctor involved during VC included: approx. 160 g of CO2eq for all VC | ***Place of residence:*** Among 51 respondents, 31% reported travelling <20km to the clinic and 35% reported travelling>50km. ***Personal characteri-stics associated with discrimen-ation:*** Half respondents (n=22) who completed VC dependent on escort | NCD |  |  | C1>C2 (N) |  |  |  |  |  | PSn |
| **Orthopaedics (and Trauma)** | Curtis 2021: UK [Orthopaedic emergency clinic in large district general hospital](Curtis et al., 2021) | Establish whether NF2F clinics sustainable according to “triple bottom line” framework by considering impact on patients, planet, and  financial cost | Observ.: Retrospective cohort: C1 Telehealth vs C2 F2F [180, 76 F2F, 104 TH] | NR: mean age was 48 (range: 3 months to 92 years), with 56% female and 44% male participants. Patient demographics did not vary significantly (p > 0.05) between groups | NR | Patients contacted by telephone and asked questions about mode of transport. Estimates of CO2eq made for each mode of transport | Patient travel [kgCO2eq: 1, 2 and 3] | Mean return journey distance (home to hospital): 18.6m. Reduced CO2eq: 65% car, 84% taxi, 57% bus due to NF2F clinics. Overall, total carbon emissions reduced: 563.9 kgCO2eq (66%) or 3.1 kgCO2eq/ person=2,106m driven in medium-sized petrol car. Outpatient carbon cost associated with each visit (heating, lighting, waste generated): 56 kgCO2eq /patient8=10,080 kgCO2eq for all 180 patients. Utilizing NF2F consultations for 104 patients, led to 58% reduction 5,846 kgCO2eq | ***Personal characteristics associated with discrimination:*** Those unable to use VC significantly older by 17 years (p < 0.001) | >< (S) |  | C1>C2 (N) |  |  |  | ><(S) |  | Accessibility ><(S) |  |
| **Orthopaedics (and Trauma)** | Muschol 2022: Germany [Single University hospital, Depart. Trauma, Hand and Reconst-ructive Surgery](Muschol et al., 2022) | Provide first health economic analysis comparing telemedicine in follow-up of patients in O+T surgery with knee and shoulder disorders with conventional F2F examinations in clinic in Germany | RCT: C1 Telehealth vs C2 F2F [60, 30 TH, 30 Control] | Knee and shoulder disorders: N (%). Medical indication C1 v C2: Knee - 10 (38) v 9 (35), Shoulder - Intervention 16 (62) v 17 (65); Age: 18-40 7(27). V 5 (19), 41-60, 17(65), v 15 (58), >60 2 (8) v 6(23); Female: 11 (42) v 10 (38); Employed: 20 (77) v 19 (76) | Intervention: replaced standard outpatient follow-up appointments with real-time online VC with treating physician. VC platform was browser-based for physicians and accessible via digital health apps or browser-based software for patients. The VC procedures were simplified to ensure practicality, involving direct communication between physicians and patients without involvement of other medical providers. Patients received written instructions for VC and incurred no additional costs, as the digital health app or software was free to use. Patients needed a device with microphone and camera capabilities and an internet connection, with examination costs covered by health insurance | 1) Environmental impact assessed by multiplying average emissions/ passenger-km by km travelled by car to/from clinic. Public transport emissions not calculated due to minimal usage in study. 2) Average environmental costs per passenger-km calculated using a cost rate from Federal Environment Agency. 3) Model estimates potential savings in emissions and environmental costs if 8 patients/week opt for VC instead of clinic consultations | Patient travel [Average emissions/ passenger-kilometre (pkm): 3] | Total emissions saved (26 patients in TH group): 292.448 kg. Use of TH saved approx. 3.73 in environmental costs per patient= 97.07 for all patients study. Potential savings for 1 year (8 patients/wk VC instead of clinic consultation): For 384 patients who would not have to travel to clinic each year: 4009.88 kg GHG, 24.80 kg of CO, 3.96 kg volatile hydrocarbons, 10.02 kg NOx, 0.16 kg particulates could be avoided. In addition, at 195/ ton CO2eq, 1330.91 could be saved. Environment costs could further be reduced by €2661.82, at £95/ton CO2eq, or by €6798.33, at €680 per ton of CO2 equivalent | ***Occupation:***  In the TH group, follow-up appointments cost €16.11 for employed patients and €5.85 for unemployed patients, contrasting Societal costs of lost production were €241.74 for full-time employees and €114.97 for part-time employees in the TH group |  |  | C1>C2 (N) | C1>C2 (S)^e^ |  | >< (S) |  |  | Work absence >< (S) |  |
| **Orthopaedics (and Trauma)** | Richards 2022: UK [Hospital (Musgrove Park, Taunton)](Richards et al., 2022) | Aimed to examine outcomes of Virtual Arthroplasty Follow-Up service and benefits for trust, patients, and planet | Observ.: retrospective cohort: c1 Telehealth vs c2 F2F [132 of 240 eligible]^f^ | Hip arthroplasty: Hip arthroplasty. Patient survey group (n=52). Mean age: 75.5. Mean oxford hip score: 836.9/48, Mean UCLA activity score 4.19 | First follow: VC 6wks post op. 2nd virtual follow up: 1yr post op. 7-yr follow up only for patients who received initial op under 50yrs of age. 10yr follow up: all patients. If patients>80yrs at 10yr follow up can be discharged to GP. All others to continue follow up at 13yrs. At 13yrs: all patients over 80 discharged. Further follow up in 3yr intervals for<80yrs. Key changes from F2F: key changes were a virtual follow-up (via telephone) at 1yr and removal of 5yr follow-up for all patients to a seven-year follow-up for patients who had their index operation performed at age <50yrs | 52 patients surveyed travelled average 24.6 miles total (to/from the hospital) for clinic appointments=1,279 miles not driven. If all drive equivalent of average petrol car=358kg CO2eq saved.^3^ Additionally, use of F2F clinic space has associated environmental cost (lighting, heating, waste generated), previously calculated between 56 and 76kg CO2eq/clinic slot.^4,5^ | Use/reuse (clinic space), disposal, patient travel [kg CO2 e: 1, 2, 3] | If all patients drive equivalent of average petrol car: 358kg CO2eq saved. Additionally, using F2F clinic space has associated environmental cost (lighting, heating, waste generated), previously calculated between 56 and 76kg CO2eq/clinic slot^4,5^ between 2,912 and 3,952 kg CO2eq for this group=total carbon saving for 52 patients to a lower estimate of 3,270kg CO2eq or 62.9kg CO2eq/patient per appointment. Upscaling these averages to all 132 VARF patients equates to over 8 tonnes CO2eq saved | ***Place of residence/ge:*** Age and distance= weak correlations with satisfaction levels. Patients with lower scores on the OHS= 9x more likely to be neutral or unsatisfied with the service. There was a moderate correlation between UCLA activity score and overall satisfaction | NCD |  | C1>C2 (N) |  |  |  |  |  |  | ACS, PF, QOL, PS, ACY |
| **Ear, Nose and Throat** | Dorrian 2009: UK [Remote tele endoscopy in Gilbert Bain Hospital Shetland](Dorrian et al., 2009) | Feasibility study to establish whether ENT tele-endoscopy suitable for service delivery for patients living in Shetland Island | Observ.: Prospective cohort: C1 Telehealth vs C2 F2F [42] | Head and neck cancer: Symptoms of possible head/neck cancer. Otherwise, NR | Patients referred from primary care selected for pilot study by consultant otolaryngologist in Aberdeen and two local doctors on Shetland. Laryngoscope (ENF GP Rhino-laryngoscope, Olympus) connected to videoconferencing unit via S-video cable. Video conferencing with Aberdeen conducted at 384 kbit/s via ISDN network. First 20 patients followed up after 2 and 6 months to confirm patient safety. Initially tele-endoscopy images recorded in Aberdeen and captured on DVD recorder on theatre stack used for endoscopic examination. This allowed ENT consultant to compare images for diagnostic accuracy. After first two clinics, ENT consultant decided live videoconferencing images supported accurate diagnosis | CO2 savings from avoided travel were calculated based on the DEFRA guidelines. journey from the hospital Shetland Islands to the specialist centre in Aberdeen’s Road distance calculated using a standard route planner, the distance between airports determined using Vincenty’s formula. TH enabled an ENT consultant to see 42 patients remotely. Emissions from a car with an average-sized diesel engine calculated at 0.199 kg CO2/km. Considering air travel, the emission from the short domestic flight was estimated at 0.158 kg CO2/km | Staff travel [kg CO2: 3] | Avoided road travel saved 9.15kg CO2 emissions and 0.158 kg CO2/km from a short (domestic) flight. Avoided air travel saved emission of 52.2kg CO2. Total saved emissions for journey: 61.3kg CO2/person (one-way) or 123kg CO2/person (return) | NR | NCD |  |  |  | Initial consultation C1>C2 (N) |  | NCD | C2>C1 (S)^g^ |  | PS, DD, ACS, AE |
| **Ear, Nose and Throat** | Tselapedi-Sekeitto 2023: Canada [otolaryngology clinic, Hospital, London Ontario](Tselapedi-Sekeitto et al., 2023) | Investigate patients' satisfaction, travel cost, productivity loss, and CO2 emissions  involved with synchronous virtual care and in-person assessments in rhinology and sleep apnoea clinics | Prospective comparative study: C1 Telehealth vs C2 F2F [94, 34 TH, 60 F2F]^h^ | Rhinology pathologies e.g. chronic rhinosinusitis, nasal septal deviation, sleep apnoea, allergic rhinitis, or post-operative rhinosinusitis: In the virtual care  group; mean age was 48 ± 16 years, 14 (42.4 %) males and 19 (57.6 %) females, while in the in-person group, mean age was 51.4 ± 19, 35 (58.3 %) males and 25 (41.7 %) females | NR | Carbon footprint and environmental impact assessed based on CO2 emission, calculated as 252.5g CO2/km (travel distance - round trip), expressed in Kg/consultation | Patient travel [kg CO2: 3] | Carbon footprint analysis showed an environmental impact generated by in-person group visits of 32 ± 39 kgCO2 emitted/consultation | ***Personal characteristics associated with discrimination*** Among 7 domains evaluated by PSQ-18, satisfaction with "Time spent with the doctor" correlated directly with age in the in-person group (r = 0.27; p = 0.037). In a subgroup analysis based on diagnosis, patients with allergic rhinitis had significantly lower general satisfaction scores in VC visits vs in-person visits (3.28 vs. 4.25, p = 0.04) | >< (S) |  |  | C1>C2 (N) |  |  |  |  |  | PPL |
| **Urology** | Connor 2019: UK [Tertiary centre: Virtual uteric colic clinic](Connor et al., 2019) | Evaluate clinical, fiscal and environmental impact of specialist-led acute ureteric colic virtual clinic pathway | Observ.: Prospective cohort: C1 Telehealth vs C2 F2F [1008] | Ureteric colic: 763 male, 245 female. Mean age: male 40.5 (13.1), female 32 (5.7). Majority of patients referred were of working age: 702 men (92.0%) and 220 women (89.8%) | VC TC: by specialist nurse/consultant urologist via patient’s personal mobile or landline. VC pro-forma used; approx. 15 min. Attempt to contact patient min. 3 times. Following this, patients deemed ‘did not attend’. VC, letter sent to patient/GP+ documented on patient records. Documentation shared with referrer. VC patient outcomes: discharge investigations and further VC, FTF clinic or direct referral for stone intervention (PCNL, URS or ESWL). VC supervised by 3xurologists. In case of clinical uncertainty, patient referred to FTF clinic. Adverse events (repeat presentation with sepsis, obstruction) +complaints logged. Min. follow-up 3 months. Working age defined 18– 65 yrs | Carbon footprint generated on patient mode of transport: train or car. Department of Transport vehicles analysis used example of 1800 cc petrol engine car to calculate presumed journeys^.6^ Total trip from patient’s home to hospital calculated using patient’s residential address. Distance calculated using Google Maps. Carbon footprint using calculator supplied by Carbon Footprint.^7^ No. trees required to offset calculated carbon footprint derived using FTF clinic as alternative and inputting data into the published agricultural algorithm provided by ‘Trees for the Future’^8^ | Patient travel [Metric tons CO2EQ: 3] | Mean (IQR) patient distance travelled: 4.3 (2.5–6) miles. Carbon footprint attributable to travel avoidance: 0.70–2.93 metric tonnes of CO2eq production (depending on transport mode). To offset this carbon footprint would require planting 14.7 trees | NR |  | C1>C2 (N) |  |  | NCD |  | NCD | C1>C2 |  | TR, TS, AE, SC, PH |
| **Urology** | Croghan 2021: Ireland [Urban tertiary referral unit- virtual clinic](Croghan et al., 2021) | Evaluate impact of virtual outpatient clinics on travel time, monetary cost to patients and carbon emissions | Observ.l: Cross-sectional: C1 Telehealth vs C2 F2F [1016, 736 TH] | General urology and subspecialist (including uro-oncology, urolithiasis and female, functional and reconstructive: NR. Of virtual care patients, 40% (295 of 736) considered ‘rural-dwelling’. Mean age: 62.9 yrs (range 18–95), significant portion of patients were of ‘working age’; 54.7% (403 of 736) 66 years old or younger, the state retirement age, 68.75% (506 of 736) aged 70 years or below | Outpatients triaged to determine clinical urgency/appropriateness of virtual review. F2F consultations scheduled when required. Any necessary imaging or blood tests arranged in advance of VC. VCs performed by telephone, although video-conferencing platforms available. Symptoms discussed, investigation or treatment plan agreed upon with patient, and documentation of interaction recorded in chart. A letter to patient’s GP is generated, and a prescription, where required, posted to patient | Patients' usual mode of transport to hospital visits was determined during clinical consultations. Travel time, petrol, toll, and parking costs, along with carbon emissions for hospital attendance, were calculated for each patient based on this information. AA Route Planner and Google Maps were used to calculate travel distances and times. Carbon emissions were estimated using an online calculator from Carbon Footprint Ltd, and the number of trees required to absorb emissions was calculated using information from the Tree Council of Ireland. Public transport carbon emissions were not calculated | Patient travel [tonnes CO2: 3] | Establishing VC: estimated reduction 6.07 tonnes CO2 emissions, based on predicted carbon footprint of ‘car traveller’ patients. Estimated  volume CO2 emissions would take 434 established (10-year-old) evergreen trees to absorb in 1yr | ***Age:*** Significant portion of patients were 'working age'  ***Place of residence:*** distance saved: Overall 31,038 miles (49,951km)/rural-dwelling patient 93.8 miles (151km). |  | C1>C2 (N) | C1>C2 (N) | C1>C2 (N) |  | NCD |  |  |  | Cl.E, AT |
| **Urology** | Filfilan 2021: France [Teleconsultations for 2 academic urology departments in Paris](Filfilan et al., 2021) | Assess environmental cost of urology teleconsultation vs F2F consultations | Observ.: Cross-sectional: C1 Telehealth vs C2 F2F [80] | Oncological (n = 49; 61%), functional urology (n = 14; 18%) and benign prostatic hyperplasia (n = 13; 16%): Reason for consultation: oncological (n = 49; 61%), functional urology (n = 14; 18%) and benign prostatic hyperplasia (n = 13; 16%). Median age [IQR] was 66 years [56—71], 10 patients were female (13%). 20 (25%) new patients | Teleconsultations led by 5xsenior urologists and had been introduced in these departments for first time 1 month previously as a response to COVID-19 lockdown. Teleconsultation performed using website doctolib. Patients who lived in another country excluded from to have better far distance homogeneity | TC: Energy consumption was calculated for a 20-minute live video connection, assuming 15 minutes for consultation and 5 minutes for administrative tasks. Energy usage was converted to CO2eq using French National Environmental calculator conversion factors. Estimated energy consumption for F2F included travel mode and distance. For car journeys, Car emissions were estimated using average diesel car emissions. Carbon and equivalent costs of public transport were evaluated using national French railway company emissions. Patients walking was considered emission-free | Use/ reuse, patient travel [CO2eq: 2 and 3] | Estimated CO2eq emissions avoided due to lack of travel: 1.1 tonnes during 1-month study period. Teleconsultations (two computers connected) responsible for 1.1 kg CO2eq emissions vs in-person consultations (1 computer used by consultant): 0.5kgCO2CO2eq. Total reduction GHGs: 1141 kg CO2eq, a 99% decrease in emissions | NR | NCD |  | C1>C2 (N) | C1>C2 (N) |  |  |  |  |  | PS, CS |
| **Urology** | Miah 2019: UK [Urology clinic](Miah et al., 2019) | Quantify clinical, financial and environmental benefits of virtual urology clinic | Observ.: Prospective cohort: C1 Telehealth vs C2 F2F [409] | Purpose: Venous biochemistry review; Venous haematology review; Radiological investigation review; Symptom review; Pathology review: 281 male (mean age 60 yrs), 128 female patients (mean age 61.5 yrs. Majority patients, male and female (n = 162, 57.7%, and n = 71, 55.5%, respectively) working age | TH using either patient’s landline or mobile number. All patients selected for VC follow-up made aware of this method of follow-up consultation in their prior F2F clinical encounter and agreed to use it. They were given number of administrative team to raise any concerns regarding VC. Patients made aware of protocol if they were not contactable on multiple attempts. Patients scheduled for results review that unable to contact were provided with a letter explaining result and plan of action; sent to both patient and GP. Alternatively, patient offered F2F clinic. VC undertaken by middle-grade urologist under supervision of named consultant available for clinical concerns/ queries. Adverse events complaints log performed following min. 4mnth period after each VC | Calculated range for carbon footprint generated. For journeys by car, selected 1800 cc petrol engine car Total travel distance for each patient calculated on round-trip from patient’s residential address to institution using Google Maps and selecting car as mode of travel. Estimated carbon footprint calculated using calculator supplied by Carbon Footprint. No. trees required to offset carbon footprint generated by alternative F2F clinic calculated using need to plant five trees for one to mature into an adult (Trees for the Future) | Patient travel [CO2 tonnes: 3] | Estimated avoided carbon footprint due to travel: 0.35–1.45 metric tonnes CO2eq. Predicted 12-month avoided carbon footprint: 1.05–4.35 metric tonnes of CO2eq. No. trees needed to be planted to offset carbon production: 1.75–7.25 trees. No. to be planted to offset higher estimate of predicted 12-month carbon production: 21.75 | NR | NCD | C1>C2 (N) |  |  |  |  |  | C1>C2 (N) |  | PS |
| **Urology** | Natale 2022: UK [Urology unit SW England](Natale et al., 2022) | To determine whether standalone tele-consultation is effective alternative to F2F assessment of patients requiring circumcision. Determine environ-mental and efficiency benefits result from service alteration | Observ.: Retrospective cohort: C1 Telehealth vs C2 F2F [101, 42 TH, 59 F2F] | Circumcision: Circumcision. Mean age: TC 36, F2F 50. Charlson comorbidity index <1 TC 72%, F2F 38%. Smoker TC 13% F2F 10%, Obesity TC 11% F2F 31e%, Diabetes mellitus TC 11%, F2F 24% | Arranged TC with day-of-operation consent and examination | Crude estimation of CO2 emission conducted using Environmental and Social Sustainability in NHS Innovation toolkit^9^ | NR [kgCO2: CT] | For studied cohort, estimated 3647 kg CO2 generated. A further 6897m3 of water used and 43 kg of waste produced. Per patient this equates to 45kgCO2, equivalent to driving from London to Sheffield. Had all patients been seen in a telephone clinic estimated reduction of 637kgCO2 could have been achieved | ***Personal characteristics associated with discrimination:*** to control for older, more comorbid population in F2F group, subgroup analysis performed for patients aged over 50yrs (N=43, 12 TC vs 31 F2F). No sig. difference in odds of cancellation (p=0.28) |  |  |  |  |  | >< (S) |  | C1>C2 (N) |  |  |
| **Oncology** | Beswick 2016: USA [Tertiary otolaryngology facilities- 2 Remote VHA sites: ENT/ Oncology](Beswick et al., 2016) | To evaluate telemedicine model utilizing AV teleconference as a preop. visit | Feasibility study/ Retrospective cohort: C1 Telehealth vs C2 F2F [21, 15 patients full protocol (pre to post op] | Head and neck cancer: Total 47. Remote patients (21): Pathology, no.  Carcinoma 5, Warthin’s tumour 3 ,Low-grade salivary neoplasm 3, Osteoradionecrosis 1, Substernal goitre 1, Cystic lesion 1, Low-grade laryngeal chondrosarcoma 1; F2F patients (26). 24 with high-grade neoplasms (carcinoma 5; melanoma 5; & metastatic thyroid cancer 5 1) and 2 with low-grade pathology (atypical fibroxanthoma 1, and osteoradionecrosis 1) | Eligible patients were offered telemedicine consultations upon referral, alongside standard in-person consultations. The telemedicine protocol involved tissue diagnosis and imaging acquisition at a remote site, followed by a review of clinical data and discussion at a multidisciplinary tumour board. Preoperative counselling was conducted via TH, involving the patient, nurse, and speech pathologist, facilitating nasopharyngoscopy. Surgical patients received preoperative clearance during telemedicine visits, with referrals made electronically for necessary evaluations. Operative intervention and immediate postoperative care were provided at the local tertiary site, with routine follow-up and additional telemedicine visits as needed. Non-operative patients received treatment in their home area or were referred to appropriate specialists | Parameters related to patient's treatment timeline calculated, including time from referral request to time of telemedicine consultation and time from telemedicine consultation to intervention. Travel time based on average driving or flying time from remote locations to hospital. Cost of travel and procedures based on federal government's reimbursement rate for travel^10^ and calculations by VHA finance department when determining cost of the fee based on specific procedures. CO2 emissions calculated from the Environmental Protection Agency's formula and based on road travel in a car or light truck/ patient | Patient Travel [CO2 tonnes: 3] | Intervention prevented 14.5 metric tons CO2 emissions based on Environmental Protection Agency formulas^11^ | Remote patient  ***Mean age*** 64yrs ( 28–95 yrs) ***Gender:*** all men In person patients. Mean age NR (range NR), gender NR, |  |  | C1>C2 (N) | C1>C2 (N) | Time to referral C1>C2 (N), evaluation to OR/surgery C2>C1 (N) |  |  |  |  |  |
| **Oncology** | Jiang 2021: USA [Ann Arbor Veterans Affairs Medical Center](Jiang et al., 2021) | To better understand tele oncology’s potential to facilitate VHA-based care, Assess Veteran views & satisfaction with technology. Generate estimates of private and social, financial, and environmental impacts to inform future policy trade-offs | Observ.: Retrospective cohort: C1 Telehealth vs C2 F2F [100 out of 366 eligible initial survey, 42 follow up survey] | Cancer, N.S.: For 366 eligible vs 100 surveyed. Primary site of disease^i^: Bladder 8 v 2, Breast 10 v 4, CNS 1 v 0, Endocrine 1 v 9, GI/ Hepatobiliary 110 v 23 Testicular 3 v 0, Head/neck 23 v 4, Lung 82 v 18, Neuroendocrine 9 v 4 Prostate 93 v 29, Renal 6 v 3, Skin/Soft tissue 20 v 9 | Oncology encounters completed via tele oncology (video visits, telephone, secure messages, electronic consults) from March to June 2020. Types of tele oncology visits: audio-only, audiovisual only, or both audio-only and audiovisual visits | Travel distance and time estimates between patient ZIP code and VAMC Ann Arbor generated using Google Maps, selected for shortest time if multiple routes available, and multiplied by two to estimate round-trip distance. Automobile CO2 emissions served as proxy of carbon footprint. CO2 emissions estimated as product of round-trip distance and mean automobile CO2 emission of 411 g CO2/mile, converted to metric tonnage.^12^ Refer to Data Supplement for additional information on calculations for financial and environmental impacts | Patient travel [Metric tons CO2 emissions: 3] | 560 total tele oncology encounters conducted between March 2020 and June 2020 saved geographically diverse patient population cumulative 86,470m travel (mean 154.4 m/patient encounter). Transitioning to tele oncology responsible for estimated carbon footprint reduction:35.5 metric tons CO2, approx. 106 metric tons of CO2 on an annualized basis in savings | For 366 eligible v 100 surveyed  ***Race/Ethnicity:*** Included population - White/Caucasian 288 v 76 Black/African American 38 v 11, Hawaiian or Pacific Islander 6 v 3 Did not declare 34 v 10 ***Personal characteristics associated with discrimination:*** Poor access to or difficulty using technology impacted patient satisfaction ***Mean age:*** 68.7(SD9.3) v 68.6(SD 8.8). 16(4.4) female v female | NCD | C1>C2 (N) | C1>C2 (N) | C1>C2 (N) |  |  |  |  |  | PSn |
| **Oncology** | Lambert 2023: Canada [CancerCare Manitoba, provincial agency](Lambert et al., 2023) | Describe patterns of visit types (in-person versus virtual) during pandemic at CancerCare Manitoba, and impact of virtual visits on hypothetical travel distance, travel time, and CO2 emissions generated by travel | Observ.: retrospective database review: C1 Telehealth vs C2 F2F [Total visits: 306, 234, In person: 160,668, Virtual: 145, 566] | All visits for invasive and in situ cancers: Age group (N): Under 18: 5198, 18–39: 13,805, 40–64:108,457, 65–79: 137,951, 80+: 40,823. Gender (n, %) Women 147,178, Men 159,043, Other 13. Cancer site- Breast: 41,178 (13.4), Digestive: 45,957 (15.0) Gynaecologic: 21,514 (7.0), Haematology: 45,879 (15.0), In situ and benign: 23,774 (7.8) Men’s genitourinary: 42,571 (13.9), Respiratory: 30,093 (9.8), Other: 55,268 (18.0) | Telehealth provides videoconferencing through many facilities across Manitoba for health care services, continuing education, meetings, and family visits. For some appointments, instead of an in-person visit, individuals could remain in their homes and interact with health care providers through telephone and videoconferencing. This was in addition to the telemedicine already used in Manitoba (Manitoba Telehealth) prior to pandemic where individuals could travel to a health care facility and have a videoconference with a health care provider at another facility. Manitoba Telehealth visits combined with in-person visits because of requirement of travel to health care facility | Estimated travel distance converted into estimated metric tons of CO2 emissions: 206g CO2/km=average based on newly registered vehicles in 2017 in Canada^13^ | Patient travel [CO2 tonnes: 3] | Estimated CO2 emissions prevented during study period varied from 87 to 155 metric tons/month  ***PROGRESS PLUS***  ***Place of residence:*** Northern Manitoba residents longest travel distances and times for cancer care, with Northern region often having lowest rates of VC, though this trend changed in part during 2021. Interlake–Eastern RHA, had among lowest monthly rates of VC | ***Gender:*** Men had higher rates of VC, with small differences observed during COVID-19 lockdowns. ***Age:*** *%* of VC visits increased with age, particularly between ages <18 vs 18- 39 |  | C1>C2 (N) | C1>C2 (S) | C1>C2 (N) |  |  |  |  | Environmental cost C1>C2 (N)) |  |
| **Oncology** | Lewis 2009: [Bronglais General Hospital, Aberystwyth](Lewis et al., 2009) | Evaluation of environmental impact of using VC vs meeting in person | Observ.: Cross-sectional:C1 VC vs C2 F2F [60 clinicians, 21 meetings] | Cancer: NR | Telemedicine service launched in Sept 2005 to assist meetings between MDT to improve cancer services. Implemented throughout the network | In Oct 06 and Oct 07, users of VC equipment at hospital completed questionnaires to quantify if time/travel costs reduced by attending meetings via VC vs F2F. Questionnaires recorded distance travelled, journey time, vehicle engine size, petrol/diesel, no passengers, and staff group. Journeys converted into CO2 savings using UK government calculator.^14^ Journey cost calculated from financial information provided by Trust finance department | Patient travel [kgCO2 emissions: 3] | During October 2006 total of 18000km car travel avoided, equivalent to 1696kg CO2 emission. During October 2007, total of 20800km car travel avoided, equivalent to 2590kg CO2 emission. Estimate 48 trees would take 1yr to absorb that quantity CO2 | NR |  |  |  |  |  |  |  | C1>C2 (N) | Clinician travel saved C1>C2 (N) |  |
| **Oncology** | Patel 2023: USA [single-institution National Cancer Institute-designated comprehensive cancer centre](Patel et al., 2023) | To assess carbon savings achieved from telemedicine visits | Observational: Cross-sectional: C1 vs C2 F2F [49329 TH visits, 23,228 patients] | NR: NR: For patients with visits within 60min vs >60min of driving time, median (IQR) age was 62.0 years (52.0-71.0 years) v 67.0 years (57.0-74.0 years), 12 334 v 13 468 of the visits were female, and 9934 v 10 217 of the visits were by patients privately insured; 1685 v 1056 were Black, 1500 v 1364 were Hispanic, and 16 010 v 22 457 were non-Hispanic White | Telemedicine: real-time care delivered through a synchronous videoconferencing. Starting in April 2020, instituted a synchronous video platform (Zoom Meetings) for telemedicine visits | All patients within Florida assumed to travel round-trip by car from their home addresses to MCC. Addresses were geocoded to calculate driving distance using the Buxton Company's analytics platform. CO2 emissions savings for vehicle travel were estimated using the EPA emissions calculator, with emissions per mile ranging from 386 g to 435 g. | Patient travel [kg CO2: 3] | Patients within a 60-minute driving distance from MCC saved over 1 million round-trip miles through telemedicine, resulting in 424,471 kgCO2 emissions savings. Each visit saved an average of 48.1 miles and 19.8 kgCO2 emissions. For those living more than 60 minutes away, approximately 6.7 million miles were saved, corresponding to 2,744,248 kgCO2 emissions savings. Each visit for these patients saved an average of 239.8 miles and 98.6 kgCO2 emissions. Patients living over 60 minutes away had about six times more CO2 emissions savings. Using different emissions per mile estimates, savings ranged from 398,651 to 449,257 kg for patients within 60 minutes and 2,577,323 to 2,904,496 kg for patients living over 60 minutes away | ***Place of residence:*** Subgroups divided based on driving time of 60 minutes or less vs greater than 60 minutes for further analysis to determine CO2 emissions saved between the 2 groups [See CE column]. ***Race/ ethnicity:*** 1685 (7.8%) were for Black patients, 1500 (7.0%) for Hispanic patients, and 16 010 (74.5%) for non-Hispanic White patients |  | C1>C2 (N) |  |  |  |  |  |  | Patient petrol saved C1>C2 (N) | Patient electricity and energy use, equivalent carbon sequestration (tree seedlings grown/10 years and acres US forests/1 yr |
| **Oncology** | Thota 2020: USA [Telehealth oncology clinic: Sevier Valley Hospital linked to tertiary medical centre](Thota et al., 2020) | Can telehealth between a tertiary cancer centre and rural health systems improve access to cancer care, decrease financial burdens, save time for patients with cancer living in rural Utah, and support local health delivery systems? | Observ.: retrospective cohort: C1 Telehealth vs C2 F2F [119 patients, 1025 encounters] | Cancer: Diagnosis - Malignant Haematology: 18%, Solid tumours, local: 23%, Solid tumours, Metastatic: 35%, classical haematology: 24% | Patients seen by medical oncologists, certified oncology nurses, and other subspecialists and ancillary staff via TH. Sustaining TH: Identify local providers to manage cancer care with support from a consulting oncologist, arrange TH enabled clinic rooms for synchronous video-based calls between tertiary and rural-based facilities, collaborate with patient navigators, social workers, palliative care, and cancer network services, ensure nearby and adequate emergency medical support, Support ongoing evaluation and treatment with local laboratory, radiology, and infusion services, provide ongoing administrative support to ensure compliance and implement regulatory changes, safely administer chemotherapy and immunotherapy under supervision of certified oncology nurses and oncologists | Travel hours and mileage saved calculated using Google Maps to estimate driving distance from Sevier County to IHC oncology facility emissions calculated using data from US Environmental Protection Agency, which calculates driving 1 mile=404 g carbon emissions^15^ (based on average 8,887g CO2 generated/gallon gasoline and 22.0m travelled/ gallon consumed for typical passenger vehicle^15^ | Patient travel [kg CO2: 3] | Intervention reduced carbon emissions by approximately 150,000 kg for 119 patients over 4-year period | NR |  | C1>C2 (N) | C1>C2 (N) |  |  |  |  | NCD |  | SP |
| **Gynaecology** | Mojdehbakhsh 2021: N [GynOnc Carbone Cancer Center  clinic at University of Wisconsin School of Medicine and Public Health](Mojdehbakhsh et al., 2021) | Within 1wk of initial intervention, convert min 50% of all F2F outpatient encounters to TH, 100% documentation of TH consent in providers’ notes 3) Elicit patient feedback regarding TH and determine potential impact on patient care. 4) Calculate amount of CO2 emissions prevented | Observ.: Cross-sectional: C1 Telehealth vs C2 F2F [192, Of which 113 responded (217 encounters)] | NR: Based on 192. Mean age 63,42 (SD 13.26). Primary Cancer site: Uterus 80 (41.7%), ovary 62 (32.3%), Fallopian tube 13(6.8%), cervical 11(5.7%), vulva/vagina 5(2.6%), peritoneum 4(2.1%), GTN 2(1.0%). Stage: one 72(37.5%), two 17(8.9%), three 47(24.5%), five 27(14.1%). | Using PDSA cycles, initial intervention agreed upon at meeting of GynOnc physicians. Meeting topics included: institutional TH platform, location of instructions and available support; visit coding in EHR, time & consent documentation; workflow to transition encounters to TH using visit schedulers. **Initial intervention**: all GynOnc providers convert appropriate surveillance visits to TH format through clinic scheduler. 1wk after, data analysed and fell short of primary and secondary aims, therefore second PDSA cycle implemented with more intervention over a two-day period, including: 1) APPs and nursing staff leading conversion to TH visits with physician approval; 2) implementation of standardized TH note template; 3) additional review of coding in EHR, time and consent documentation | Distance from patient’s home address to the Carbone Cancer Clinic calculated and multiplied by two to represent round trip estimate of number of miles saved by conducting telemedicine visit. This number= 15,511.8 miles for sample of 192 patients. Using EPA’s estimate of 4.03 x10-4 metric tons of CO2 emissions/mile driven in an average passenger vehicle | Patient travel [metric tons of CO2eq emissions: 3] | 6.25 metric tons of CO2 emissions prevented by patients during 4wk study period | NR | NCD | TH (N) |  |  |  |  | NCD |  |  | PSn, Cl.E, PS |
| **Gastroenterology** | King 2023: UK [GE outpatient appointments 11 NHS Trusts across the South  East UK analysed (5 tertiary centres and 6 non-tertiary centres)](King et al., 2023) | Calculate true reduction in CE resulting from transition to virtual consultations during global pandemic and assess safety compared with F2F consultations | Observational: Retrospective cross-sectional: C1 Telehealth vs C2 F2F [2140, F2F 1081 (only 756 analysed for CE outcome), Virtual: 1059 (only 1055 analysed CE data)] | NR: Total 2140: F2F Median age 53 (IQR 39-67,) 59.8% f. Centre: Tertiary 486(45%), non-tertiary 595 (55%). Virtual Median age 52 (IQR 37-67), 57% f. Centre: Tertiary 474 (44.76%), non-Tertiary 585 (55.24%) | NR | Distance travelled: estimated using shortest route from patient's home to hospital, assuming return trip. Carbon emissions from cars and taxis based on national average (146.5g/km), while motorbikes assumed to emit 116.7g/km. Public transport emissions not included as not impacted by patient attendance. For VC, emissions from infrastructure and VC services considered, with telephone-only clinics assuming higher emissions from mobile phone calls. Consultation durations adjusted to 20mins to minimize underestimation of emissions for group 2 appointments | Patient travel, software [kg CO2eq: 2 and 3] | Overall reduction of 1159.92kg CO2eq (99.37%; p=0.0001). No sig. difference in kg CO2eq between non-tertiary and tertiary sites overall (group 1: p=0.62, group 2: p=0.95) or when adjusting for no. appointments (group 1: p=0.45, group 2: p=0.89) | NR |  |  |  |  |  | C1<C2(S) | C1<>C2(S)j |  |  |  |
| **Gastroenterology** | Sillcox 2023a: USA [Bariatric surgery clinic](Sillcox et al., 2023a) | Hypothesized telemedicine would  decrease carbon emissions, improve patient compliance with appointments, and decrease overall preoperative evaluation time to surgery | Observ.: before and after C1 Telehealth vs C2 F2F [106, 51 F2F, 55 TH] | Bariatric surgery: F2F: Average age 44.5(range 25.8-66 years). 18 underwent RYGB, 42 (82.3%) F. Telemedicine: Average age 46(range 28-68 years), 32 (58.2%) underwent FYGB, 49(89%)F | Preop. visits with dietician, mental health provider, or bariatric surgery provider were classified as in-person or telemedicine. Telemedicine visits: clinic appointments completed remotely using AV conferencing. Each of our patients seen in-clinic at least once by surgeon preop. | Estimated CO2 emissions for each visit determined using EPA) formula [15] of 404g CO2/mile. Assumed patients drove to clinic from primary address. GHG emissions from TH use assumed to be small compared to emissions from vehicle travel and main focus was on estimated GHG emissions reduction due to personal vehicular travel differences | Patient travel [kg CO2: 3] | In-person visits:145 patient travel distances recorded-median [IQR]  29.5 [13.7, 85.1] miles resulting in 38.22–39.61 kgCO2-eq emitted. For telemedicine visits, mean (SD) visit time: 40.6 (17.1) min. Telemedicine GHG emissions ranged from 2.26 to 2.99 kgCO2-eq depending on device used. In-person visit=25X more GHG emissions vs telemedicine visit (p<0.001) | NR |  | C1>C2 (S) |  |  | evaluation to OR/surgery >< (S) | C1<>C2(S) | >< (N) |  |  |  |
| >< = no difference/ no detrimental effect, C1 > C2 = Analysis favoured Comparator 1 over C2, C1<> C2 = both comparator favoured in some scenario. a=263 scans. b=of 389 eligible, c=Service provided an immediate saving to commissioners of £6060, but this excludes the blood sampling costs in primary care. Generating a definitive estimate of cost saving for the tele-clinic project has proved challenging due to the way that activity and costs are recorded in secondary care. Isolating the total specific costs for a face-to-face clinic versus a teleclinic not possible d=of 51 consultations, e=Based on treatment/appointment duration, f=52 provided patient experience feedback, g=Threshold at which tele-ENT became cheaper than travel was workload of 35 patients/yr. Actual workload during pilot study=29 patient/yr, h=Of 329 approached, i=Numbers for primary site of disease don't add up for 100 surveyed, j=For 90 day admission/mortality rate. Favours F2F for no.blood test requests after appointment. AC=Acceptability, ACY=Acceptability, ACS=N.new patients accessing/Accessibility, AD=Appointment Duration, AE=Adverse Events/patient safety, APP=Advanced Practice Providers, AT=Attendance, AV=Audiovisual, BMI=Blood Mass Index BP=Blood Pressure, BTC=Blood Tests ordered Correctly/Available, C1=Comparator 1, C2=Comparator 2, CE=Carbon Emissions, CKD=Chronic Kidney Disease, Cl.E.=Clinical Effectiveness, CNS=Central Nervous System, CO2=Carbon Dioxide, CS=Clinician Satisfaction, CT=Can’t Tell, DD=Discharge Destination, DSL=Digital Subscriber Line, EHR=Electronic Health Record, ENT=Ear, Nose and Throat, EPA=Environmental Protection Agency, ESWL=Extracorporeal Shockwave Lithotripsy, F2F=Face-to-Face, GHG=Greenhouse Gas, GI=Gastrointestinal, GP=General Practitioner, HF=Heart Failure, HR=Hazard Ratio, HR=Heart Rate, IQR=Interquartile Range, ISU=Inappropriate Service Use, K=Potassium, LVEF=Left Ventricular Ejection Fraction, MDT=Multi-disciplinary Team, N=Number, N=Narrative synthesis, NA=Not Applicable, NCD=No Comparative Data, NP=Nurse Practitioner, NR=Not Reported, NS=Not specified, O+T=Orthopaedics and Trauma, PH=Patient Health, PSa=Patient Safety, PF=Physical Functioning, PPL=Patient Productivity Loss, PU=Patient Understanding, PCN=Percutaneous Nephrolithotomy, PDSA=Plan Do Study Act, PS=Patient Safety, PSn=Patient Satisfaction, SBP=Systolic BP, S=Statistical analysis, SC=Service/staff costs, SP=Service Profit, TC=Telephone Call, T2DM=Type 2 Diabetes Mellitus, TH=Telehealth, TR=Time to Referral, TS=Time to Surgery, UA=Urgent Admissions, URS=Ureteroscopy, USD=US Dollars, VC=Video/Virtual Consultations, VDSL=Very High Speed Digital Subscriber Line, VHA=Veteran’s Health Administration. 1=Department of Industry, Innovation and Science. PSMA Geocoded National Address File (G-NAF), https://data. gov.au/dataset/19432f89-dc3a-4ef3-b943-5326ef1dbecc 2=United States Environmental Protection Agency. Greenhouse Gas Emissions from a Typical Passenger Vehicle, https://www.epa.gov/greenvehicles/greenhousegas-emissions-typical-passenger-vehicle (2018, accessed June 2018). 3= UK Government. Greenhouse gas reporting: conversion factors 2020: condensed set (for most users). (2020). Accessed: November 2, 2022: https://www.gov.uk/government/publications/greenhouse-gas-reportingconversion-factors-2020 4=Curtis A, Parwaiz H, Winkworth C, et al.: Remote clinics during coronavirus disease 2019: lessons for a sustainable future. Cureus. 2021, 13:e14114. 10.7759/cureus.14114, 5=Manchester University NHS Foundation Trust. Code green: delivering net zero carbon at MFT . (2022). Accessed: Novemeber 2, 2022: https://mft.nhs.uk/app/uploads/2022/02/MFT-Green-Plan_V1.0.pdf .6=Driver and Vehicle Licensing Authority. Vehicle Licensing Statistics: 2013. London: Department for Transport, 2014 7= Kmietowicz Z. NHS hits target on reducing carbon emissions. BMJ 2016; 352: i587 8=Trees for the Future. Carbon tree plantation calculator, 2018. Available at: www.trees.org. Accessed March 2019. 9= Waddingham P. Environmental and Social Sustainability in NHS Innovation toolkit, 2021, Yorkshire and Humber Health Sciences Network. 10=Privately owned vehicle mileage reimbursement rates. Available at: http:// www.gsa.gov/portal/content/100715. Accessed March 9, 2015. 11=Passenger vehicles per year. Available at: http://www.epa.gov/cleanenergy/ energy-resources/refs.html. Accessed May 13, 2015. 12=Greenhouse Gas Emissions From a Typical Passenger Vehicle. 2018. https://www.epa.gov/greenvehicles/greenhouse-gas-emissions-typical-passenger-vehicle. 13= International Energy Agency. Fuel Economy in Major Car Markets: Technology and Policy Drivers 2005–2017; International Energy Agency: Paris, France, 2019 14=Department for Environment, Food and Rural Affairs. Act on CO2 Calculator: Public Trial Version. London: DEFRA, 2007. 15=US Environmental Protection Agency: Greenhouse gas emissions from a typical passenger vehicle. Office of Transportation and Air Quality 2018; https:// nepis.epa.gov/Exe/ZyPDF.cgi?Dockey5P100U8YT.pdf. | | | | | | | | | | | | | | | | | | | |

***Overview of non-LCA Studies - Decentralised care***

| **Speciality** | **Author, date: Country [Setting]** | **Aim** | **Study design: Intervention vs Comparator [Participants N]** | **Specific health condition: Patient features** | **Intervention description** | **CE calculation methods** | **Boundary of system evaluated [Unit: CE Scope]** | **Carbon Emission findings** | **PROGRESS-PLUS** | **Patient travel/ Distance to point of care** | **Travel related sustainability** | **Service costs saved** |
| --- | --- | --- | --- | --- | --- | --- | --- | --- | --- | --- | --- | --- |
| **Renal** | Asghari 2020: Iran/ France [Renal home health service](Asghari & Al-e-Hashem, 2020) | To improve classic pickup and delivery models, make them more useful for decision-makers to enhance performance of sharing operations while serving customers with timely home health services and provides the individuals a compact source of income | Modelling: C1 - care equipment delivery vs C2 - NA [13] | Renal failure: Patient's receiving HHM | System objective: determine optimal configuration of routes, vehicle types, and delivery sequence/pickups to satisfy patients' needs while minimizing total costs of item sharing system and reducing carbon emissions from vehicles. System supplies Home Health Monitoring (HHM) devices either from central depot or from individual owners. In sharing economy model, individuals who own HHM devices can participate in home health care system by sharing their devices through company's fleet, earning income. After delivering portable HHM devices to patients, they are collected, disinfected, and reallocated other customers. The Systematic Depot Pickup Problem is comprehensive model where company aims to serve kidney patients with limited set of portable haemodialysis machines using fleet of vehicles. Patients' requests, time windows, and individual HHM pickup time windows and max. rental time received in advance. Key differences between depot and individuals include depot being compulsory starting point for fleet tour, while individuals are not necessarily starting point. Picking up HHMs from individuals incurs renting costs based on rental period, unlike depot.  Patients typically need HHMs for a few hours (≤2 h) and used HHMs picked up by vehicles can be prepared for the next use after necessary disinfection and safety checks. Distribution involves mixed sequence of pickup-delivery activities, where vehicles can visit nodes multiple times | Optimization model aims to minimize company's total loss by reducing transportation costs, including fuel consumption and penalty costs, while minimizing total carbon emissions from vehicles. Model utilizes linear mathematical formulation solved by fuzzy aggregated method and meta-heuristic approach called self-learning NSGA-II for medium- and large-sized problems. The self-learning aspect adjusts probabilities based on changes in fitness function value. Sensitivity analysis compares different approaches. For detailed assumptions/sets, see paper | Patient travel [CO2 per litre of fuel or km travelled, 3] | Sharing policy: 25% saving in total cost, and 21% drop in total carbon emissions. Effectiveness and applicability of proposed model demonstrated by computational results on real case. Tests confirm economic and environmental benefit of scarce delivery-pickup platform significantly profits from economies of sharing in both solution techniques | NA |  |  | C1>C2 |
| **Radiology** | Bond 2009: UK [Norfolk and Norwich University Hospital NHS Trust, 2xhospitals& mobile units](Bond et al., 2009) | Compare distances travelled by patients attending mobile breast screening clinics compared to distance travel if screening services were centralized | Observ. Retro-spective data-base review: C1 Mobile clinic vs C2 Central-ized care (hospital) [60675: 21415 hospital; 39260 mobile clinic, (valid postcodes obtained for 99.5%=60372))] | Breast cancer screening: NR | Breast screening service provided by the Norfolk and Norwich University Hospital NHS Trust. Breast screening in central Norfolk provided at 2Xxhospital sites in Norwich and 20 dispersed locations by mobile units | Estimated carbon reduction benefits in terms of patients’ journeys to existing services vs travel if no mobile services were provided. Anonymised records of attendances for breast screening over 2004–2006 examined (contained person’s postcode, clinic attended, appointment date). Postcodes of 2xhospitals and locations of each mobile clinic obtained. Home address/ clinic postcodes converted into Ordnance Survey grid references and straight-line distance between home address and clinic attended and straight-line distance between each home address and closest of 2 hospitals calculated. Straight line distances increased by 20% account for approx. ratio of driving distances along road network vs straight line distances.^1,2^ 2 distance estimates compared to identify savings in travel provided by mobile clinics. Carbon implications: assumed patients drove to appointment in cars with average emission of 167.2 g/km CO2.^3^ (equivalent to emissions 2008 1.8 litre Ford Focus.^4^ This figure must be offset by CO2 emitted through transport and servicing of mobile breast screening units. Range of distances driven use 1695–1977 ltrs diesel, converted to kg CO2 using factor developed by the US EPA of 2664.2g/itre of diesel.^5^ | Patient travel, travel+ servicing of mobile breast screening unit [kg CO2, tonnes: 1,3] | Return journey distance: savings of 1,429,908km through use of mobile screening clinics=239 tonnes of CO2 over 3yrs.CO2 emitted through transport/servicing of mobile breast screening units: Range of distances driven use 1695–1977ltrs diesel: 4516–5267kg CO2. Thus, in 1 year, use of mobile screening units saves approx. 75 tonnes CO2 | NR | C1>C22 (N) |  |  |
| **Oncology** | Forner 2021: Canada [Head and neck oncology clinic, Cancer Treatment Centre](Forner et al., 2021) | Estimate the carbon footprint savings associated with head and neck surgery outreach clinic | Cross-section-al survey: C1 Outreach clinic vs C2 Regional cancer centre) [118] | Head and neck cancer: Mean age 64.8yrs (SD: 13.2). Gender (N, %): Male: 76 (67.3), Female: 37 (32.7), Income Quintile (N, %): 1: (Lowest): 25 (23.4), 2: 38 (35.1), 3: 25 (25.2), 4: 13 (12.5), 5 (Highest): 3 (2.8), Missing: 2 (1.9) (Income Quintile n=107 = number of people used for carbon footprint analysis. Missing value due to inability to perform geocoding) | Regionalized advanced head and neck surgical oncology service, Outreach clinic: approx. 325 km from regional centre. Surgical outreach clinic held by attending surgeon from regional centre for new consultations and ongoing oncology surveillance, allowing patients living in province of PEI to access aspects of care intra-provincially. Pathology treated includes upper aerodigestive tract, thyroid, salivary gland, and cutaneous malignancies of head/neck. Only surgeon travels from regional centre to outreach clinic as all other necessary staff (e.g. nursing/ administrative) local | NRC 1995-2020 Fuel consumption rate datasets for motorized vehicles accessed.12 Datasets contain make, model & year of vehicles with their associated fuel consumption rates (L/100 km). Rates generated using combination of data from vehicle manufacturers that incorporate standard laboratory testing and procedures to estimate fuel consumption rates of models. 13 The product of combined fuel consumption rate (L·100 km− 1) of each vehicle and amount of CO2 generated per ltre fuel (2300 g·L− 1) yields CO2 generated/km (g·km− 1) of travel; these values used to calculate carbon footprint as function of distance travelled. Two additional variables used to calculate specific CE yields but were unavailable in this study (engine size and number of cylinders). To accommodate this uncertainty, both lowest and highest available CE for each make, model, and year calculated and ranges provided. Postal code of each participant and surgical outreach clinic entered into Google Maps to calculate distance travelled Return trip distances travelled by each patient used to calculate/compare total vehicle CO2 emissions (g) as product of distance (km) and CE efficiency (g·km− 1). Average total annual CE savings: multiplying average CE difference/ person by expected no. patients/3-month period (approx. 100 patients) and multiplying by four | Patient travel, surgeon travel [CO2 emissions (grams) generated by their vehicles as a product of distance (kilometres) and CE efficiency (g·km-1): 3] | Median distance participants homes to surgery outreach clinic: 29.0 km (IQR 6.0–51.9;). Median distance to regional centre: 327.0 km (IQR 309.0- –337.0)=difference of 317.5 km (IQR 250.2–325.6). CE efficiency vehicles: mean 199.6 g·km− 1 (SD: 43.4) to 243.6 g·km− 1 (SD: 61.6). Median observed low estimate of CE: 10,411.2 g (IQR: 2267.2–21,254.4) vs expected estimate: 130,082.0 g (IQR: 107,724.0–149,960.0)=mean CE difference of 117,495.4 g (SD: 29,040.0). High estimate saved CE: 143,570.9 g (SD: 40,236.0). Extrapolating three-month period to an annual basis: CE savings of approx. 46,998,160 g. Total distance travelled by attending surgeon: 330.0km. CE efficiency: 211 g·km− 1, yielding carbon footprint of 69,630g/clinic. Across time of study, 3 clinics held= 208,890 g carbon emitted | NR |  | NCD |  |
| 1=Williams AP, Schwartz WB, Newhouse JP, Bennett BW. How many miles to the doctor? New Engl J Med 1983;331: 958–63 2=Transport Direct .CO2 information. 2008. See http://www. transportdirect.info/Web2/staticnoprint.aspx?id=_web2_ help_helpcarbon (last checked 30 April 2008) 3=What Green Car. 2008. See http://www.whatgreencar.com/ news-item.php?Record-low-for-new-car-CO2-emissions (last checked 29 April 2008) 4=Vehicle Certification Agency. VED Calculator. 2008. See http://www.vcacarfueldata.org.uk/search/vehicleDetails. asp?id=17908 (last checked 29 April 2008) 14 5=US Environmental Protection Agency. Average Carbon Dioxide Emissions Resulting from Gasoline and Diesel Fuel. Report No. EPA420-F-05-001. Washington, DC: EPA, 2005. A&E=Accident and Emergency, CF=Conversion Factor, CE=Carbon Emissions, CO2=Carbon Dioxide, EPA=Environmental Protection Agency, DEFRA=Department of Food and Rural Affairs, GH=GH, Interquartile Range, LSOA=Lower Super Output Area, MI=MI, NHS=National Health Service, N=Number, NCD=No Comparative Data, NR=Not Reported, NRC=Natural Resources Canada, Ppci=Primary percutaneous coronary intervention, SD=Standard Deviation, STEMI= ST elevation MI, UK=United Kingdom | | | | | | | | | | | |  |

## 8 Setting – overview of non-LCA studies

| **Speciality** | **Author date: Country [Setting]** | **Aim** | **Study design: Intervention vs Comparator [Participants N]** | **Specific health condition /procedure: Patient features** | **Intervention description** | **Carbon emission calculation methods** | **Boundary of system evaluated [Unit: CE Scope]** | **Carbon emission findings** | **PROGRESS-PLUS** | **Patient Satisfaction** | **Patient Clinical outcome** | **Clinician Satisfaction** | **Service products saved** | **Savings in service product purchased** | | **Service burden** | | **Durability** | | **Service costs saved** | | **Service energy savings** | | **Service waste saved** | | **Social sustainability** | |
| --- | --- | --- | --- | --- | --- | --- | --- | --- | --- | --- | --- | --- | --- | --- | --- | --- | --- | --- | --- | --- | --- | --- | --- | --- | --- | --- | --- |
| **Gastroenterology** | Betts 2022: UK [Endoscopy Unit: Royal Cornwall Hospital](Betts, 2022) | 1. Organize the unit and empower staff to recycle all sterile water bottles that are used on the unit daily 2. To change the CLO test reporting system to reduce waste and low value use of admin staff time | Experimental: Before and After; C1 - Waste management a: Sterile water bottle recycling b: Electronic CLO testing C2: Usual care [NA;NA'NA] | Endoscopy: Patients undergoing endoscopy | Waste management: We set up a recycling bin, ensuring there was one clear collection point for bottles each day. We discussed the change at morning safety huddles for one week, and laminated signs for all procedure rooms to remind staff and labelled the recycling bin point. We converted the electronic results already on our systems to a word document that could be emailed to GP practices | Tracked daily bottle recycling rates over 2wk period and projected annual savings. Carbon emissions from waste disposal were calculated using data from Rizan et al. CLO test usage over 3 months was analysed alongside consumable costs, including paper and postage. Paper emissions were determined using a weight-based approach, while postage emissions were estimated from financial data. Email substitution emissions were derived from overall paper savings | Water bottles: Waste disposal, CLO test results: use+ reuse [kg CO2eq / year: 1] | Combined, the projects will save 921.44kg CO2eq and £1,558.72, equivalent to driving 2,653.9 miles in an average sized car. Water bottle recycling saving: 362.548kg CO2eq / year, Electronic CLO testing; 558.89 kg CO2eq / year | NR |  | C1>C2 [N] |  |  |  | |  | |  | | C1>C2 | |  | | C1>C2 | | C1>C2 [N] | |
| **Gastroenterology** | Cunha Neves 2023: Portugal [Portimão endoscopy unit of Algarve University Hospital Centre](Neves et al., 2022) | To assess and compare the waste carbon footprint and waste processing expenses induced by endoscopic procedures (upper endoscopy and diagnostic colonoscopy | Experimental: Before and After; C1 - Waste management  C2: Before intervention; [535:NR:NR] | Patients undergoing endoscopy. Before - 185 (85 upper endoscopies and 100 colonoscopies), 178 in 1 month after intervention (84 upper endoscopies and 94 colonoscopies) 4 months after: 172 (75 upper endoscopies and 97 colonoscopies) | 1-week intervention was held. Entire endoscopy unit team (medical, nursing, and auxiliary staff) involved. Presentation of retrieved data from study’s first stage and two seminars regarding waste handling, segregation, and disposal in endoscopy units. Additionally, recycling bins acquired at cost of approximately €60, labelled and placed within endoscopy rooms, and landfill and RMW bins were relocated to avoid landfill and RMW systematic misclassification | CO2eq used as measurement unit to calculate waste carbon footprint. Equivalence of 1kg of landfill waste to 1kgCO2eq and 1kg of RMW to 3 kgCO2eq applied | Waste disposal [kgCO2eq: 1] | Preintervention versus 1 month after intervention - An overall reduction of the waste carbon footprint of 31.6% (138.8 kg CO2eq) was obtained (109.7 kg CO2eq vs 74.9 kg CO2eq, p=0.018), corresponding to a waste carbon footprint’s yearly reduction of 1665.6 kg CO2eq (figure 4). One month after intervention versus 4 months after intervention. Mean waste carbon footprint (74.9 kg CO2eq vs 71.7 kg CO2eq, p=0.841) | NR |  |  | C1>C2 [N] |  |  |  | |  | | C1>C2 [S] | |  | | C1>C2 | |  | |  |
| **Gastroenterology** | Owens 2023: UK [Endoscopy unit Swansea Bay University Health Board](Owens, 2023) | 1. To reduce printing and paper use in the Endoscopy department by transitioning to electronic ways of working; 2. To redirect Contrast waste from sharps (incineration) disposal to be recycled | Modelling; C1 - Multiple: Setting; C2 - Usual care [NA] | Endoscopy: Patients undergoing endoscopy | 1a Paper reduction: - Proposed giving patients links to online leaflets instead of printing information. Considered transitioning to electronic endoscopy reports, Patient follow-up and care pathways were ensured. - Patients notes: Electronic reporting system uploads to the WCP. Endoscopy images made available on WCP. Histology and referring consultant: Encouraged registration for WCP alerts instead of printing reports. Patient and GP: Encouraged registration for digital access to reports and results, trialling digital alerts for practices unable to access WCP. - utilizing confidential waste disposal for printed reports with errors., Introduced electronic submission of post-procedure questionnaires. 1b Established a process for recycling contrast by sending it back to the supplier in recycling pots instead of disposing of it in sharps bins | An emissions factor for one A4 piece of recycled paper (0.003 kg CO2eq) was provided by our paper supplier, Steinbeis. To calculate savings from ink, we used an emissions factor based on pounds spent from the Small World Consulting Database of 0.392 per pound spend, provided by CSH (this database is not publicly available). The CO2eq for one piece of paper printed with double-sided ink is 0.0284 kgCO2eq. For patient questionnaires, we assumed that it takes patients 5 minutes for a patient to fill out questionnaire on an iPad to calculate the energy consumption of this, using the emissions factor for energy from the Government Database | Waste prevention+ disposal [ kg CO2eq: 1] | i) Patient information leaflet - Saving: 142.5 double sided pages per week = 4.047 kg CO2eq ii) Endoscopy reports - Saving: 450 double sided pages printed per day = 12.78 kg CO2eq - Saving per week: 63.9 kg CO2eq iii) Patient questionnaires 360 double sided pages printed = 10.224 kg CO2 - 360 questionnaires completed on iPad = 0.25 kg CO2eq - Saving per day = 9.974 kg CO2- Saving per week = 49.87 kg CO2eq Total reduction per week: 117.8 kg CO2eq. Projected across a year across the three units, we could save 6,126.48 kg CO2eq per year (2,042.16 kg CO2eq per unit). 1b) Recycling of contrast. We estimate a reduction of 19.5kg / year (0.0195 tonnes / year). This equates to a saving of 21 kg CO2eq / year. Our project combined will save 6,147.48 kg CO2eq per year, equivalent to driving 17,705.9 miles in an average car | NR | C1>C2 [N] |  |  |  |  |  | |  | | C1>C2 | |  | |  | |  | | |
| **Gastroenterology** | Yong 2022:UK [Three hospital sites of the Imperial College Healthcare NHS trust, London (St Mary’s, Charing Cross and Hammersmith Hospitals)](Yong et al., 2022) | Determine whether combining multiple small colorectal polyps within a single specimen pot can reduce carbon footprint, without an associated  deleterious clinical impact | Observ.: retrospective  C1: Single pot vs C2: multiple pots [2502 procedures performed where 5125 polyps removed] | Patients receiving lower gastrointestinal endoscopy- polyps removal. 610 procedures (1281 polyps) performed as part of the Bowel Cancer Screening Programme (652 (1456 polyps) as a part of surveillance, while 1240 (2388 polyps) were for the investigation of GI symptoms | Multiple pots - Sending colorectal polyps resected during endoscopy as separate samples. Single pots - We determined that a number of pot usage if all polyps less than 10mm were placed within a single colonic segment (rectum, sigmoid, descending, splenic flexure, transverse, ascending and caecum), hemicolon (right hemicolon: caecum to ascending colon; left hemicolon: hepatic flexure to sigmoid and rectal) or throughout the colon | Estimates of the carbon footprint were based on previous work in this area, which described the processing of a single pot with an emission of 0.28 kgCO2eq.5 Our local pathway from tissue processing to production of histology report was in accordance with the Royal College of Pathology Tissue Pathway. Statistical analysis was performed by using IBM SPSS Statistics for Windows, V.28.0 (IBM). A p<0.05 was considered statistically significant. Descriptive statistics were used to report the data. The statistical significance in reduction in carbon footprint by putting all small polyps in whole colon was analysed with Z test | Waste prevention  [kgCO2eq:1] | Reduction in pot usage would have resulted in a reduction in carbon footprint to 572 kgCO2eq, 490 kgCO2eq and 289 kgCO2eq, respectively. The reduction in carbon footprint by putting all small polyps in a pot for the whole colon, in comparison with one pot per hemicolon was statistically significant (p<0.00001), as was the comparison between placing in segmental distribution versus hemicolon distribution  **PROGRESS-PLUS:**  ***Age:*** Mean age of patients with detected polyp: 63.9yrs (range 24-96yrs)  Other outcome: Fidelity to clinical process: C1 <> C2 |  |  |  |  |  |  |  | |  | |  | |  | | C1>C2 | |  | |  |
| **Renal** | Bird 2022:UK [Renal transplant department](Bird, 2022) | Measure environmental, social & financial benefits of new postal system  compared with old postal system | Modelling C1: Waste management - Changing composition of patient blood test kit C2: Usual care [30:30:NR] | Renal transplant or dialysis: Patients awaiting renal transplant or simultaneous kidney and pancreas transplant | Use of lightweight, recyclable plastic pouch with pre-paid postage labels, eliminating the trip to the post office (in favour of closest post box) to convenience of patients | A process-based carbon foot printing analysis was used to estimate the carbon footprint of both kits (blood tests were the same in both kits and therefore excluded from analysis). Data on type of material taken from product specification sheets, and each material weighed. Assumed both kits disposed of in domestic waste. Carbon emission factors for waste disposal taken from recent study from Rizan (2021) looking at carbon footprint of waste streams in a UK hospital. Financial data was used to estimate the carbon emissions associated with postage. Looked only at the emissions associated with sending kits from hospital to patient only | Use+ reuse+ transport to patient +waste disposal [kg CO2eq: 1] | The total emissions per test (kit + postage) were reduced by 5.495 kgCO2eq. Extrapolated across a year with average of 30 low clearance/pre-dialysis clinic patients requiring 3 monthly blood tests, annual total of 222 blood tests, this is a saving of 1219.9 kgCO2eq | NR | C1>C2 [N] | >< | C1>C2 [N] |  |  |  | |  | | C1>C2 | |  | |  | |  | |  |
| **Renal** | Milne 2010:UK [Maidstone dialysis unit](Milne, 2010) | Investigated the potential costs and benefits of retrofitting heat exchangers to their existing Braun Dialog+ haemodialysis machines | Modelling C1: Heat exchangers; C2: Conventional care [NA] | Patients receiving haemodialysis treatment | Heat is recaptured from the dialysis effluent (‘used’ dialysate) and transferred to the incoming dialysate, warming it up before it enters the heater and thereby saving energy and reducing the environmental impact of a haemodialysis treatment. The renal technicians at the Maidstone dialysis unit decided to investigate the possibility of retrofitting heat exchangers to their existing machines. Retro-fit heat exchanger kits for Braun Dialog+ machines can be fitted by most renal technicians in less than half an hour | Assuming each machine is used twice daily, six days a week for 52 weeks of the year, an annual power saving of 536.64 kWh per machine (2 * 6 * 52 * 0.86) is predicted | Use+ reuse [tonnes CO2eq: 2] | Applying a conversion factor of 0.50748 kg CO2 equivalents per kWh, this in turn equates to an annual saving of 272.33 kg (0.272 Tonnes) of CO2 equivalents per machine per year. For the 83 machines across the Kent and Canterbury renal service, this equates to an annual power saving of 44,541 kWh and an annual carbon saving of 22.6 Tonnes of CO2 equivalents | NR |  |  |  |  |  |  | |  | | C1>C2 | | C1>C2 | |  | |  | |  |
| **Renal** | Milne 2023:UK [East Kent Renal Service](Milne, 2023) | Investigate the possibility of retro-fitting heat exchangers to their existing machines | Modelling C1: Heat exchangers; C2: Conventional care [NA] | Patients receiving haemodialysis treatment | Retrofit of heat exchangers to 52 machines across East Kent renal service | Assuming each machine is used twice daily, six days a week for 52 weeks of the year, an annual power saving of 536.64 kWh per machine (2 * 6 * 52 * 0.86) is predicted | Use+ reuse [tonnes CO2eq: 2] | Applying a conversion factor of 0.50748 kg CO2 equivalents per kWh, this in turn equates to an annual saving of 316.5 kg (0.3165 Tonnes) of CO2 equivalents per machine per year. For the 52 machines retrofitted across the Kent and Canterbury renal service, this equates to an annual power saving of 27,905 kWh and an annual carbon saving of 16.46 tonnes of CO2 equivalents. Although the manufacture of heat exchangers incurs a carbon cost in itself, this is estimated to amount to less than one percent of the carbon savings derived from the improved energy efficiency in the first year of use alone | NR |  |  |  |  |  |  | |  | | **C1>C2** | | **C1>C2** | |  | |  | |  |
| **Radiology** | Buttner 2021: Germany; [University department (three major university campuses and several  smaller hospitals)](Buttner et al., 2021) | Investigate if switching off workstations after core working hours can lower energy consumption considering both ecological and economical aspects | Cross-sectional/ modelling C1: Energy savings - a: Switching off workstations outside core working hours; b: Reduced working workstations outside normal working hrs C2: Usual care [NA;NA'NA] | NR:NA | The energy consumption of reading workstations was measured, comprising a desktop computer, two medical-grade diagnostic monitors, and a third monitor for the radiology information system. Various theoretical work scenarios were extrapolated based on measurements and shift planning. C1a: Workstations being turned on during the 9 -h core working time 5 days a week between 7.30 a.m. and 4:30 p.m. and then turned off..., users were briefed to switch off the workstations after core working hours. In addition, reminders were attached to the workstations to shut them down at the end of the working day. C1b: All radiology workstations are switched on during 9h core working hours (7:30 a.m. to 4:30 p.m.). After core working time, most of the workstations are turned off, only 15 workstations remain on for late shift (4:30 p.m. to 10 p.m.), 6 workstations for night shift (10 p.m. to 7:30 a.m.), and an additional 6 workstations for 24 -h shifts at weekends and on national holidays | The reading workstations, comprising a desktop computer, two medical-grade diagnostic monitors and a third monitor for the radiology information system (RIS), had their power consumption measured in watts. Extrapolations for various work scenarios were made based on these measurements and shift planning. Ammeters were installed at three workstations for continuous power consumption measurement over 6 months, with users subsequently briefed to switch off workstations after core hours and reminders attached. The measured energy consumption was extrapolated to calculate total annual consumption for all 227 workstations. The cost difference and CO2 emissions were also estimated based on current electricity prices and the country's energy mix. Startup and standby mode times were measured, and personnel costs resulting from manual restart waiting time were calculated based on hospital salary tables | Use+ Reuse [Tonnes of CO2: 2] | For C2, annual emission was 123.0 tons of CO2 emissions. C1a would reduce kWh consumption to 31.8 tons of CO2. C1b would reduce emissions to 35.3 tons of CO2), a reduction of 71.3% compared to scenario 1. This scenario is considered to be ideal and feasible for radiology departments. After briefing users to switch off the workstations after work, extrapolated consumption would be 102,871.7 kWh/year (see Fig. 2), resulting in a saving of 2,100.7 USD and 3.2 tons of CO2. Potential saving in the ideal but realistic situation (C1b) would be an additional 35,970.69 kWh (see Fig. 2), meaning a further reduction of 35.0% or 19.0 tons of CO2. Compared to our initial situation, in total, the power consumption of our workstations could be reduced by 38.6%, accordingly 22.2 tons of CO2 emissions could be avoided | NR |  |  |  |  |  |  | |  | | C1>C2 | | C1>C2 | |  | |  | |  |
| **Radiology** | Heye 2023: Switzerland [Tertiary care radiology department](Heye et al., 2023) | Identify idle energy consuming imaging modalities and electronic devices in a hospital setting to reduce energy consumption and CO2 emissions | Prospective cohort C1: Energy saving C2: Before intervention; [NA;NA'NA] | NA | From Oct - Dec2022, monitored 60 medical imaging systems, including CT, MRI, PET/CT, radiography, angiography, and ultrasound units, along with 80 PACS workstations, 165 personal computers, six smart monitors, and 53 printers. Using a self-developed Python script, the network status of each device was automatically tracked in 15-minute intervals by pinging their respective IP addresses or DNS host names. Data on client names, network statuses, and timestamps were recorded in a database and visualized using live dashboards or business intelligence software. Live dashboards were provided to department staff, showing devices left on during off-hours and potential energy, cost, and CO2 emission savings. A workflow was established to power down devices not in use during off-hours, including nights and weekends | A self-developed Python script designed to track activity of devices by querying network status (online vs offline) at 15-minute intervals using their IP addresses or DNS host names. This script records client names, network statuses, and timestamps in a database and visualizes the data using live dashboards or business intelligence software. Energy consumption of devices measured using in-house power meters, and savings calculations based on national-specific carbon intensity of 0.128 kg CO2 equivalent per kilowatt-hour | Use+ reuse [Metric tons CO2 emissions: 3] | The realized per-year energy savings was 72 337 kWh, representing $19 531 in energy costs in 2022 ($60 937 in 2023) and 9.26 metric tons in CO2 emissions | NR |  |  |  |  |  |  | |  | | C1>C2 | | C1>C2 | |  | |  | |  |
| **Radiology** | Klein 2023: Germany [1) Energy optimized medical centre with different clinical disciplines and radiology practice, 2) Open MRI practice](Klein, 2023) | We examined ways to improve energy efficiency in radiology by using regenerative and energy-friendly technology in the construction & operation of two radiological facilities | NR: C1: Energy optimised medical centre C2: Open MRI practice/Practice for low-field MRI C3: Medical centre without regenerative technology [NA;NA'NA] | NA | C1: In 2009-2010, an energy-efficient medical center was constructed, featuring a 29.92 kWp photovoltaic system and a heat exchanger for thermal energy recovery. It housed various clinical disciplines, including a radiology practice with a 1.5 T MRI machine and a CT scanner.in 2012, a four-floor building was built to accommodate various medical services, meeting German Energy Saving Ordinance regulations. The radiology practice in this building utilized a CT scanner, ultrasound equipment, and a 1.5 T MRI machine, with heat recovery for building heating. C2: In 2019, an energy-efficient open MRI practice was established nearby. It featured a 0.35 T open MRI machine powered by a permanent magnet, along with a photovoltaic system and a 10 kWh lithium-ion battery for sustainable energy production. Additionally, a photovoltaic array was installed on the building's roof | Energy consumption and production processes were documented for all types of centers, with a focus on carbon emissions associated with these processes. Energy consumers included heating/air conditioning, radiology equipment (1.5 T MRI, 0.35 T MRI, and CT), and data processing systems, while energy producers included heat exchangers and photovoltaic systems. Electricity and gas consumption data were collected for the medical center, while energy monitoring for the open low-field MRI practice was conducted using electronic measuring equipment. Consumption values were measured for various operating states of the components, with the generation of carbon dioxide (CO2) used as a parameter for energy efficiency. Normalization to CO2 generation was applied to electricity, gas consumption, and thermal energy variables for comparative analysis | Use [kg CO2eq: 2] | Energy optimization of the medical center resulted in an annual CO2 reduction of about 54% from 153,146 to 70,631 kg/year.  See full text for more info | **NR** |  |  |  |  |  |  | |  | | **C1>C2>C3** | | **C1>C2>C3** | |  | |  | |  |
| **Radiology** | McCarthy 2014: Ireland [University teaching hospital, Dublin](McCarthy et al., 2014) | We sought to perform an energy audit of our department to identify where savings could be made. We re-audited the energy use 18 months after an educational session within the department | Before and After; C1: Energy conservation C2: Before intervention [NA;NA'NA] | NA | Results of 1 week energy audit presented at a departmental teaching session | The energy consumption throughout the department was measured using a power monitor. Electrical energy usage during overnight hours on weekdays (5pm to 8am) and throughout weekends (from Saturday 8am to Monday 8am) was calculated, totalling 6396 hours annually. In cases where connecting the monitor to a device was not feasible, technical manuals were consulted instead. Monitoring of computers and monitors left on at 6.30pm during a one-week period allowed for estimation of annual power consumption when not in active use. Air conditioner status was also observed during this time. Central air conditioning in other areas could not be measured or turned off. Additionally, air conditioning units in conference rooms were frequently left running. Technical manuals for these units were obtained, and the average power consumption was documented | Use+ reuse [metric tons of CO2 equivalent: 2] | From desktop computers left on overnight/weekends /both: estimated greenhouse emissions of 17.7 metric tons of CO2 EQ, similar to emissions from 3.7 passenger cars. From PACS reporting stations left on overnight/at weekends/both: 33.5 metric tons, CO2, equivalent to annual emissions of 7 passenger cars. Overnight operation of two air con units: 26.2 metric tons of CO2eq, similar to emissions of 5.5 passenger cars/year. Follow up audit indicated a slight increase to number of PACS workstations left on overnight | NR |  |  |  |  |  |  | |  | | NC | | NC | |  | |  | |  |
| **Radiology** | Woolen 2023; USA [Department of radiology and biomedical imaging, university of California, San-Francisco outpatient](Woolen et al., 2023) | To determine the energy, cost, and carbon savings that could be achieved through different scanner power management  strategies | Retrospective cohort C1: Energy saving C2: Normal hours [NA;NA'NA] | NA | C1a: COCIR self-regulatory initiative for the eco-design of medical imaging equipment was followed to identify the highest-impact energy modes to target for innovation (6). COCIR identified the nonproductive scanning periods as targets for MRI. The off mode was targeted for innovation. MRI unit 1 included a power-save mode. In MRI units 2–4 (i.e., the three scanners not equipped with power-save mode), the cold head compressor for individual MRI units was switched off for 30 minutes to measure the power consumption. The cold head compressor data were used to simulate the technique used with MRI unit 1 to evaluate the impact of implementing this capability on other systems. MRI Operations When Not in Use Scanner hours of operation were set in accordance with the COCIR report (6), which defined a typical outpatient day as having 12 overnight hours of non-use | To assess energy, cost, and carbon emission savings from low-power scanner modes during off hours, two standardized operational MRI models were examined. The first model involved 12 hours of non-use overnight daily, while the second model extended this to 48 hours during weekends. Power meters were installed in equipment rooms to monitor energy consumption continuously. Data collected from September 29 to November 1, 2022, and January 13–17, 2023, were analysed. Different power modes of the MRI scanners were assessed, including off, idle, prepared-to-scan, scan, and an Eco-Power mode. Nonproductive energy consumption also evaluated, with carbon emission savings calculated based on the U.S. national average carbon dioxide marginal emission rate using the AVERT tool | Use+ reuse [CO2 tonnes equivalent: 2] | Scenario 1, where MRI units 1–4 were set to the lowest power mode for 12 hrs overnight- potential annual savings of 8.7–14.9 MTCO2 eq. For switching from idle to off mode, with an additional 6.2–8.1 MTCO2 eq for switching from off to power-save mode. Scenario 2, where units were set to the lowest power mode for 12 hrs overnight on weekdays and 48 hrs on weekends, potential annual savings were 11.2–19.2 MTCO2 eq for switching from idle to off mode, with an additional 8.0–10.4 MTCO2 eq for switching from off to power-save mode. Extrapolating these results, a department with 30 MRI machines turned off for 12 hrs overnight could save annually 260.9–447.2 MTCO2 eq | NR |  |  |  |  |  | C1>C2 | |  | | C1>C2 | | C1>C2 | |  | |  | |  |
| **Multiple: orthopaedic & traumatology, ear/nose/throat surgery, ophthalmology, visceral, urology, gynaecology, neurosurgery, obstetrics, cardiology, liver, kidney, pancreatic, cardiac, radiology, gastrointestinal** | Chambrin 2023: France [4 University hospitals Lyon with surgical activity](Chambrin et al., 2023) | To assess whether implementing information campaigns was associated with a decrease in carbon footprint related to inhaled halogenated anesthetics | Retrospective cohort C1: Anaesthesia Education C2: NR [121 anesthesia providers (53 nurses, 38 anesthesiologists, 30 residents) Questionnaire: 180 anesthesia providers completed (80 nurses, 62 anesthesiologists, 38 residents)] | Extracorporeal lithotripsy, liver, kidney and heart transplants; NA | An initiative on sustainable anesthesia practices was launched in January 2018, involving regular meetings every six months at each hospital to disseminate information. Topics covered included the environmental impact of anesthetic drugs, waste reduction, recycling, and energy-saving measures. Additionally, an awareness campaign in June 2019 specifically addressed the environmental impact of hypnotic drugs, with meetings held at each hospital and information distributed via email. An online questionnaire was provided to gauge anesthesia providers' interest and intention to change practices regarding the use of halogenated anesthetics, with reminders sent between June and July 2019, and the questionnaire accessible until October 2019 | From Jan 2015-Feb 2020, data on sevoflurane, desflurane, and propofol purchases were collected from monthly product order databases at each hospital. Monthly carbon footprint estimates from desflurane& sevoflurane perioperative emissions were expressed as CDE100. An estimate of annual N2O consumption distributed to each hospital from external gas cylinders was made. The total no of procedures performed monthly under local, regional, and general anesthesia was recorded. Data were retrieved from EMR systems, primary endpoint was CDE100 in tons related to inhaled halogenated anesthetics. Interrupted time-series data were analyzed by segmented regression for carbon footprint, hypnotic purchases, and costs related to the 3 gases. seasonality-adjusted analyses performed and the number of general anesthesia uses included in the regression formula. The analysis was conducted using R 4.0.3 | Use/reuse [CO2 equivalency over 100 years CDE100 : 1] | After the establishment of sustainable anesthesia practices, the carbon footprint of sevoflurane and desflurane showed a significant decrease, with a slope changing from -0.27 to -14.16 tons/month for desflurane and a decrease in slope to -7.58 tons/month after a targeted information campaign. The median carbon footprint from perioperative desflurane decreased from 271.1 tons to 22.4 tons, and from 12.3 tons to 22.2 tons for sevoflurane. When weighted by surgical activity, the median emissions from perioperative inhaled halogenated anesthetics decreased from 66.2 kg CDE100/general anesthesia to 6.5 kg CDE100/general anesthesia | NR |  |  |  | C1<C2 for all three gases | >< for all three gases |  | |  | | C1>C2 | |  | |  | |  | |  |
|  |  |  |  |  |  |  |  |  |  |  |  |  |  |  |  | |  | |  | |  | |  | |  | | |
| **Orthopaedics** | Kodumuri 2022;2023: UK [2 district general hospitals](Kodumuri et al., 2023; Kodumuri, 2022) | Model 1 - To determine the carbon footprint of CTR, Model 2 - To construct and implement the lean and green model for the operation. The financial costs associated with the two models were also determined, we also commented on the environmental, financial and social impacts of the study | Experimental: Before and After C1 - Setting; C2 - Standard model [110: 103 intervention, 7 control] | Patients undergoing carpel tunnel surgery | The study utilized the four-step approach suggested by the Centre for Sustainable Healthcare (CSH), involving goal setting, studying the existing system, studying the lean and green model system, and measuring impact. Phase 1 evaluated the standard clinical practice of carpal tunnel release using a generic hand set of surgical instruments and formal extremity drape system over 2 months. Phase 2 introduced a lean and green model via two pilot lists, which included reducing the generic handset to a carpal tunnel release-specific set, changing to smaller drapes, and minimizing the use of single-use non-recyclable items. Phase 3 involved implementing the carpal tunnel release in the lean and green model as standard practice following review of the pilot lists from Phase 2 | A digital suspension weighing machine to measure waste generated after each CTR, documenting the mass of waste in clinical waste bags post-surgery. The carbon footprint for CTR was defined by calculating carbon emissions for each inventory item using an online calculator based on LCA from the CSH. Disposable item emissions were determined based on material type and weight. In the standard model, surgical trays and instruments were weighed, and the carbon emission factor for stainless steel adjusted for re-sterilization. This, combined with disposable item emissions, yielded the carbon footprint of a CTR. For the lean and green model, essential disposable item emissions were added to the carbon emissions of re-sterilizing a specific tray with essential instruments. The difference between the two models estimated probable CO2 emissions savings for each CTR | Disposal [kg CO2eq: 1] | Prospective evaluation of carbon footprint calculation of the lean and green model showed a reduction in carbon emissions to 6.6 kg (range 6.2–7.3) (Figure 5). This represented an 80% reduction with the carbon footprint of the sterile field reduced by 98% just by changing to smaller drapes (9.3 kg versus 0.2 kg). Smaller trays with essential instruments reduced the emissions for the instrumentation component by 66% (14.1 kg versus 4.7 kg) | NR |  |  |  |  |  | C1>C2 [N] | |  | | C1>C2 | |  | | C1>C2 | |  | |  |
| **Ophthalmology** | Vo 2023: USA [Retina clinics](Vo et al., 2023) | Analyses the feasibility, environmental impact, and cost of reusing shipping materials for intravitreal injection medications, as compared to wasting coolers and cold packs after single-use | Observational: cohort; C1: Reuse Packaging C2: Standard practice [NA;NA'NA] | NA | Shipping materials related to weekly shipments of repackaged bevacizumab for intravitreal injection were collected upon receipt in a clinic after overnight shipping from an outsourcing facility in New York via UPS. The packaging materials included 3 polystyrene coolers, 11 cold packs, and 3 card boxes, which were typically discarded. These materials were inspected and returned to the outsourcing facility via UPS ground shipping, with damaged items replaced. Temperature compliance was ensured by reviewing the cold packs upon arrival to confirm they were frozen, while the syringes of Avastin were not frozen. The study spanned 10 weeks, consisting of 10 roundtrips for the packing materials | The materials were weighed using a multifunction scale and recorded in a spreadsheet. Photographs were taken with an iPhone 11. The cost analysis utilized material and shipping costs from the outsourcing facility, considering applicable bulk discounts. Carbon dioxide equivalent (CO2eq) was estimated based on Environmental Protection Agency (EPA) data, as described previously | Transport (from raw materials stage to clinic and return of used materials to outsourcing facility), waste disposal) [kg CO2eq: 2] | Total CO2eq emissions were reduced 43% by reusing shipping materials, as compared to the standard practice of disposing containers after single use, as shown in Table 1 | NR |  |  |  |  |  |  | | C1>C2 [N]* | | C1>C2 | |  | | C1>C2 | |  | |  |
| *Cold packs shipping materials were less durable than polystyrene foam coolers, Green cell - statistically significant, [N]=supported by narrative write up (no formal statistics), C1<> C2 -both comparator favoured in some scenario, >< - no detrimental effect/ no difference, BEIS=Business, Energy & Industrial Strategy, C=Comparator, CDE100=CO2 equivalency over 100 years, CLO=Campylobacter-like organism, CO2=Carbon Dioxide, COCIR=The European Co-ordination Committee of the Radiological, Electromedical, and Healthcare Information Technology Industry, CSSU=Central sterile services unit, CT=Computed tomography, CTR=Carpal tunnel release, GHG=Greenhouse gases, DNS=Domain name system; EMR=Electronic Medical Record, GP=General practitioner, ICE=The Inventory of Carbon & Energy database, IQR=Interquartile range, MRI=Magnetic resonance imaging, N20=Nitrous oxide, NA=Not Applicable, NCD=No comparative data, NR=Not reported, OR=Operating Room, PACS=Picture archiving and communication system, PET=Positron emission tomography, RMW=Regulated medical waste, SDU=Sustainable Development Unit, UPS=United Parcel Service | | | | | | | | | | | | | | | | | | | | | | | | | | |  |

##

## 9 Product level – additional tables

***Summary other impact categories - LCAs evaluating 'Product-level' interventions (Reuseable instruments) within urology***

| **Study, Speciality: Study Design** | **Name of Interventions (C1 vs C2 etc…)** | **Other Impact Categories** | | | | | |
| --- | --- | --- | --- | --- | --- | --- | --- |
|  |  | **Mineral resource depletion** | **Ecotoxicity** | **Acidification** | **Eutrophication** | **Solid Waste produced** | **Cost** |
| Baboudjian 2022, Urology; LCA(Baboudjian et al., 2022) | C1: RU flexible cystoscopes vs C2: SU cystoscopes | Favours SU | No difference | Favours SU | No difference |  |  |
| Hogan 2022, Urology; Prospective single-centre cohort study: controlled trial^a,b^ (Hogan et al., 2022) | C1: RU vs  C2: Disposable flexible cystoscopes |  |  |  |  | Favours D |  |
| Kemble 2023, Urology; Inventory Analysis(Kemble et al., 2023) | C1: SU vs  C2: RU cystoscopes |  |  |  |  |  |  |
| Wombwell 2023, Urology; Inventory analysis(Wombwell et al., 2023) | C1: SU Ambu® aScope™ 4 Cysto System (Ambu®) vs  C2: RU Olympus CYF-VH flexible video-cystoscope |  |  |  |  |  |  |
| Davis 2018, Urology; Inventory analysis^c^(Davis et al., 2018) | C1: RU flexible ureteroscopes vs C2 2: Disposable flexible ureteroscopes |  |  |  |  |  |  |
| Green cell=Study appraised as Low risk of bias, Blue cell=Study appraised as Medium risk of bias, Orange cell=study appraised as High risk of bias. ^a^Incorporates simplistic LCA methods, ^b^Results queried by Rizan, ^c^Stated as LCA but incomplete impact assessment. C=Comparator, D=Disposable, RU=Reuseable, SU=Single Use | | | | | | | |

***Summary other impact categories - LCAs evaluating 'Product-level' interventions (Reuseable instruments) within gastroenterology***

| **Study, Name of Interventions (C1 vs C2…)** | **Findings from Other Environmental Impact Categories** | | | | | | | | | | | | | | | | | | | | | | | | |
| --- | --- | --- | --- | --- | --- | --- | --- | --- | --- | --- | --- | --- | --- | --- | --- | --- | --- | --- | --- | --- | --- | --- | --- | --- | --- |
|  | **Stratospheric ozone depletion** | **Ozone depletion** | **Smog** | **Ionising radiation** | **Ozone formation: human health** | **Ozone formation: Terrestrial ecosystems** | **Fine particulate matter** | **Mineral resource depletion** | **Ecotoxicity** | **Terrestrial ecotoxicity** | **Freshwater ecotoxicity** | **Marine water ecotoxicity** | **Acidification** | **Freshwater Eutrophication** | **Marine eutrophication** | **Land use** | **Fossil resource scarcity** | **Water consumption** | **Human carcinogenic toxicity** | **Human non-carcinogenic toxicity** | **Endpoint: Resources** | **Endpoint: Non-renewable resource use** | **Endpoint: Ecosystem quality** | **Endpoint: Human Health** | **Cost** |
| Boberg 2022, C1: Single-use trocar system vs C2: Reusable trocar system vs C3: Mixed trocar systems for laparoscopic chole-cystectomies(Boberg et al., 2022) |  |  |  |  |  |  |  |  |  |  |  |  |  |  |  |  |  |  |  |  | SU vs RU: Favours RU. SU vs Mixed: No SD |  | SU vs RU: Favours RU. SU vs Mixed: No SD | SU vs RU: Favours RU. SU vs Mixed: Favours Mixed | RU and mixed trocar systems approx. half as expensive as SU |
| Le 2022, C1: Reuseable duodenoscope vs C2: Reuseable duodenoscopes with disposable endcaps vs C3: Single-use duodenoscopes(Le et al., 2022) |  |  |  |  |  |  |  |  |  |  |  |  |  |  |  |  |  |  |  |  |  | C3 vs C1+C2: Favours C1+C2. C1 vs C2: No SD | C3 vs C1+C2: Favours C1+C2. C1 vs C2: No SD | C3 vs C1+C2: Favours C1+C2.^a^ C1 vs C2: Favours C2^b^ |  |
| Rizan 2022, C1: Single-use vs C2: Hybrid surgical instruments used for Laparoscopic cholecystectomy (laparoscopic clip appliers, laparoscopic scissors, and ports)(Rizan & Bhutta, 2022a) | H |  |  | SU | H | H | H | H |  | See SM | SU^c^ | SU^c^ | H | H | SU | H | H | H | H | H | H |  | H | For endpoint categories: combination of hybrid laparoscopic clip appliers, scissors, and ports for single laparoscopic cholecystectomy saved estimated 1.13 e−5 DALYs | H |
| Sherman 2018, C1: Reusable vs Single-use/ disposable laryngoscopes(Sherman et al., 2018) |  | RU | RU |  |  |  | RU^d^ |  | RU |  |  | RU^e^ | RU |  | RU |  |  |  | RU | RU |  |  |  |  | RU |
| Green cell=Study appraised as Low risk of bias, Blue cell=Study appraised as Medium risk of bias, Orange cell=study appraised as High risk of bias ^a^SD 13-26 times worse than 2 types of RDs in terms of environmentally mediated human health impacts (not counting direct impact from infections). If infections included, human health burden of SD close to total human health impact of RD. If assumption disposable endcap reduce infection risk of RDs by 50% realized, human health burden of RDs with disposable endcaps would then be lower than that of SDs (a factor of .75 of the SD lower bound). If infection rate of RDs decreases to 23/500,000 or .0046%, overall negative human health impact of RD will fall below lower bound health impact of an SD, ^b^RDs with disposable endcaps perform similarly to traditional RDs in all categories, with advantage of potentially reducing infections, ^c^RE: Laparoscopic scissors only, ^d^Labelled as 'Respiratory effects', ^e^HLD of reusable handle produced the fewest emissions in all impact categories except fossil fuel depletion, HLD fewest emission for Blades across all impact categories. C=Comparator, H=Hybrid, RU=Reuseable, SD=Significant Difference, SU=Single Use | | | | | | | | | | | | | | | | | | | | | | | | | |

***Summary other impact categories - LCAs evaluating 'Product-level' interventions (Reuseable instruments) within cardiac, ICU and respiratory***

| **Study, speciality: intervention comparison** | **Findings from Other Environmental Impact Categories** | | | | | | | | | | | | | | | | | | | | | | |
| --- | --- | --- | --- | --- | --- | --- | --- | --- | --- | --- | --- | --- | --- | --- | --- | --- | --- | --- | --- | --- | --- | --- | --- |
|  | **Ozone depletion** | **Smog** | **Ionising radiation** | **Ozone formation: human health** | **Fine particulate matter** | **Ecotoxicity** | **Freshwater ecotoxicity** | **Marine water ecotoxicity** | **Acidification** | **Eutrophication** | **Freshwater Eutrophication** | **Marine eutrophication** | **Eutrophication terrestrial** | **Land use** | **Fossil resource scarcity** | **Water consumption** | **Human carcinogenic toxicity** | **Human non-carcinogenic toxicity** | **Loss of scarce resources** | **Resource use: energy carriers** | **Resource use: metals and minerals** | **Respiratory inorganics (Disease incidents)** | **Cost** |
| Schulte 2021, Cardiac; C1: newly-manufactured catheter vs C2: remanufactured catheter (Schulte et al., 2021) | RM |  | RM | RM^a^ |  |  | RM |  | RM |  | Virginj | RM | RM | Virgin^b^ |  | No difference | RM | RM |  | RM | RM | RM |  |
| Sorensen 2018 , Respiratory; C1: SU flexible device for bronchoscopy vs C2: RU bronchoscope(Sørensen & Grüttner, 2018) |  |  |  |  |  |  |  |  |  |  |  |  |  |  |  |  |  |  | SU^e^ | SU^e^ |  |  |  |
| Green cell=Study appraised as Low risk of bias, Blue cell=Study appraised as Medium risk of bias, Orange cell=study appraised as High risk of bias. ^a^'Photochemical ozone formation', ^b^Primary data for virgin production missing. Due to this lower level of detail calculated environmental impacts of virgin production route tend to be underestimated vs medical remanufacturing route, ^c^Labelled as 'Respiratory effects', ^d^Disposable cuffs not cleaned over 5-day stay show slight cost advantage vs reusables. When reusable cuffs shared+cleaned after each patient encounter in the Regular Ward, additional labour and wipe costs incurred mean disposable cuffs preferable, even when dedicated disposables disinfected daily. However, if Regular Ward patients to use dedicated reusable cuffs, e.g. in ICU setting, reusables would be more favourable, ^e^Using one set of protective wear/operation and materials for cleaning and disinfection determine reusable scopes have higher values of resource consumption. Cleaning two or more reusable scopes per set of PPE makes the impacts fairly comparable. C=Comparator, RM=Remanufactured, RU=Reuseable, SU=Single Use | | | | | | | | | | | | | | | | | | | | | | | |

***Summary other impact categories - LCAs evaluating 'Product-level' interventions (Equipment composition) within urology***

| **Study, Speciality: Name of intervention comparison** | **Findings from Other Environmental Impact Categories** |
| --- | --- |
| Stripple 2008, Urology; C1: TPU catheter vs C2: PVC catheter vs C3: Polyolefin-based elastomer catheter(Stripple et al., 2008) | ***NOx and SO2 emissions:*** follow the same emissions pattern as for CO2. ***Eco-indicator 99 model - summary findings:*** Compared to TPU, new polyolefin-based elastomer shows lower environmental impact in all categories except ecotoxic emissions and extraction of minerals. Compared to PVC, polyolefin-based elastomer shows a lower impact in six of nine categories (Impact categories: Climate change, Ozone layer depletion, Carcinogenic substances, Respiratory substances (org) Respiratory substances (inorg), Ecotoxic emissions, Extraction of minerals, Extraction of fossil fuels Acidification and Eutrophication) . ***CM2 model - summary findings:*** New material shows an overall low environmental impact. Compared to TPU, polyolefin-based elastomer has a lower or equivalent environmental impact in all impact categories. Compared to PVC, its impact is lower in five out of 10 impact categories (Impact categories: Abiotic depletion ADP, Global warming GWP100, Ozone layer depletion, Eutrophication EP, Acidification AP, Photochemical oxidation POCP, Human toxicity, Terrestrial ecotoxicity, Marine aquatic ecotoxicity, Freshwater aquatic ecotoxicity). ***EPS 2000 model- summary findings:*** Results show highest environmental impact for TPU catheter, while the polyolefin-based elastomer and the PVC catheters show almost equivalent environmental impact, with a small favour towards the PVC catheter (Final scores based on weighted values) ***EPD model - summary findings:*** New material has low general environmental impact compared vs older materials+lower impact than TPU in all impact categories and lower impact than PVC in all categories except global warming, eutrophication and photochemical ozone. Increased acidifying potential for PVC attributable to HCl emissions to water caused by landfilling of PVC and higher ODP level caused by use of CFC/HCFC in PVC polymer production. TPU’s high eutrophication potential is caused by the polyurethane material’s nitrogen content. Potential environmental gain from waste energy relatively low (impact categories included: Energy resources: non-renewable (%) Energy resources (%): renewable Global warming potential (%) Eutrophication potential (%) Acidifying potential (%) Photochemical ozone POCP (%) Ozone depletion potential ODP 20 (%)) |
| Blue shaded=study appraised as Medium risk of bias. C=Comparator, GWP=Global Warming Potential, NOx=Nitrous Oxide, PVC=Polyvinyl Chloride, SOx=Sulphur Oxides | |

***Summary carbon emission findings- non-LCA studies (Equipment type)***

| **Speciality** | **Author date: Country [Setting]** | **Aim** | **Study design: Intervention vs Comparator [Participants N]** | **Specific health condition: Patient features** | **Intervention description** | **Carbon emission calculation methods** | **Boundary of system evaluated [Unit: CE Scope]** | **Carbon emission findings** | **PROGRESS-PLUS** | **Patient Clinical outcome** | **Clinician Satisfaction** | **Fidelity to clinical process** | **Saving in service products used** | **Service costs saved** | **Service energy savings** | **Service waste savings** | **Social sustainability** | |
| --- | --- | --- | --- | --- | --- | --- | --- | --- | --- | --- | --- | --- | --- | --- | --- | --- | --- | --- |
| **Trauma and Orthopaedics** | Chan 2023:UK [Orthopaedic surgery Gloucester-shire Hospitals NHS Foundation Trust](Chan, 2023) | 1) Evaluate and compare the carbon footprint of the Ecopulse compared to the Pulsvac Plus  2) Evaluate and compare the cost of Ecopulse compared to Pulsvac Plus  3) Clinical evaluation of Ecopulse by surgeons | Before and After study; Pule lavage Equipment C1: Ecopulse vs C2: Pulsvac Plus [NR] | Patients receiving total joint arthro-plasty | Ecopulse - the only commercially  available carbon neutral pulsatile lavage system on the market. The main difference between these systems is that the Ecopulse is powered via the power tool handpiece already in use on joint replacement sets. This removes the battery waste and reduces the size and weight of the product, resulting in less raw materials required | Obtained product weights and packaging information from manufacturers and confirmed through individual weighing. Carbon footprints calculated using UK Government GHG conversion factors for both materials and transportation. Total carbon footprint for each product projected annually. The Ecopulse model had formal carbon footprint analysis by Carbon Footprint Ltd., providing detailed report. Authors couldn't replicate this level of detail for other models, simplified method aimed to verify results and enable a more accurate comparison | Use+ reuse+ transport [kgCO2eq: 1,3] | Overall footprint of the Ecopulse significantly smaller than Pulsvac, reflecting a 2.6x carbon emissions saving compared to battery-powered Pulsvac. Assuming, 95% of cases are eligible to switch to the Ecopulse project a saving of 4,501.1 kgCO2eq=equivalent to driving 12,9634 miles in average car. Switching from battery to AC powered option for remaining 5% of cases will save further 128.5 kgCO2eq giving total saving estimate of 4,629.6 kgCO2eq (13,334 miles driven). | NR |  | C1 > C2 [N] |  |  | C1 > C2 |  |  | C1 > C2 [N] | |
| **Multiple: General, Gynaecology, Ophthalmology, Orthopaedics, Plastics, Urology** | Field 2023:USA [Operating room/ anasthesia](Field et al., 2023) | Assess whether or not low-volume anesthesia machines, deliver volatile anesthetics more efficiently than traditional anesthesia machines and, secondarily, whether this was in a meaningful economic or environmentally conscious way | RCT C1: Low-volume anesthesia machine (Maquet Flow-i C20 anesthesia workstation (MQ)) vs C2 traditional anesthesia machines (GE Aisys CS) [103 of 100 analyzed; MQ: 52; GE:51] | Patients scheduled for surgery. Height (cm, MQ vs GE): 167 ± 11 vs 169 ± 10 Weight (kg, MQ vs GE): 71 ± 12 vs 70 ± 13;BMI (kg/m2, MQ vs GE): 25 ± 3.0 vs 24 ± 3.1; Total case length (min, MQ vs GE): 210 ± 122 vs 236 ± 125; Surgery type (n, MQ vs GE): General 12 vs18; Gynecology 18 vs 5 Ophthalmology 2 vs 2 Orthopedics 6 vs 12 Otolaryngology 0 vs 1 Plastics 6 vs 7 Urology 8 vs 6 | The study team pre-filled and weighed two MQ cassettes/vaporizers to minimize workflow disruption. The study protocol was reviewed with anesthesia providers before patient transport to ensure uniform practice. During induction, fresh gas flow was set at 15 L/minute, and tidal volumes were standardized at 6-8 mL/kg ideal body weight with a PEEP of 6 cmH2O. Upon reaching steady state, fresh gas flow was reduced to 2 L/minute, and the second cassette/vaporizer was used. Data logging continued during the emergence phase | NR | Use reuse  [Metric tonnes CO2: 1] | This 20% decrease in CO2 equivalent emissions corresponds to 201 metric tons less greenhouse gas emissions over a decade compared to the GE, which is equivalent to 491,760 miles driven by an average passenger vehicle or 219,881 pounds of coal burned. To put sevoflurane usage reduction found in this study in the larger context, over the course of one year in a 2023 surgical facility with 20 operating rooms performing 5.5 cases per day, the total difference in greenhouse gas production between the two anesthesia machines would be approximately 402.26 metric tons of CO2 [10,11]. This is equivalent to the greenhouse gas production from an average passenger vehicle driven 983,521 miles, the CO2 emissions from 48.2 homes’ energy use for one year, or the greenhouse gas emissions avoided by 140 tons of waste recycled instead of landfilled | ***Gender*** (n, MQ VS GE): Female 35 vs 29, Male 17 vs 22; Age in years (n, MQ vs GE): 42 ± 14 vs 44 ± 13 | **C1>C2** |  | **C1 <> C2** | **C1>C2** | **C1>C2** |  |  |  | |
| **Ophthalmology** | Moussa 2021:UK [Three tertiary hospital units : Manchester Royal Eye Hospital (MREH), Birmingham and Midland Eye Centre (BMEC), and University Hospitals Coventry and Warwickshire (UHCW)](Moussa et al., 2021) | Report the potential reduction of carbon emissions by utilising AT instead of fluorinated gases in the management of RRDs at two large tertiary referral vitreoretinal (VR) centres | Retrospective, continuous, comparative multicentre study; C1: Air versus C2: gas tamponade [3239;NR;NR] | Patients with rhegmatogenous retinal detachment (RRD) | Fluorinated gases - sulphur hexafluoride (SF6), hexafluoroethane (C2F6) and octafluoropropane (C3F8) Air tamponade - NR | Data from three different hospitals (MREH, BMEC, UHCW) were collected through various methods including Microsoft Access databases, electronic patient records, and surgeons' logbooks. Gas masses were converted to GWP100 using IPCC reference values. Efficiency was determined through staff surveys and pharmacy order history. Statistical analysis was conducted using IBM SPSS Statistics (Version 27.0), with significance set at p<0.05 and adjustments made for multiple analyses using Bonferroni correction | Use [mean equivalent mass of CO2 (kg) per patient: 1] | UHCW reduced CO2 emissions by 47.0% and 41.1% compared to MREH and BMEC, respectively, through the use of AT. BMEC also showed a 10.0% reduction in emissions per patient compared to MREH due to different gas tamponade proportions (p<0.001). The gas cylinders at MREH result in 63 times higher CO2 emissions per RRD repair compared to UHCW. Assuming 30% of RRDs are suitable for AT, this could lead to 2,921 fewer RRDs repaired with fluorinated gas annually in the UK, reducing CO2 emissions by 44.3% to 56.6%. This could save up to 716.5 tons of CO2 annually, equivalent to electricity for 121 homes | NR |  |  |  |  | **C2>C1** |  |  |  | |
| **Ophthalmology** | Moussa 2022:UK [Three tertiary hospital units: Manchester Royal Eye Hospital (MREH), Birmingham and Midland Eye Centre (BMEC), and University Hospitals Coventry and Warwickshire (UHCW)](Moussa et al., 2022) | Investigate the direct contribution to carbon emissions of fluorinated gases used in all vitreoretinal (VR) procedures utilizing gas tamponade and assess the respective carbon footprint of the three different gas delivery system | Retrospective, continuous, comparative multicenter study; Three different gas delivery systems and fluorinated gas use [4877; NR;NR] | Patients undergoing vitreoretinal procedures utilizing gas tamponade | UHCW: 30 mL single-use gas canisters (Arcadophta, Toulouse, France); BMEC: 75 mL multi-use gas canisters (ALCHIMIA Srl, Padova, Italy); MREH: traditional gas cylinders of 2 kg SF6, 1 kg C2F6, and 1 kg C3F8 (British Oxygen Company [BOC] Healthcare, UK | Data Acquisition: VR procedures data extracted from databases at MREH, BMEC, and UHCW. Environmental Impact Calculations: Gas masses converted to Global Warming Potential (GWP) over 100 years (GWP100) using the modified Ideal Gas Law formula. Significance defined as P < .05; Statistical analysis: IBM SPSS Statistics used; Metrics for Meaningful Comparisons: Total and mean CO2 equivalent calculated for each fluorinated gas; Bonferroni correction applied for multiple analyses | Use [mean equivalent mass of CO2 (kg) per patient: 1] | The CO2 emission ranged from a mean equivalent of 3.17 kg per patient using 30 mL canisters to 124.8 kg using cylinders metric tons) for BMEC and MREH for each intraocular gas. Over 4 years, the GWP100 of equivalent CO2 at BMEC and MREH amounted to 16.7 and 276.8 tons, corresponding to an annual equivalent CO2 mass of 4.2 tons and 69.2 tons, respectively. Figure 2B shows the potential equivalent CO2 mass production at BMEC and MREH over the 4-year period if each unit were to use the three gas delivery systems (30 mL canister, 75 mL canister, and the cylinder). This resulted in a wide range of CO2 equivalent mass production, with the gas cylinders reaching 40 times higher emissions compared with the 30 mL canisters | NR |  |  |  |  |  |  |  |  | |
| Green cell=Statistically significant outcome, >< = no difference/ no detrimental effect. ASA=American Society of Anesthesiologists status, AT=Air tamponade, C1 > C2=Analysis favoured Comparator 1 over C2; C1<> C2 -=both comparator favoured in some scenario, CE=Carbon emissions, CO2=Carbon Dioxide, DEFRA=Department of Food and Rural Affairs, EPA=Environmental Protection Agency, GE=GE Aisys CS, Gi=Gastrointestinal, GHG=Greenhouse gases, GWP100=Global Warming Potential (GWP) over 100 years, IQR= Interquartile Range, MQ=Maquet Flow-i C20 anaesthesia workstation, [N]=Narrative or descriptive synthesis, NA= Not Applicable, NR=Not reported | | | | | | | | | | | | | | | | | |  |

## 10 Care delivery– additional tables

*Summary carbon emission findings- non-LCA studies – treatment regimen and surgical procedure*

| **Speciality** | **Author, date: Country [Setting]** | **Aim** | **Study design: Intervention vs Comparator [Participant N]** | **Specific health condition /procedure: Patient features** | **Intervention description** | **Carbon emission calculation methods** | **Boundary of system evaluated [Unit: CE Scope]** | **Carbon Emission findings** | **PROGRESS-PLUS** | **Patient satisfaction** | **Patient outcome** | **Patient travel saved** | **Patient time saved** | **Patient travel cost saved** | **Accessibility** | **Patient complications** | **Service cost saved** |  |
| --- | --- | --- | --- | --- | --- | --- | --- | --- | --- | --- | --- | --- | --- | --- | --- | --- | --- | --- |
| **Renal** | Chen 2017: China [Medical centre](Chen et al., 2017) | Determine carbon footprints of differing modalities/ treatment  regimes to deliver PD | Experimental- Controlled Trial: Different treatment regimen: Modality/Treatment site/Dialysate volume l/day [68 total: DAPD/Home/6 (13), DAPD/Home/8 (10), CAPD/Home/6 (16), CAPD/Home/8 (21), DAPD/Hospital/6 (1), DAPD/Hospital/8 (4), CAPD/Hospital/6 (1), CAPD/Hospital/8 (2)] | Kidney failure: Patients receiving continuous ambulatory peritoneal dialysis or daytime ambulatory peritoneal dialysis | RRT: life-sustaining  treatment for patients with ESRD. Includes HD and PD (alternative form of RRT). Daytime exchange consisted of 1.5 or 2.5% glucose with total volume of 6–8l/day in all patients. Automated PD not included. PD regimen: DAPD Home 6 13, PD regimen: DAPD Home 8 10, PD regimen: CAPD Home 6 16, PD regimen: CAPD Home 8 21, PD regimen: DAPD Hospital 6 1, PD regimen: DAPD Hospital 8 4, PD regimen: CAPD Hospital 6 1, PD regimen: CAPD Hospital 8 2 | Based on PAS protocol 2050 developed by BSI and DEFRA,^1^ activity data collected for various aspects of PD treatment, including energy/water use, patient/staff travel, paper, electricity, waste disposal, procurement. Emissions reported in kgCO2CO2eq/yr. PD treatment emissions included fixed, variable, and random components, normalized to a 2-liter PD dialysate dose and presented as median values. Emission factors for PD dialysate solution and packaging materials calculated using ICE database,^2^ including distances and modalities, collected for outpatient appointments, inpatient admissions, dialysis treatments, and laboratory investigations. PD-related energy consumption included dialysate heating and disinfection methods. Assumed optimal waste management strategies within practical limits. Primary activity data for waste production collected through direct measurement, emission factors applied accordingly | Procurement + Use (Building energy use, travel, transport), waste [kg CO2eq/year; 1, 2, 3] | Fixed emissions higher in patients receiving PD therapy in centre than at home, mostly due to consumption of electricity. PD treatment performed in centre emission than at home, resulting from reduced constituent percentage of waste disposal and transport. In total, PD treatment in centre produced less carbon footprints than HHD, showing advantage reducing GHG by medical disposal. Actual impact of PD on GHG emissions could be underestimated since waste disposal generated in manufacturing process not considered, including reuse, recycling, and sale. A no. raw materials supposed to exceed final quantity of PD products in manufacturing process | NR |  |  |  |  |  |  |  |  |  |
| **Oncology** | Coombs 2016: UK [Radiotherapy centre](Coombs et al., 2016) | To quantify the journeys and CO2 emissions if women with breast cancer are treated with risk-adapted single-dose targeted intraoperative radiotherapy (TARGIT) rather than several weeks’ course of external beam whole breast radiotherapy (EBRT) treatment | Experimental: Randomised controlled trials: C1-TARGIT IORT vs C2 -EBRT [485 Breast cancer: 249: TARGIT, 236 EBRT] | Breast cancer screening: NR | The TARGIT-A trial was an international randomised controlled trial initiated in the UK that showed that a single dose of IORT using the Intrabeam device (Carl Zeiss) was not inferior to traditional EBRT in local control after breast-conserving surgery.17 This delivers a single fraction of radiotherapy (20 gy) into the tumour cavity and adds about 20–40 min to operative procedure. Patients who received TARGIT were recommended additional breast EBRT (without a tumour bed boost) if their final tumour histology had prespecified adverse prognostic factor | For each patient, the study calculated the shortest driving distance and travel time to the radiotherapy centre using Google Maps, factoring in additional journeys for consent and radiotherapy planning for those receiving external beam radiotherapy (EBRT). Patients living more than 60 miles from a TARGIT trial centre were excluded. Comparison was made between treatment arms and trial centres. Carbon dioxide emissions from private transport were estimated based on fuel economy assumptions, with diesel and petrol cars producing different amounts of CO2 per mile driven (CO2 produced by a 40-mpg diesel car is 299 g/mile (186 g/km) and that produced by a 40-mpg petrol car is 272 g/mile (169 g/km)) | Use of fuel to attend treatment [Total CO2 emissions for all patients (tonnes), mean per patient (kg), 3] | TARGIT patients travelled significantly fewer miles: TARGIT 21 681, mean 87.1 (SE 19.1) versus EBRT 92 591, mean 392.3 (SE 30.2); had lower CO2 emissions 24.7 kg (SE 5.4) vs 111 kg (SE 8.6) and spent less time travelling: 3 h (SE 0.53) vs 14 h (SE 0.76), all p<0.0001. Patients treated with TARGIT in 2 hospitals in semirural locations were spared much longer journeys (753 miles, 30 h, 215 kg CO2 per patient)" | ***Place of residence:*** Two-thirds (63%) of the UK population live outside of towns that have a radiotherapy centre |  |  | **C1>C2** | **C1>C2** |  |  |  |  |  |
| **Oncology** | Frick 2023: USA [Radiation oncology department](Frick et al., 2023) | Characterize the outcomes of a hypo fractionated radiation schedule for transportation associated GHG emissions using rectal cancer as a case study | Experimental: Controlled Trial: C1 - SCRT vs C2 LCRT [334. SCRT:73, LCRT: 261] | Rectal cancer: Patient receiving short or long form radiation therapy for rectal cancer | The median dose delivered for SCRT was 25 Gy in 5 fractions | Estimated travel distance and time to radiation appointments using Google Maps based on patients' home addresses, selecting the shortest travel time route. GHG emissions were calculated according to vehicle type and statewide registration statistics, considering emissions from fuel production and use. GHG emissions were converted to carbon dioxide equivalents (CO2eq) for comparison. Travel costs determined using 2022 IRS mileage reimbursement rate. Comparative analyses were conducted using t-tests in Stata version 14.2 | Patient travel: Well-to- wheel model (accounts for all emissions related to fuel) : kg CO2eq, Total CO2eq, 3] | Over the total treatment course, LCRT was associated with nearly 4.5 times greater GHG emissions than SCRT. Total CO2eq emissions for LCRT and SCRT were 665.3 kg CO2eq and 149.9 kg CO2eq per patient treatment course, respectively (P < .001), with a net difference of 515.4 kg CO2eq | NR |  |  | C1>C2 | C1>c2 | C1>C2 |  |  |  |  |
| **Oncology** | Langstaff 2023; UK [Christie NHS Foundation Trust, Manchester UK](Langstaff, 2023) | To evaluate the clinical, social, financial, and environmental impacts of PBM as a supplemental  treatment for the prevention and/or reduction of oral mucositis for base of tongue and tonsil  oncology patients undergoing radical radiotherapy +/- chemotherapy. | Before and After: C1 - PBM VS C2 - Conventional care [22: Intervention (PBM): 11 vs Control: 11] | Oral mucositis: Base of tongue and tonsil oncology patients undergoing radical radiotherapy +/-chemo-therapy | The prevention of oral mucositis involves applying light to tissues to promote healing, reduce inflammation, and increase cell metabolism. PBM stimulates the natural healing process by displacing mitochondrial nitric oxide, reducing oxidative stress, and increasing cellular ATP production. A trial was conducted over four months at The Christie involving 11 head and neck patients who received PBM treatment alongside their radiotherapy for 30 consecutive days. PBM treatment was administered before each radiotherapy session using a hand-held probe to deliver light to the oral mucosa for one minute per area, totalling approximately 15 minutes per session. Subsequent treatments were self-administered by the patients, contributing to reduced treatment duration and patient burden | The carbon savings from Pharmacological Pain Management treatment estimated based on various factors. This included estimating CO2eq for unplanned admissions using emissions factors for ward bed days and patient travel distances. Reductions in CO2eq due to decreased medication usage calculated using emissions factors for pharmaceuticals. However, carbon savings from nasogastric tube insertion and feeds not included due to data limitations. CO2eq from PBM device and treatment estimated by calculating electricity use/patient, although device itself was not carbon footprinted due to its frequent use resulting in negligible emissions/use | Use+ reuse+ patient transport [kgCO2eq:1,2,3] | For a 30-day period, each patient contributes 0.04 kgCO2eq, resulting in a total carbon saving of 2,613.99 kg CO2eq per year for 11 patients. When considering eligibility for full treatment among 180 tonsil and base of tongue cancer patients annually, the savings increase to 42,774 kgCO2eq per year. Additional benefits, e.g. reduced nasogastric tube usage & medication courses post treatment, may result in further savings. CO2eq savings include reductions in bed days, travel, antibiotic prescriptions, morphine usage, and medication prescriptions during radiotherapy, favouring the treatment group in each instance | NR | NCD* | C1>C2** |  |  |  |  | C1>c2 | C1>C2 |  |
| **Oncology** | Vaidya 2022: Multi-country [Centres using TARGIT-IORT: 242 centres across 35 countries](Vaidya et al., 2022) | TARGIT-IORT delivers radiotherapy targeted to the fresh tumour bed exposed immediately after lumpectomy for breast cancer. TARGIT-A trial found TARGIT-IORT to be as effective as whole-breast radiotherapy, with significantly fewer deaths from non–breast cancer causes. This paper documents its worldwide impact and provides interactive tools for clinicians and patients | Observational: retrospective cohort: C1 - TARGIT-IORT vs C2 - EBRT [44752] | Breast cancer: Patients receiving treatment for breast cancer | Targeted intraoperative radiotherapy (TARGIT-IORT) delivers radiotherapy targeted to the fresh tumour bed exposed immediately after lumpectomy for breast cancer. This treatment delivers effective radiotherapy targeted to the fresh tumour bed exposed immediately after lumpectomy (4, 5) while sparing nearby tissues and nearby vital organs such as the heart and lung. TARGIT-A RCT (Coombs) used risk adapted single-dose TARGIT-IORT during lumpectomy | international network was established among centres using TARGIT-IORT for breast cancer treatment. Data on the first patient treated and total cases were collected via Google forms and electronic communication from numerous centres, including those not involved in TARGIT trials. The collected information was visualized using Google My Maps, showcasing each hospital's first case date and total cases treated. Patients undergoing. A previous study found that patients in the TARGIT-A trial saved an average of 305 to 753 miles of travel, depending on their location. These savings were converted into CO2 emissions saved, considering standard emissions for a medium-sized car. Study assumed higher proportion urban dwellers and developed an interactive web application for individual travel estimates, incorporating patient feedback | Patient travel [KgCO2, 3] | Scaling up the journeys saved by avoiding EBRT, because of the use of TARGIT-IORT, to the 44,752 patients, we estimate that over 20 million (20,134,909) miles of travel have already been saved, representing a carbon footprint reduction of 5.6 million kgCO2 emissions | NR |  | C1>C2 (Non cancer mortality) ><breast cancer outcomes/mortality | C1>C2 |  |  |  |  |  |  |
| **Oncology** | Woods 2015: USA [Surgery/ Operating room](Woods et al., 2015) | Quantify/ compare total greenhouse gas emissions, or ’carbon footprint’, attributable to three surgical modalities | Observational: retrospective database review: C1: Robotically-assisted laparoscopy (RA-LSC) vs C2: Laparoscopy (LSC) vs C3: Laparotomy (LAP) [50] | Endometrial cancer: Patients undergoing staging procedure for endometrial cancer | NR | Data from 150 staging surgeries conducted between 2008 and 2011 collected to calculate carbon footprint by summing associated solid waste production and energy consumption. Waste production categorized, and energy consumption determined for various components (environmental, equipment, instrument, and robotic systems), with statistical analyses comparing variables and controlling for confounding factors. Student’s t-test, Kruskal–Wallis test, or ANOVA, with Bonferroni’s method used for multiple comparisons. Categorical variables compared using Pearson’s χ2 test, with an α-level of 0.05 | Transport from clinic to outsourcing facility) + waste disposal: Cold pack landfill Emissions solely based on transportation to landfill (20 miles), Across all stages: materials: foam, cardboard and cold pack plastic [ KgCO2eq ,1] | Total carbon footprint for all 150 procedures: 4498 kg CO2eq, average 30 kg CO2eq/patient. RA-LSC=highest carbon footprint (40.3 kg CO2eq/patient), followed by LSC (29.2 kg CO2eq/patient) and LAP (22.7 kg CO2eq/patient). Energy consumption varied, with RA-LSC consuming most energy (26 kg CO2eq/patient), followed by LSC (18 kg CO2eq/)patient and LAP (14.4 kg CO2eq/patient). Environmental energy usage comparable across modalities, with LAP producing least solid waste (8.3 kg CO2eq/patient), followed by LSC (11.24 kg CO2eq/) patient and RA-LSC (14.3 kg CO2eq/patient) | NR |  |  |  |  |  |  |  |  |  |
| * The requirement to attend a different area of the hospital for PBM posed challenge for some patients with reduced mobility, ** Patient outcome: pain, hospital admission. Intervention participants generally prescribed pain medication longer. Green cell - statistically significant outcome, [N] = supported by narrative write up (no formal statistics), ATP - Adenosine triphosphate, BSI=British Standards Institution, CAPD=Continuous Ambulatory Peritoneal Dialysis, C1> C2 -C1 u over C2, CO2=Carbon Dixoxide, DAPD=Daytime Ambulatory Peritoneal Dialysis, DEFRA- Department for Environment, Food, and Rural Affairs, EBRT - External beam radiotherapy, ESRD - End Stage Renal Disease, GHG - Greenhouse gases, HD - Haemodialysis, HHD - Home Haemodialysis, HHM - Home Health Monitoring, ICHD=In center Haemodialysis, LAP - Laparotomy, LCRT - Long course radiation therapy, LSC - Laparoscopy, NA - Not Applicable; NR - Not reported, PBM=Photobiomodulation, PD=Peritoneal Dialysis, RA-LSC - Robotically-assisted laparoscopy, RRT=Renal Replacement Therapy, SCRT - Short court radiation therapy, SDPP - Systematic Depot Pickup Problem , TARGIT-IORT - Targeted intraoperative radiotherapy | | | | | | | | | | | | | | | | | | |

*Care pathway – overview of non-LCA studies*

| **Specialty** | **Author, date: Country [Setting]** | **Aim** | **Study design: Comparison (Intervention vs Control/ Comparator) [Participants N]** | **Specific health condition: Patient characteristics** | **Intervention description** | **CE Calculation methods** | **Boundaries of system evaluated [Unit: CE Scope]** | **Summary of CE findings** | **PROGRESS-PLUS** | **No. F2F visits** | **Patient travel distance** | **LOS** | **Service costs** | **N. Physio appointments** | **Readmissions** | **Other outcomes measured with no comparative data** |
| --- | --- | --- | --- | --- | --- | --- | --- | --- | --- | --- | --- | --- | --- | --- | --- | --- |
| **Orthopaedics** | Cooper 2023: UK [Separate sites within Calderdale and Huddersfield NHS Foundation Trust](Cooper et al., 2023) | To understand if use of digital system could improve patient experience and efficiency of post operative physiotherapy care provided. (1) Explore if wearable sensor reduced no. F2F physiotherapy visits, (2) Measured reception and utilization of messaging system by patients and clinicians | Experimental: Pilot controlled trial - CE data based on modelling: C1: Digital day case care pathway vs C2: standard care pathway [21, 14 day case, 7 control (5 short stay, 2 long stay)] | Knee arthroplasty: 16 TKA, 5 UKA. Mean age: 57.6 yrs (SD 8.9 yrs), 9f & 12m patients | See paper for detail | Model created using pilot study data for all 435 knee arthroplasties/ yr where 79% knee replacement patients have ASA grade of I or II10. Assuming 65% patients would have access to a smart device for use with BPM pathway, total of 218 patients (51.4%) enrolled in digital day-case program. Decision tree created showing not all patients would be suitable for accelerated digital day-case pathway, as patients with ASA grade of ‡III unsuitable for discharge within 24hrs and patients without smartphone unable to utilize remote-monitoring system. Only 51.4% of all possible knee arthroscopy patients in budget-impact model shown to be suitable for accelerated digital day-case program, and remaining 48.6% budgeted according to the standard pathway. To test robustness of reported model, several parameters subjected to univariate deterministic sensitivity analysis to determine impact of variation in parameters. Parameters systematically varied between upper and lower bounds. Costs varied by ±20% of the base case values, and LOS and percentage of patients with ASA grade of ‡III varied by ±20%, according to guidance from Hospital Episode Statistics database and National Joint Registry.^a^ Data regarding hospital LOS in digital day-case cohort presented as mean and 95% confidence interval, time spent on messaging is estimated. See paper for detail re: sustainability assumptions and parameters | BMP pathway box & USB-C charger, strap& device, patient bed day, outpatient appointment, patient travel [kg CO2: NA] | NA | NR |  |  | C1> C2 (N) | C1> C2 (N) | C1> C2 (N) |  | C, PF, PS, ST |
| **Orthopaedics** | Cooper 2022: UK [2 separate sites within the Calderdale and Huddersfield NHS Foundation Trust](Cooper et al., 2022) | Assess implementation of digital day-case pathway for knee replacement surgery | Experimental: Pilot controlled trial - CE data based on modelling: C1: Digital day case care pathway vs C2: Standard care pathway [21, 14-day case, 7 control (5 short stay, 2 long stay] | Knee arthroplasty: 16 TKA, 5 UKA. Mean age: 57.6 yrs (SD 8.9 yrs), 9f & 12m patients |  | NR | BMP pathway box & USB-C charger, strap& device, patient bed day, outpatient appointment, patient travel [: Scope 1, 2, 3] | Because of reduced No. F2F visits, model predicted incremental reduction of 119,381 kg CO2 emissions associated with knee replacement procedures | NR | C1> C2 (N) |  |  |  |  |  | PC, PF, PS |
| **Cardiology** | Nielsen 2022: UK [Cardiac ICU Southampton Hospital](Nielsen, 2022) | NR | Before and After: C1: Early mobilisation vs C2: Before intervention [238 recruited to mobilisation programme]^b^ | Patients receiving open heart surgery | CICU team recruited therapy technician to work alongside qualified physiotherapist, to help set up project and deliver therapy sessions. Technician helped: educating CICU staff, including use of Motomed equipment required for exercising sedated patients. Therapy assistant systematically initiated mobilising patients who fitted protocol criteria. These patients received 30mins rehab 2xday, continuing until discharge from CICU. Staff selected highest level of activity in which patient could participate eg if patient intubated and ventilated, Motomed device used for passive exercise; if patient awake then options included sitting on edge of bed, standing, marching on spot, transferring from bed to chair and mobilising | Data gathered before and after introduction of EMP on no.days patients received artificial ventilation, LOS intensive care, in CHC beds, on ward and total hospital LOS. CE calculation methods NR | NR. Assumed: Use/Reuse [tonnes CO2eq: CT] | Carbon footprint of no. days saved: 48.5 tonnes CO2eq, equivalent to annual carbon footprint of almost 5 UK citizens and 18 return trips London-Sydney in economy class over 2-year programme | NR |  |  | C1> C2 (N)^c^ | C1> C2 (N) |  |  |  |
| **Cardiology** | Zander 2011: UK [NHS hospital, east of England](Zander et al., 2011) | Little attention has been paid on the carbon footprint of different healthcare service models. We examined this question for service models for patients with acute STEMI | Modelling: C1 Ambulance transport based on pPCI model in tertiary centres vs C2 Thrombolysis model based in hospitals [41449 patients. pPCI care model: 3 hospitals, thrombolysis model: 18 hospitals] | MI during 5-year period: NR | pPCI care model based in tertiary centres. pPCI benefits are critically dependent on its timeliness (more so than for thrombolysis). Clinical guidelines recommend pPCI ideally conducted within 600 from time administration of thrombolysis would have been possible, and within 900 from ‘first medical contact’. Unlike thrombolysis that can be effectively and safely administered in the community (pre-hospital thrombolysis) or at GH, pPCI requires ‘24/7’ availability of on-call interventional cardiology team and cardiac catheterization facilities. Clinical quality best assured in high volume centres. These factors restrict pPCI provision to a much smaller number of care points (i.e. tertiary cardiac units) compared with district GHs. Change in pPCI care model for STEMI patients means ambulances have to travel to 3xcardiac centres (as opposed to 18 district GHs in historical thrombolysis model) | Two matrices of destination ‘care points’ constructed. 1 for thrombolysis care model (corresponding to 18 regional district GH with A&E departments) and one for pPCI care model (corresponding to 3 regional pPCI centres). ArcEditor GIS software with Network Analyst extension used to compute ‘real world’ distances from LSOA centroids to district GH A&E destinations, using OS Meridian2 East of England road network info. Distance from each LSOA centroid to closest care point under two models selected from all other possible care point destinations (either one of 18 district GHs under thrombolysis model or 1 of 3 pPCI centres under new model) using Excel ‘MIN’ formulas. For either service model, total (minimum) transport distances required for all STEMI patients from their LSOA centroid to closest care point during 5-year study period averaged=mean ambulance journey mileage required for management of a STEMI patient under either service model. Mileage converted to CO2 emissions using standard coefficients, published by DEFRA.12 In absence of CF specifically for ambulances, used CO2 CF for ‘large vans’ as appropriate proxy, (1 km travelled to 0.2661 kgCO2).12 This CF estimated using average values for UK van fleet in 2005, calculated based on average speed and distance of UK trips and adds 15% on emissions to model ‘real world’ driving effects | Patient transport via ambulance [CO2 tonnes: 1] | Average distance to transport STEMI patient to closest care point: 13.0km (thrombolysis model) vs 42.2km (pPCI model)=CO2 emissions of 3.46 and 11.2kg, respectively. Introducing pPCI management>triples ambulance journey mileage and associated CE required for STEMI patient transport (factor of 3.24). Using HES online data 2002 –03 to 2006 –07 for all MI admissions (STEMIs and non-STEMIs) and multiplying by 0.4 to derive estimate of only STEMI events,13,14 = 3316 STEMI hospital admissions expected to occur/yr in East of England. If all events occurred out-of-hospital and required transport to either thrombolysis or pPCI care point=total patient (ambulance) journey mileage of 43 100km/yr (or 11.5tonnes CO2 emissions) for thrombolysis model and 139 000km/year (or 37.2tonnes CO2) for pPCI model. Thus, introducing pPCI care model: additional 96 700km/yr ambulance travel and additional 25.7 tonnes/yr CO2 emissions. If CF (CO2 emissions/km traveled) changed from baseline 0.2661 to 0.2042 (for cars), then emissions/STEMI patient proportionally reduced to 2.7 and 8.6kg CO2 under thrombolysis and pPCI care models, respectively. If CF increased to 0.4200 (half of CF value for average bus) emissions/STEMI patient increase proportionally to 5.5 and 17.7kg CO2, respectively. If no. STEMI events requiring ambulance transfer to NHS hospital approx. halved to 1650/yr,13 total distance travelled will be proportionally reduced to 21 500 and 69 600 km/yr for thrombolysis and pPCI care models, respectively, whereas CO2 emissions proportionally reduced to 18.9 and 61.4 tonnes/year under the pPCI care model | NR |  | C2>C1(N) |  |  |  |  |  |
| **Urology** | Phull 2023: UK [NA: 116 UK hospital trusts](Phull et al., 2023) | Investigate estimated difference in carbon footprint between day-case and inpatient TURBT surgery in England | Observational: Retrospective Review of Data: C1: Day pathway vs C2: Inpatient stay [209269. 41 583 (20%) day cases, 167680 (86 %) inpatients] | TURBT: Day vs inpatient n (%) : Age group <50 yr: 2633 (6.3) vs 5057 (3), 50–59 yr: 5032 (12) vs 12 427 (7), 60–69 yr: 10487 (25) vs 33 895 (20), 70–79 yr: 15522 (37) vs 61 721 (37), Over 80 yr: 7909 (19) vs 54 586 (33). Gender: Female: 10 804 (26) vs 36, 817 (22). HFRS category: None: 25 194 (61) vs 79 093 (47), Mild: 12 056 (29) vs 57 039 (34), Moderate: 3987 (9.6) vs 27 616 (17), Severe: 346 (0.82) vs 3938 (2.3). IMD quintile: 1 (most deprived): 6298 (15) vs 28 965 (17), 2: 7626 (19) vs 31 014 (19), 3: 8966 (22) vs 34 845 (21), 4: 9134 (22) vs 36 560 (22), 5 (least deprived): 9206 (22 )vs 34 716 (21) | Day case admission for transurethral resection of bladder tumour surgery | Carbon footprint of day-case and inpatient TURBT procedures calculated using Sustainable Healthcare Coalition (SHC) carbon footprint data.^d^ SHC data estimate carbon footprint of ‘‘average’’ surgical procedure as 35.1 kg CO2eq.^e^ This figure is a generic estimate, including items covering consumables, equipment, medical gases, staff travel, energy, water use, and waste; used in this study as a proxy for carbon footprint for TURBT. All data analysed on a secure server controlled by NHS England using standard statistical software. Data summarised using standard descriptive statistics, depending on data level. Carbon factors calculated at patient level and aggregated for day case and overnight-stay patients and per financial year, as appropriate. Total carbon saved calculated using 2013–2014 as baseline. To provide context, CO2eq emissions converted to CO2eq to power an average UK home with electricity for 1 yr using conversion factor of 1098.9 kg CO2eq/home/ yr | Use/reuse, waste, staff transport [kg CO2: 1, 2, 3] | From 2013–2014 to 2021–2022, increase in day-case surgery reduced carbon footprint of TURBT by 24kg CO2eq/patient. Estimated cumulative saving from baseline of 2013–2014 was approx. 2.9 million kg CO2eq (2.9 kilotons), equivalent to powering 2716 UK homes with electricity for 1yr. For financial year 2021–2022, average carbon footprint=41.5kg CO2eq for pathway involving day case surgery and 115.0kg CO2eq for pathway with inpatient surgery. Adjusted upper-quartile value=39% day-case surgery. If all 87 trusts below upper-quartile for day-case surgery rate had met this target, total carbon saving would be 217,599kg CO2eq (equivalent to powering 198 homes for 1yr). Adjusted upper-decile value=51% day-case surgery. If all 104 trusts below upper-decile for day-case surgery rate had met this target, total carbon saving=372,127kg CO2eq (equivalent to powering 339 UK homes with electricity for 1yr) | ***Personal characteristics associated with discrimination:*** no evidence of nonlinearity in the relationship between log odds of day-case surgery and age. Day-case patients were younger and less frail than inpatients |  |  | C2> C2 (S) |  |  | C2> C2 (N) |  |
| **Ear, Nose and Throat** | Burton 2022: UK-Wales [NR](Burton, 2022) | To support care pathway to be retained post-Covid and spread to other hospitals in Wales and UK, by: Analysing social, financial and environmental impact of new fractured nose manipulation LA pathway, compare new LA pathway to fractured nose manipulation GA pathway, embed change within department by educating clinicians on benefits of new pathway, capture data on patient satisfaction for fractured nose manipulation under LA | Modelling: C1: LA vs C2: GA [NA] | Patients requiring urgent but not life/ limb saving treatment for fractured nose | Treatment for fractured nose under LA. ENT team involved in implementing change, including consultants, specialist registrars, senior house officers, foundation doctors, ANPs, ENT clinic nurses, HCAs and receptionist. LA procedure protocol created, with procedures initially performed by specialist registrars. Training/competency document subsequently created to formalise the teaching and assessment process for ANPS and junior doctors, expanding no. competent staff available to safely perform LA procedure. Staff reassured strong evidence for use of LA for fractured nose manipulation in most patients. While bleeding risk from LA procedure is small, plans and stock put in place in case of any significant epistaxis | Theatre data from 3 years post pandemic obtained to estimate average no. adult  procedures performed/ yr with average of 122 adult cases/yr. However, as not all  cases are suitable for LA, took 90% of cases for more realistic estimate of potential environmental and financial savings. Hybrid approach to carbon footprinting (bottom up and top-down methods used) taken. Applied emissions factors to activities and consumables involved in GA and LA pathways to compare CO2eq of procedure under each approach. Applied saving from performing LA procedure to 90% of total procedures for fractured nose manipulation completed annually to provide CO2eq saving. Emissions factors taken from The Sustainable Development Units 2015 Care Pathways Guidance on Appraising Sustainability.^f^ Attendance at outpatient clinic (Emission factor for a GP consultation used as more closely mimics 10-min. ENT casualty clinic appointment for assessment of fractured nose) and elective theatre (1.14 kgCO2eq/per visit), patient travel to and from appointments (2.9 kgCO2eq/one way), A&E visit (13.8 kgCO2eq, Inpatient bed day - low intensity ward (37.9 kgCO2eq/ bed day), surgical procedure (35.1 kgCO2eq/66 minutes) | Use, reuse, patient transport [ kg CO2eq: 1, 2, 3] | Based on 90% of 122 annual cases switching to LA instead of GA, anticipate savings of 4,137.26 kgCO2eq/yr, equivalent to driving 11,916m (19,177km), or 15 return trips from Cardiff to Glasgow in average car. If 100% of cases performed under LA, savings would increase to 4,596.96 kgCO2eq, equivalent to 13,239 m (21,306 km) travelled in average car, or 16.7 return journeys from Cardiff to Glasgow by car | NR |  |  |  | C1> C2 (N) |  |  | PS |
| ^a^NJR. National Joint Registry. https://www.njrcentre.org.uk/njrcentre/default.aspx2021, ^b^Conflicting numbers presented. Previous audit without input: N=41, Current trial 17-18: N=121, Current trial 2018-19: n=117 = Total of 279. ^c^Including ventilation, cardiac ICU, cardiac high care, ward and total LOS, ^d^Coalition for Sustainable Pharmaceuticals and Medical Devices. Care pathways: guidance on appraising sustainability, main document. Newton Abbot, UK: Sustainable Healthcare Coalition; 2015. ^e^Coalition for Sustainable Pharmaceuticals and Medical Devices. Care pathways: guidance on appraising sustainability, surgical procedure module. Newton Abbot, UK: Sustainable Healthcare Coalition; 2015. ^f^Moore A, Burton H. Genetic ophthalmology in focus. A needs assessment and review of specialist services for genetic eye disorders, PHG Foundation, 2008. ACP=Advanced Care Practitioner, ANP=Advanced Nurse Practitioners, ASA=American Society of Anesthesiologists, C=Complications, C1/C2=Comparator 1 or 2, CE=Carbon Emission, CHC=Cardiac High Care, CICU=Cardiac Intensive Care Unit, CO2=Carbon Dioxide, CT=Can’t Tell, DEFRA=Department for Environment, Food, and Rural Affairs, EMP=Early Mobilisation Programme, ENT=Ear, Nose and Throat, F=Female, F2F=Face to Face, GA=General Anaesthetic, GH=General Hospital, HCA=Health Care Assistants, ICU=Intensive Care Unit, IV=Intravenous, LA=Local Anaesthetic, LOS=Length of Stay, M=Male, [N]=supported by narrative write up (no formal statistics), NA=Not Applicable, MR=Modified Release, NHS=National Health Service, NR=Not Reported, OT=Occupational Therapist, PC=Patient Compliance PF=Physical Functioning, pPCI=Primary percutaneous coronary intervention, PS=Patient Satisfaction, ROM=Range of Motion, SD=Standard Deviation, ST=Staff time, STEMI=ST segment elevation myocardial infarction, TKA=Total Knee Arthroplasty, TURBT=Transurethral Resection of Bladder Tumour, UKA=Unicondylar Knee Arthroplasty | | | | | | | | | | | | | | | | |

## 11 Multiple components – additional tables

| Specialty | **Author date: Country [Setting]** | **Aim** | **Study design: Intervention vs Comparator [Participants N]** | **Specific health condition /procedure: Patient features** | **Intervention description** | **Carbon emission calculation methods** | **Boundary of system evaluated [Unit: CE Scope]** | **Carbon emission findings** | **PROGRESS-PLUS** | **Patient Satisfaction** | **Patient Clinical outcome** | **Patient travel distance saved** | **Clinician Satisfaction** | **Service costs saved** | **Water consumption** | **Service energy savings** | **Service waste saved** |
| --- | --- | --- | --- | --- | --- | --- | --- | --- | --- | --- | --- | --- | --- | --- | --- | --- | --- |
| **Renal** | Bendine 2020; France [Nephro-Care centres](Bendine et al., 2020) | Describe data collection begun in the Nephro-Care centres in France and the changes observed during a 13-year period regarding environmental parameters | Observational: retrospective cohort, C1: Multiple-Setting; C2: Standard practice [2642 in 2018] | Renal failure: Patients receiving haemodialysis treatment | FMC's environmental commitment started in 2005 with eco-reporting on electricity, water consumption, and waste production. Dialysis-related consumption tracked individually, with data sent quarterly to headquarters for benchmarking. Environmental plans, implemented in phases, focus on awareness, eco-efficient technologies, and ISO 14001 certification, with precise targets set in subsequent plans. See paper for full detail | Number of dialysis sessions automatically recorded by clinic management system, linked with dialysis machines (4008, transitioning to 5008). Carbon equivalents of power, water, and waste sparing are calculated using a tool from the Association Bilan Carbone, accessible to trained personnel | Use+ reuse [CO2 tonnes equivalent: 2] | Due to electricity savings, CO2 equivalent reduced for analysed period from 92,400 tons, 10,000 tons for care-related wastes, and 17.5 tons for water. In total, 102,440 tons of CO2 equivalent were saved, an amount that represents CO2 production of plane flying around the globe 11,500 times | NR |  |  |  |  |  | C1>C2 | C1>C2 | C1>C2 |
| **Renal** | Hardy 2022; UK [Satellite dialysis unit, Leeds teaching Hospitals NHS Trust](Hardy, 2022) | To reduce carbon & waste | Modelling; C1 - Multiple: Setting; C2 - Conventional care [NA] | Haemodialysis: Patients receiving haemodialysis treatment | Aims: 1. Reducing disinfections of dialysis machines to once every 24 hours in a staggered manner and replacing others with rinsing processes. 2. Placing dialysis machines on standby mode after the initial priming process until patients are connected. 3. Decreasing pharmacy deliveries from weekly to biweekly for the satellite dialysis unit. Staff engagement was sought and encouraged for aims 1-3, with daily handovers used to discuss proposed plans and gather input. The entire unit's visibility aided in reminding staff of the changes during the initial stages. Aim 3 also involved exploring storage space availability, rearranging cupboards, and relabelling them to accommodate the new delivery schedule | Aim 1: Electricity and water usage during disinfection and rinse cycles for one dialysis machine were measured over 24-hour periods, with projections made for one year. Aim 2: Data on waiting times between priming dialysis machines and patient connection were collected over a week to calculate average daily waiting times per dialysis station. Consumption of electricity, water, and central acid during this waiting period was measured per minute and projected over a year, with potential additional savings from reduced central acid deliveries. Aim 3: Reducing pharmacy deliveries from weekly to biweekly could save an average of 104 miles in transportation per year. Collaboration with other satellite units within the Trust is underway to explore similar changes for increased mileage savings | Use+ reuse, + transport [kgCO2eq: 1,3 | Conversion factors for travel, water, and energy sourced from UK government BEIS 2021 database, pharmaceutical carbon factors obtained from Greener NHS Team 2020-21. The implemented changes from aims 1, 2, and 3 projected to save 1,914.4 kgCO2eq and £2,837.05. Other ongoing projects were excluded from analysis. Implementing the proposed changes could result in a reduction of 0.1845 kgCO2eq per patient per dialysis session. If similar changes were applied nationwide to the 24,365 individuals receiving dialysis in the UK, and assuming consistent energy consumption across the 70 renal centers, the national CO2 emission reduction could be approximately 4,495 kg per treatment session, translating to around 700 tonnes per year if each patient underwent thrice-weekly dialysis | NA |  |  |  |  | C1>C2 | C1>C2 | C1>C2 |  |
| **Radiation oncology** | Cheung 2023; Canada [Single institution](Cheung et al., 2023) | To assess the environmental effect of our single radiation oncology department’s collective strategic changes implemented during the pandemic to reduce foot traffic to the hospital | Experimental: Retrospective before and after. Quality improvement C1 - Multiple: waste reduction, care delivery, travel, telemedicine; C2 - before intervention [10 175. Intervention: 4877, Control: 5298] | Cancer, N.S: Control v intervention - Treatment course (N/%): Conventional curative 2175 (41.0%) vs 1452 (29.8%) Hypofractionation curative 969 (18.3%) vs 1,236 (25.4%) Conventional palliative 106 (2.0%) vs 123 (2.5%) Hypofractionation palliative 1552 (29.3%) vs 1488 (30.5%) SBRT 496 (9.4%) vs 578 (11.9%) | Specifically, the main changes that were implemented at our department were an increased use of (1) hypo fractionated regimens, (2) virtual patient care, and (3) personal protection equipment during the COVID-19 pandemic | Patient travel distances were calculated from postal codes using Google Maps. Assumptions included passenger vehicles, normal driving conditions, and exclusion of out-of-province patients. CO2 emissions were calculated based on vehicle types. LINAC power usage was measured per fraction delivered to patients, with CO2eq calculated based on electricity generation data. PPE orders (bouffant cap, disinfectant wipes, face shields, gloves, disposal gowns, surgical masks and N95 respirators, safety glasses, and shoe covers) were compared between two fiscal years, with CO2eq calculated based on LCA | Use+ reuse+ patient travel [kg CO2eq: 2, 3] | The changes in hypofractionation radiation regimens, with the consequent reduction in radiation therapy visits, combined with the rapid switch from in-person to virtual care, even accounting for the slight increase in LINAC power usage, and PPE, translated into a net saving of 743,641 kg CO2eq (Fig. 3). The CO2eq emission from the accounted sources was 1,956,175 kg CO2eq in 2019 to 2020 FY and 1,212,534 kg CO2eq in 2020 to 2021 FY, representing a 39% reduction in the CO2eq emission. The CO2eq saving was equivalent to the CO2eq sequestered by approximately 12,000 seedling trees planted and grown for 10 years25 or the CO2eq from the annual energy consumption of 182 Canadian households.26 | Control vs Intervention group. ***Mean age***: 63.6 [range 1.6-101.9] vs 64.5 [range 1.8-99.3]. ***Gender*** Male/ Female (%): 52:48 vs 50:50 |  | C1>C2 | C1>C2 |  |  |  |  |  |
| **Gastroenterology** | Materacki 2023; UK [4 Endoscopy units, Gloucestershire](Materacki, 2023 ) | To establish a multiprofessional green endoscopy working group in Gloucestershire; To make at least one change to improve sustainability in endoscopy in Cheltenham General Hospital and measure its environmental (CO2eq), financial and social impact | Before and After; C1: Multiple - setting, product-level C2: Conventional care [17;NA:NA] | Endoscopy: Patients receiving endoscopy | The endoscopy department implemented several sustainability initiatives: 1. Offering patients electronic copies of pre-procedure booklets via email. 2. Designating a 'green champion' within the bookings/ administrative team to enhance engagement. 3. Negotiating with the leaflet printing company to use recycled paper for future leaflet orders. 4. Providing washable patient gowns instead of single-use shorts during lower GI endoscopies. 5. Reducing prophylactic inkopad usage by implementing responsive practice based on endoscopist request or high-risk situations. 6. Encouraging patients to bring their own water bottles or reusable coffee cups to reduce single-use cup usage post-procedure | 1. Pre-endoscopy leaflets: The environmental impact was estimated by calculating the carbon emissions factor per leaflet. We have calculated the paper and envelope emissions using weights, postage and travel. 2a. Single use shorts. The environmental impact was determined by considering materials, transport, and waste disposal. Reusable gowns: Carbon emissions were approximated based on assumptions about gown weight, material, washing process, and disposal. 2b. Inkopads: Carbon emissions per inkopad were calculated based on material weight, with assumptions about distribution from Sweden | Use+ reuse+ transport (from manufacturer to Gloucester Royal Hospital or supplier to hospital) [kgCO2eq: 1,3] | 1. 49% of patients opted for electronic leaflets which would reduce the annual no of paper leaflets from 16,971 to 8,655, resulting in savings of 1,701 kgCO2eq. 2a. About 95% of patients undergoing colonoscopy & flexible sigmoidoscopy were estimated to switch to reusable gowns. The carbon emissions saved annually from reduced shorts manufacture and waste incineration and considering the procurement and laundry costs for reusable gowns, were estimated at 1,886 kgCO2eq. 2b Estimating a reduction in inkopad use by 10% for OGD or flexible sigmoidoscopy and 50% for colonoscopy, approx. 12,735 fewer inkopads would be used annually, resulting in savings of 3,032 kgCO2eq from reduced manufacture and incineration. Implementing these interventions across all endoscopy units would yield annual savings of £9,568 and 6,619 kgCO2eq | NR |  | >< [N] |  | C1<>C2 [N] | C1>C2 |  |  |  |
| Green cell - statistically significant outcome, >< - no difference/ no detrimental effect , [N] = supported by narrative write up (no formal statistics), C1 > C2 - Analysis favoured Comparator 1 over C2, C1<> C2 - both comparator favoured in some scenario, CE=Carbon emissions, CO2=Carbon Dioxide, CSSU=Central Sterile Services Unit, CTR=Carpal Tunnel Release; EMEA=Europe, Middle East and Africa , FMC =Fresenius Medical Care, GHG=Greenhouse Gases; GP=General Practitioner; ICE= The Inventory of Carbon & Energy database; KPI=Key Performance Indicators, [N]=Narrative/descriptive analysis – no formal statistics, NA =Not Applicable, NR=Not Reported, NHS=National Health Trust, OGD=Oesophago-Gastro-Duodenoscopy , PPE=Personal Protective Equipment, SBRT=Stereotactic Body Radiation Therapy, WCP=Welsh Clinical Portal | | | | | | | | | | | | | | | | | |
