# Supplementary material for: Effectiveness of Interventions to Reduce Carbon‐Emissions Within Secondary Healthcare: Systematic Review and Evidence and Gap Map
Source: Campbell Syst Rev. 2025 Dec 23;21(4):e70077. doi: 10.1002/cl2.70077 (PMC12723626; doi:10.1002/cl2.70077)
Supplement: Supplementary file 6 — Excluded_studies. [file CL2-21-e70077-s005.docx]

## Excluded studies

| **Reference** | **Reason for exclusion** |
| --- | --- |
| (2013). Mainstreaming Carbon Management in Healthcare Systems: A Bottom-Up Modelling Approach. **47:** 678-686. | Specialty |
| Adams, L., et al. (2009). "Development of nurse-led, cancer follow-up clinics in community hospitals." European Journal of Cancer, Supplement **7**(2-3): 237. | Abstract |
| Agarwal, B. B. and K. C. Mahajan (2010). "Carbon footprint of laparoscopic cholecystectomy performed with or without energized dissection-a case controlled study." Surgical Endoscopy and Other Interventional Techniques **24**(1 SUPPL. 1): S590. | Abstract |
| Al Fannah, J., et al. (2023). "Towards a green hospital approach in Oman: A case study of quantifying an environmental impact." International Journal of Health Planning and Management. | Specialty |
| Alshqaqeeq, F., et al. (2020). "Choosing radiology imaging modalities to meet patient needs with lower environmental impact." Resources, Conservation and Recycling **155**: 104657. | CE data NR |
| Andrade, R. S., et al. (2014). "Endobronchial ultrasonography versus mediastinoscopy: a single-institution cost analysis and waste comparison." The Annals of thoracic surgery **98**(3): 1003-1007. | Specialty |
| Ang, K. S., et al. (2023). "Developing a quality improvement project to tackle the desflurane problem." Bmj Open Quality **12**(1). | Specialty |
| Babu, M. A., et al. (2019). "Greening the Operating Room: Results of a Scalable Initiative to Reduce Waste and Recover Supply Costs." Neurosurgery **85**(3): 432-437. | CE data NR |
| Bacon, M. (2014). "Occupancy analytics: a new basis for low-energy–low-carbon hospital design and operation in the UK." Architectural Engineering & Design Management **10**(1/2): 146-163. | Specialty |
| Baddeley, R., et al. (2022). "Green Endoscopy: Counting the Carbon Cost of Our Practice." Gastroenterology **162**(6): 1556-1560. | Study design |
| Balys, M., et al. (2021). "LCA and economic study on the local oxygen supply in Central Europe during the COVID-19 pandemic." Science of the Total Environment **786**: 147401. | Specialty |
| Baxter, N. B., et al. (2021). "Variability in the Use of Disposable Surgical Supplies: A Surgeon Survey and Life Cycle Analysis." Journal of Hand Surgery **46**(12): 1071-1078. | Study design |
| Bell, J., et al. (2022). "Carbon footprint of maintenance and reliever therapy (MART) versus maintenance plus SABA (Mx+SABA) regimens for asthma: Results from the healthCARe-Based environmental cost of treatment (CARBON) programme." European Respiratory Journal **60**(Supplement 66). | Abstract |
| Black, S. and K. Torlei (2013). "Designing a New Type of Hospital Gown: A User-centred Design Approach Case Study." Fashion Practice-the Journal of Design Creative Process & the Fashion Industry **5**(1): 153-160. | Study design |
| Blankush, J. M., et al. (2020). "Unforeseen Consequences: Comparative Environmental Impacts of Robotic and Open Ventral Hernia Repair." Journal of the American College of Surgeons **231**(4 Supplement 2): e149. | Abstract |
| Bolger, M. P., et al. (2016). "The environmental impact of interventional radiology." Cardiovascular and Interventional Radiology **39**(3 Supplement 1): S213. | Abstract |
| Boucheron, T., et al. (2022). "Cost and Environmental Impact of Disposable Flexible Cystoscopes Compared to Reusable Devices." Journal of Endourology **36**(10): 1317-1321. | CE data NR |
| Bunani, A. and T. Villaneuva (2014). "Green mileage - Sustainable program towards eco-friendly dialysis in Saudi Arabia: Results of phase one." Pediatric Nephrology **29**(9): 1780. | Abstract |
| Burguburu, A., et al. (2022). "Comparative life cycle assessment of reusable and disposable scrub suits used in hospital operating rooms." Cleaner Environmental Systems **4**. | Specialty |
| Cameron, T. W., 3rd, et al. (2021). "Medical Waste Due to Intravitreal Injection Procedures in a Retina Clinic." Journal of vitreoretinal diseases **5**(3): 193-198. | Study design |
| Campion, N., et al. (2015). "Sustainable healthcare and environmental life-cycle impacts of disposable supplies: a focus on disposable custom packs." Journal of Cleaner Production **94**: 46-55. | CE data NR |
| Carpenter, M., et al. (2023). "Robotic-assisted radical prostatectomy as a day-case procedure." Anaesthesia **78**(Supplement 1): 10. | Abstract |
| Chau, C., et al. (2022). "The environmental impacts of different mask options for healthcare settings in the UK." Sustainable Production and Consumption **33**: 271-282. | Study design |
| Chenven, L. and D. Copeland (2013). "FRONT-LINE WORKER ENGAGEMENT: GREENING HEALTH CARE, IMPROVING WORKER AND PATIENT HEALTH, AND BUILDING BETTER JOBS." New Solutions: A Journal of Environmental & Occupational Health Policy **23**(2): 327-345. | CE data NR |
| Chinchilla, G., et al. (2022). "Project Green Endoscopy: GE online successfully reduces carbon footprint associated with patient travel in a metropolitan endoscopy unit." Journal of Gastroenterology and Hepatology **37**(Supplement 1): 249. | Abstract |
| Coca, K., et al. (2022). "Impact of Telemedicine on Financial Burden to Patients and Carbon Footprint at an Endocrine Oncology Clinic." Otolaryngology - Head and Neck Surgery **167**(1 Supplement): P23. | Abstract |
| Connor, M. J., et al. (2019). "Clinical, fiscal and environmental benefits of a specialist-led virtual ureteric colic clinic: a prospective study." BJU International **124**(6): 1034-1039. | Duplicate |
| Cowboy, E. N., et al. (2009). "Reducing the Carbon Footprint by tele-ICU model." Chest **136**(4). | Abstract |
| Cummings, J., et al. (2022). "Estimating the carbon footprint of the radiotherapy pathway and changes in response to COVID-19." Radiotherapy and Oncology **170**(Supplement 1): S893-S894. | Abstract |
| Cunha Neves, J. A., et al. (2022). "IMPROVING ENDOSCOPY UNIT THROUGHPUT USING AN AUTOMATED CYBER-PHYSICAL MONITORING SYSTEM : A PILOT STUDY GREEN ENDOSCOPY TO REDUCE COGENERATED BY ENDOSCOPIC WASTE - GECO." Gastrointestinal Endoscopy **95**(6 Supplement): AB128. | Abstract |
| Cunha Neves, J. A., et al. (2023). "Targeted intervention to achieve waste reduction in gastrointestinal endoscopy." Gut **72**(2): 306-313. | Duplicate |
| Curtis, A., et al. (2021). "Remote orthopaedic clinics during covid-19: Lessons for a sustainable future." British Journal of Surgery **108**(SUPPL 6): vi129. | Abstract |
| Davies, J. F., et al. (2023). "Operation clean up: A model for eco-leadership and sustainability implementation." Anaesthesia and Intensive Care **51**(2): 88-95. | Specialty |
| De Jong, D., et al. (2022). "TOWARDS A GREENER ENDOSCOPY ROOM: RECYCLING PLASTIC WASTE." United European Gastroenterology Journal **10**(Supplement 8): 1082-1083. | Abstract |
| De Rydt, F., et al. (2020). "Sevoflurane consumption with the How-i ventilator in two versions of automatic gas control algorithms and two settings of manually controlled anesthesia : an economic and ecological assessment." Acta Anaesthesiologica Belgica **71**: 15-20. | Specialty |
| Dengiz, A. O., et al. (2021). "A goal programming approach for multi objective, multi-trips and time window routing problem in home health care service." Journal of the Faculty of Engineering and Architecture of Gazi University **36**(4): 2167-2181. | Language |
| Do Thi, H. T., et al. (2021). "Applicability of Membranes in Protective Face Masks and Comparison of Reusable and Disposable Face Masks with Life Cycle Assessment." Sustainability **13**(22). | Specialty |
| Donahue, L. M., et al. (2020). "A Comparative Carbon Footprint Analysis of Disposable and Reusable Vaginal Specula." Obstetrical and Gynecological Survey **75**(6): 352-354. | Study design |
| Duane, B., et al. (2014). "Carbon mitigation, patient choice and cost reduction - triple bottom line optimisation for health care planning." Public Health **128**(10): 920-924. | Specialty |
| Dullet, N. W., et al. (2017). "Impact of a University-Based Outpatient Telemedicine Program on Time Savings, Travel Costs, and Environmental Pollutants." Value in Health **20**(4): 542-546. | Specialty |
| Dunbar-Reid, K. and E. Buikstra (2017). "Waste reduction in haemodialysis: a multicentre quality activity." Renal Society of Australasia Journal **13**(2): 45-52. | CE data NR |
| Eckelman, M., et al. (2012). "Comparative Life Cycle Assessment of Disposable and Reusable Laryngeal Mask Airways." Anesthesia and Analgesia **114**(5): 1067-1072. | Specialty |
| Edison, M., et al. (2019). "Prospective clinical, cost analysis and environmental impact of a clinician-led virtual ureteric colic treatment decision pathway." Journal of Clinical Urology **12**(1 Supplement): 79-80. | Abstract |
| Enos, M., et al. (2014). "Carbon footprints of an in-centre haemodialysis device, the nxstage system one and the vivia haemodialysis system." Nephrology Dialysis Transplantation **29**(SUPPL. 3): iii219. | Abstract |
| Essa, H., et al. (2021). "One year outcomes of heart failure multispecialty multidisciplinary team virtual meetings." European Heart Journal **42**(SUPPL 1): 971. | Abstract |
| Farrell, E. and D. Smyth (2021). "The environmental impact of personal protective equipment in a pre and post COVID era in the ENT clinic." European Archives of Oto-Rhino-Laryngology **278**(12): 5051-5058. | CE data NR |
| Fatima, R., et al. (2022). "PREVENTING UNNECESSARY CT CORONARY ANGIOGRAPHY BY UTILISING PREVIOUS CT THORACIC IMAGING: A RETROSPECTIVE ANALYSIS." Heart **108**(Supplement 2): A12. | Abstract |
| Ford, B., et al. (2022). "Reducing Single-Use Surgical Instruments During Laparoscopic Appendicectomy: Using Sustainable Quality Improvement as a Catalyst to Encourage Wider Behavioural Change in a Surgical Department." British Journal of Surgery **109**(Supplement 6): vi5-vi6. | Abstract |
| Fort, E. J., et al. (2021). "Social and environmental benefits of virtual fracture clinics in trauma and orthopaedic surgery: Reduced patient Travel Time." British Journal of Surgery **108**(SUPPL 6): vi124. | Abstract |
| Freihoefer, K., et al. (2018). "Setting the Stage: A Comparative Analysis of an Onstage/Offstage and a Linear Clinic Modules." Herd-Health Environments Research & Design Journal **11**(2): 89-103. | CE data NR |
| Freund, J., et al. (2022). "Environmental considerations in the selection of medical staplers: A comparative life cycle assessment." Journal of Cleaner Production **371**. | CE data NR |
| Frick, M., et al. (2022). "The Environmental Impact of Telemedicine in a Radiation Oncology Clinic." American Journal of Clinical Oncology: Cancer Clinical Trials **45**(9): S60-S61. | Abstract |
| Friedericy, H. J., et al. (2022). "Reducing the Environmental Impact of Sterilization Packaging for Surgical Instruments in the Operating Room: A Comparative Life Cycle Assessment of Disposable versus Reusable Systems." Sustainability **14**(1). | Specialty |
| Furlan, L., et al. (2023). "The environmental cost of unwarranted variation in the use of magnetic resonance imaging and computed tomography scans." European Journal of Internal Medicine **111**: 47-53. | Intervention |
| Furlan, L., et al. (2023). "The environmental cost of unwarranted variation in the use of magnetic resonance imaging and computed tomography scans." European Journal of Internal Medicine **111**: 47-53. | Study design |
| Gerris, J., et al. (2014). "Self-operated endo-vaginal tele-monitoring versus traditional monitoring of ovarian stimulation in ART: Prospective randomized trial." Human Reproduction **29**(SUPPL. 1): i112. | Abstract |
| Gil-Candel, M., et al. (2023). "Developing a telepharmacy programme with home medication dispensing and informed delivery in a tertiary hospital: description of the model and analysis of the results." European Journal of Hospital Pharmacy **30**(2): 107-112. | Specialty |
| Goel, H., et al. (2021). "Improving productivity, costs and environmental impact in International Eye Health Services: using the 'Eyefficiency' cataract surgical services auditing tool to assess the value of cataract surgical services." BMJ open ophthalmology **6**(1): e000642. | Study design |
| Gough, V., et al. (2022). "Laparoscopic Cholecystectomy-Can we make it both Greener and cheaper?" British Journal of Surgery **109**(Supplement 9): ix19-ix20. | Abstract |
| Griffing, E. and M. Overcash (2023). "Reusable and Disposable Incontinence Underpads: Environmental Footprints as a Route for Decision Making to Decarbonize Health Care." Journal of Nursing Care Quality **38**(3): 278-285. | Specialty |
| Grimmond, T. and S. Reiner (2012). "Impact on carbon footprint: A life cycle assessment of disposable versus reusable sharps containers in a large US hospital." Waste Management and Research **30**(6): 639-642. | Specialty |
| Grimmond, T. R., et al. (2021). "Before/after intervention study to determine impact on life-cycle carbon footprint of converting from single-use to reusable sharps containers in 40 UK NHS trusts." Bmj Open **11**(9). | Specialty |
| Hainc, N., et al. (2020). ""Green Fingerprint" Project: Evaluation of the Power Consumption of Reporting Stations in a Radiology Department." Academic Radiology **27**(11): 1594-1600. | CE data NR |
| Hernandez-de-Anda, M. T., et al. (2023). "Environmental impacts of a Mexican haemodialysis unit through LCA." Journal of Cleaner Production **384**. | Study design |
| Hicks, A. L., et al. (2016). "Environmental impacts of reusable nanoscale silver-coated hospital gowns compared to single-use, disposable gowns." Environmental Science-Nano **3**(5): 1124-1132. | Specialty |
| Hogan, D., et al. (2022). "The carbon footprint of single-use flexible cystoscopes compared to reusable cystoscopes." European Urology Open Science **39**(Supplement 1): S89. | Abstract |
| Hong, Z., et al. (2022). "One step forward to sustainability: The carbon footprint of cataract surgery in Australia." Clinical & experimental ophthalmology. | Study design |
| Hong, Z., et al. (2023). "One step forward to sustainability: The carbon footprint of cataract surgery in Australia." Clinical and Experimental Ophthalmology **51**(2): 180-182. | Study design |
| Hu, X., et al. (2021). "The carbon footprint of general anaesthetics: A case study in the UK." Resources, Conservation & Recycling **167**: N.PAG-N.PAG. | Specialty |
| Hubert, J., et al. (2022). "Carbon emissions during elective coronary artery bypass surgery, a single center experience." Journal of Clinical Anesthesia **80**: 110850. | Study design |
| Hunt, F. J. N. and A. Wilkinson (2021). "Carbon footprint analysis of the salford lung Study (asthma): A susqi analysis." Thorax **76**(SUPPL 1): A190. | Abstract |
| Ito, Y., et al. (2021). "Environmental impact of anaesthetic gases at a tertiary hospital: a comparison of subspecialties and analysis of anaesthetic choices." Anaesthesia **76**: 103-103. | Abstract |
| Jain, M. and V. Agrawal (2023). "Making endoscopy practice environmentally sustainable-Early experience from Central India." Indian Journal of Gastroenterology. | Study design |
| Jamal, H., et al. (2021). "Non-sterile examination gloves and sterile surgical gloves: which are more sustainable?" Journal of Hospital Infection **118**: 87-95. | Specialty |
| Janson, C., et al. (2022). "The carbon footprint of respiratory treatments in Europe and Canada: an observational study from the CARBON programme." European Respiratory Journal **60**(2): 2102760. | Intervention |
| Jemai, J. and B. Sarkar (2019). "Optimum Design of a Transportation Scheme for Healthcare Supply Chain Management: The Effect of Energy Consumption." Energies **12**(14). | Specialty |
| Jemai, J., et al. (2020). "Environmental effect for a complex green supply-chain management to control waste: A sustainable approach." Journal of Cleaner Production **277**. | CE data NR |
| Khan, B. A., et al. (2019). "Greenhouse gas emission from small clinics solid waste management scenarios in an urban area of an under developing country: A life cycle perspective." Journal of the Air & Waste Management Association (Taylor & Francis Ltd) **69**(7): 823-833. | Specialty |
| Khatkar, H., et al. (2022). "The environmental impact of orthopaedic surgery: assessing strategies for change." British journal of hospital medicine (London, England : 2005) **83**(11): 1-4. | Study design |
| Kim, S. and F. Roodt (2023). "Almost 30% reduction in carbon footprint using volatile anaesthesia - a quality improvement project introducing low-flow anaesthesia in a regional hospital." Southern African Journal of Anaesthesia and Analgesia **29**(1): S4-S5. | Abstract |
| King, J., et al. (2022). "Towards NHS Zero: greener gastroenterology and the impact of virtual clinics on carbon emissions and patient outcomes. A multisite, observational, cross-sectional study." Frontline Gastroenterology. | Duplicate |
| Kokare, S., et al. (2022). "A comparative life cycle assessment of stretchable and rigid electronics: a case study of cardiac monitoring devices." International Journal of Environmental Science & Technology (IJEST) **19**(4): 3087-3102. | Specialty |
| Koo, K., et al. (2021). "The cost of convenience: Estimating the environmental impact of single-use and reusable flexible cystoscopes." Journal of Urology **206**(SUPPL 3): e683-e684. | Abstract |
| Kuvadia, M., et al. (2020). "'Green-gional' anesthesia: The non-polluting benefits of regional anesthesia to decrease greenhouse gases and attenuate climate change." Regional Anesthesia and Pain Medicine **45**(9): 744-745. | Study design |
| Kwakman, J. A., et al. (2022). "Single-use duodenoscopes compared with reusable duodenoscopes in patients carrying multidrug-resistant microorganisms: A break-even cost analysis." Endoscopy International Open **11**(6): E571-E580. | CE data NR |
| Lalman, C., et al. (2023). "To Dispose or to Reuse? Analyzing the Life Cycle Impacts and Costs of Disposal, Sterilization, and Reuse of Electrophysiological Catheters." Sustainability **15**(6). | Specialty |
| Lee, S. M. and D. Lee (2022). "Developing Green Healthcare Activities in the Total Quality Management Framework." International Journal of Environmental Research and Public Health **19**(11). | CE data NR |
| Lehtimaki, L., et al. (2020). "Minimising the environmental impact of inhaled therapies." European Respiratory Journal **318**(6): 2000721. | Study design |
| Lichter, K. E., et al. (2022). "Transitioning to Environmentally Sustainable, Climate-Smart Radiation Oncology Care." International Journal of Radiation Oncology Biology Physics **113**(5): 915-924. | Study design |
| Lippert, J. F., et al. (2014). "A Pilot Study to Determine Medical Laser Generated Air Contaminant Emission Rates for a Simulated Surgical Procedure." Journal of Occupational and Environmental Hygiene **11**(6): D69-D76. | Specialty |
| Lodi, C. A., et al. (2020). "The environmental impact of disposables in a new configuration of hemodialysis (HD) system." Nephrology Dialysis Transplantation **35**(SUPPL 3): iii1419. | CE data NR |
| Luo, H. Y., et al. (2021). "An ACO-based heuristic approach for a route and speed optimization problem in home health care with synchronized visits and carbon emissions." Soft Computing **25**(23): 14673-14696. | Specialty |
| MacNeill, A. J., et al. (2017). "The impact of surgery on global climate: a carbon footprinting study of operating theatres in three health systems." Lancet Planetary Health **1**(9): E381-E388. | Specialty |
| Mak, K., et al. (2023). "Reducing the use of ethyl chloride spray in obstetrics with a cool new idea: a quality improvement project." International Journal of Obstetric Anesthesia **54**(Supplement 1): 103729. | Abstract |
| Maria, M. S., et al. (2022). "Health care in rural areas: proposal of a new telemedicine program assisted from the reference health centers, for a sustainable digitization and its contribution to the carbon footprint reduction." Heliyon **8**(7). | Specialty |
| Marwick, T. H. and J. Buonocore (2011). "Environmental impact of cardiac imaging tests for the diagnosis of coronary artery disease." Heart **97**(14): 1128-1131. | CE data NR |
| Materazzo, M., et al. (2022). "MAINTAINING GOOD PRACTICE IN BREAST CANCER MANAGEMENT AND REDUCING THE CARBON FOOTPRINT OF CARE: STUDY PROTOCOL AND PRELIMINARY RESULTS." World Cancer Research Journal **9**. | CE data NR |
| McAlister, S., et al. (2023). "Carbon emissions and hospital pathology stewardship: a retrospective cohort analysis." Internal Medicine Journal **53**(4): 584-589. | Specialty |
| McCarthy, C. J., et al. (2014). ""EcoRadiology"-Pulling the plug on wasted energy in the radiology department." Academic Radiology **21**(12): 1563-1566. | Duplicate |
| McGain, F., et al. (2012). "A Life Cycle Assessment of Reusable and Single-Use Central Venous Catheter Insertion Kits." Anesthesia and Analgesia **114**(5): 1073-1080. | Specialty |
| McGain, F., et al. (2016). "Hospital steam sterilizer usage: could we switch off to save electricity and water?" Journal of Health Services Research & Policy **21**(3): 166-171. | Specialty |
| McGain, F., et al. (2017). "Financial and environmental costs of reusable and single-use anaesthetic equipment." British Journal of Anaesthesia **118**(6): 862-869. | Specialty |
| McPherson, B., et al. (2019). "The impact on life cycle carbon footprint of converting from disposable to reusable sharps containers in a large US hospital geographically distant from manufacturing and processing facilities." Peerj **7**. | Specialty |
| Meierling, S. (2023). "A race we must win: reuse of disposable products in the operating theater The example of a nearly failed recycling concept." Chirurgie **94**(3): 216-219. | Language |
| Michard, F., et al. (2023). "Pulse contour techniques for perioperative hemodynamic monitoring: A nationwide carbon footprint and cost estimation." Anaesthesia Critical Care and Pain Medicine **42**(5): 101239. | Specialty |
| Moses, R., et al. (2016). "Reducing the carbon footprint in a regional long term ventilation service with the use of remote monitoring." Thorax **71**(Supplement 3): A187. | Abstract |
| Moses, R., et al. (2019). "Reducing the carbon footprint in a regional long term ventilation service with the use of remote monitoring." Physiotherapy (United Kingdom) **105**(Supplement 1): e209-e210. | Abstract |
| Moussa, G., et al. (2022). "Environmental Effect of Fluorinated Gases in Vitreoretinal Surgery: A Multicenter Study of 4,877 Patients." American Journal of Ophthalmology **235**: 271-279. | Duplicate |
| Moussa, G., et al. (2023). "The use of fluorinated gases and quantification of carbon emission for common vitreoretinal procedures." Eye (Basingstoke) **37**(7): 1405-1409. | Duplicate |
| Mushtaq, M. H., et al. (2022). "Environmental Performance of Alternative Hospital Waste Management Strategies Using Life Cycle Assessment (LCA) Approach." Sustainability **14**(22). | Specialty |
| Nagai, K. and N. Itsubo (2022). "Environmental Impact of Care for End-stage Kidney Disease on the Earth and Humans." JMA journal **5**(1): 109-113. | Intervention |
| Nagasaki, K., et al. (2023). "The environmental impact of inhaler replacement: A carbon footprint and economic calculation of the National Database of Health Insurance Claims in Japan." Journal of General and Family Medicine. | Intervention |
| Nair, S. and J. Gautier (2019). "Home hemodialysis (HHD) with low dialysate volume (LDV)-the green benefits." Nephrology Dialysis Transplantation **34**(Supplement 1): a587. | Abstract |
| Nair, S. and J. gautier (2019). "MON-112 Home Haemodialysis (HHD) with low dialysate volume (LDV) - The green benefit." Kidney International Reports **4**(7 Supplement): S350. | Abstract |
| Namburar, S., et al. (2022). "Estimating the environmental impact of disposable endoscopic equipment and endoscopes." Gut **71**(7): 1326-1331. | CE data NR |
| Neves, J. A. C., et al. (2022). "Green Endoscopy to reduce CO2eq generated by endoscopic waste-The GECO2eq interventional study." British Journal of Surgery **109**(Supplement 5): v3. | Abstract |
| Nuijts, R. M. M. A., et al. (2022). "The carbon footprint of cataract surgery in a Dutch University hospital." Acta Ophthalmologica **100**(Supplement 268): 38. | Abstract |
| Olmos, M., et al. (2023). "Evaluating the potential impact of spinal anesthesia use in lumbar surgery on global healthcare cost and climate change." Brain and Spine **3**: 101754. | Intervention |
| Park, E. A. and K. C. LaMattina (2020). "Economic and Environmental Impact of Single-use Plastics at a Large Ophthalmology Outpatient Service." Journal of glaucoma. | Intervention |
| Perez Diaz, P., et al. (2021). "Carbon footprint as a marker of environmental impact in patients included in a remote monitoring pacemaker programme." European Heart Journal **42**(SUPPL 1): 409. | Abstract |
| Pernigotti, D., et al. (2021). "Reducing carbon footprint of inhalers: Analysis of climate and clinical implications of different scenarios in five European countries." BMJ Open Respiratory Research **8**(1): e001071. | Specialty |
| Plusa, T. and A. M. Badowska-Kozakiewicz (2020). "Carbon footprint of inhalers in COPD therapy in 2018 and 2019 in Poland in response to the Kigali amendment." Polski merkuriusz lekarski : organ Polskiego Towarzystwa Lekarskiego **48**(288): 391-393. | Specialty |
| Pollard, A. S., et al. (2014). "The carbon footprint of acute care: How energy intensive is critical care?" Public Health **128**(9): 771-776. | Intervention |
| Power, B., et al. (2021). "Analyzing the Carbon Footprint of an Intravitreal Injection." Journal of ophthalmic & vision research **16**(3): 367-376. | Study design |
| Raila, E. M. and D. O. Anderson (2017). "Black carbon emission reduction strategies in healthcare industry for effective global climate change management." Waste Management & Research: The Journal for a Sustainable Circular Economy **35**(4): 416-425. | Specialty |
| Ramani, S. (2022). "A Comparison Study Quantifying Environmental Impact of All Surgical Modalities of Hysterectomies." Journal of Minimally Invasive Gynecology **29**(11 Supplement): S12. | Abstract |
| Renton, D., et al. (2018). "Reprocessed single-use devices in laparoscopy: assessment of cost, environmental impact, and patient safety." Surgical Endoscopy **32**(10): 4310-4313. | Study design |
| Reynolds, R., et al. (2019). "The carbon footprint of fluorescein angiography compared to OCT angiography." Investigative Ophthalmology and Visual Science **60**(9). | Abstract |
| Ribes-Iborra, J., et al. (2022). "Improving perioperative management of surgical sets for trauma surgeries: the 4S approach." BMC health services research **22**(1): 1298. | CE data NR |
| Rizan, C., et al. (2021). "The carbon footprint of waste streams in a UK hospital." Journal of Cleaner Production **286**. | Specialty |
| Rizan, C., et al. (2022). "Life cycle assessment and life cycle cost of repairing surgical scissors." International Journal of Life Cycle Assessment **27**(6): 780-795. | Specialty |
| Rizan, C., et al. (2022). "Minimising carbon and financial costs of steam sterilisation and packaging of reusable surgical instruments." British Journal of Surgery **109**(2): 200-210. | Specialty |
| Robert, B., et al. (2019). "Plastic waste reduction in different peritoneal dialysis strategies: The impact of disposable choice on carbon footprint." Nephrology Dialysis Transplantation **34**(Supplement 1): a237. | Abstract |
| Rochon, M., et al. (2023). "Image-based digital post-discharge surveillance in England: measuring patient enrolment, engagement, clinician response times, surgical site infection, and carbon footprint." Journal of Hospital Infection **133**: 15-22. | CE data NR |
| Roy, W., et al. (2023). "Tele-visits for GERD: "Ecofriendly, efficient and effective"." Journal of Gastroenterology and Hepatology (Australia) **38**(6): 905-909. | CE data NR |
| Saitch, H., et al. (2022). "A comparison of the sustainable value of single-use direct laryngoscopes versus re-usable videolaryngoscopes." British Journal of Anaesthesia **128**(5): e331. | Abstract |
| Sanchez SA, Eckelman MJ, Sherman JD. Environmental and economic comparison of reusable and disposable blood pressure cuffs in multiple clinical settings. Resources, Conservation and Recycling. 2020;155:104643. | Specialty |
| Schleser, A., et al. (2016). "The impact of disposables towards more eco-friendly and less costly haemodialysis." Nephrology Dialysis Transplantation **31**(SUPPL. 1): i494. | Abstract |
| Sellars, H., et al. (2020). "Video consultation for new colorectal patients." Colorectal disease : the official journal of the Association of Coloproctology of Great Britain and Ireland **22**(9): 1015-1021. | Specialty |
| Serra, C. M., et al. (2022). "Impact on the reduction of CO2 emissions due to the use of telemedicine." Scientific Reports **12**(1). | Specialty |
| Sharma, S., et al. (2022). "Environmental Impact of Ambulatory Telehealth Use by a Statewide University Health System During COVID-19." Telemedicine and E-Health. | Specialty |
| Simpson, I. and V. Bhandari (2022). "CONSIDERING THE SUSTAINABILITY IMPACT OF CONNECTED INHALERS IN THE TREATMENT OF ASTHMA." ONdrugDelivery **2022**(139): 42-48. | Study design |
| Smith, A. J. B., et al. (2013). "The carbon footprint of behavioural support services for smoking cessation." Tobacco Control **22**(5): 302-307. | Intervention |
| Snigdha, et al. (2023). "Environmental footprints of disposable and reusable personal protective equipment-a product life cycle approach for body coveralls." Journal of Cleaner Production **394**. | Specialty |
| Somner, J. E. A., et al. (2008). "Surgical scrubbing: can we clean up our carbon footprints by washing our hands?" Journal of Hospital Infection **70**(3): 212-215. | Specialty |
| Southorn, T., et al. (2013). "Reducing the carbon footprint of the operating theatre: a multicentre quality improvement report." Journal of perioperative practice **23**(6): 144-146. | CE data NR |
| Sullivan, G. A., et al. (2023). "Operating Room Recycling: Opportunities to Reduce Carbon Emissions Without Increases in Cost." Journal of Pediatric Surgery. | Specialty |
| Tan, E. and T. Jennings (2015). "Impact of virtual clinics and tele-health medicine in rural nephrology, New Zealand: A Waikato perspective." Nephrology **20**(SUPPL. 3): 62. | Abstract |
| Thiel, C. L., et al. (2016). "Environmental life cycle assessment and costs of phacoemulsification at aravind eye hospital in pondicherry, India." Investigative Ophthalmology and Visual Science **57**(12): 5575. | Abstract |
| Thiel, C. L., et al. (2017). "Cataract surgery and environmental sustainability: Waste and lifecycle assessment of phacoemulsification at a private healthcare facility." Journal of Cataract and Refractive Surgery **43**(11): 1391-1398. | Study design |
| Thiel, C. L., et al. (2020). "Resource use and carbon footprint of inpatient stays in a us hospital." Journal of General Internal Medicine **35**(SUPPL 1): S258. | Abstract |
| Tsang, D., et al. (2022). "Evaluating and reducing carbon footprint in urological surgery." Journal of Clinical Urology **15**(1 Supplement): 51-52. | Abstract |
| Unger, S. and A. Landis (2016). "Assessing the environmental, human health, and economic impacts of reprocessed medical devices in a Phoenix hospital's supply chain." Journal of Cleaner Production **112**: 1995-2003. | Specialty |
| Unger, S. R., et al. (2017). "Do single-use medical devices containing biopolymers reduce the environmental impacts of surgical procedures compared with their plastic equivalents?" Journal of Health Services Research and Policy **22**(4): 218-225. | CE data NR |
| Usmani, O. S. and M. L. Levy (2023). "Effective respiratory management of asthma and COPD and the environmental impacts of inhalers." npj Primary Care Respiratory Medicine **33**(1): 24. | Study design |
| Vali, M., et al. (2022). "Care process optimization in a cardiovascular hospital: an integration of simulation-optimization and data mining." Annals of Operations Research **318**(1): 685-712. | CE data NR |
| Van Leeuwen, R., et al. (2020). "Reducing waste and carbon footprint of intravitreal injections." Acta Ophthalmologica **98**(SUPPL 264): 41-42. | Abstract |
| Vidal-Alaball, J., et al. (2019). "Impact of a Telemedicine Program on the Reduction in the Emission of Atmospheric Pollutants and Journeys by Road." International Journal of Environmental Research and Public Health **16**(22). | Specialty |
| Vozzola, E., et al. (2018). "Environmental considerations in the selection of isolation gowns: A life cycle assessment of reusable and disposable alternatives." American Journal of Infection Control **46**(8): 881-886. | Specialty |
| Vozzola, E., et al. (2020). "An Environmental Analysis of Reusable and Disposable Surgical Gowns." Aorn Journal **111**(3): 315-325. | Specialty |
| Wang, A. Y., et al. (2022). "Assessing the Environmental Carbon Footprint of Spinal versus General Anesthesia in Single-Level Transforaminal Lumbar Interbody Fusions." World Neurosurgery **163**: e199-e206. | Specialty |
| Wang, E. Y., et al. (2021). "Environmental emissions reduction of a preoperative evaluation center utilizing telehealth screening and standardized preoperative testing guidelines." Resources, Conservation and Recycling **171**: 105652. | Specialty |
| Wanigasooriya, K., et al. (2022). "Index telephone two-week wait lower gastrointestinal clinics as a substitute to face to face outpatient appointments during the COVID-19 pandemic." Colorectal Disease **24**(SUPPL 1): 167. | Abstract |
| Wilkinson, A. J. K., et al. (2019). "Costs of switching to low global warming potential inhalers. An economic and carbon footprint analysis of NHS prescription data in England." Bmj Open **9**(10): e028763. | Specialty |
| Williams, L. M., et al. (2023). "Circular External Fixator Removal in the Outpatient Clinic Using Regional Anaesthesia: A Pilot Study of a Novel Approach." Strategies in Trauma and Limb Reconstruction **18**(1): 7-11. | CE data NR |
| Wood, K. F., et al. (2020). "Patient accessibility, cost savings and environmental impact of providing specialist diabetes support by telemedicine clinic in a remote and rural setting." Diabetic Medicine **37**(SUPPL 1): 168-169. | Abstract |
| Woodcock, A., et al. (2022). "Effects of switching from a metered dose inhaler to a dry powder inhaler on climate emissions and asthma control: Post-hoc analysis." Thorax: thoraxjnl-2021-218088. | Study design |
| Woods, D., et al. (2013). "Comparison of the environmental impact of commonly used surgical approaches to hysterectomy." Gynecologic Oncology **130**(1): e143. | Abstract |
| Wormer, B. A., et al. (2013). "The Green Operating Room: Simple Changes to Reduce Cost and Our Carbon Footprint." American Surgeon **79**(7): 666-671. | Specialty |
| Wyssusek, K., et al. (2022). "Greenhouse gas reduction in anaesthesia practice: a departmental environmental strategy." Bmj Open Quality **11**(3). | Specialty |
| Yong, K. K., et al. (2023). "Rationalising the use of specimen pots following colorectal polypectomy: A small step towards greener endoscopy." Frontline Gastroenterology **14**(4): 295-299. | Duplicate |
| Zhang, X., et al. (2022). "Carbon footprinting for hospital care pathways based on routine diagnosis‐related group (DRG) accounting data in Germany: An application to acute decompensated heart failure." Journal of Industrial Ecology **26**(4): 1528-1542. | Intervention |
| Zhao, X., et al. (2022). "How sustainable are the biodegradable medical gowns via environmental and social life cycle assessment?" Journal of Cleaner Production **380**. | Specialty |
| Ziya-Gorabi, F., et al. (2022). "A new fuzzy tri-objective model for a home health care problem with green ambulance routing and congestion under uncertainty." Expert Systems with Applications **201**. | Specialty |
| Bargallo-Rocha, J. E., et al. (2017). "The impact of the use of intraoperative radiotherapy on costs, travel time and distance for women with breast cancer in the Mexico City Metropolitan Area." Journal of Surgical Oncology 116(6): 683-689. | CE data NR |
| Beige, J., et al. (2023). "Green dialysis Effects of sustainable techniques on the real carbon footprint." Nephrologie. | Language |
| Bhanvadia, R. R., et al. (2022). "Safety and Feasibility of Telehealth Only Preoperative Evaluation Before Minimally Invasive Robotic Urologic Surgery." Journal of Endourology 36(8): 1070-1076. | CE data NR |
| Bickhardt, J., et al. (2022). "Reduction of greenhouse gas emissions by inhaler choice in the therapy of asthma and COPD patients." Pneumologie 76(05): 321-329. | Language |
| Bonsall, A. (2021). "Unleashing carbon emissions savings with regular teledermatology clinics." Clinical and Experimental Dermatology 46(3): 574-575. | Abstract |
| Bringier, R., et al. (2023). "An integrated environmental, economic, and clinician satisfaction comparison between single-use and reusable flexible bronchoscopes for tracheal intubation." British Journal of Anaesthesia 131(1): E4-E7. | SD |
| Burns, C. L., et al. (2017). "Cost analysis of a speech pathology synchronous telepractice service for patients with head and neck cancer." Head and Neck-Journal for the Sciences and Specialties of the Head and Neck 39(12): 2470-2480. | CE data NR |
| Checcucci, E., et al. (2021). "Implementing telemedicine for the management of benign urologic conditions: a single centre experience in Italy." World Journal of Urology 39(8): 3109-3115. | CE data NR |
| Dohmen, J., et al. (2023). "Recycling of Disposable Surgical Instruments - Is It Worth It?" Zentralblatt Fur Chirurgie 148(04): 329-336. | Language |
| Farooq, F., et al. (2022). "Telehealth Service Utilization for Gynae & Obstetrics During COVID-19 Pandemic in Lahore, Pakistan." Annals of King Edward Medical University Lahore Pakistan 28(3): 335-342 | CE data NR |
| George, K., et al. (2022). "Utility and patient acceptance of telemedicine in nephrology." Journal of Nephrology 35(9): 2325-2331. | CE data NR |
| Haripriya, A., et al. (2023). "Changing operating room practices: the effect on postoperative endophthalmitis rates following cataract surgery." British Journal of Ophthalmology 107(6): 780-785. | CE data NR |
| Hlavin, C., et al. (2023). "Clinical Outcomes and Hospital Utilization Among Patients Undergoing Bariatric Surgery With Telemedicine Preoperative Care." Jama Network Open 6(2): 11. | CE data NR |
| Hogan, D. and D. B. Hennessey (2023). "Re: The Carbon Footprint of Single-Use Flexible Cystoscopes Compared with Reusable Cystoscopes-Letter by Rizan et al (2022): Clarification of Methods Due to Apparent Misinterpretation." Journal of Endourology: 2. | SD |
| Lambooy, S., et al. (2021). "Telemedicine for Outpatient Care of Kidney Transplant and CKD Patients." Kidney International Reports 6(5): 1265-1272. | CE data NR |
| Lee, A. W. L., et al. (2021). "Life cycle assessment of single-use surgical and embedded filtration layer (EFL) reusable face mask." Resources Conservation and Recycling 170: 12. | Speciality |
| Liang, S. Y., et al. (2022). "The effect of COVID-19 on telehealth: Next steps in a post-pandemic life." International Journal of Gynecology & Obstetrics 159(3): 996-997. | CE data NR |
| Margolin, E. J., et al. (2021). "Telemedicine in management of genitourinary malignancies: Patient and physician perspectives." Urologic Oncology-Seminars and Original Investigations 39(8): 480-486. | CE data NR |
| Maria, M. S., et al. (2022). "Health care in rural areas: proposal of a new telemedicine program assisted from the reference health centers, for a sustainable digitization and its contribution to the carbon footprint reduction." Heliyon 8(7): 6. | Speciality |
| Masino, C., et al. (2010). "The Impact of Telemedicine on Greenhouse Gas Emissions at an Academic Health Science Center in Canada." Telemedicine Journal and E-Health 16(9): 273-276. | Speciality |
| McAlarnen, L. A., et al. (2021). "Virtual visits among gynecologic oncology patients during the COVID-19 pandemic are accessible across the social vulnerability spectrum." Gynecologic Oncology 162(1): 4-11. | CE data NR |
| Meiklejohn, D. A. and V. M. Chavarri (2021). "Cold Technique in Adult Tonsillectomy Reduces Waste and Cost." Ent-Ear Nose & Throat Journal 100(5_SUPPL): 427S-430S. | CE data NR |
| Meissner, M., et al. (2023). "Evaluating the Environmental Impact of Single-Use and Multi-Use Surgical Staplers with Staple Line Buttressing in Laparoscopic Bariatric Surgery." Risk Management and Healthcare Policy 16: 1423-1433. | CE data NR |
| Mojdehbakhsh, R. P., et al. (2022). "The long game: Telemedicine patient satisfaction metrics and methods of recurrence detection for gynecologic cancer patients throughout the initial year of the COVID-19 pandemic." Gynecologic Oncology Reports 42: 5. | CE data NR |
| Morris, M. I. R. and A. Hicks (2022). "Life cycle assessment of stainless-steel reusable speculums versus disposable acrylic speculums in a university clinic setting: a case study." Environmental Research Communications 4(2): 13. | Speciality |
| Nardelli, L., et al. (2023). "Incremental peritoneal dialysis allows to reduce the time spent for dialysis, glucose exposure, economic cost, plastic waste and water consumption." Journal of Nephrology 36(2): 263-273. | CE data NR |
| Ong, C. S. H., et al. (2021). "Implementation of a Ureteric Colic Telemedicine Service: A Mixed Methods Quality Improvement Study." Urology 147: 14-20. | CE data NR |
| O'Reilly, D., et al. (2021). "Virtual oncology clinics during the COVID-19 pandemic." Irish Journal of Medical Science 190(4): 1295-1301. | CE data NR |
| Patel, S., et al. (2021). "Climate Change Impact of Virtual Urology Meetings." European Urology 80(1): 121-122. | Abstract |
| Pollard, A. S., et al. (2013). "Mainstreaming Carbon Management in Healthcare Systems: A Bottom-Up Modeling Approach." Environmental Science & Technology 47(2): 678-686. | Speciality |
| Pradhan, R., et al. (2021). "Virtual phone clinics in orthopaedics: evaluation of clinical application and sustainability." Bmj Open Quality 10(4): 7. | CE data NR |
| Quam, N., et al. (2022). "Perception of Telehealth During the COVID-19 Pandemic Among Survivors of Gynecologic Cancer." Oncologist 27(6): 512-515. | CE data NR |
| Rajeev, A., et al. (2023). "Patient Outcomes of Virtual Foot and Ankle Telephone Clinics During COVID-19 Pandemic: 1 Year Experience." Journal of Foot & Ankle Surgery 62(3): 571-575. | CE data NR |
| Ramdas, Y., et al. (2020). "First Intraoperative Radiation Therapy Center in Africa: First 2 Years in Operation, Including COVID-19 Experiences." Jco Global Oncology 6: 1696-1703. | CE data NR |
| Richter, H., et al. (2020). "The carbon footprint of anaesthesia How the choice of volatile anaesthetic affects the CO2 emissions of a department of anaesthesiology." Anasthesiologie & Intensivmedizin 61: 154-161. | Speciality |
| Shiff, B., et al. (2021). "Patient Satisfaction With Telemedicine Appointments in an Academic Andrology-focused Urology Practice During the COVID-19 Pandemic." Urology 153: 35-41. | CE data NR |
| van der Pol, M. and L. McKenzie (2010). "Costs and benefits of tele-endoscopy clinics in a remote location." Journal of Telemedicine and Telecare 16(2): 89-94. | CE data NR |
| Yadav, N. and A. Tanksale (2023). "A multi-objective approach for reducing Patient's inconvenience in a generalized home healthcare delivery setup." Expert Systems with Applications 219. | CE data NR |
| Zhou, J., et al. (2021). "A novel two-phase approach for the bi-objective simultaneous delivery and pickup problem with fuzzy pickup demands." International Journal of Production Economics 234. | Speciality |
